# Distribution of Pico- and Nanosecond Motions in Disordered Proteins from Nuclear Spin Relaxation

**DOI:** 10.1016/j.bpj.2015.06.069

**Published:** 2015-09-01

**Authors:** Shahid N. Khan, Cyril Charlier, Rafal Augustyniak, Nicola Salvi, Victoire Déjean, Geoffrey Bodenhausen, Olivier Lequin, Philippe Pelupessy, Fabien Ferrage

**Affiliations:** 1 Département de Chimie, École Normale Supérieure-PSL Research University, Paris, France; 2 Sorbonne Universités, UPMC Univ Paris 06, LBM, Paris, France; 3 Centre National de la Recherche Scientifique, UMR 7203 LBM, Paris, France; 4 Institut des Sciences et Ingénierie Chimiques, École Polytechnique Fédérale de Lausanne, BCH, Lausanne, Switzerland

## Abstract

Intrinsically disordered proteins and intrinsically disordered regions (IDRs) are ubiquitous in the eukaryotic proteome. The description and understanding of their conformational properties require the development of new experimental, computational, and theoretical approaches. Here, we use nuclear spin relaxation to investigate the distribution of timescales of motions in an IDR from picoseconds to nanoseconds. Nitrogen-15 relaxation rates have been measured at five magnetic fields, ranging from 9.4 to 23.5 T (400–1000 MHz for protons). This exceptional wealth of data allowed us to map the spectral density function for the motions of backbone NH pairs in the partially disordered transcription factor Engrailed at 11 different frequencies. We introduce an approach called interpretation of motions by a projection onto an array of correlation times (IMPACT), which focuses on an array of six correlation times with intervals that are equidistant on a logarithmic scale between 21 ps and 21 ns. The distribution of motions in Engrailed varies smoothly along the protein sequence and is multimodal for most residues, with a prevalence of motions around 1 ns in the IDR. We show that IMPACT often provides better quantitative agreement with experimental data than conventional model-free or extended model-free analyses with two or three correlation times. We introduce a graphical representation that offers a convenient platform for a qualitative discussion of dynamics. Even when relaxation data are only acquired at three magnetic fields that are readily accessible, the IMPACT analysis gives a satisfactory characterization of spectral density functions, thus opening the way to a broad use of this approach.

## Introduction

Intrinsically disordered proteins (IDPs) and regions (IDRs) lack a stable three-dimensional structure organized around a hydrophobic core (1). Such proteins nevertheless play crucial roles in many cellular processes (2). The discovery of IDPs and IDRs is a challenge for the structure-function paradigm (3) and has opened the way to new biophysical contributions to modern proteomics (4). The characterization of the conformational space of IDPs and IDRs can provide insight into the ensemble representation of their three-dimensional organization (5–8). A detailed and quantitative description of the time dependence of the exploration of the conformational space of IDPs and IDRs is required to predict (9) and understand the molecular mechanisms underlying their biological function at the atomic scale.

NMR spectroscopy is a powerful tool for probing molecular motions at atomic resolution on a broad range of timescales in both ordered and disordered proteins (6,10,11). In particular, nuclear spin relaxation can be used to probe a diversity of motions from fast (picoseconds to nanoseconds) reorientation to slow (microseconds to milliseconds) chemical exchange (11,12). Pico- and nanosecond motions of protein backbones are most often characterized by analyzing nitrogen-15 relaxation rates, primarily the longitudinal, *R*_1_, and transverse, *R*_2_, relaxation rates, usually supplemented by ^15^N-{^1^H} nuclear Overhauser effects (NOEs). The most general level of analysis provides a map of the spectral density for reorientational motions of the internuclear ^15^N-^1^H vectors of the protein backbone (13). In folded proteins, a further step consists in the deconvolution of overall motion (rotational diffusion) and internal dynamics, which is possible when these two types of motions are statistically independent (14,15). The most popular framework for such an analysis is the model-free approach (14), for which the motions of each NH vector are described by a correlation time for overall motion and a correlation time and an order parameter for local motions. The so-called extended model-free approach was later introduced to account for internal motions characterized by two correlation times (16).

The structural flexibility of IDPs and IDRs on nanosecond timescales casts serious doubt on the separation of overall and internal motions. The very notion of a single overall motion can be challenged for IDPs. At best, an overall diffusion tensor would correspond to an average over a set of time-dependent diffusion tensors. In addition, local reorientations of bond vectors due to conformational changes that may occur on timescales similar to the instantaneous overall diffusion would make the statistical independence of internal and overall motions less plausible. New methods therefore have to be developed to describe and rationalize the dynamic properties of IDPs and IDRs (17–19).

Several approaches have been developed in the last 15 years to extract quantitative information about pico- and nanosecond dynamics in IDPs and IDRs from nuclear spin relaxation rates. Often based on spectral density mapping (20,21), most of these approaches rely on the model-free formalism, with residue-specific correlation times (22,23) (i.e., without an overall diffusion tensor), a distribution of picosecond and nanosecond correlation times (24,25), or a statistical analysis of extended model-free parameters (26). An analysis based on a distribution of correlation times necessarily introduces a physical bias, since one must choose a mathematical function to describe the distribution. In the case of the model-independent correlation (MIC) time distribution (26), the statistical independence of three types of motions is not required, since the extended model-free results are considered as a simplified representation of a continuous distribution of correlation times. However, the significance of such a statistical treatment is necessarily limited, since it provides little information about the number of modes of the actual distribution of correlation times. Neither approach seems suited to describe a distribution of correlation times that is a priori unknown. However, simply increasing the number of correlation times or distributions in either approach would be questionable, since the empirical information available from nuclear spin relaxation is limited.

Here, we introduce an approach we call interpretation of motions by a projection onto an array of correlation times (IMPACT) to analyze multiple-field relaxation data in disordered proteins. This method relies as little as possible on any particular physical model of protein motions but constitutes a mathematical reconstruction of the distribution of correlation times. We define an array of *n* correlation times, *τ*_*i*_ (or, equivalently, of reciprocal frequencies, *ω*_*i*_ = 1/*τ*_*i*_) in a range that is effectively sampled by nitrogen-15 relaxation. The experimental spectral density function is then reproduced by a sum of *n* Lorentzian functions, *J*_*i*_(*ω*), one for each correlation time *τ*_*i*_. The result of this process, similar to a projection onto a basis of Lorentzian functions, is a discrete distribution of correlation times spanning a range that is relevant to rationalize relaxation. This approach is analogous to the discretization step encountered in regularization methods (27,28), but the volume of experimental data exploited in this study is too limited to use a full regularization approach. Nevertheless, the multimodal character of the distribution of correlation times can be nicely revealed, and the most relevant correlation times for backbone motions can clearly be identified.

IMPACT was originally conceived for a set of relaxation rates obtained at five magnetic fields ranging from 9.4 to 23.5 T (i.e., with proton Larmor frequencies of 400, 500, 600, 800, and 1000 MHz) and later applied to a more limited set recorded at 500, 600, and 800 MHz. Relaxation rates were recorded for a uniformly nitrogen-15-labeled sample of the protein Engrailed 2. Engrailed 2 is a transcription factor that possesses a well-folded DNA-binding homeodomain and a long, 200-residue, mostly disordered N-terminal region. The disordered region plays a crucial role in the regulation of the activity of the protein and, in particular, in binding to transcriptional regulators (29,30). We decided to study an Engrailed 2 fragment (residues 146–259) encompassing the folded homeodomain (residues 200–259) and an N-terminal 54-residue disordered region (residues 146–199) (31). The results of IMPACT show that motions with correlation times close to 1 ns dominate reorientational dynamics in the most disordered regions of the protein, which is believed to be a general property of IDPs and IDRs (32). Yet, the broad variability of correlation times of backbone motions throughout the disordered region of Engrailed stands in stark contrast with the homogeneous dynamic properties of the folded homeodomain. This study reveals a surprising richness of backbone dynamics in IDPs and IDRs on pico- and nanosecond timescales, not found in folded proteins that have been widely studied over the past three decades.

## Materials and Methods

### Sample

All experiments were performed on a sample of uniformly nitrogen-15-labeled chicken Engrailed 2 (residues 146–259) at a concentration of 0.6 mM in 40 mM sodium succinate buffer at pH 6 supplemented with 1 *μ*g/mL of each of the three protease inhibitors leupeptin, pepstatin, and AEBSF, as well as 10 *μ*M EDTA, which allow one to increase the lifetime of the protein (33). The protein was prepared as described elsewhere (31). Note that the protein construct comprises the residues Gly-Pro-Met at the N-terminus before residue Glu^146^, which remain after cleavage of the GST-tag by PreScission protease (GE Healthcare, Little Chalfont, UK). All experiments were carried out at 303 K, which was adjusted in each spectrometer to have a chemical shift difference of 1.462 ppm between the signals of the methyl and hydroxyl protons of pure methanol (4% protonated and 96% deuterated).

### NMR spectroscopy

The relaxation rates were measured at five different static fields of 9.4, 11.7, 14.1, 18.8, and 23.5 T, with corresponding proton Larmor frequencies of 400, 500, 600, 800, and 1000 MHz. Three aliquots of the same sample were used for all experiments, except at 11.7 T, which was performed on a separate, but identical, sample.

At each field, a full set of ^15^N relaxation measurements was obtained. The longitudinal relaxation rates, *R*_1_(^15^N), were obtained in the traditional way (34–36), with saturation of the water signal for each scan, whereas the transverse relaxation rates, *R*_2_(^15^N), were recorded with a train of ^15^N *π*-pulses (Carr-Purcell-Meiboom-Gill pulse train), interleaved with ^1^H *π*-pulses to suppress cross-correlated relaxation effects. ^15^N-{^1^H} NOEs were obtained by detecting the ^15^N steady-state polarization while saturating the protons with a train of *π*-pulses, with suitable interpulse delays and *rf* amplitudes (37,38). Finally, experiments to measure the transverse and longitudinal cross-relaxation rates due to correlated fluctuations of the nitrogen-15 chemical shift anisotropy (CSA) and the dipolar coupling between the ^15^N nucleus and the amide proton were recorded using the so-called symmetrical reconversion principle (39,40). All experiments were recorded on Bruker Avance spectrometers (Billerica, MA). Experiments at 500 MHz, 800 MHz, and 1 GHz, and the NOE at 600 MHz, have been recorded using triple-resonance indirect-detection cryogenic probes (41) equipped with *z*-axis pulsed-field gradients. Other experiments at 600 MHz were recorded on an indirect-detection triple-resonance probe with triple-axis gradients with detection coils at room temperature. Experiments at 400 MHz were recorded on a liquid-nitrogen-cooled cryogenic probe (Prodigy BBO, Bruker) equipped with a *z*-axis gradient.

### Spectral density analysis

The full analysis was carried out at 11 points obtained with the reduced spectral mapping, *J*(0.87*ω*_H_) and *J*(*ω*_N_) at five fields and *J*(*ω* = 0) calculated from relaxation rates measured at 23.5 T. Analyses with two sets of three magnetic fields used seven points on the spectral density function; *J*(*ω* = 0) was derived from the relaxation rates measured at the highest magnetic field, i.e., 18.8 T or 23.5 T. A Monte Carlo simulation with 510 steps was performed to evaluate the error of each parameter, *A*_*i*_. All simulations were carried out with Mathematica (42).

### Supporting Material

The Supporting Material includes tables of all relaxation rates used in the analysis and tables of all parameters resulting from conventional analysis with two and three correlation times as well as from our IMPACT analysis; equations relevant for reduced spectral density mapping; a plot of transverse relaxation rates, *R*_2_, measured at 18.8 T; a comparison of Akaike’s Information Criteria (AIC) for IMPACT and conventional analyses with two or three correlation times; one-dimensional plots of AIC for five- and six-correlation-time analyses; a correlation of consecutive IMPACT coefficients; plots of IMPACT coefficients and the IMPACT barcode representation of the analysis of relaxation rates based on data recorded at a set of three fields, which cover a broad range (9.4, 14.1, and 23.5 T) and at a set of three more widely accessible fields (11.7, 14.1, and 18.8 T); and IMPACT coefficients for an analysis with five correlation times and *τ*_max_ = 38 ns.

## Results and Discussion

### Secondary structure

**Figure 1:**
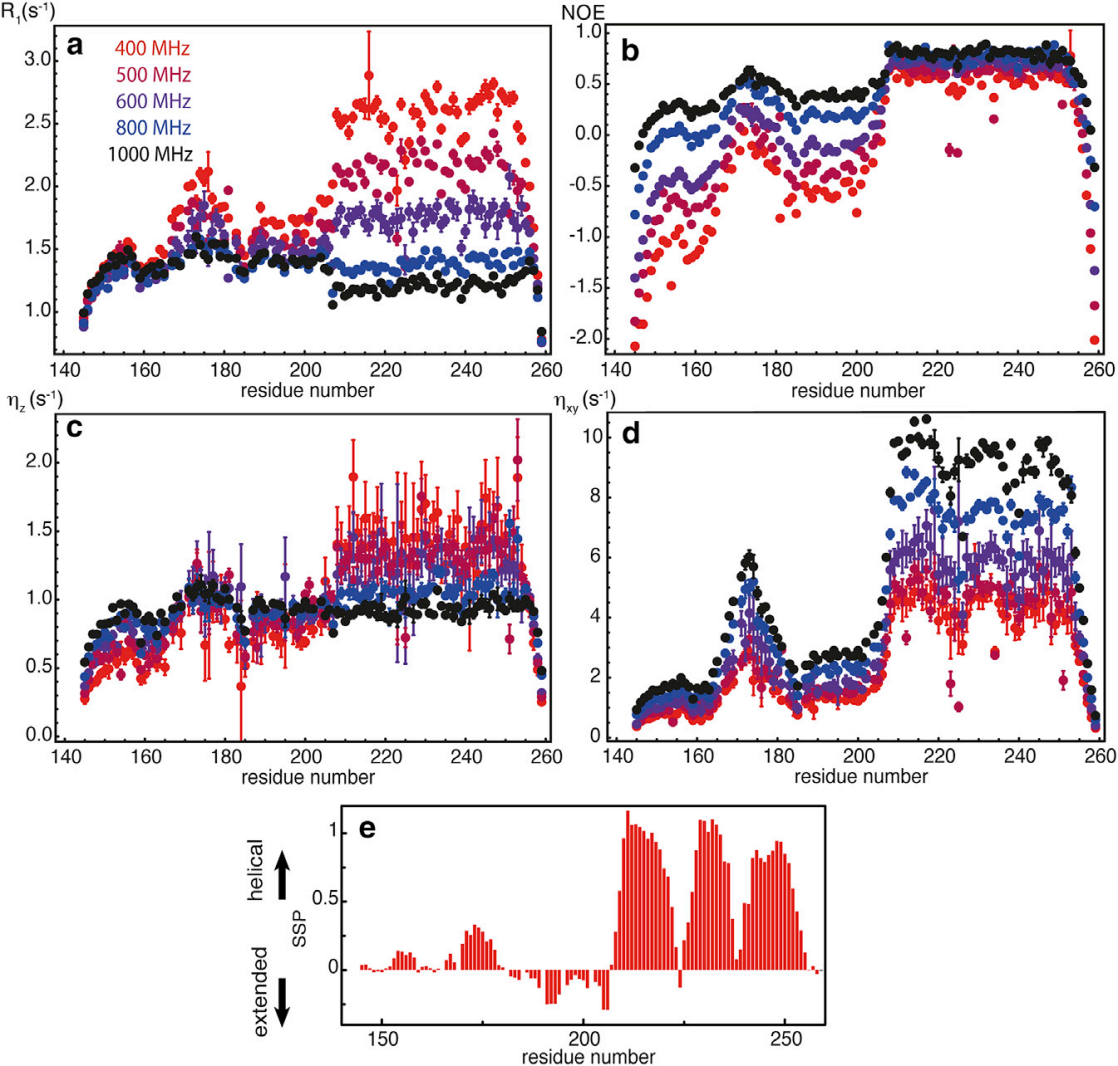
Backbone ^15^N relaxation rates and NOEs measured in Engrailed 2 at five magnetic fields: 400 MHz (*red*), 500 MHz (*burgundy*), 600 MHz (*purple*), 800 MHz (*blue*), and 1000 MHz (*black*). (*a*) Longitudinal relaxation rates, *R*_1_, of ^15^N. (*b*) ^15^N-{^1^H} NOE ratios. (*c*) Longitudinal cross-relaxation rates, *η*_*z*_, due to correlated fluctuations of the ^15^N CSA and the ^15^N-^1^H dipolar couplings. (*d*) Transverse cross-relaxation rates, *η*_*xy*_, due to the same correlated fluctuations. (*e*) SSP calculated from the chemical shifts of carbonyl and *α* and *β* carbon-13 nuclei. To see this figure in color, go online.

Fig. 1*e* displays the secondary structure propensity (SSP) (43) based on the assignment of the protein (31). The three *α-*helices of the homeodomain (residues 200–259) are well identified by SSP scores close to 1. Another region, including the so-called hexapeptide (residues 169–174, WPAWVY) and surrounding residues, displays SSP scores close to 0.3, thus highlighting the presence of some (residual) structure. The region connecting the hexapeptide and the homeodomain features negative SSP scores, which suggests a trend toward extended conformations.

Relaxation experiments were carried out at 400, 500, 600, 800, and 1000 MHz (Fig. 1, *a*–*d*) to determine longitudinal *R*_1_ nitrogen-15 relaxation rates, the steady-state ^15^N-{^1^H} NOE, as well as the longitudinal *η*_*z*_ and transverse *η*_*xy*_ cross-relaxation rates due to correlated fluctuations of the nitrogen-15 CSA and the dipolar coupling with the amide proton. Transverse relaxation rates, *R*_2_, were measured using Carr-Purcell-Meiboom-Gill echo trains at 800 MHz (Fig. S1 in the Supporting Material). All experiments were analyzed with NMRPipe and the intensities were obtained from a fit of peaks with the routine nlinLS (44). In some exceptional cases, the limited resolution of spectra measured at 9.4 and 11.7 T may have led to inaccuracies in the intensities of a few poorly resolved peaks.

The uniform decrease of the longitudinal relaxation rates, *R*_1_, with increasing magnetic field *B*_0_ in the 200–259 homeodomain (Fig. 1*a*) indicates motions in the nanosecond range, resulting from overall rotational diffusion. The variations *R*_1_(*B*_0_) are much less pronounced in the disordered region, except in the 169–174 hexapeptide region. Longitudinal cross-relaxation rates, *η*_*z*_ (Fig. 1*c*), increase with *B*_0_ in the IDR. This reflects the very slow decay, slower than 1/*ω*, of the spectral density function in the range 40–100 MHz (i.e., the range of ^15^N Larmor frequencies between 9.4 and 23.5 T), as the increase of the amplitude of the CSA interaction counterbalances the decay of the spectral density function with increasing frequency. The profile of transverse relaxation rates, *R*_2_, is marked by variations along the sequence of the protein of both the distribution of picosecond-nanosecond motions and contributions of chemical exchange (Fig. S1). NOEs are sensitive markers of local order in IDPs and IDRs and have been used as such for many years (45). Indeed, the variations of NOEs along the sequence are pronounced at moderate fields (9.4–14.1 T); however, the profile of NOEs is much flatter at high fields, in particular at 23.5 T. On the other hand, transverse cross-correlation rates, *η*_*xy*_, which depend primarily on *J*(*ω* = 0), exhibit sharp variations at all fields that are strongly correlated with SSP scores. This suggests that transverse cross-correlation rates, *η*_*xy*_, should become the method of choice to characterize order in IDPs and IDRs.

### Spectral density mapping

Most current software packages designed to characterize protein dynamics based on relaxation rates (46–50) offer a direct derivation of the parameters of local dynamics (order parameters and correlation times for local motions). This approach is efficient and reliable when the theoretical framework of the analysis has been validated. Since a general understanding of motions in intrinsically disordered proteins is still lacking, the derivation of the spectral densities from relaxation rates provides a representation of experimental data that is more amenable to physical analysis than relaxation rates (20).

**Figure 2:**
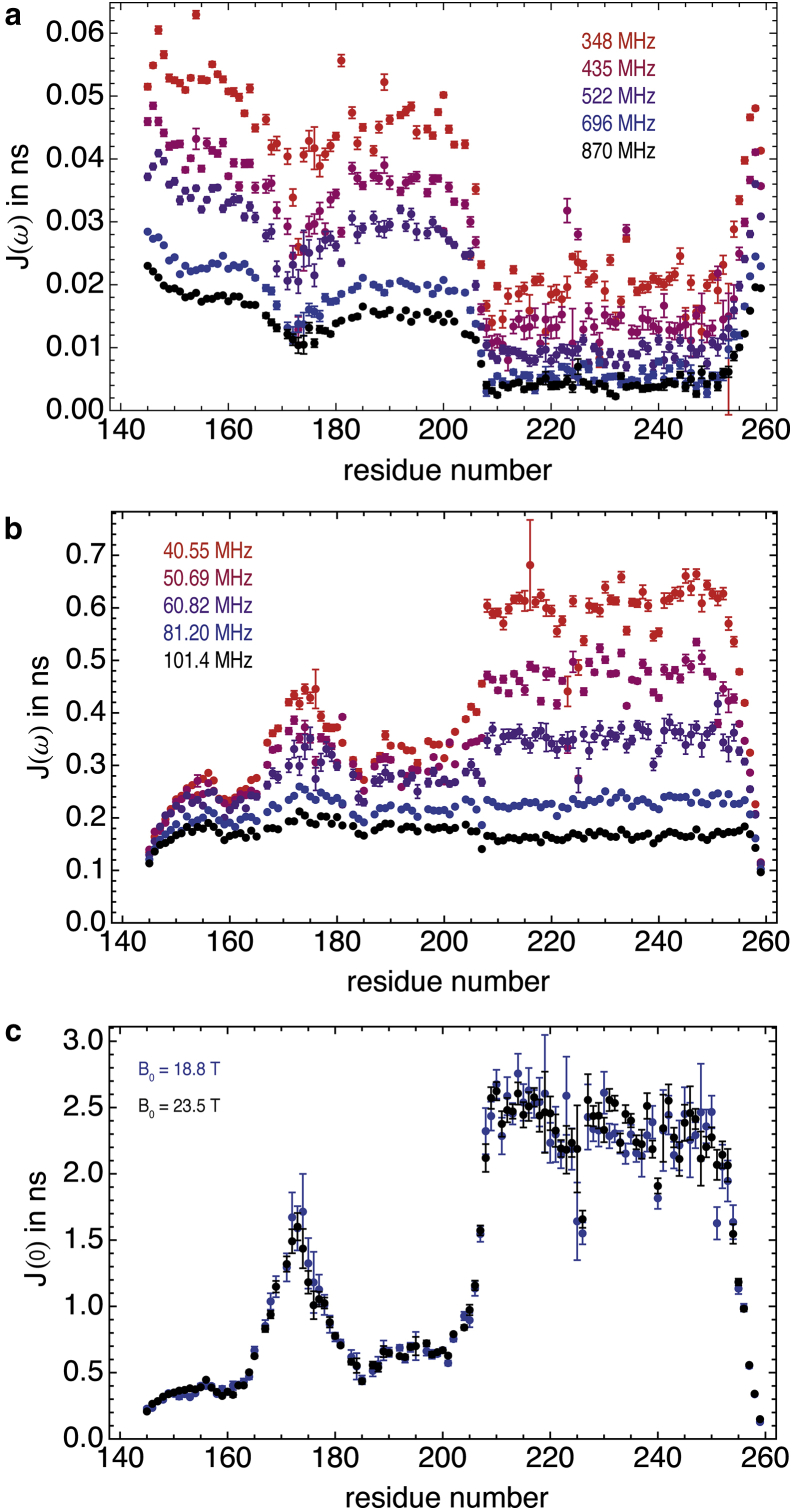
Spectral density functions for backbone NH vectors in Engrailed 2. (*a*) Effective spectral density near the proton Larmor frequency, *J*(0.87*ω*_H_) (ns). (*b*) Spectral density at the Larmor frequency of nitrogen-15, *J*(*ω*_N_) (ns). (*c*) Spectral density at zero frequency, *J*(0) (ns). All data are color-coded as a function of the magnetic field at which the relaxation rates were recorded, with the same code as in Fig. 1. To see this figure in color, go online.

Spectral density mapping (13) can be achieved without resorting to any proton auto-relaxation rate (51,52). The effective spectral density at high frequency, *J*(0.87*ω*_H_) (see Fig. 2*b*), can be derived from {^1^H}-^15^N NOE and longitudinal nitrogen-15 relaxation rates for different magnetic fields according to (51)

(1) $$J\left(0.87\omega_{\text{H}}\right)=\frac{4\gamma_{\text{N}}R_{1}\left(NOE-1\right)}{5d^{2}\gamma_{\text{H}}},$$

with $d=\left(\mu_{0}/4\pi \right)\left(\hbar \gamma_{\text{H}}\gamma_{\text{N}}/\langle r_{\text{NH}}^{3}\rangle \right)$. *μ*_0_ is the permittivity of free space; *γ*_H_ and *γ*_N_ are the gyromagnetic ratios of the proton and nitrogen-15 nuclei; ℏ is Planck’s constant divided by 2*π* and *r*_NH_ = 1.02 Å is the distance between the amide proton and the nitrogen-15 nucleus.

Our data recorded at five magnetic fields allowed us to fit the spectral density function at high frequency, *J*(0.87*ω*_H_), to the expression

(2) $$J\left(\omega \right)=\lambda +\frac{\mu }{\omega^{2}},$$

in analogy to an earlier study of carbon-13 relaxation (53). The parameters *λ* and *μ* are real positive numbers. This functional form is expected to be a good approximation of the spectral density at high frequency in a folded protein, but not necessarily for a protein with significant motions with correlation times in the hundreds of picoseconds. Nevertheless, we obtain satisfactory fits for all residues in the IDR as well as in the homeodomain. This validates the self-consistency of the use of a single effective frequency, *ω*_eff_ = 0.87*ω*_H_, in Eqs. 8–10 of the article by Farrow et al. (51) (see Eqs. S1–S6), where the spectral density function at high frequency was assumed to be of the form of Eq. 2 in both the folded homeodomain and the IDR. Thus, most results of spectral density mapping pertaining to disordered proteins that have been published in recent years are validated.

The results of the fit of the spectral density were used to evaluate contributions to the spectral density at higher frequencies (at *ω*_H_ ± *ω*_N_) in the derivation of *J*(*ω*_N_) from the rate *R*_1_ according to the equation

(3) $$J\left(\omega_{\text{N}}\right)=R_{1}/\left(3d^{2}/4+c^{2}\right)-\left(6J\left(\omega_{H}+\omega_{\text{N}}\right)+J\left(\omega_{\text{H}}-\omega_{\text{N}}\right)\right)/\left(3+4c^{2}\text{/}d^{2}\right),$$

with $c=\gamma_{\text{N}}B_{0}\Delta \sigma /\sqrt{3}$ and Δ*σ* = 160 ppm is the axially symmetric CSA of the nitrogen-15 nucleus.

Overall, contributions of high-frequency terms to *R*_1_ are small (54), so that the deviations between the values of *J*(*ω*_N_) obtained from a series of approximations (51) and the current method are limited to ∼2%, which is commensurate with the estimated precision (Fig. S2). Again, this validates, a posteriori, many spectral density mapping studies performed on IDPs. In addition, the low sensitivity of *J*(*ω*_N_) upon the model used to describe the spectral density at high frequency shows that the enhanced accuracy expected from more sophisticated approaches, for instance, following Kadeřávek et al. (20), would be smaller than the typical precision of our measurements. The values of *J*(*ω*_N_) derived at five magnetic fields are shown in Fig. 2*b*.

To avoid contributions from line-broadening due to chemical exchange, we did not consider transverse relaxation rates, *R*_2_(^15^N), and only used longitudinal and transverse CSA/DD cross-correlated relaxation rates, *η*_*z*_ and *η*_*xy*_, to derive the spectral density *J*(0) from *J*(*ω*_N_) using (55)

(4) $$J\left(0\right)=J\left(\omega_{\text{N}}\right)\frac{3}{4}\left(2\frac{\eta_{xy}}{\eta_{z}}-1\right).$$

As can be seen in Fig. S3, measurements of *η*_*z*_ and *η*_*xy*_ are not precise enough at lower fields to provide reliable estimates of *J*(0) by lack of sensitivity (in particular for *η*_*z*_). However, the data recorded at 18.8 T and 23.5 T (Fig. 2*c*) are very similar and do not show any of the outliers observed at lower fields. Significant chemical exchange contributions to *R*_2_(^15^N) can be observed in the hexapeptide region of the disordered region and in the homeodomain (see Fig. S1). Such contributions preclude the proper derivation of *J*(0) from *R*_2_(^15^N) rates, in particular at high magnetic fields.

### Principles and optimization of IMPACT

**Figure 3:**
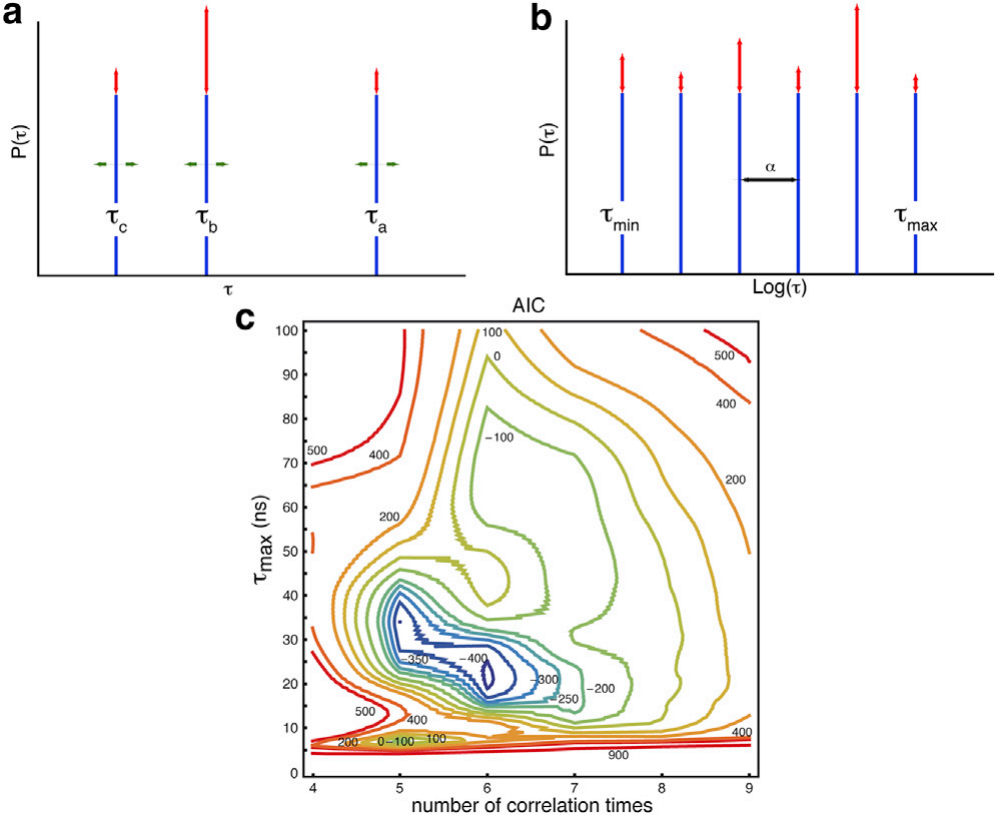
Principle and optimization of the parameters of our IMPACT analysis. (*a*) In the 3CT analysis, both the value and the relative weight of each correlation time must be adjusted. (*b*) In IMPACT, the values of the correlation times are fixed and equally spaced on a logarithmic scale, so that only their relative weights need adjusting. (*c*) Optimization of IMPACT by considering AIC. The range (*τ*_m*i*n_, *τ*_max_) of correlation times characterized by IMPACT was varied from (1 ps, 1 ns) to (100 ps, 100 ns) and the number of correlation times was varied in the range *n* = 4–9. Despite the solid lines shown in the contour plot (*c*), the reader should be aware that the number of correlation times is an integer. To see this figure in color, go online.

The limitations of conventional approaches to the analysis of relaxation rates in IDPs and IDRs result from the complexity of their dynamics. These span at least three orders of magnitude, so that it appears unlikely that they can be accurately described by a single distribution of correlation times or by a small number of correlation times. However, the scarcity of relaxation rates limits the number of adjustable parameters that can be determined and thus the sophistication of spectral density functions that can be postulated. Here, we significantly increase the number of correlation times by defining an array of *n* fixed correlation times. Only the relative coefficient of each correlation time in the distribution is fitted to experimental data, so that the number of adjustable parameters is reduced. Thus, our only assumption is that the correlation function can be approximated by a sum of exponentials. The physical content of the IMPACT model is thus limited to a minimum. IMPACT can be described as a mathematical approach that converts experimental relaxation rates (or, equivalently, spectral density mapping results) into a distribution of correlation times that is more amenable to physical interpretation than the raw experimental data. The array of *n* correlation times is defined as a geometric series, so that correlation times are equally spaced on a logarithmic scale (Fig. 3, *a* and *b*):

(5) $$\tau_{i}=\alpha^{i-1}\tau_{\text{max}}\;\alpha ={\left(\tau_{\text{min}}/\tau_{\text{max}}\right)}^{\frac{1}{n-1}}.$$

Thus, *J*(*ω*) is a sum of Lorentzian functions:

(6) $$J\left(\omega \right)=\sum_{i=1}^{n}J_{i}\left(\omega \right)=\frac{2}{5}\sum_{i=1}^{n}\frac{A_{i}\tau_{i}}{1+{\left(\omega \tau_{i}\right)}^{2}},$$

where *A*_*i*_ is the coefficient of correlation time *τ*_*i*_ in the spectral density function. The coefficients *A*_*i*_ must be positive and fulfill the normalization constraint

(7) $$\sum_{i=1}^{n}A_{i}=1.$$

Thus, the number of free parameters is *n*−1.

A preliminary step of the IMPACT analysis is the optimization of the three parameters *τ*_m*i*n_, *τ*_max_, and *n*. The first step is to define the range of correlation times that are probed by relaxation rates. A series of correlation times could be chosen as the inverse of the Larmor frequencies at which the spectral density is mapped, in analogy to the study by LeMaster (56). Considering that the range of frequencies where a Lorentzian function varies extends beyond the inflection point, we chose a slightly different approach. We first define the range of correlation times that are sampled by various ^15^N relaxation rates. We consider that the lowest magnetic field adapted to protein studies is 400 MHz, whereas the highest accessible field currently is 1 GHz. Thus, the lowest nonzero frequency at which the spectral density is sampled is *ω*_N_/2*π* = 40 MHz, and the highest is 0.87*ω*_H_/2*π* = 870 MHz. A Lorentzian function with a correlation time of *τ*_c_ = 40 ns drops to 1% of *J*(0) at *ω*_N_/2*π* = 40 MHz. A Lorentzian with *τ*_c_ = 18 ps merely decreases to 99% of *J*(0) at 0.87*ω*_H_/2*π* = 870 MHz. The resulting range spans slightly more than three orders of magnitude. Therefore, we have decided to limit the range to three orders of magnitude

(8) $$\tau_{\text{max}}/\tau_{\text{min}}=10^{3}.$$

To define the optimal values of *τ*_m*i*n_ and *τ*_max_, we carried out a series of IMPACT analyses for [*τ*_m*i*n_, *τ*_max_] = [1 ps, 1 ns] to [100 ps, 100 ns], as well as for 4 < *n* < 9. In contrast to the approach of LeMaster (56), the number of correlation times is adjustable. The statistical relevance of each combination of parameters was evaluated from the resulting Akaike’s information criteria (AIC) (57–60):

(9) $$\text{A}\text{I}\text{C}=n_{\text{exp}}\text{ln}\left(\sum_{k=1}^{nres}\chi_{k}^{2}/n_{\text{exp}}\right)+2n_{\text{model}}+C.$$

*n*_exp_ = *n*_*J*_ × *nres* is the total number of experimental data, with *n*_*J*_ = 11 points at which the spectral density function *J*(*ω*) is sampled when relaxation data at five magnetic fields are used; *nres* = 108 is the number of residues included in the analysis; and *n*_model_ = (*n* − 1) × *nres* is the number of free parameters in each model. Here, the constant is *C* = 0. AIC are shown in Fig. 3*c*. Two local minima were found for [*τ*_m*i*n_, *τ*_max_] = [34 ps, 34 ns] with *n* = 5 and for [*τ*_m*i*n_, *τ*_max_] = [21 ps, 21 ns] with *n* = 6. The likelihood of the latter array of correlation times is 10^3^ times higher than that of the former. Here, we will thus present the IMPACT analysis with [*τ*_m*i*n_, *τ*_max_] = [21 ps, 21 ns] and *n* = 6; the analysis with [*τ*_m*i*n_, *τ*_max_] = [34 ps, 34 ns] and *n* = 5 can be found in the Supporting Material. This result is dominated by the diverse dynamic properties of the IDR of Engrailed. Indeed, if we exclude the rigid residues of the homeodomain, the optimal parameters change slightly (residues 146–207) to [*τ*_m*i*n_, *τ*_max_] = [42 ps, 42 ns] with *n* = 5, whereas the optimal set of parameters for the rigid part of the homeodomain alone (residues 208–259) is significantly different, [*τ*_m*i*n_, *τ*_max_] = [10 ps, 10 ns] and *n* = 4.

### Application of IMPACT to Engrailed

**Figure 4:**
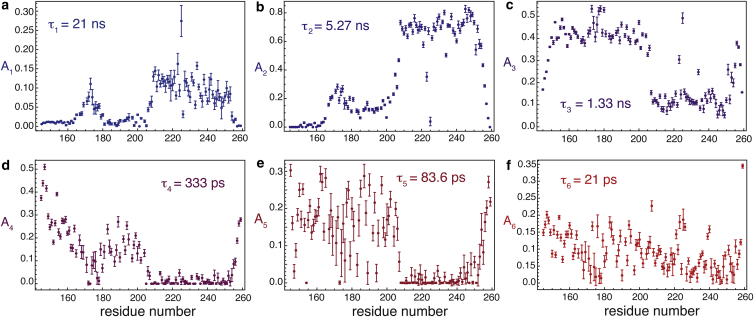
Plots of the six coefficients, *A*_*i*_ (*i* = 1, 2… 6) of the *n* = 6 correlation times, *τ*_*i*_, in the range [*τ*_m*i*n_, *τ*_max_] = [21 ps, 21 ns] determined by the IMPACT analysis of Engrailed: (*a*) *τ*_1_ = 21 ns; (*b*) *τ*_2_ = 5.27 ns; (*c*) *τ*_3_ = 1.33 ns; (*d*) *τ*_4_ = 333 ps; (*e*) *τ*_5_ = 83.6 ps; (*f*) *τ*_6_ = 21 ps. To see this figure in color, go online.

The optimal parameters *n* = 6 and [*τ*_m*i*n_, *τ*_max_] = [21 ps, 21 ns] were employed to analyze the spectral density function in Engrailed. Note that the coefficients *A*_*i*_ were fitted to spectral density mapping results. In principle, relaxation rates could also be used directly as input for the IMPACT analysis. Fig. 4 illustrates the remarkable variety of dynamics found in Engrailed. In the homeodomain, the second correlation time, *τ*_2_, lies just below the correlation time for overall rotational diffusion, which is close to 7 ns, as can be seen from the analysis based on only two correlation times (vide infra). Thus, the second coefficient is by far the most important in the homeodomain. A small amplitude *A*_1_ of *τ*_1_ corrects for the fact that *τ*_2_ is shorter than the actual correlation time for the motion of the whole domain. Note that the correlation time for overall diffusion, *τ*_m_, is well approximated by:

(10) $$\tau_{\text{m}}\approx \left(A_{1}\tau_{1}+A_{2}\tau_{2}\right)/\left(A_{1}+A_{2}\right).$$

The average value over the helices of the homeodomain is <*τ*_m_> = 7.19 ns, which is in good agreement with an estimate of the correlation time for overall diffusion (see below). Small but significant and mostly uniform contributions *A*_3_ of the correlation time *τ*_3_ in the three *α-*helices, which are also obtained in a conventional model-free analysis (vide infra), may be attributed to fluctuations of the overall diffusion tensor (61), likely due to conformational fluctuations of the IDR (residues 146–199). Enhanced values of *A*_3_ in the loops may reflect the flexibility of these regions (41). The very small coefficients *A*_4_ and *A*_5_ demonstrate the presence of a gap in the distribution of correlation times, as was also observed in ubiquitin (62). Finally, the coefficients *A*_6_ for the shortest correlation time *τ*_6_ indicate the presence of fast motions in the tens of picoseconds range. Note that the Lorentzian function *J*_6_(*ω*) drops by ∼1% of *J*_6_(0) at the highest frequency explored in this analysis (i.e., *ω*/2*π* = 870 MHz). Thus, this last term in the spectral density function can be approximated to a constant that effectively represents all fast motions:

(11) $$J_{6}\left(\omega \right)\approx \frac{2}{5}A_{6}\tau_{6}\approx \frac{2}{5}\int_{0}^{\tau_{5}}p\left(\tau \right)d\tau ,$$

where *p*(*τ*) is the probability function of correlation times, containing little information on the complexity of such motions (63).

**Figure 5:**
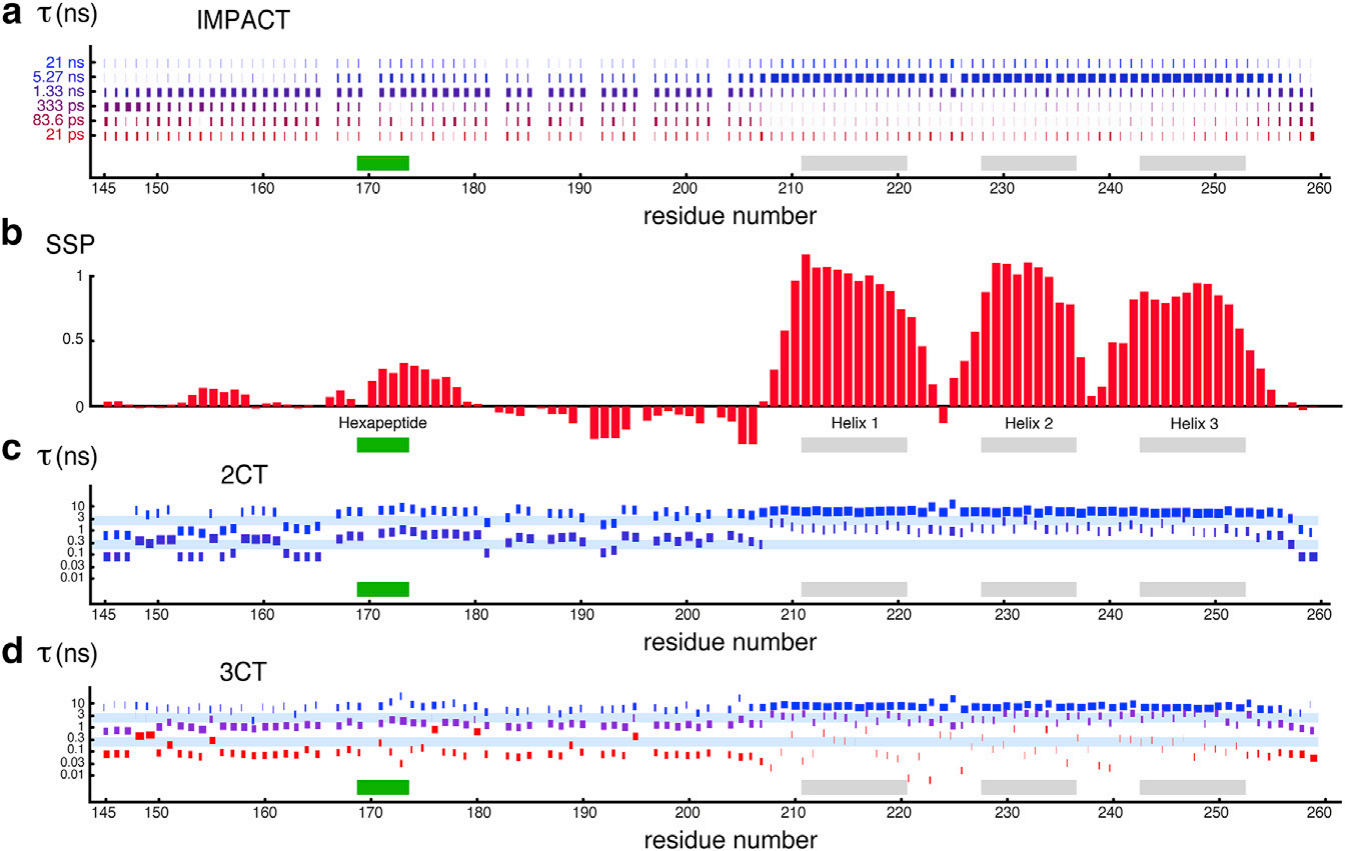
Graphical representations of (*a*) IMPACT, (*c*) 2CT, and (*d*) 3CT analyses of the spectral density function in Engrailed 2. Histograms are drawn for all residues and represent the contributions of (*a*) each of the six correlation times, *τ*_*i*_ (*i* = 1, 2, …6), considered in IMPACT, (*c*) each of the two correlation times, *τ*_*a*,*b*_, determined by the 2CT analysis, (*d*) each of the three correlation times, *τ*_a,b,c_, determined by the 3CT analysis. The width of each rectangle is proportional to the corresponding weights *A*_*i*_ in IMPACT (*a*), *B*_a,b_ in 2CT (*c*); and *B*_a,b,c_ in 3CT (*d*). In (*c*) and (*d*), the light blue horizontal bars represent the ranges of correlation times, *τ*, for which reciprocal frequencies lie in the constrained regions between 40 < 1/(2*π τ*) < 100 MHz or between 348 < 1/(2*πτ*) < 870 MHz. Gray rectangles in (*a*), (*c*), and (*d*) indicate rigid *α-*helices, and a green rectangle shows the rigid hydrophobic hexapeptide sequence. (*b*) As in Fig. 1*e*, the SSP is shown to guide the comparison between structural and dynamic features. To see this figure in color, go online.

Results obtained in the disordered region of Engrailed will be discussed with the help of Fig. 4 but also with the IMPACT barcode shown in Fig. 5. In the latter, for each residue, the width of each histogram represents the coefficient *A*_*i*_ associated with the correlation time *τ*_*i*_ that can be read on the *y* axis. This graph appears to be a convenient way to display the results of the IMPACT analysis in a single figure.

For the first residues at the N-terminus and the last residues at the C-terminus, significant coefficients *A*_3–6_ are found for the four shortest correlation times. This seems to indicate the presence of motions that are broadly distributed over all timescales up to 1 ns. On the other hand, the two disordered regions just at the N-terminus and the C-terminus of the hexapeptide display a high density of motions around *τ*_3_. The coefficients for the correlation time *τ*_4_ decrease almost linearly with the distance to the N- or C-termini of the polypeptide chain in disordered regions and reach different plateaus in each disordered segment. A notable difference between the disordered region at the N-terminus and the one between the hexapeptide and the homeodomain is the slight decrease of the coefficient *A*_3_ and a significant increase of *A*_2_. It is difficult to assign this change to a particular process without a better characterization of the conformational space of the protein (such characterization is beyond the scope of this article and is a work in progress). Nevertheless, two effects may contribute to the presence of some orientational order beyond 1 ns. First, this IDR is short and located between a folded domain and a small hydrophobic cluster, so that its dynamics is likely influenced by the overall diffusion of both structured elements. Second, this IDR contains a majority of residues that favor extended conformations, as confirmed by SSP scores: three proline residues, nine positively charged, and only one negatively charged residue between positions 177 and 198, which should restrict the conformational space and possibly slow down reorientational dynamics.

The barcode representation of IMPACT coefficients nicely illustrates variations of the ensemble of correlation times between successive structural elements. Thus, for instance, the decrease of motions in the subnanosecond range in the hexapeptide is accompanied by an increase in the suprananosecond range. Similarly, variations of the coefficients for correlation times at the N- and C-termini of the homeodomain (residues 200–210 and 254–260) illustrate the smooth transition of motional properties along the sequence of the polypeptide. Finally, even in the homeodomain, where a classical analysis of relaxation data should be most appropriate, the dynamic transitions between helices and the two loops are clearly visible and quantitatively characterized by the IMPACT approach. Loop *α*1-*α*2 features enhanced dynamics in both the 1 ns and tens of picoseconds ranges, as expected from the motions demonstrated by paramagnetic relaxation enhancement studies (41), whereas loop *α*2-*α*3 shows a significant but more moderate enhancement of motions. Overall, the IMPACT representation offers an elegant view of the correlation of structural and dynamic features, as can be seen from the SSP scores.

### Comparison with conventional analyses based on two or three correlation times

For the sake of comparison, we also fit two simple models where the spectral density function is assumed to consist of a sum of two and three Lorentzians in the manner of the familiar model-free and extended model-free approaches. However, as discussed in the Introduction, the core hypotheses of the model-free formalism cannot be fulfilled a priori, since the longest correlation time is probably an effective correlation time rather than the correlation time of overall rotational diffusion. The spectral density, *J*_2CT_, assuming two correlation times (2CT) can be written as

(12) $$J_{2\text{CT}}\left(\omega \right)=\frac{2}{5}\left[S^{2}\tau_{\text{a}}\text{/}\left(1+{\left(\omega \tau_{\text{a}}\right)}^{2}\right)+\left(1-S^{2}\right)\tau_{\text{b}}^{\prime }/\left(1+{\left(\omega \tau_{\text{b}}^{\prime }\right)}^{2}\right)\right]\;,$$

where $\tau_{b}^{\prime -1}=\tau_{a}^{-1}+\tau_{b}^{-1}$, *τ*_*a*_ is the long correlation time, *τ*_b_ is the short effective correlation time, and *S*^2^ is similar to the model-free order parameter. The spectral density function *J*_3CT_, assuming three correlation times (3CT), can be defined as

(13) $$J_{3\text{CT}}\left(\omega \right)=\frac{2}{5}\left[S^{2}\tau_{\text{a}}\text{/}\left(1+{\left(\omega \tau_{\text{a}}\right)}^{2}\right)+\left(S_{\text{f}}^{2}-S^{2}\right)\tau_{\text{b}}^{\prime }\text{/}\left(1+{\left(\omega \tau_{\text{b}}^{\prime }\right)}^{2}\right)+\left(1-S_{\text{f}}^{2}\right)\tau_{\text{c}}^{\prime }\text{/}\left(1+{\left(\omega \tau_{\text{c}}^{\prime }\right)}^{2}\right)\right],$$

where $\tau_{\text{c}}^{\prime -1}=\tau_{\text{a}}^{-1}+\tau_{\text{c}}^{-1}$, $\tau_{\text{a}}>\tau_{\text{b}}>\tau_{\text{c}}$, and $S_{\text{f}}^{2}$ is equivalent to the extended model-free order parameter for fast processes with a correlation time $\tau_{c}$. The two functions were fitted to the experimental spectral density, and a simple model selection was based on the comparison of the second order variant of AIC (see the Supporting Material), with *n*_*J*_ = 11 and *n*_model_ = 3 for the 2CT analysis and 5 for the 3CT analysis.

**Figure 6:**
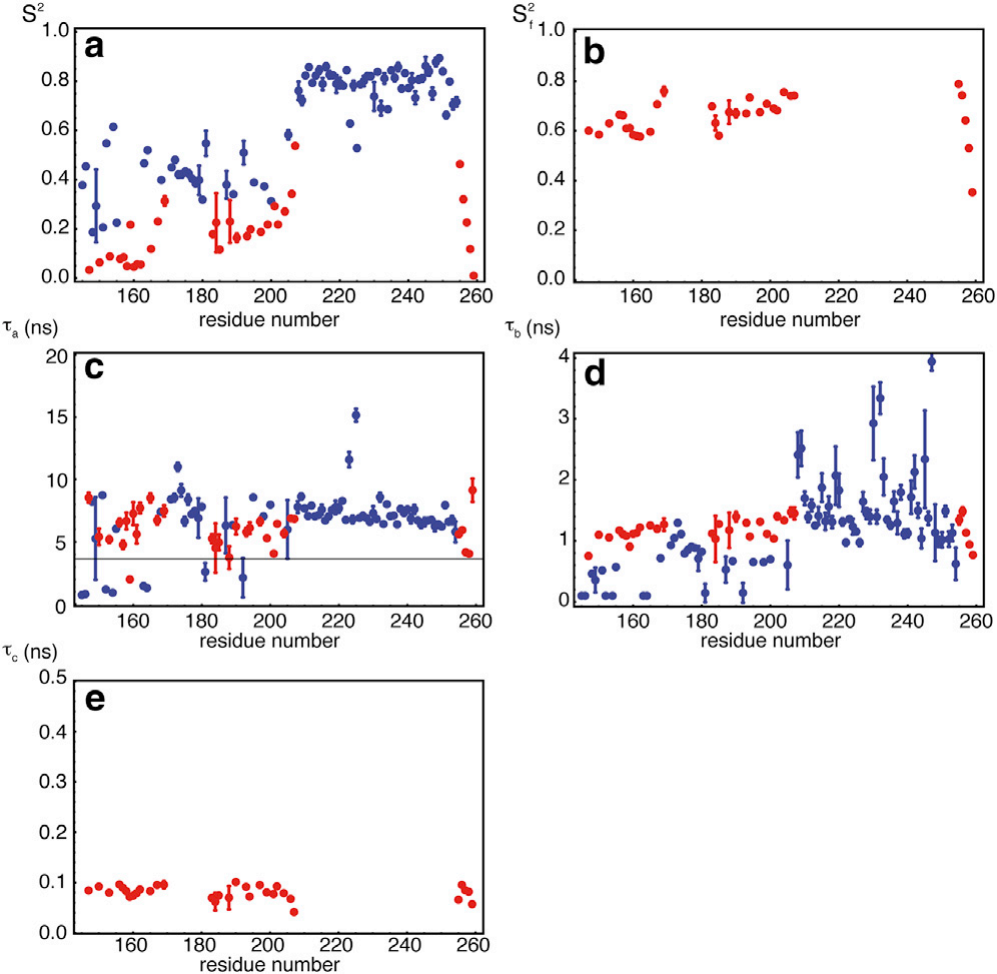
Results obtained for a conventional analysis with 2CT (*blue*) or 3CT (*red*). (*a*) Order parameter *S*^2^. (*b*) Order parameter *S*^2^_f_ for the fastest motion in the 3CT analysis. (*c*) Longest correlation time, *τ*_*a*_. (*d* and *e*) Intermediate correlation time, *τ*_*b*_ (*d*), and shortest correlation time, *τ*_c_ (*e*). Either the 2CT or the 3CT model was selected based on the lowest AIC. To see this figure in color, go online.

Results of this analysis are shown in Fig. 6. From a statistical point of view, the 2CT analysis is found to be sufficient to describe the motions in the mostly rigid homeodomain (residues 200–259), except at the flexible N- and C termini. With few exceptions, the longest correlation time yields a reliable measure of overall rotational diffusion, and the average value over the helices of the homeodomain is <*τ*_a_>_hom_ = 7.08 ns, in good agreement with the IMPACT analysis, with <*τ*_m_> = 7.19 ns. Interestingly, the second correlation time, *τ*_b_, found for most residues in the rigid homeodomain lies in the range 0.9 < *τ*_b_ < 1.8 ns. This is in agreement with the IMPACT analysis and is possibly due to fluctuations of the overall diffusion tensor resulting from conformational transitions in the disordered N-terminal region on timescales between 1 and 100 ns (61,64). In the disordered region (residues 146–199), the 2CT and 3CT analyses seem equally probable, with no particular pattern along the sequence, except in the hydrophobic hexapeptide cluster (residues 169–174), where the 2CT analysis is more satisfactory. The random patterns of 2CT versus 3CT selection seems to point to some instability of the model-selection step in the fit procedure. A 2CT or 3CT analysis can be performed with no model selection (see Fig. 5). The built-in absence of site-specific model selection in IMPACT shields this analysis from such a drawback. Low order parameters *S*^2^ are found throughout the IDR, with a significant increase in order in the hydrophobic cluster. The long correlation times in the disordered regions have a broad distribution (standard deviation of 2.5 ns), but the average value, <*τ*_a_>_IDR_ = 5.9 ns, is similar to what was found in unfolded proteins (32) and IDPs (65) and very close to the correlation time for overall tumbling of the homeodomain. The intermediate correlation time, which corresponds to the dominant term in the spectral density function, lies in the range 0.1 < *τ*_b_ < 1.4 ns, in agreement with the IMPACT analysis. The shortest correlation time is rather uniform and lies in the range 40 < *τ*_c_ < 120 ps.

One should be careful with the physical interpretation of these observations in the IDR of Engrailed. The three correlation times obtained are clearly separated in the time domain, which indicates a broad range of dynamic processes. The results should not be considered a priori as actual correlation times of particular motions, but rather as the best rendition of experimental results using two or three correlation times. This is illustrated by the jumps of order parameters and correlation times observed between the 2CT and 3CT models in the IDR, which illustrates the effective character of the fitted correlation times in this region, at least in the 2CT analysis. For instance, it is difficult to assign the longest correlation time to any particular dynamical process in the absence of complementary experimental or computational information. Such a process could be a single well-defined type of motion, such as the rotational diffusion of an IDR segment. Alternatively, the longest correlation time might account for the tail of a continuous distribution of correlation times and reflect slower motions in parts of the conformational space of the IDR. Interestingly, the correlation times obtained in a 3CT analysis often correspond to reciprocal frequencies (*ω* = 1/*τ*) that lie outside regions where the spectral density function can be adequately sampled (i.e., below 40 MHz, between 100 MHz and 348 MHz, and above 870 MHz). This is particularly true in the flexible region between the rigid hexapeptide and the rigid homeodomain and at the C-terminus of the protein. The regions where the spectral density function is most sensitive to the choice of correlation times correspond to ranges where we lack experimental constraints. This would be expected in the presence of a broad distribution of correlation times that would lead to a smooth decay of the spectral density function.

A direct comparison between the results of the 3CT analysis and our IMPACT approach is illustrated in Fig. 5. For the sake of comparison, we define in Fig. 5*a* the coefficients *B*_a,b,c_ associated with the correlation times *τ*_a,b,c_, as

(14) $$B_{\text{a}}=S^{2},B_{\text{b}}=S_{\text{f}}^{2}-S^{2},B_{\text{c}}=1-S_{\text{f}}^{2}.$$

The statistical significance of the fit resulting from our IMPACT analysis is often better than with either the 2CT or the 3CT analysis in the disordered regions, although it is somehow comparable to that of the 2CT model in the rigid homeodomain (Fig. S4). In particular, the almost complete absence of abnormally elevated *χ*^2^ values in the IDR of Engrailed shows that a faithful set of fitted *A*_*i*_ parameters can be obtained with diverse dynamical features. Interestingly, as can be seen from the schematic representation of correlation times that correspond to reciprocal frequencies that can be determined by spectral density mapping, the inflection points of most of the Lorentzian functions often lie in frequency regions where spectral density mapping does not yield any results. This is particularly true in the IDR between the hexapeptide and the homeodomain and at the C-terminus of the protein. This seems to indicate that the decay of the spectral density function is smoother than can be described by a sum of three Lorentzian functions. The fit pushes the inflection points of individual contributions to the spectral density function beyond the areas that benefit from rich experimental constraints. In addition, the absence of a residue-specific model selection in IMPACT provides results that are directly comparable, residue by residue, which allows for a better qualitative description of dynamic properties along the protein sequence. Admittedly, model selection can be omitted in the 2CT or 3CT analysis, as in Fig. 5, *a* and *b*.

A potential concern of the IMPACT analysis lies in the fact that the correlation functions *J*_*i*_(*ω*) are not independent, since they suffer from significant overlap. Hence, it is possible that different ensembles of coefficients *A*_*i*_ can describe the same experimental data. To test the sensitivity of our analysis to this potential flaw, we have plotted correlations of consecutive coefficients *A*_*i*_ (i.e., *A*_*i*_ as a function of *A*_*i*+1_) for all 510 steps of the Monte Carlo procedure employed in the fit. Typical results are shown in Fig. S6. There is a small anticorrelation between consecutive coefficients in several instances. This will give rise to a broader distribution of individual coefficients and thus lead to a decrease of the precision of these coefficients. In the worst case, a potential decrease of accuracy due to the interdependence of consecutive coefficients will be accompanied with a decrease in precision.

A potential concern of the IMPACT analysis is the risk of overinterpretation of the results. Here, we should be clear and provide a set of rules that should be followed by users of this approach.

1) The correlation times, *τ*_*i*_, are not physical correlation times of the system a priori. The range of correlation times is defined by the experimental observables, but the individual values *τ*_*i*_ are derived from a statistical analysis, not a physical analysis.
2) A nonzero coefficient *A*_1_, with *τ*_1_ the longest correlation time, means not that some motions with a correlation time *τ*_1_ were detected but that the distribution of correlation times is larger than zero for some correlation times larger than *τ*_2_.
3) Similarly, as mentioned in Eq. 11, the coefficient *A*_*n*_ of the shortest correlation time *τ*_*n*_ is an effective representation of all fastest motions.
4) If the coefficient *A*_*i*_ is larger than zero, the distribution of correlation times is larger than zero somewhere between *τ*_*i*+1_ and *τ*_*i*-1_.
5) If the coefficients *A*_*i*_ and *A*_*i*+1_ are both zero, the distribution of correlation times is expected to be zero at least between *τ*_*i*+1_ and *τ*_*i*_.

Finally, very few relaxation studies have compared rates at five or more magnetic fields (66,67). We have tested whether an IMPACT analysis of relaxation rates recorded at only three fields could give meaningful results, using either a broad range of magnetic fields (9.4, 14.1, and 23.5 T) or a narrow range of readily accessible magnetic fields (11.7, 14.1, and 18.8 T). In either case, this requires about two weeks of experimental time. The results of the analysis of relaxation rates at 9.4, 14.1, and 23.5 T shown in Figs. S7 and S8 are remarkably similar to those presented in Figs. 4 and 5. When the range of magnetic fields is restricted, with relaxation rates measured at 11.7, 14.1 and 18.8 T (Figs S9 and S10), some significant changes of IMPACT coefficients can be observed, but the overall description of the distribution of correlation times is very similar to what is obtained with relaxation rates at five magnetic fields. Hence, IMPACT can be applied to many proteins at a moderate cost in experimental time, and does not necessarily require exceptionally large data sets or magnetic fields as high as 23.5 T.

## Conclusions

We have presented a set of nitrogen-15 relaxation rates in the 114-residue, partially disordered protein Engrailed 2 recorded at five different magnetic fields. The transverse cross-correlated rate, *η*_*xy*_, was shown to be the most sensitive to the extent of order and disorder at all magnetic fields. The analysis validates the reduced spectral density mapping approach originally developed for folded proteins and already extensively applied to IDPs and IDRs. The spectral density functions can be fitted reasonably well with two or three correlation times, although such results may be difficult to interpret. We have introduced an approach to the analysis of spectral density functions, which we call IMPACT. This provides a better quantitative description of spectral density functions in IDRs as found in Engrailed than an analysis with three correlation times with the same number of adjustable parameters. We also introduce a barcode representation of IMPACT, which provides a condensed graphical representation of large amounts of data in a single figure. This representation lends itself to a qualitative discussion of order and disorder in proteins. IMPACT can also be useful for analyzing a smaller set of relaxation rates recorded at only three magnetic fields. IMPACT provides a unique framework for the description of the timescales of motions in IDPs and IDRs. Our approach is complementary to the determination of conformational ensembles (7,68). Insight into the dynamics of IDPs and IDRs should greatly benefit from a combined analysis.

## Author Contributions

F.F., O.L., and P.P. designed the research; S.N.K., C.C., R.A., N.S., O.L., P.P., and F.F. performed the research; C.C., N.S., V.D., G.B., and P.P. contributed analytical tools; S.N.K., C.C., R.A., and V.D. analyzed the data; and S.N.K., C.C., G.B., O.L., P.P., and F.F. wrote the manuscript.

## Supporting Material

Document S1. Supporting Materials and Methods, twelve figures, and thirteen tables

Document S2. Article plus Supporting Material
