# Supplementary material for: Distribution of Pico- and Nanosecond Motions in Disordered Proteins from Nuclear Spin Relaxation
Source: Biophys J. 2015 Sep 1;109(5):988–99. doi: 10.1016/j.bpj.2015.06.069 (PMC4564687; doi:10.1016/j.bpj.2015.06.069)
Supplement: Document S1. Supporting Materials and Methods, twelve figures, and thirteen tables [file mmc1.pdf]

Biophysical Journal

Supporting Material

Distribution of Pico- and Nanosecond Motions in Disordered Proteins from Nuclear Spin Relaxation

Shahid N. Khan,[1,2,3] Cyril Charlier,[1,2,3] Rafal Augustyniak,[1,2,3] Nicola Salvi,[4] Victoire Déjean,[1,2,3] Geoffrey Bodenhausen,[1,2,3,4] Olivier Lequin,[1,2,3] Philippe Pelupessy,[1,2,3] and Fabien Ferrage[1,2,3,*]

[1]Département de Chimie, École Normale Supérieure-PSL Research University, Paris, France; [2]Sorbonne Universités, UPMC Univ Paris 06, LBM, Paris, France; [3]Centre National de la Recherche Scientifique, UMR 7203 LBM, Paris, France; and [4]Institut des Sciences et Ingénierie Chimiques, École Polytechnique Fédérale de Lausanne, BCH, Lausanne, Switzerland

Table of contents:

1. Equations for reduced spectral density mapping: 3
2. Nitrogen-15 transverse relaxation rates at 18.8 T: 4
3. Comparison of reduced spectral density mapping methods: 7
4. Spectral density function at zero frequency: 8
5. Comparison of Akaike Information Criteria: 9
6. 1D Optimization of IMPACT 12
7. Correlations of consecutive IMPACT coefficients: 12
8. IMPACT analysis with relaxation data at five magnetic fields using 5 correlation times: 14
9. IMPACT analysis with relaxation data at three magnetic fields: 15
10. Relaxation rates: 17
11. Spectral density mapping results: 25
12. Two correlation-time analysis of the spectral density function: 33
13. Three correlation-time analysis of the spectral density function: 35
14. IMPACT analysis of the spectral density function: 37
15. References: 40

1. Equations for reduced spectral density mapping:

Reduced spectral density mapping is performed with the use of effective frequencies to account for the spectral density at high frequencies (i.e. $\omega_H + \omega_H$. ω_H. and $\omega_H - \omega_H$). The derivation of the effective frequency for the interpretation of the dipolar cross-relaxation between the ^{15}N and ^{1}H nuclei (Equations 8-10 in Farrow et al.(1)) is reproduced here. We assume that:

$$J(\omega) = \lambda + \mu/\omega^2 \quad \text{(S1)}$$

We need to derive A and ω_{eff} so that:

$$6J(\omega_H + \omega_N) - J(\omega_H - \omega_N) = AJ(\omega_{eff}) \quad \text{(S2)}$$

Hence:

$$A = 5 \quad \text{(S3)}$$

and:

$$6/(\omega_H + \omega_N)^2 - 1/(\omega_H - \omega_N)^2 = 5/\omega_{eff}^2 \quad \text{(S4)}$$

Using $\omega_H/\omega_N = \gamma_H/\gamma_N$ we obtain:

$$\omega_{eff} = \{5/[6/(1 + \gamma_N/\gamma_H)^2 - 1/(1 - \gamma_N/\gamma_H)^2]\}^{\frac{1}{2}}\omega_H \quad \text{(S5)}$$

The numeric application gives:

$$\omega_{eff} = 0.870\omega_H \quad \text{(S6)}$$

2. Nitrogen-15 transverse relaxation rates at 18.8 T:

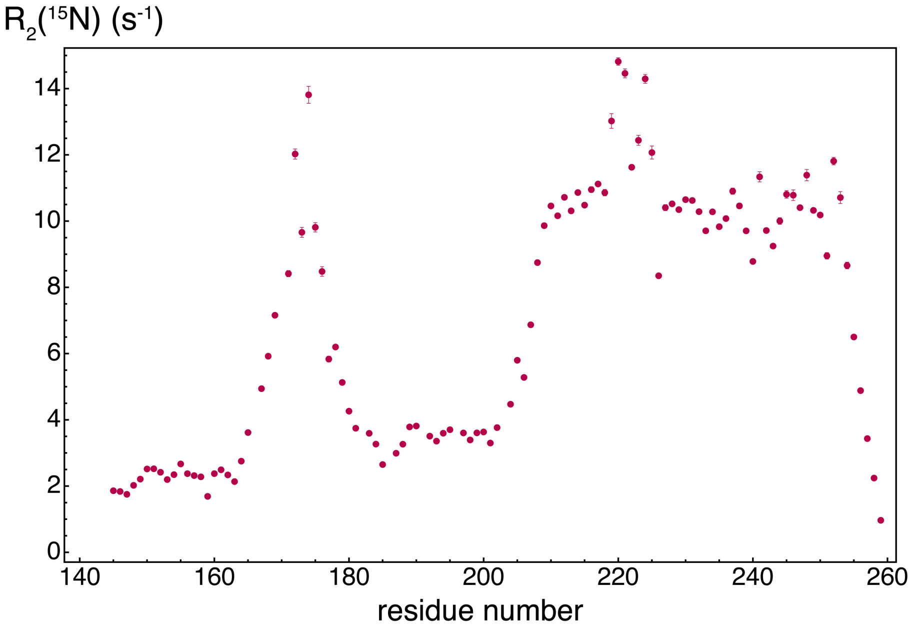

Figure S1. Nitrogen-15 transverse relaxation rates R_2. measured at 18.8 T under a Carr-Purcell-Meiboom-Gill (CPMG) train of 180° pulses and an interpulse delay of 1 ms.

Table S1	R_2 (^{15}N) at 18.8 T (s^{-1})		
residue			
145	1.86	±	0.02
146	1.84	±	0.01
147	1.75	±	0.01
148	2.02	±	0.02
149	2.21	±	0.02
150	2.52	±	0.02
151	2.52	±	0.02
152	2.42	±	0.02
153	2.20	±	0.02
154	2.34	±	0.02
155	2.67	±	0.02
156	2.37	±	0.02
157	2.32	±	0.02
158	2.28	±	0.02
159	1.69	±	0.02
160	2.38	±	0.02
161	2.49	±	0.03
162	2.34	±	0.02
163	2.14	±	0.02
164	2.75	±	0.03
165	3.62	±	0.03
167	4.94	±	0.04
168	5.92	±	0.06
169	7.16	±	0.07
171	8.41	±	0.09

172	12.02	±	0.15
173	9.66	±	0.15
174	13.81	±	0.26
175	9.81	±	0.14
176	8.48	±	0.14
177	5.83	±	0.09
178	6.20	±	0.07
179	5.13	±	0.06
180	4.26	±	0.04
181	3.75	±	0.03
183	3.59	±	0.04
184	3.27	±	0.06
185	2.65	±	0.03
187	2.99	±	0.04
188	3.26	±	0.05
189	3.79	±	0.05
190	3.81	±	0.04
192	3.51	±	0.02
193	3.36	±	0.03
194	3.60	±	0.03
195	3.70	±	0.05
197	3.60	±	0.03
198	3.39	±	0.04
199	3.61	±	0.02
200	3.63	±	0.02
201	3.30	±	0.02
202	3.77	±	0.02
204	4.47	±	0.03
205	5.80	±	0.05
206	5.28	±	0.04
207	6.87	±	0.04
208	8.75	±	0.08
209	9.86	±	0.06
210	10.46	±	0.05
211	10.16	±	0.07
212	10.72	±	0.07
213	10.31	±	0.05
214	10.86	±	0.07
215	10.48	±	0.07
216	10.95	±	0.09
217	11.12	±	0.06
218	10.86	±	0.10
219	13.02	±	0.22
220	14.82	±	0.11
221	14.46	±	0.14
222	11.62	±	0.07
223	12.44	±	0.15
224	14.29	±	0.13
225	12.07	±	0.20
226	8.35	±	0.06
227	10.41	±	0.09
228	10.52	±	0.06
229	10.35	±	0.07
230	10.65	±	0.08
231	10.62	±	0.06
232	10.28	±	0.05
233	9.71	±	0.06
234	10.28	±	0.06
235	9.83	±	0.04
236	10.08	±	0.05
237	10.90	±	0.09
238	10.46	±	0.08
239	9.71	±	0.07

240	8.78	±	0.06
241	11.34	±	0.15
242	9.72	±	0.08
243	9.25	±	0.06
244	10.00	±	0.10
245	10.81	±	0.11
246	10.78	±	0.16
247	10.41	±	0.06
248	11.39	±	0.17
249	10.32	±	0.07
250	10.18	±	0.07
251	8.95	±	0.10
252	11.81	±	0.11
253	10.71	±	0.18
254	8.66	±	0.10
255	6.50	±	0.04
256	4.88	±	0.03
257	3.43	±	0.02
258	2.24	±	0.02
259	0.97	±	0.01

3. Comparison of reduced spectral density mapping methods:

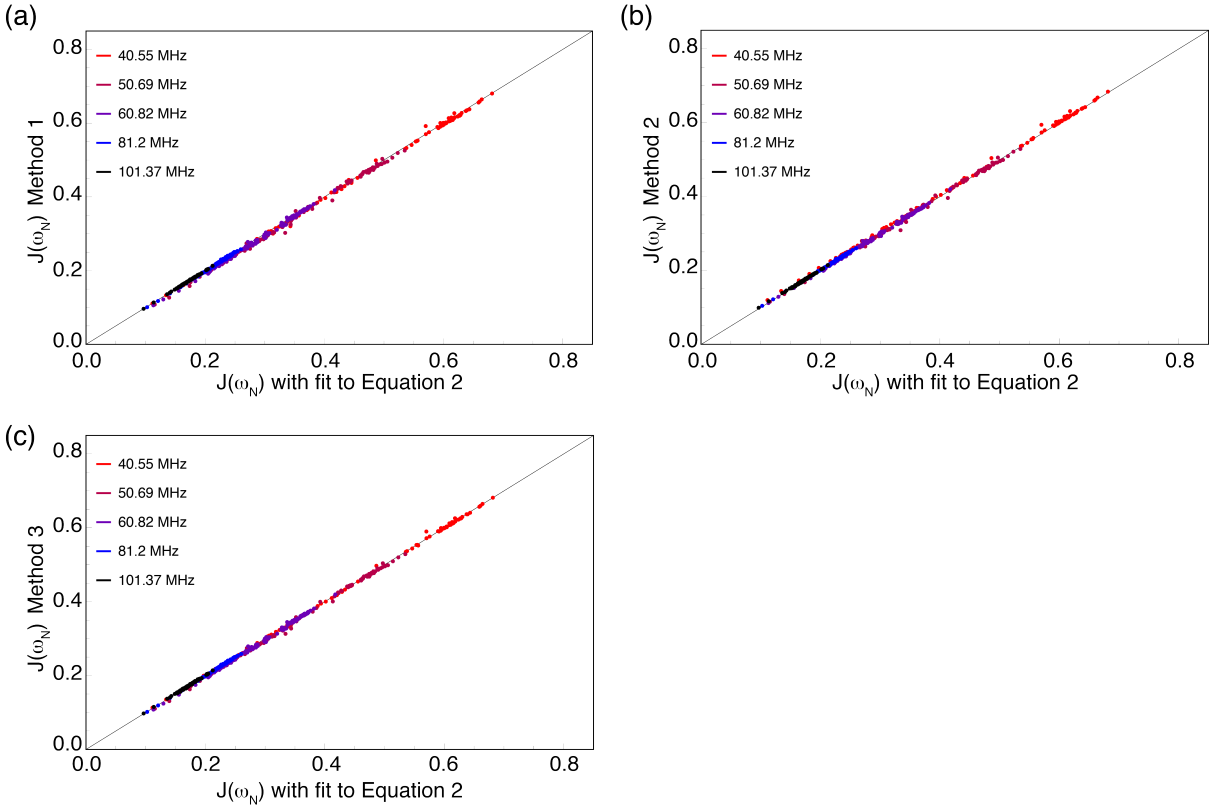

Figure S2. Comparison of the spectral density at the Larmor frequency of nitrogen-15 for a series of approximations. We compare the results obtained in the present study, with a fit of the spectral density function at high frequency with the function of equation 2 with results obtained with the three methods presented in the original reduced spectral density approach.(1) The spectral density function at high frequency (i.e. near ω_H) was derived following: (a) method 1. where for each dataset. the spectral density function is considered to be constant (i.e. $J(\omega_H) = J(\omega_H + \omega_N) = J(\omega_H - \omega_N) = J(0.87\omega_H)$); (b) method 2, where the spectral density at high frequency is derived from $J(0.87\omega_H)$ assuming that $J(\omega) \propto 1/\omega^2$; (c) method 3, where the spectral density at high frequency is derived from the values of $J(0.87\omega_H)$ at two magnetic fields following a linear approximation.

4. Spectral density function at zero frequency:

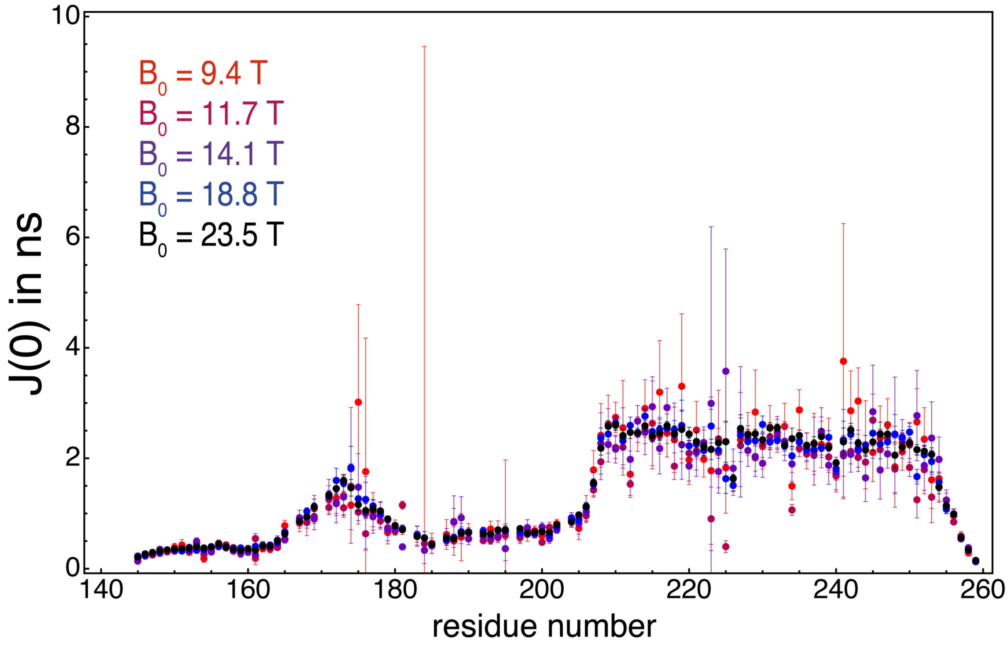

Figure S3. Spectral density function at zero frequency derived from relaxation rates measured at all five magnetic fields. The data obtained at the two highest fields, shown in Figure 2.c are complemented by the values obtained at the three lower fields.

5. Comparison of Akaike Information Criteria:

$$AICc = AIC + \frac{2n_{model}(n_{model}+1)}{n_{exp} - n_{model} - 1}$$

with

$$AIC = n_{exp} ln\left(\frac{\chi^2}{n_{exp}}\right) + 2n_{model} + C$$

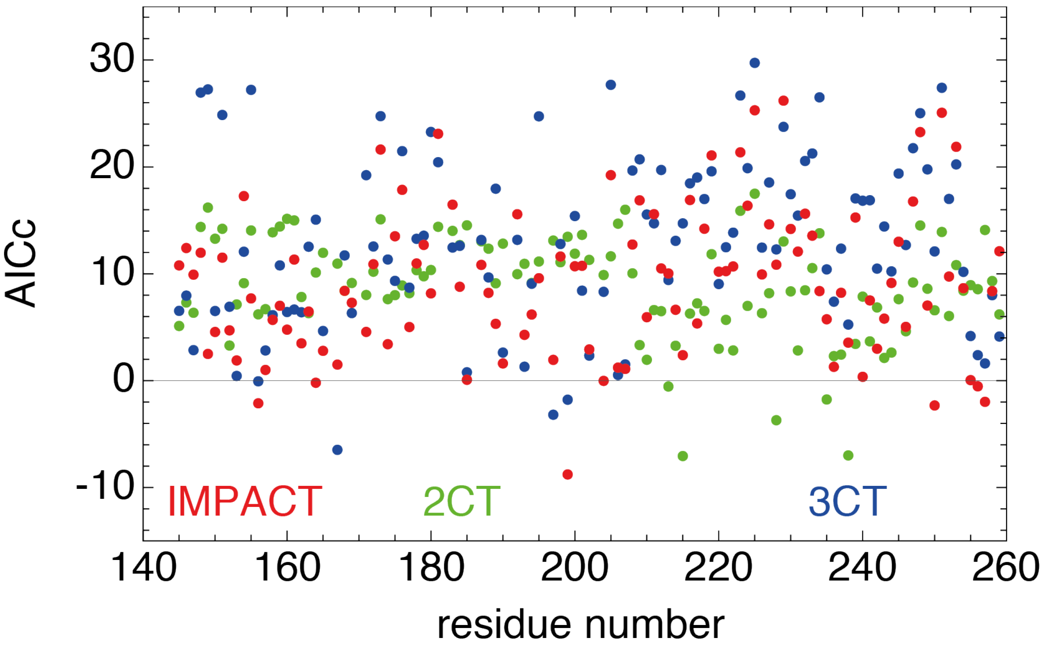

Figure S4. Second order variant of the Akaike Information Criteria AICc's obtained in the IMPACT (red), the two correlation-time (green) and the three correlation-time analyses (blue). Here, n_j = 11, n_{model} = 5 for IMPACT n_{model} = 3 for 2CT and n_{model} = 5 for 3CT analysis.

**Table S2**	AICc		
Residue number	2CT	3CT	IMPACT
145	5.11	6.53	10.78
146	7.31	7.95	12.40
147	6.35	2.85	9.91
148	14.38	26.95	11.97
149	16.19	27.25	2.50
150	13.28	6.51	4.55
151	14.21	24.86	11.51
152	3.28	6.91	4.70
153	7.13	0.44	1.89
154	9.12	12.06	17.27
155	14.05	27.21	7.69
156	6.20	-0.06	-2.12

157	6.69	2.82	1.00
158	13.88	6.12	5.68
159	14.42	10.78	7.00
160	15.14	6.42	4.77
161	14.99	6.65	11.33
162	7.83	6.40	3.49
163	6.31	12.53	6.46
164	10.12	15.06	-0.20
165	11.96	4.64	2.79
167	10.95	-6.48	1.50
168	8.40	11.71	8.40
169	9.14	6.32	7.29
171	8.01	19.22	4.56
172	10.19	12.55	10.89
173	15.09	24.75	21.62
174	7.62	11.33	3.41
175	8.00	9.33	13.51
176	8.90	21.47	17.85
177	8.18	8.70	5.01
178	10.33	13.27	10.96
179	9.76	13.56	12.70
180	10.35	23.27	8.16
181	14.40	20.44	23.10
183	14.01	12.45	16.46
184	12.69	12.61	8.78
185	14.53	0.78	0.08
187	13.03	13.16	10.83
188	12.35	9.66	8.21
189	9.11	17.96	5.31
190	12.82	2.62	1.61
192	9.96	13.17	15.57
193	10.93	1.30	4.28
194	9.14	9.07	6.19
195	11.15	24.73	9.58
197	13.11	-3.19	1.94
198	11.08	12.79	11.61
199	13.46	-1.79	-8.78
200	11.88	15.40	10.70
201	13.64	8.42	10.74
202	11.30	2.34	2.92
204	9.88	8.31	-0.02
205	11.63	27.68	19.23
206	14.69	0.53	1.22
207	15.99	1.52	1.09
208	10.04	19.66	12.74
209	3.33	20.71	16.87
210	1.95	15.54	5.94
211	6.61	14.72	15.58
212	6.50	19.71	10.50
213	-0.55	9.42	10.03

214	3.26	13.08	6.63
215	-7.06	14.71	2.38
216	6.27	18.46	16.90
217	7.23	19.01	5.35
218	6.52	16.99	14.21
219	11.83	19.59	21.07
220	2.98	9.04	10.20
221	5.69	12.48	10.24
222	2.83	13.85	10.69
223	15.89	26.68	21.36
224	6.99	19.88	16.37
225	17.49	29.73	25.30
226	6.31	12.45	9.94
227	8.18	18.54	14.62
228	-3.71	12.28	10.85
229	13.02	23.75	26.20
230	8.35	17.44	14.20
231	2.82	15.44	12.08
232	8.45	20.56	15.62
233	10.53	21.26	13.56
234	13.79	26.51	8.38
235	-1.77	10.41	5.74
236	2.29	7.39	1.29
237	2.43	12.35	8.22
238	-7.00	5.25	3.55
239	3.43	17.05	15.26
240	7.86	16.84	0.36
241	3.67	16.88	7.50
242	6.85	10.48	2.98
243	2.13	14.42	5.80
244	2.63	10.22	9.14
245	7.62	19.38	13.00
246	4.62	12.69	5.03
247	9.18	21.75	16.76
248	14.52	25.03	23.26
249	8.60	19.77	7.02
250	6.58	12.09	-2.32
251	13.91	27.40	25.07
252	6.04	17.00	9.74
253	10.81	20.23	21.88
254	8.41	10.18	8.65
255	8.94	4.17	0.05
256	8.56	2.38	-0.54
257	14.09	1.61	-1.99
258	9.32	7.99	8.37
259	6.20	4.12	12.11

6. 1D Optimization of IMPACT

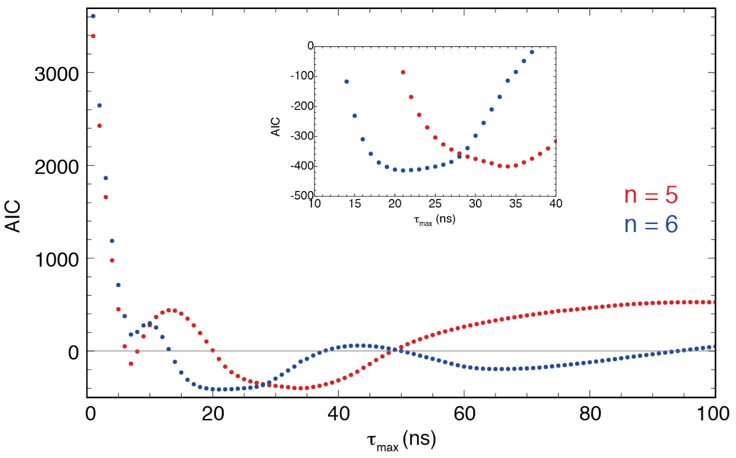

Figure S5. 1D optimization of IMPACT using data at 5 magnetic fields using 5 correlation times (red) of 6 correlation times (bleue).

7. Correlations of consecutive IMPACT coefficients:

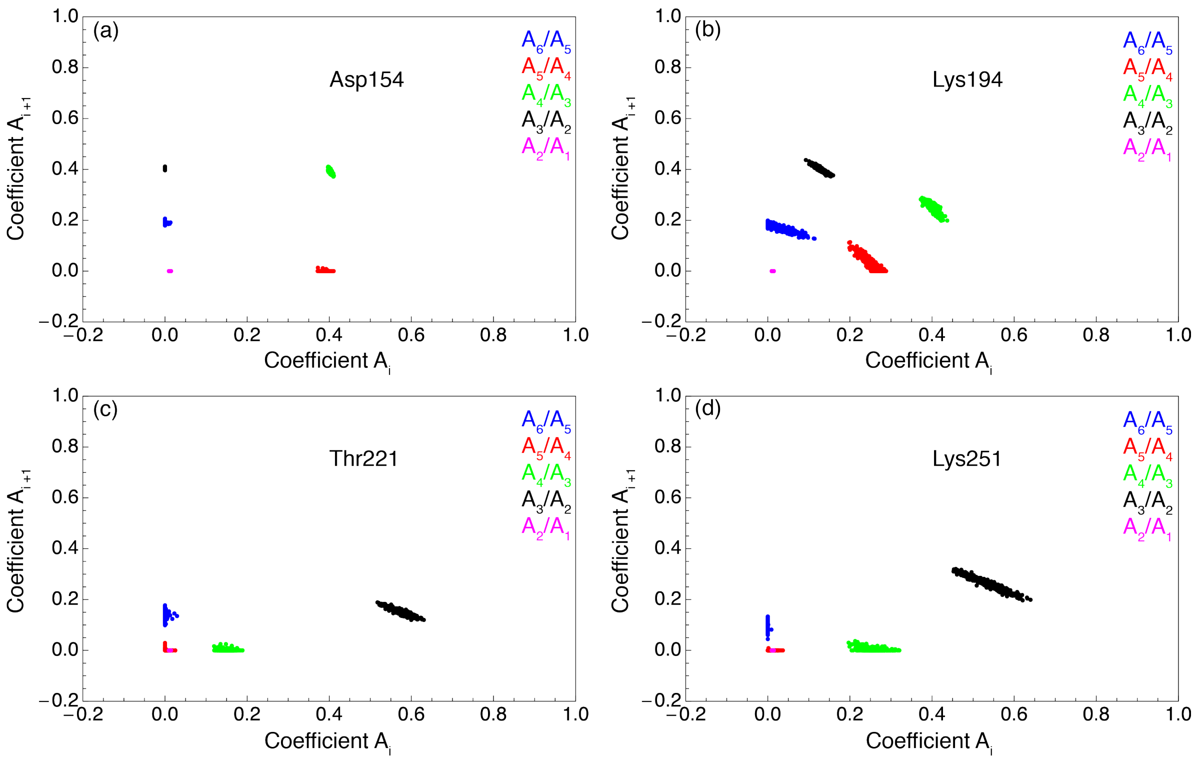

Figure S6. Correlation of consecutive IMPACT coefficients. A_i coefficients are displayed on the x-axis as a function of A_{i+1} coefficients displayed on the y-axis: A_2 as a function of A_1 (magenta); A_3 as a function of A_2 (black); A_4 as a function of A_3 (green); A_5 as a function of

A_4 (red); A_6 as a function of A_5 (blue). Typical results are shown for residues in different regions of the protein: Asp154; Lys194; Thr221; Lys251.

8. IMPACT analysis with relaxation data at five magnetic fields using 5 correlation times:

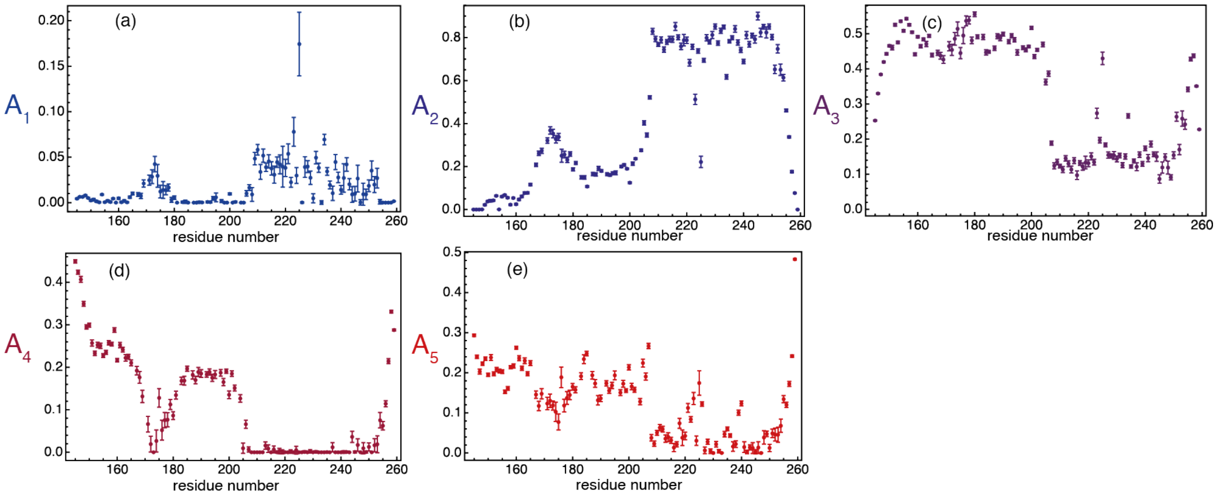

Figure S7. IMPACT results with relaxation rates measured at five magnetic fields. The number of correlation times was n = 5 and the range of correlation times was [34 ps. 34 ns]. (a) A_1 with τ_1 = 34 ns; (b) A_2 with τ_2 = 6.04 ns; (c) A_3 with τ_3 = 1.08 ns; (d) A_4 with τ_4 = 191.2 ps; (e) A_5 with τ_5 = 34 ps.

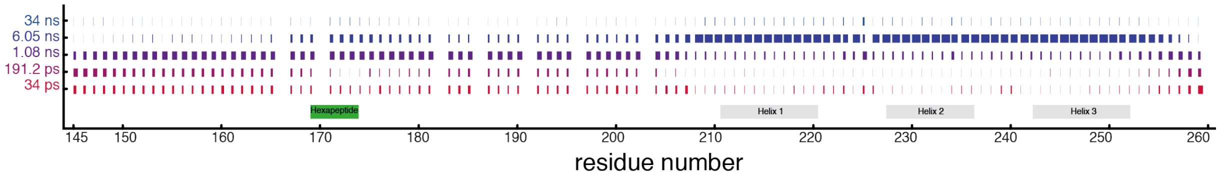

Figure S8. Bar-code representation of the IMPACT analysis of the spectral density function in Engrailed. Histograms are drawn for all residues with the following rules: for each correlation time obtained or used in the analysis of the spectral density function τ_i. a rectangle is represented at the corresponding position along the y-axis. with a logarithmic scale; the width of each rectangle is proportional to the corresponding weight. A_i. The main structural features are illustrated by grey rectangles for alpha helices and a green rectangle for the location of the hydrophobic hexapeptide.

9. IMPACT analysis with relaxation data at three magnetic fields:

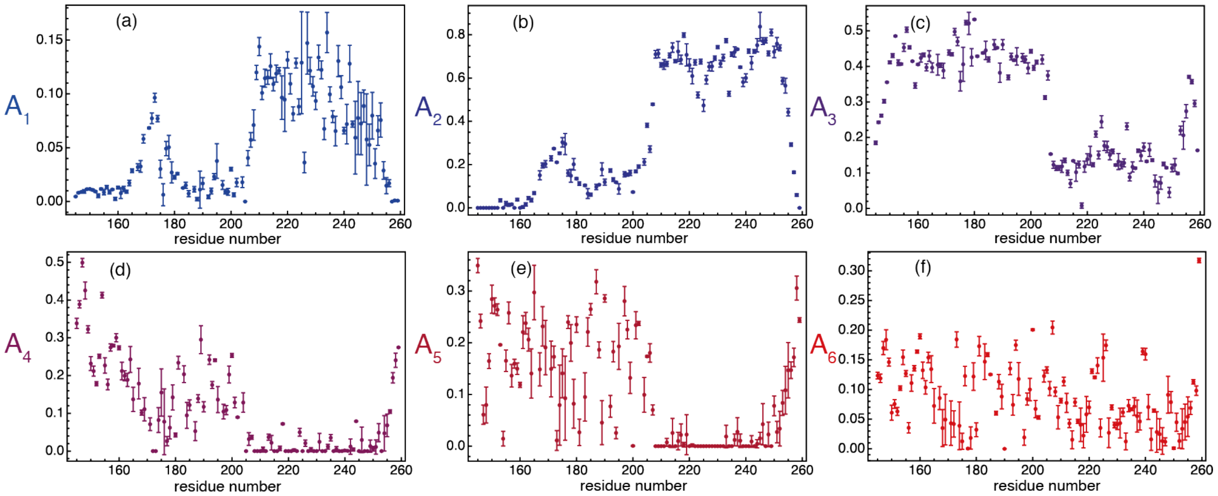

Figure S9. IMPACT results with relaxation rates measured at three magnetic fields: 9.4 T; 14.1 T; and 23.5 T. As for the results presented in Figure 4. the number of correlation times was n = 6 and the range of correlation times was [21 ps. 21 ns]. (a) A_1 with τ_1 = 21 ns; (b) A_2 with τ_2 = 5.27 ns; (c) A_3 with τ_3 = 1.33 ns; (d) A_4 with τ_4 = 333 ps; (e) A_5 with τ_5 = 83.6 ps; (e) A_6 with τ_6 = 21 ps.

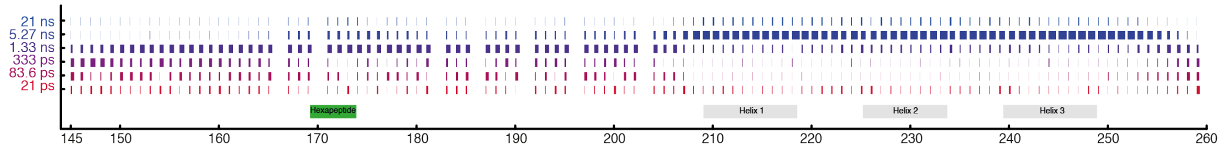

Figure S10. Bar-code representation of the IMPACT analysis of the spectral density function in Engrailed. Histograms are drawn for all residues with the following rules: for each correlation time obtained or used in the analysis of the spectral density function τ_i. a rectangle is represented at the corresponding position along the y-axis. with a logarithmic scale; the width of each rectangle is proportional to the corresponding weight. A_i. The main structural features are illustrated by grey rectangles for alpha helices and a green rectangle for the location of the hydrophobic hexapeptide.

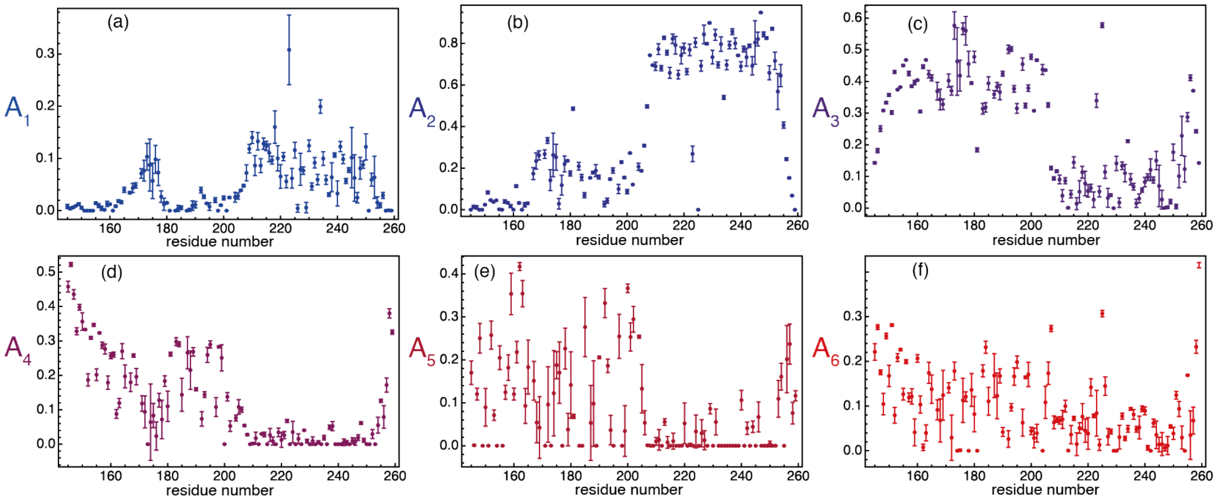

Figure S11. IMPACT results with relaxation rates measured at three magnetic fields: 11.7 T; 14.1 T; and 18.8 T. As for the results presented in Figure 4. the number of correlation times was n = 6 and the range of correlation times was [21 ps. 21 ns]. (a) A_1 with τ_1 = 21 ns; (b) A_2 with τ_2 = 5.27 ns; (c) A_3 with τ_3 = 1.33 ns; (d) A_4 with τ_4 = 333 ps; (e) A_5 with τ_5 = 83.6 ps; (e) A_6 with τ_6 = 21 ps.

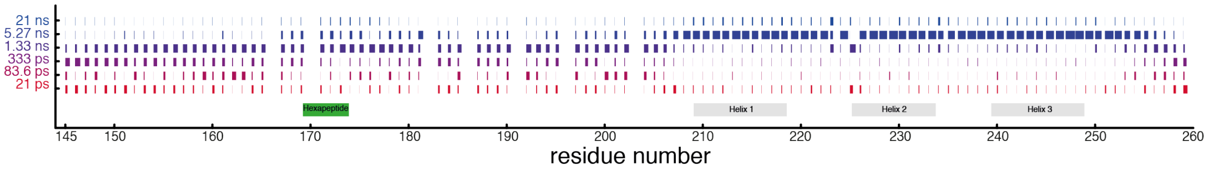

Figure S12. Bar-code representation of the IMPACT analysis of the spectral density function in Engrailed. Histograms are drawn for all residues with the following rules: for each correlation time obtained or used in the analysis of the spectral density function τ_i. a rectangle is represented at the corresponding position along the y-axis. with a logarithmic scale; the width of each rectangle is proportional to the corresponding weight. A_i. The main structural features are illustrated by grey rectangles for alpha helices and a green rectangle for the location of the hydrophobic hexapeptide.

10. Relaxation rates:

Table S3: Nitrogen-15 longitudinal relaxation rates $R_1(^{15}N)$ (s^{-1})

residue	9.4 T			11.8 T			14.1 T			18.8 T			23.5 T		
145	0.956	±	0.007	0.926	±	0.006	0.882	±	0.012	0.911	±	0.002	0.993	±	0.003
146	1.096	±	0.006	1.085	±	0.006	1.008	±	0.011	1.022	±	0.002	1.144	±	0.003
147	1.208	±	0.007	1.111	±	0.008	1.150	±	0.012	1.121	±	0.002	1.227	±	0.003
148	1.246	±	0.009	1.160	±	0.007	1.238	±	0.014	1.168	±	0.003	1.243	±	0.003
149	1.293	±	0.007	1.225	±	0.008	1.238	±	0.014	1.207	±	0.003	1.279	±	0.003
150	1.361	±	0.009	1.310	±	0.009	1.308	±	0.018	1.273	±	0.004	1.344	±	0.004
151	1.379	±	0.011	1.323	±	0.010	1.268	±	0.023	1.188	±	0.004	1.359	±	0.005
152	1.430	±	0.009	1.395	±	0.008	1.388	±	0.018	1.347	±	0.003	1.444	±	0.004
153	1.464	±	0.008	1.335	±	0.008	1.323	±	0.016	1.278	±	0.003	1.374	±	0.004
154	1.449	±	0.009	1.510	±	0.050	1.370	±	0.016	1.308	±	0.003	1.440	±	0.004
155	1.511	±	0.013	1.362	±	0.010	1.296	±	0.019	1.329	±	0.004	1.415	±	0.004
156	1.557	±	0.010	1.455	±	0.013	1.413	±	0.019	1.413	±	0.003	1.495	±	0.004
157	1.516	±	0.010	1.402	±	0.010	1.433	±	0.020	1.384	±	0.004	1.444	±	0.004
158	1.371	±	0.007	1.325	±	0.008	1.359	±	0.015	1.307	±	0.003	1.365	±	0.004
159	1.363	±	0.010	1.295	±	0.009	1.192	±	0.020	1.264	±	0.004	1.269	±	0.005
160	1.331	±	0.010	1.242	±	0.008	1.251	±	0.019	1.274	±	0.003	1.317	±	0.004
161	1.360	±	0.017	1.313	±	0.013	1.304	±	0.033	1.231	±	0.005	1.342	±	0.006
162	1.398	±	0.011	1.354	±	0.013	1.350	±	0.021	1.311	±	0.003	1.361	±	0.005
163	1.400	±	0.011	1.335	±	0.010	1.260	±	0.023	1.338	±	0.005	1.294	±	0.005
164	1.498	±	0.013	1.386	±	0.011	1.382	±	0.026	1.331	±	0.005	1.383	±	0.005
165	1.462	±	0.013	1.330	±	0.015	1.355	±	0.027	1.283	±	0.005	1.304	±	0.005
167	1.742	±	0.020	1.569	±	0.019	1.454	±	0.035	1.369	±	0.007	1.400	±	0.007
168	1.798	±	0.025	1.683	±	0.023	1.551	±	0.046	1.443	±	0.009	1.400	±	0.009
169	1.819	±	0.025	1.680	±	0.023	1.499	±	0.050	1.391	±	0.010	1.369	±	0.010
171	2.000	±	0.029	1.806	±	0.027	1.588	±	0.059	1.468	±	0.013	1.463	±	0.013
172	2.012	±	0.036	1.873	±	0.033	1.727	±	0.082	1.507	±	0.017	1.461	±	0.017
173	1.883	±	0.046	1.689	±	0.036	1.557	±	0.089	1.606	±	0.020	1.599	±	0.021
174	2.103	±	0.044	1.837	±	0.056	1.760	±	0.112	1.578	±	0.027	1.536	±	0.027
175	2.052	±	0.045	1.760	±	0.036	1.849	±	0.111	1.521	±	0.018	1.457	±	0.017
176	2.119	±	0.156	1.560	±	0.052	1.477	±	0.111	1.468	±	0.020	1.420	±	0.018
177	1.904	±	0.039	1.762	±	0.032	1.638	±	0.079	1.561	±	0.014	1.543	±	0.014
178	1.817	±	0.032	1.744	±	0.030	1.754	±	0.064	1.528	±	0.011	1.543	±	0.011
179	1.832	±	0.031	1.701	±	0.027	1.708	±	0.060	1.473	±	0.009	1.442	±	0.010
180	1.840	±	0.017	1.574	±	0.014	1.611	±	0.037	1.514	±	0.006	1.542	±	0.007
181	1.748	±	0.015	1.969	±	0.016	1.270	±	0.025	1.455	±	0.005	1.427	±	0.005
183	1.628	±	0.023	1.585	±	0.019	1.554	±	0.049	1.339	±	0.008	1.428	±	0.008
184	1.503	±	0.032	1.472	±	0.023	1.334	±	0.061	1.297	±	0.010	1.329	±	0.010
185	1.450	±	0.014	1.358	±	0.013	1.304	±	0.030	1.266	±	0.005	1.315	±	0.006
187	1.576	±	0.020	1.551	±	0.019	1.489	±	0.045	1.349	±	0.007	1.381	±	0.008
188	1.640	±	0.027	1.620	±	0.024	1.500	±	0.053	1.413	±	0.008	1.419	±	0.009
189	1.834	±	0.038	1.646	±	0.032	1.580	±	0.069	1.412	±	0.009	1.461	±	0.010
190	1.717	±	0.020	1.615	±	0.022	1.531	±	0.040	1.450	±	0.006	1.471	±	0.007
192	1.725	±	0.015	1.391	±	0.012	1.563	±	0.027	1.399	±	0.004	1.388	±	0.005
193	1.693	±	0.018	1.487	±	0.017	1.486	±	0.033	1.433	±	0.006	1.430	±	0.006
194	1.699	±	0.020	1.542	±	0.017	1.532	±	0.038	1.382	±	0.006	1.413	±	0.007
195	1.586	±	0.032	1.525	±	0.021	1.394	±	0.058	1.318	±	0.008	1.360	±	0.009
197	1.743	±	0.020	1.511	±	0.014	1.473	±	0.036	1.404	±	0.006	1.420	±	0.006
198	1.712	±	0.019	1.626	±	0.016	1.569	±	0.038	1.382	±	0.006	1.384	±	0.007
199	1.740	±	0.012	1.562	±	0.010	1.476	±	0.021	1.384	±	0.003	1.406	±	0.004
200	1.623	±	0.009	1.431	±	0.008	1.502	±	0.024	1.389	±	0.004	1.417	±	0.005
201	1.721	±	0.015	1.753	±	0.014	1.459	±	0.027	1.344	±	0.004	1.378	±	0.005
202	1.801	±	0.014	1.559	±	0.011	1.468	±	0.022	1.422	±	0.004	1.401	±	0.005
204	1.899	±	0.018	1.706	±	0.013	1.554	±	0.033	1.491	±	0.005	1.434	±	0.006
205	1.869	±	0.023	1.664	±	0.019	1.582	±	0.050	1.454	±	0.008	1.347	±	0.007
206	1.891	±	0.023	1.702	±	0.020	1.513	±	0.044	1.358	±	0.007	1.326	±	0.007
207	2.018	±	0.023	1.666	±	0.018	1.375	±	0.035	1.150	±	0.005	1.057	±	0.005

208	2.572	±	0.049	2.148	±	0.032	1.719	±	0.069	1.397	±	0.010	1.205	±	0.009
209	2.501	±	0.037	2.103	±	0.025	1.815	±	0.058	1.370	±	0.007	1.199	±	0.008
210	2.537	±	0.033	2.030	±	0.022	1.641	±	0.044	1.321	±	0.006	1.128	±	0.006
211	2.428	±	0.049	2.111	±	0.032	1.754	±	0.070	1.324	±	0.008	1.147	±	0.008
212	2.551	±	0.039	1.997	±	0.027	1.762	±	0.059	1.300	±	0.008	1.181	±	0.007
213	2.657	±	0.032	2.179	±	0.022	1.794	±	0.043	1.338	±	0.005	1.178	±	0.006
214	2.648	±	0.045	2.109	±	0.027	1.812	±	0.064	1.333	±	0.008	1.152	±	0.008
215	2.621	±	0.080	2.032	±	0.042	1.707	±	0.064	1.376	±	0.008	1.193	±	0.008
216	2.884	±	0.350	2.227	±	0.035	1.675	±	0.065	1.363	±	0.009	1.180	±	0.009
217	2.622	±	0.038	2.214	±	0.027	1.783	±	0.057	1.371	±	0.007	1.183	±	0.007
218	2.672	±	0.048	1.943	±	0.029	1.817	±	0.075	1.319	±	0.010	1.121	±	0.010
219	2.536	±	0.061	2.156	±	0.054	1.749	±	0.119	1.360	±	0.021	1.232	±	0.022
220	2.540	±	0.041	2.175	±	0.031	1.698	±	0.066	1.355	±	0.010	1.178	±	0.010
221	2.384	±	0.044	1.911	±	0.036	1.639	±	0.077	1.219	±	0.012	1.153	±	0.011
222	2.474	±	0.034	2.114	±	0.024	1.728	±	0.054	1.325	±	0.008	1.154	±	0.007
223	1.967	±	0.120	1.583	±	0.049	1.734	±	0.086	1.314	±	0.015	1.192	±	0.014
224	2.655	±	0.044	2.287	±	0.087	1.748	±	0.079	1.412	±	0.012	1.241	±	0.012
225	2.210	±	0.064	1.361	±	0.019	1.416	±	0.117	1.374	±	0.021	1.235	±	0.028
226	2.346	±	0.039	2.038	±	0.029	1.799	±	0.065	1.351	±	0.008	1.178	±	0.008
227	2.596	±	0.056	2.268	±	0.030	1.636	±	0.077	1.398	±	0.011	1.261	±	0.012
228	2.585	±	0.033	2.188	±	0.022	1.770	±	0.052	1.374	±	0.007	1.209	±	0.007
229	2.496	±	0.047	2.362	±	0.035	1.833	±	0.070	1.416	±	0.009	1.281	±	0.009
230	2.725	±	0.043	2.283	±	0.028	1.707	±	0.063	1.491	±	0.009	1.239	±	0.009
231	2.661	±	0.037	2.183	±	0.024	1.798	±	0.053	1.370	±	0.007	1.192	±	0.007
232	2.607	±	0.030	2.161	±	0.020	1.750	±	0.043	1.414	±	0.005	1.185	±	0.006
233	2.794	±	0.042	2.338	±	0.027	1.841	±	0.059	1.486	±	0.008	1.296	±	0.009
234	2.457	±	0.034	1.941	±	0.017	1.696	±	0.059	1.428	±	0.007	1.232	±	0.007
235	2.619	±	0.031	2.123	±	0.019	1.731	±	0.041	1.325	±	0.005	1.151	±	0.005
236	2.603	±	0.030	2.106	±	0.020	1.864	±	0.043	1.387	±	0.006	1.194	±	0.006
237	2.694	±	0.053	2.257	±	0.036	1.821	±	0.077	1.409	±	0.011	1.206	±	0.009
238	2.590	±	0.043	2.192	±	0.030	1.803	±	0.073	1.367	±	0.010	1.235	±	0.009
239	2.361	±	0.039	2.020	±	0.026	1.510	±	0.055	1.319	±	0.008	1.104	±	0.006
240	2.392	±	0.033	1.979	±	0.024	1.641	±	0.050	1.303	±	0.007	1.179	±	0.007
241	2.637	±	0.060	2.206	±	0.042	1.819	±	0.109	1.429	±	0.017	1.226	±	0.017
242	2.653	±	0.042	2.162	±	0.026	1.897	±	0.071	1.452	±	0.010	1.269	±	0.010
243	2.695	±	0.041	2.203	±	0.027	1.804	±	0.060	1.426	±	0.008	1.245	±	0.008
244	2.708	±	0.055	2.151	±	0.034	1.821	±	0.084	1.441	±	0.012	1.220	±	0.012
245	2.791	±	0.057	2.269	±	0.045	1.680	±	0.086	1.427	±	0.013	1.189	±	0.012
246	2.726	±	0.055	2.235	±	0.047	1.777	±	0.093	1.374	±	0.018	1.209	±	0.016
247	2.819	±	0.042	2.424	±	0.028	1.844	±	0.057	1.473	±	0.007	1.287	±	0.008
248	2.591	±	0.065	2.300	±	0.056	1.688	±	0.108	1.382	±	0.018	1.199	±	0.017
249	2.744	±	0.038	2.183	±	0.027	1.783	±	0.057	1.348	±	0.008	1.146	±	0.008
250	2.693	±	0.038	2.158	±	0.025	1.846	±	0.057	1.458	±	0.008	1.225	±	0.008
251	2.695	±	0.054	1.785	±	0.033	2.078	±	0.095	1.479	±	0.013	1.266	±	0.018
252	2.707	±	0.047	2.068	±	0.055	1.726	±	0.076	1.370	±	0.012	1.241	±	0.011
253	2.477	±	0.040	1.966	±	0.097	1.668	±	0.121	1.483	±	0.020	1.282	±	0.025
254	2.381	±	0.040	2.022	±	0.038	1.846	±	0.077	1.399	±	0.013	1.297	±	0.013
255	2.189	±	0.021	1.836	±	0.018	1.701	±	0.038	1.412	±	0.006	1.327	±	0.007
256	2.001	±	0.017	1.734	±	0.013	1.636	±	0.030	1.476	±	0.005	1.405	±	0.006
257	1.670	±	0.013	1.513	±	0.010	1.425	±	0.022	1.352	±	0.004	1.331	±	0.005
258	1.294	±	0.008	1.193	±	0.007	1.220	±	0.015	1.117	±	0.003	1.176	±	0.004
259	0.783	±	0.003	0.762	±	0.003	0.756	±	0.005	0.769	±	0.001	0.843	±	0.001

Table S4: Longitudinal cross-correlated cross-relaxation rates η_z (s^{-1})

residue	9.4 T	11.8 T	14.1 T	18.8 T	23.5 T
145	0.276 ± 0.037	0.315 ± 0.016	0.366 ± 0.029	0.437 ± 0.012	0.543 ± 0.007
146	0.357 ± 0.024	0.415 ± 0.013	0.465 ± 0.020	0.583 ± 0.008	0.654 ± 0.005
147	0.395 ± 0.027	0.527 ± 0.017	0.526 ± 0.021	0.654 ± 0.009	0.748 ± 0.005
148	0.408 ± 0.035	0.603 ± 0.021	0.548 ± 0.027	0.676 ± 0.012	0.747 ± 0.006
149	0.457 ± 0.029	0.555 ± 0.018	0.659 ± 0.024	0.723 ± 0.011	0.820 ± 0.006
150	0.481 ± 0.042	0.585 ± 0.022	0.653 ± 0.039	0.760 ± 0.016	0.822 ± 0.009
151	0.494 ± 0.069	0.572 ± 0.029	0.662 ± 0.064	0.723 ± 0.024	0.827 ± 0.013
152	0.613 ± 0.040	0.675 ± 0.020	0.739 ± 0.034	0.839 ± 0.013	0.942 ± 0.008
153	0.541 ± 0.037	0.615 ± 0.019	0.657 ± 0.031	0.835 ± 0.012	0.849 ± 0.007
154	0.628 ± 0.065	0.452 ± 0.036	0.798 ± 0.029	0.883 ± 0.013	0.969 ± 0.007
155	0.577 ± 0.050	0.852 ± 0.033	0.811 ± 0.045	0.797 ± 0.017	0.898 ± 0.009
156	0.625 ± 0.048	0.698 ± 0.034	0.819 ± 0.040	0.906 ± 0.015	0.968 ± 0.009
157	0.613 ± 0.044	0.683 ± 0.023	0.762 ± 0.038	0.884 ± 0.016	0.948 ± 0.009
158	0.536 ± 0.030	0.643 ± 0.019	0.682 ± 0.026	0.832 ± 0.012	0.900 ± 0.006
159	0.423 ± 0.043	0.488 ± 0.023	0.629 ± 0.043	0.605 ± 0.017	0.684 ± 0.010
160	0.454 ± 0.063	0.594 ± 0.022	0.688 ± 0.047	0.791 ± 0.017	0.849 ± 0.001
161	0.598 ± 0.139	0.530 ± 0.046	0.738 ± 0.112	0.746 ± 0.036	0.879 ± 0.021
162	0.556 ± 0.044	0.641 ± 0.053	0.708 ± 0.040	0.780 ± 0.016	0.839 ± 0.009
163	0.524 ± 0.044	0.568 ± 0.024	0.639 ± 0.043	0.686 ± 0.019	0.738 ± 0.010
164	0.631 ± 0.064	0.698 ± 0.029	0.717 ± 0.053	0.833 ± 0.021	0.900 ± 0.012
165	0.506 ± 0.057	0.673 ± 0.035	0.797 ± 0.051	0.775 ± 0.021	0.837 ± 0.011
167	0.767 ± 0.084	0.782 ± 0.045	0.834 ± 0.069	0.883 ± 0.031	0.931 ± 0.015
168	0.864 ± 0.111	0.914 ± 0.057	0.940 ± 0.094	0.921 ± 0.040	1.022 ± 0.019
169	0.756 ± 0.117	0.901 ± 0.060	0.962 ± 0.102	0.945 ± 0.047	0.949 ± 0.024
171	1.015 ± 0.131	1.024 ± 0.073	1.086 ± 0.123	1.089 ± 0.066	1.066 ± 0.034
172	1.054 ± 0.158	1.046 ± 0.088	1.083 ± 0.158	0.979 ± 0.079	1.033 ± 0.040
173	1.157 ± 0.185	1.264 ± 0.104	1.233 ± 0.195	1.099 ± 0.089	1.091 ± 0.048
174	1.001 ± 0.240	0.916 ± 0.159	0.988 ± 0.228	1.048 ± 0.125	1.109 ± 0.070
175	0.668 ± 0.260	0.921 ± 0.106	0.954 ± 0.223	0.940 ± 0.099	1.040 ± 0.050
176	0.887 ± 0.463	0.937 ± 0.190	1.140 ± 0.354	0.985 ± 0.141	1.049 ± 0.076
177	1.056 ± 0.223	1.023 ± 0.102	1.173 ± 0.190	1.033 ± 0.074	1.103 ± 0.040
178	0.991 ± 0.150	0.936 ± 0.075	1.030 ± 0.141	1.000 ± 0.055	1.015 ± 0.028
179	1.009 ± 0.172	0.979 ± 0.084	0.976 ± 0.149	1.002 ± 0.052	0.993 ± 0.029
180	0.856 ± 0.075	1.082 ± 0.045	1.088 ± 0.074	1.053 ± 0.029	1.080 ± 0.015
181	0.669 ± 0.051	1.180 ± 0.048	1.084 ± 0.050	0.959 ± 0.018	1.003 ± 0.009
183	0.694 ± 0.156	0.743 ± 0.063	0.936 ± 0.141	0.864 ± 0.051	0.943 ± 0.013
184	0.367 ± 0.627	0.735 ± 0.100	1.095 ± 0.312	0.787 ± 0.100	0.858 ± 0.060
185	0.517 ± 0.081	0.586 ± 0.038	0.578 ± 0.082	0.690 ± 0.029	0.770 ± 0.017
187	0.694 ± 0.145	0.764 ± 0.061	0.878 ± 0.127	0.915 ± 0.044	0.924 ± 0.024
188	0.843 ± 0.213	0.653 ± 0.115	0.822 ± 0.199	0.903 ± 0.060	0.982 ± 0.037
189	0.759 ± 0.230	0.803 ± 0.123	0.811 ± 0.227	0.927 ± 0.074	0.976 ± 0.045
190	0.785 ± 0.099	0.873 ± 0.059	0.839 ± 0.081	0.907 ± 0.029	0.930 ± 0.017
192	0.734 ± 0.065	0.820 ± 0.032	0.904 ± 0.052	0.878 ± 0.019	0.972 ± 0.011
193	0.732 ± 0.088	0.808 ± 0.046	0.902 ± 0.074	0.909 ± 0.027	0.967 ± 0.016
194	0.687 ± 0.107	0.837 ± 0.048	0.914 ± 0.090	0.906 ± 0.030	0.956 ± 0.019
195	0.880 ± 0.379	0.797 ± 0.097	1.168 ± 0.286	0.812 ± 0.089	0.862 ± 0.064
197	0.785 ± 0.110	0.836 ± 0.045	0.930 ± 0.098	0.967 ± 0.032	0.912 ± 0.019
198	0.753 ± 0.100	0.738 ± 0.041	0.828 ± 0.082	0.944 ± 0.031	0.943 ± 0.019
199	0.682 ± 0.046	0.782 ± 0.023	0.856 ± 0.039	0.883 ± 0.014	0.935 ± 0.008
200	0.691 ± 0.033	1.039 ± 0.029	0.920 ± 0.048	0.941 ± 0.017	0.986 ± 0.010
201	0.810 ± 0.060	1.108 ± 0.049	0.876 ± 0.052	0.934 ± 0.018	0.934 ± 0.011
202	0.778 ± 0.051	0.992 ± 0.032	0.869 ± 0.041	0.907 ± 0.015	0.932 ± 0.009
204	0.827 ± 0.084	0.896 ± 0.038	0.957 ± 0.070	0.967 ± 0.023	0.987 ± 0.014
205	1.135 ± 0.171	0.858 ± 0.058	1.019 ± 0.129	0.978 ± 0.045	0.884 ± 0.025
206	0.908 ± 0.123	1.033 ± 0.061	1.000 ± 0.101	0.927 ± 0.036	0.926 ± 0.021
207	0.913 ± 0.099	1.007 ± 0.055	0.901 ± 0.080	0.818 ± 0.027	0.756 ± 0.014
208	1.405 ± 0.272	1.384 ± 0.128	1.183 ± 0.187	0.956 ± 0.063	0.907 ± 0.038
209	1.385 ± 0.178	1.205 ± 0.084	1.243 ± 0.132	1.048 ± 0.038	0.904 ± 0.025
210	1.291 ± 0.142	1.284 ± 0.069	1.242 ± 0.101	0.926 ± 0.031	0.841 ± 0.019
211	1.389 ± 0.295	1.329 ± 0.134	1.298 ± 0.196	1.053 ± 0.053	0.889 ± 0.032

212	1.897	±	0.269	1.146	±	0.092	1.456	±	0.183	1.056	±	0.051	0.893	±	0.029
213	1.486	±	0.131	1.309	±	0.069	1.139	±	0.088	1.024	±	0.029	0.931	±	0.017
214	1.400	±	0.191	1.372	±	0.096	1.301	±	0.132	1.004	±	0.045	0.917	±	0.026
215	1.594	±	0.268	1.299	±	0.215	1.103	±	0.159	1.050	±	0.050	0.961	±	0.031
216	1.383	±	0.235	1.296	±	0.122	1.301	±	0.165	1.029	±	0.054	0.903	±	0.033
217	1.473	±	0.158	1.404	±	0.085	1.182	±	0.112	1.090	±	0.037	0.961	±	0.021
218	1.587	±	0.236	1.209	±	0.096	1.287	±	0.184	0.976	±	0.063	0.888	±	0.040
219	1.332	±	0.321	1.351	±	0.225	1.497	±	0.350	1.019	±	0.134	0.947	±	0.097
220	1.419	±	0.179	1.386	±	0.100	1.323	±	0.147	1.080	±	0.053	0.875	±	0.033
221	1.234	±	0.188	1.240	±	0.123	1.194	±	0.170	0.874	±	0.064	0.852	±	0.036
222	1.425	±	0.135	1.262	±	0.077	1.177	±	0.105	1.054	±	0.039	0.895	±	0.021
223	1.318	±	0.610	0.885	±	0.213	1.194	±	0.651	0.893	±	0.089	0.860	±	0.062
224	1.547	±	0.169	1.267	±	0.291	1.339	±	0.155	1.068	±	0.062	0.960	±	0.034
225	1.446	±	0.477	0.722	±	0.071	0.916	±	0.384	1.003	±	0.135	1.019	±	0.120
226	1.256	±	0.196	1.301	±	0.098	1.044	±	0.141	1.062	±	0.045	0.910	±	0.028
227	1.455	±	0.394	1.351	±	0.125	1.029	±	0.288	0.994	±	0.084	0.897	±	0.065
228	1.453	±	0.137	1.251	±	0.068	1.258	±	0.111	1.008	±	0.035	0.891	±	0.023
229	1.660	±	0.348	1.754	±	0.130	1.336	±	0.169	1.073	±	0.049	0.970	±	0.031
230	1.701	±	0.210	1.469	±	0.096	1.433	±	0.153	1.048	±	0.052	0.974	±	0.035
231	1.409	±	0.151	1.311	±	0.076	1.248	±	0.110	1.083	±	0.037	0.902	±	0.024
232	1.598	±	0.119	1.336	±	0.058	1.226	±	0.084	1.093	±	0.028	0.890	±	0.016
233	1.636	±	0.182	1.446	±	0.088	1.377	±	0.127	1.208	±	0.041	1.069	±	0.027
234	1.209	±	0.089	1.205	±	0.045	1.380	±	0.140	1.202	±	0.036	0.954	±	0.022
235	1.255	±	0.118	1.382	±	0.059	1.202	±	0.082	1.029	±	0.026	0.888	±	0.015
236	1.478	±	0.131	1.364	±	0.063	1.343	±	0.089	1.082	±	0.029	0.945	±	0.018
237	1.410	±	0.240	1.389	±	0.129	1.257	±	0.207	1.024	±	0.060	0.879	±	0.036
238	1.587	±	0.206	1.297	±	0.113	1.199	±	0.165	1.087	±	0.052	0.945	±	0.031
239	1.130	±	0.169	1.274	±	0.086	1.191	±	0.122	0.920	±	0.040	0.839	±	0.021
240	1.385	±	0.143	1.243	±	0.080	1.180	±	0.116	0.993	±	0.039	0.892	±	0.022
241	1.005	±	0.376	1.332	±	0.160	1.349	±	0.323	1.031	±	0.114	0.910	±	0.086
242	1.270	±	0.193	1.331	±	0.086	1.444	±	0.163	1.027	±	0.055	0.900	±	0.037
243	1.190	±	0.183	1.438	±	0.091	1.359	±	0.129	1.116	±	0.042	0.951	±	0.026
244	1.550	±	0.300	1.442	±	0.133	1.498	±	0.231	1.105	±	0.073	0.985	±	0.048
245	1.742	±	0.279	1.360	±	0.166	1.186	±	0.218	1.085	±	0.075	0.959	±	0.046
246	1.528	±	0.280	1.377	±	0.165	1.401	±	0.238	1.108	±	0.108	0.935	±	0.065
247	1.600	±	0.187	1.592	±	0.105	1.438	±	0.134	1.178	±	0.041	1.037	±	0.028
248	1.677	±	0.360	1.433	±	0.255	1.379	±	0.368	0.996	±	0.127	1.005	±	0.082
249	1.415	±	0.160	1.333	±	0.086	1.216	±	0.116	0.985	±	0.044	0.943	±	0.027
250	1.463	±	0.154	1.410	±	0.081	1.265	±	0.118	1.039	±	0.043	0.920	±	0.026
251	1.303	±	0.187	0.713	±	0.105	1.216	±	0.227	1.557	±	0.093	1.005	±	0.045
252	1.366	±	0.219	1.489	±	0.193	1.251	±	0.170	1.056	±	0.068	0.954	±	0.038
253	1.891	±	0.297	2.020	±	0.299	1.186	±	0.257	1.445	±	0.084	0.958	±	0.042
254	1.234	±	0.168	1.167	±	0.102	0.974	±	0.140	1.000	±	0.061	0.953	±	0.034
255	1.105	±	0.073	1.110	±	0.044	1.066	±	0.065	1.082	±	0.026	1.000	±	0.015
256	0.938	±	0.066	0.993	±	0.032	1.019	±	0.052	0.955	±	0.019	0.963	±	0.011
257	0.683	±	0.051	0.764	±	0.025	0.816	±	0.045	0.880	±	0.015	0.914	±	0.010
258	0.489	±	0.043	0.538	±	0.018	0.568	±	0.035	0.683	±	0.013	0.761	±	0.008
259	0.253	±	0.013	0.289	±	0.007	0.319	±	0.010	0.449	±	0.005	0.481	±	0.002

Table S5: Transverse cross-correlated cross-relaxation rates η_{xy} (s^{-1})

residue	9.4 T			11.8 T			14.1 T			18.8 T			23.5 T		
145	0.37	±	0.05	0.44	±	0.04	0.45	±	0.07	0.77	±	0.02	0.93	±	0.02
146	0.54	±	0.04	0.58	±	0.03	0.75	±	0.05	0.94	±	0.02	1.18	±	0.02
147	0.61	±	0.04	0.77	±	0.04	0.83	±	0.05	1.11	±	0.02	1.33	±	0.02
148	0.63	±	0.05	0.90	±	0.05	0.93	±	0.07	1.13	±	0.02	1.42	±	0.02
149	0.69	±	0.04	0.81	±	0.04	1.04	±	0.06	1.30	±	0.02	1.58	±	0.02
150	0.75	±	0.05	0.88	±	0.05	0.97	±	0.08	1.30	±	0.03	1.56	±	0.03
151	0.83	±	0.08	0.85	±	0.06	1.01	±	0.11	1.24	±	0.04	1.59	±	0.03
152	0.78	±	0.05	1.01	±	0.04	1.16	±	0.08	1.36	±	0.02	1.74	±	0.03
153	0.83	±	0.05	0.92	±	0.04	1.18	±	0.07	1.34	±	0.02	1.67	±	0.02
154	0.63	±	0.08	0.52	±	0.08	1.16	±	0.06	1.48	±	0.02	1.81	±	0.02
155	0.79	±	0.06	1.22	±	0.06	1.12	±	0.10	1.46	±	0.03	1.75	±	0.03
156	0.98	±	0.06	1.14	±	0.07	1.34	±	0.08	1.58	±	0.03	1.99	±	0.03
157	0.94	±	0.06	1.03	±	0.05	1.23	±	0.08	1.56	±	0.03	1.81	±	0.03
158	0.74	±	0.04	0.96	±	0.04	1.09	±	0.06	1.38	±	0.02	1.69	±	0.02
159	0.58	±	0.05	0.76	±	0.05	0.87	±	0.09	1.11	±	0.03	1.28	±	0.03
160	0.68	±	0.07	0.90	±	0.05	0.99	±	0.09	1.37	±	0.03	1.64	±	0.03
161	0.58	±	0.13	1.11	±	0.10	0.91	±	0.17	1.47	±	0.05	1.60	±	0.04
162	0.78	±	0.06	1.05	±	0.12	1.20	±	0.09	1.47	±	0.03	1.74	±	0.03
163	0.73	±	0.06	0.88	±	0.05	1.11	±	0.10	1.31	±	0.04	1.60	±	0.03
164	0.98	±	0.08	1.07	±	0.06	1.40	±	0.12	1.71	±	0.04	2.16	±	0.03
165	1.16	±	0.08	1.31	±	0.08	1.60	±	0.12	2.17	±	0.05	2.54	±	0.04
167	1.57	±	0.13	1.81	±	0.11	2.30	±	0.20	2.81	±	0.07	3.34	±	0.05
168	1.76	±	0.17	2.00	±	0.14	2.54	±	0.25	3.30	±	0.09	4.05	±	0.07
169	1.86	±	0.19	2.19	±	0.16	2.60	±	0.31	3.79	±	0.12	4.54	±	0.10
171	2.49	±	0.23	2.52	±	0.19	3.81	±	0.41	4.53	±	0.17	5.37	±	0.13
172	2.76	±	0.35	2.69	±	0.26	3.15	±	0.60	4.94	±	0.29	5.82	±	0.22
173	2.83	±	0.33	3.27	±	0.27	4.15	±	0.64	5.00	±	0.26	6.03	±	0.21
174	1.91	±	0.50	2.70	±	0.46	3.71	±	0.95	5.20	±	0.47	5.70	±	0.39
175	2.59	±	0.44	2.22	±	0.26	3.25	±	0.77	3.87	±	0.27	4.81	±	0.20
176	1.95	±	0.91	1.67	±	0.34	3.06	±	0.96	3.76	±	0.29	4.25	±	0.20
177	2.19	±	0.32	2.30	±	0.20	2.95	±	0.46	3.63	±	0.15	4.36	±	0.12
178	2.14	±	0.23	2.03	±	0.17	2.52	±	0.39	3.27	±	0.12	3.95	±	0.09
179	1.57	±	0.21	1.96	±	0.17	1.87	±	0.28	2.98	±	0.10	3.59	±	0.08
180	1.57	±	0.10	2.17	±	0.10	2.25	±	0.17	2.79	±	0.06	3.31	±	0.05
181	1.25	±	0.07	2.93	±	0.11	1.79	±	0.13	2.52	±	0.04	3.04	±	0.03
183	1.17	±	0.16	1.33	±	0.12	1.74	±	0.26	2.16	±	0.08	2.46	±	0.06
184	1.05	±	0.31	1.14	±	0.17	1.38	±	0.43	1.83	±	0.12	2.28	±	0.08
185	0.76	±	0.09	0.93	±	0.07	0.98	±	0.15	1.40	±	0.05	1.72	±	0.04
187	1.18	±	0.17	1.29	±	0.11	1.72	±	0.25	1.97	±	0.07	2.41	±	0.06
188	1.15	±	0.21	1.48	±	0.18	1.82	±	0.29	1.99	±	0.09	2.42	±	0.06
189	0.95	±	0.32	1.50	±	0.23	2.09	±	0.39	2.32	±	0.10	2.75	±	0.08
190	1.36	±	0.12	1.62	±	0.12	1.50	±	0.17	2.22	±	0.06	2.60	±	0.05
192	1.30	±	0.09	1.50	±	0.07	1.71	±	0.11	2.30	±	0.04	2.76	±	0.03
193	1.39	±	0.11	1.40	±	0.09	1.69	±	0.15	2.11	±	0.05	2.66	±	0.04
194	1.31	±	0.13	1.53	±	0.10	1.75	±	0.18	2.41	±	0.05	2.90	±	0.05
195	1.27	±	0.32	1.53	±	0.16	1.58	±	0.48	2.24	±	0.10	2.73	±	0.08
197	1.25	±	0.13	1.52	±	0.09	1.88	±	0.19	2.46	±	0.06	2.86	±	0.04
198	1.31	±	0.12	1.46	±	0.09	1.81	±	0.17	2.35	±	0.06	2.72	±	0.05
199	1.25	±	0.06	1.51	±	0.05	1.76	±	0.09	2.21	±	0.03	2.72	±	0.03
200	1.22	±	0.04	1.73	±	0.07	2.17	±	0.13	2.42	±	0.03	2.90	±	0.03
201	1.34	±	0.08	2.12	±	0.10	1.61	±	0.12	2.19	±	0.04	2.68	±	0.03
202	1.53	±	0.08	2.12	±	0.07	1.97	±	0.10	2.52	±	0.03	3.18	±	0.03
204	1.64	±	0.11	1.94	±	0.08	2.41	±	0.16	3.02	±	0.05	3.45	±	0.04
205	1.82	±	0.20	1.93	±	0.12	2.37	±	0.25	3.02	±	0.08	3.72	±	0.07
206	2.10	±	0.16	2.46	±	0.14	3.01	±	0.24	3.72	±	0.07	4.56	±	0.06
207	2.79	±	0.21	3.27	±	0.17	3.84	±	0.27	4.93	±	0.07	6.01	±	0.06
208	3.98	±	0.48	4.47	±	0.35	5.59	±	0.63	6.78	±	0.16	8.17	±	0.14
209	4.52	±	0.38	5.11	±	0.27	5.86	±	0.44	7.91	±	0.11	9.82	±	0.12
210	4.64	±	0.33	4.95	±	0.23	6.11	±	0.39	7.92	±	0.10	9.88	±	0.10
211	4.58	±	0.70	4.84	±	0.44	6.19	±	0.71	7.75	±	0.16	9.41	±	0.13

212	4.54	±	0.50	3.33	±	0.22	6.24	±	0.56	8.85	±	0.17	9.51	±	0.13
213	4.53	±	0.29	5.20	±	0.22	6.19	±	0.34	8.02	±	0.09	9.96	±	0.09
214	4.86	±	0.49	5.63	±	0.35	6.52	±	0.56	8.77	±	0.15	10.53	±	0.13
215	5.41	±	0.81	5.26	±	0.69	6.65	±	0.69	8.21	±	0.16	10.00	±	0.14
216	4.80	±	0.55	4.84	±	0.38	6.16	±	0.64	8.39	±	0.18	9.80	±	0.16
217	4.69	±	0.39	5.60	±	0.32	7.06	±	0.53	8.53	±	0.13	10.62	±	0.12
218	4.61	±	0.60	4.22	±	0.33	6.37	±	0.74	7.95	±	0.20	9.85	±	0.20
219	5.02	±	0.81	5.40	±	0.76	7.58	±	1.45	8.13	±	0.49	9.75	±	0.50
220	3.89	±	0.41	4.91	±	0.39	5.44	±	0.65	7.60	±	0.23	9.27	±	0.22
221	4.46	±	0.48	4.91	±	0.47	5.85	±	0.80	6.96	±	0.26	8.76	±	0.24
222	4.06	±	0.31	4.75	±	0.28	5.57	±	0.46	7.35	±	0.14	8.73	±	0.13
223	3.53	±	0.90	1.80	±	0.40	5.54	±	0.49	7.49	±	0.33	8.05	±	0.29
224	4.21	±	0.44	4.09	±	0.86	5.17	±	0.67	7.12	±	0.26	8.87	±	0.24
225	3.71	±	0.91	1.03	±	0.14	7.21	±	2.02	5.25	±	0.40	9.26	±	0.71
226	3.11	±	0.33	3.78	±	0.26	4.09	±	0.40	5.40	±	0.12	6.70	±	0.11
227	4.11	±	0.59	4.76	±	0.32	6.00	±	0.78	7.29	±	0.20	9.23	±	0.21
228	4.59	±	0.33	4.81	±	0.22	5.69	±	0.42	7.28	±	0.11	9.13	±	0.11
229	5.68	±	0.78	5.38	±	0.39	5.59	±	0.56	7.56	±	0.14	9.51	±	0.14
230	4.69	±	0.40	5.10	±	0.29	5.98	±	0.51	7.76	±	0.15	9.35	±	0.15
231	4.66	±	0.37	4.96	±	0.24	5.97	±	0.43	7.73	±	0.12	9.82	±	0.12
232	4.81	±	0.28	5.02	±	0.19	6.14	±	0.34	7.60	±	0.09	9.63	±	0.09
233	5.01	±	0.45	5.04	±	0.28	6.40	±	0.48	7.85	±	0.13	9.49	±	0.13
234	2.87	±	0.16	2.75	±	0.12	6.29	±	0.46	7.85	±	0.11	9.72	±	0.11
235	4.49	±	0.27	5.00	±	0.19	5.97	±	0.32	7.61	±	0.08	9.42	±	0.08
236	4.12	±	0.27	4.82	±	0.21	5.90	±	0.33	7.24	±	0.09	9.04	±	0.09
237	4.04	±	0.60	4.47	±	0.43	5.00	±	0.71	6.68	±	0.19	8.29	±	0.17
238	4.70	±	0.42	5.12	±	0.34	6.18	±	0.59	7.76	±	0.16	9.78	±	0.16
239	3.66	±	0.41	4.48	±	0.30	5.37	±	0.51	7.09	±	0.13	8.48	±	0.11
240	3.53	±	0.25	3.98	±	0.22	4.64	±	0.36	6.05	±	0.10	7.48	±	0.10
241	4.21	±	0.67	4.53	±	0.45	5.52	±	1.03	7.13	±	0.33	8.85	±	0.34
242	4.41	±	0.40	4.69	±	0.26	6.07	±	0.54	7.42	±	0.16	9.28	±	0.16
243	4.42	±	0.40	4.66	±	0.28	5.86	±	0.49	7.20	±	0.12	8.89	±	0.11
244	4.18	±	0.50	4.65	±	0.34	5.36	±	0.63	7.35	±	0.20	8.85	±	0.19
245	4.47	±	0.59	5.68	±	0.54	6.90	±	0.94	7.96	±	0.23	9.79	±	0.21
246	4.48	±	0.58	4.97	±	0.49	5.56	±	0.79	7.72	±	0.33	9.66	±	0.30
247	4.88	±	0.40	5.45	±	0.32	6.17	±	0.46	7.83	±	0.12	9.89	±	0.13
248	3.90	±	0.79	4.02	±	0.66	6.09	±	1.16	7.49	±	0.38	9.10	±	0.35
249	3.77	±	0.31	4.84	±	0.27	5.83	±	0.46	7.30	±	0.14	9.24	±	0.14
250	4.21	±	0.35	4.44	±	0.25	5.89	±	0.45	7.56	±	0.14	8.83	±	0.12
251	4.17	±	0.38	1.91	±	0.31	5.68	±	0.79	7.61	±	0.21	8.46	±	0.18
252	3.93	±	0.46	4.70	±	0.68	5.21	±	0.68	6.87	±	0.22	8.54	±	0.19
253	4.27	±	0.78	4.85	±	0.93	6.02	±	0.88	8.34	±	0.37	8.07	±	0.24
254	3.07	±	0.29	3.33	±	0.29	4.04	±	0.46	5.33	±	0.18	6.16	±	0.16
255	2.55	±	0.12	2.79	±	0.11	3.34	±	0.19	4.13	±	0.07	4.99	±	0.06
256	1.85	±	0.09	2.11	±	0.07	2.70	±	0.14	3.17	±	0.04	3.91	±	0.04
257	1.17	±	0.07	1.34	±	0.05	1.62	±	0.11	2.00	±	0.03	2.47	±	0.03
258	0.67	±	0.05	0.88	±	0.04	0.98	±	0.07	1.30	±	0.02	1.59	±	0.02
259	0.32	±	0.02	0.38	±	0.02	0.42	±	0.03	0.60	±	0.01	0.74	±	0.01

Table S6: $^{15}N-\{^{1}H\}$ nuclear Overhauser effects

residue	9.4 T			11.8 T			14.1 T			18.8 T			23.5 T		
145	-2.072	±	0.020	-1.828	±	0.029	-1.401	±	0.014	-0.780	±	0.011	-0.322	±	0.006
146	-1.854	±	0.013	-1.550	±	0.025	-1.195	±	0.013	-0.524	±	0.009	-0.102	±	0.006
147	-1.855	±	0.024	-1.360	±	0.028	-1.029	±	0.014	-0.400	±	0.010	0.016	±	0.006
148	-1.590	±	0.019	-1.172	±	0.024	-0.829	±	0.012	-0.284	±	0.010	0.088	±	0.006
149	-1.330	±	0.021	-0.960	±	0.024	-0.654	±	0.015	-0.126	±	0.010	0.138	±	0.006
150	-1.200	±	0.013	-0.842	±	0.021	-0.587	±	0.014	-0.092	±	0.009	0.176	±	0.007
151	-1.153	±	0.014	-0.827	±	0.020	-0.504	±	0.010	-0.040	±	0.009	0.205	±	0.006
152	-1.034	±	0.011	-0.565	±	0.014	-0.435	±	0.008	0.025	±	0.008	0.289	±	0.006
153	-1.060	±	0.011	-0.714	±	0.016	-0.458	±	0.008	0.012	±	0.008	0.244	±	0.005
154	-1.478	±	0.018	-0.631	±	0.034	-0.471	±	0.008	0.027	±	0.007	0.259	±	0.005
155	-0.986	±	0.013	-0.611	±	0.018	-0.409	±	0.010	0.029	±	0.008	0.302	±	0.006
156	-0.921	±	0.008	-0.665	±	0.026	-0.351	±	0.007	0.090	±	0.007	0.322	±	0.005
157	-1.070	±	0.009	-0.681	±	0.019	-0.408	±	0.010	0.035	±	0.009	0.299	±	0.007
158	-1.225	±	0.014	-0.760	±	0.020	-0.488	±	0.010	-0.017	±	0.008	0.239	±	0.006
159	-1.204	±	0.014	-0.875	±	0.023	-0.535	±	0.011	-0.112	±	0.010	0.177	±	0.007
160	-1.176	±	0.009	-0.711	±	0.016	-0.508	±	0.008	-0.010	±	0.007	0.248	±	0.005
161	-1.126	±	0.007	-0.711	±	0.021	-0.467	±	0.012	-0.070	±	0.011	0.228	±	0.007
162	-1.032	±	0.017	-0.504	±	0.025	-0.422	±	0.010	0.010	±	0.009	0.241	±	0.006
163	-0.926	±	0.012	-0.678	±	0.022	-0.394	±	0.012	0.007	±	0.012	0.256	±	0.009
164	-0.950	±	0.019	-0.617	±	0.022	-0.338	±	0.012	0.055	±	0.010	0.307	±	0.008
165	-0.753	±	0.018	-0.520	±	0.032	-0.291	±	0.020	0.062	±	0.014	0.266	±	0.010
167	-0.515	±	0.020	-0.294	±	0.035	-0.092	±	0.025	0.190	±	0.022	0.386	±	0.016
168	-0.328	±	0.030	-0.224	±	0.039	-0.042	±	0.031	0.250	±	0.023	0.428	±	0.020
169	-0.333	±	0.030	-0.081	±	0.039	0.142	±	0.036	0.315	±	0.028	0.453	±	0.024
171	-0.153	±	0.032	0.076	±	0.039	0.260	±	0.040	0.491	±	0.034	0.526	±	0.028
172	0.038	±	0.034	0.252	±	0.052	0.235	±	0.053	0.548	±	0.048	0.559	±	0.035
173	0.210	±	0.034	0.564	±	0.067	0.246	±	0.056	0.512	±	0.048	0.629	±	0.043
174	-0.104	±	0.020	0.214	±	0.096	0.166	±	0.078	0.506	±	0.069	0.613	±	0.057
175	-0.191	±	0.039	0.051	±	0.050	0.228	±	0.047	0.396	±	0.036	0.486	±	0.032
176	-0.121	±	0.033	-0.086	±	0.045	0.171	±	0.040	0.390	±	0.032	0.568	±	0.025
177	-0.167	±	0.047	-0.026	±	0.043	0.100	±	0.031	0.450	±	0.030	0.523	±	0.021
178	-0.281	±	0.017	0.074	±	0.042	0.118	±	0.028	0.329	±	0.023	0.532	±	0.016
179	-0.313	±	0.018	-0.123	±	0.036	0.085	±	0.019	0.330	±	0.018	0.519	±	0.014
180	-0.351	±	0.012	-0.249	±	0.022	0.074	±	0.017	0.343	±	0.015	0.488	±	0.012
181	-0.817	±	0.025	0.179	±	0.020	-0.056	±	0.014	0.273	±	0.013	0.429	±	0.010
183	-0.658	±	0.019	-0.387	±	0.027	-0.124	±	0.015	0.183	±	0.015	0.411	±	0.010
184	-0.614	±	0.012	-0.432	±	0.028	-0.195	±	0.018	0.128	±	0.017	0.358	±	0.014
185	-0.771	±	0.013	-0.503	±	0.022	-0.268	±	0.012	0.050	±	0.011	0.283	±	0.008
187	-0.496	±	0.019	-0.374	±	0.026	-0.173	±	0.016	0.153	±	0.015	0.348	±	0.011
188	-0.565	±	0.020	-0.302	±	0.026	-0.097	±	0.015	0.209	±	0.016	0.387	±	0.011
189	-0.620	±	0.018	-0.347	±	0.032	-0.043	±	0.020	0.243	±	0.016	0.401	±	0.013
190	-0.534	±	0.016	-0.279	±	0.033	-0.105	±	0.016	0.205	±	0.015	0.380	±	0.012
192	-0.553	±	0.011	-0.430	±	0.021	-0.167	±	0.011	0.162	±	0.011	0.392	±	0.008
193	-0.605	±	0.016	-0.390	±	0.028	-0.125	±	0.015	0.197	±	0.013	0.365	±	0.010
194	-0.623	±	0.014	-0.363	±	0.026	-0.161	±	0.015	0.183	±	0.013	0.430	±	0.010
195	-0.594	±	0.016	-0.391	±	0.023	-0.163	±	0.013	0.165	±	0.014	0.362	±	0.011
197	-0.465	±	0.006	-0.307	±	0.019	-0.090	±	0.012	0.189	±	0.012	0.371	±	0.009
198	-0.456	±	0.008	-0.289	±	0.023	-0.090	±	0.013	0.235	±	0.014	0.400	±	0.012
199	-0.557	±	0.011	-0.347	±	0.018	-0.115	±	0.009	0.196	±	0.009	0.385	±	0.007
200	-0.762	±	0.012	-0.138	±	0.014	-0.071	±	0.009	0.148	±	0.009	0.433	±	0.008
201	-0.483	±	0.014	-0.156	±	0.022	-0.057	±	0.010	0.194	±	0.012	0.363	±	0.009
202	-0.339	±	0.011	-0.216	±	0.017	-0.024	±	0.010	0.235	±	0.011	0.424	±	0.009
204	-0.272	±	0.015	-0.096	±	0.020	0.094	±	0.011	0.282	±	0.011	0.516	±	0.009
205	0.246	±	0.007	-0.028	±	0.024	0.102	±	0.017	0.373	±	0.016	0.494	±	0.013
206	-0.062	±	0.021	0.105	±	0.027	0.246	±	0.017	0.420	±	0.016	0.518	±	0.012
207	0.344	±	0.015	0.448	±	0.029	0.448	±	0.016	0.549	±	0.016	0.600	±	0.014
208	0.631	±	0.017	0.652	±	0.039	0.656	±	0.022	0.876	±	0.029	0.810	±	0.026
209	0.683	±	0.024	0.708	±	0.031	0.722	±	0.017	0.812	±	0.020	0.850	±	0.020
210	0.555	±	0.011	0.690	±	0.031	0.718	±	0.017	0.763	±	0.017	0.876	±	0.017
211	0.661	±	0.029	0.718	±	0.037	0.686	±	0.019	0.801	±	0.024	0.801	±	0.016

212	0.595	±	0.025	0.771	±	0.046	0.693	±	0.022	0.825	±	0.025	0.823	±	0.020
213	0.520	±	0.012	0.651	±	0.030	0.697	±	0.016	0.740	±	0.018	0.793	±	0.015
214	0.601	±	0.024	0.606	±	0.035	0.713	±	0.022	0.790	±	0.023	0.779	±	0.018
215	0.578	±	0.016	0.637	±	0.046	0.713	±	0.021	0.768	±	0.022	0.840	±	0.020
216	0.686	±	0.032	0.659	±	0.045	0.733	±	0.027	0.773	±	0.028	0.815	±	0.026
217	0.551	±	0.014	0.624	±	0.038	0.744	±	0.024	0.860	±	0.025	0.813	±	0.018
218	0.555	±	0.018	0.689	±	0.040	0.704	±	0.025	0.782	±	0.027	0.764	±	0.022
219	0.718	±	0.042	0.615	±	0.065	0.685	±	0.057	0.764	±	0.052	0.757	±	0.047
220	0.585	±	0.028	0.657	±	0.044	0.735	±	0.028	0.766	±	0.031	0.810	±	0.028
221	0.552	±	0.028	0.605	±	0.050	0.695	±	0.037	0.776	±	0.036	0.801	±	0.029
222	0.591	±	0.027	0.589	±	0.031	0.675	±	0.020	0.702	±	0.020	0.797	±	0.018
223	0.431	±	0.038	-0.147	±	0.057	0.692	±	0.031	0.718	±	0.036	0.770	±	0.034
224	0.475	±	0.004	0.734	±	0.145	0.701	±	0.033	0.761	±	0.033	0.828	±	0.037
225	0.393	±	0.040	-0.175	±	0.031	0.603	±	0.031	0.782	±	0.051	0.677	±	0.055
226	0.436	±	0.014	0.641	±	0.035	0.644	±	0.020	0.686	±	0.021	0.780	±	0.019
227	0.551	±	0.013	0.603	±	0.030	0.740	±	0.020	0.729	±	0.022	0.817	±	0.026
228	0.531	±	0.019	0.654	±	0.032	0.677	±	0.018	0.802	±	0.019	0.842	±	0.016
229	0.809	±	0.034	0.737	±	0.037	0.717	±	0.021	0.693	±	0.020	0.810	±	0.017
230	0.602	±	0.032	0.621	±	0.033	0.747	±	0.022	0.805	±	0.026	0.823	±	0.021
231	0.488	±	0.015	0.632	±	0.029	0.709	±	0.019	0.776	±	0.021	0.871	±	0.021
232	0.689	±	0.030	0.634	±	0.027	0.763	±	0.017	0.780	±	0.018	0.893	±	0.015
233	0.647	±	0.015	0.631	±	0.032	0.728	±	0.020	0.836	±	0.023	0.758	±	0.021
234	0.367	±	0.007	0.157	±	0.024	0.764	±	0.021	0.822	±	0.022	0.827	±	0.019
235	0.552	±	0.010	0.665	±	0.027	0.700	±	0.015	0.791	±	0.018	0.806	±	0.013
236	0.585	±	0.013	0.663	±	0.027	0.727	±	0.017	0.809	±	0.019	0.806	±	0.014
237	0.586	±	0.017	0.630	±	0.045	0.668	±	0.028	0.738	±	0.028	0.836	±	0.027
238	0.567	±	0.016	0.627	±	0.039	0.690	±	0.026	0.788	±	0.029	0.834	±	0.025
239	0.491	±	0.018	0.621	±	0.035	0.727	±	0.025	0.755	±	0.024	0.808	±	0.018
240	0.510	±	0.020	0.630	±	0.038	0.609	±	0.023	0.660	±	0.021	0.819	±	0.021
241	0.567	±	0.015	0.574	±	0.047	0.749	±	0.037	0.805	±	0.039	0.808	±	0.039
242	0.550	±	0.022	0.663	±	0.033	0.697	±	0.021	0.738	±	0.021	0.797	±	0.025
243	0.541	±	0.018	0.673	±	0.034	0.738	±	0.022	0.820	±	0.024	0.807	±	0.018
244	0.484	±	0.024	0.647	±	0.045	0.652	±	0.024	0.708	±	0.030	0.733	±	0.025
245	0.701	±	0.031	0.658	±	0.054	0.749	±	0.038	0.827	±	0.036	0.820	±	0.033
246	0.576	±	0.022	0.719	±	0.066	0.680	±	0.046	0.751	±	0.049	0.765	±	0.045
247	0.682	±	0.021	0.643	±	0.031	0.717	±	0.019	0.851	±	0.022	0.884	±	0.020
248	0.724	±	0.068	0.598	±	0.059	0.664	±	0.039	0.803	±	0.042	0.709	±	0.049
249	0.586	±	0.016	0.684	±	0.036	0.762	±	0.024	0.883	±	0.027	0.792	±	0.021
250	0.549	±	0.018	0.654	±	0.033	0.668	±	0.020	0.788	±	0.025	0.745	±	0.018
251	0.596	±	0.041	0.300	±	0.034	0.724	±	0.036	0.800	±	0.034	0.827	±	0.031
252	0.513	±	0.031	0.597	±	0.080	0.613	±	0.033	0.766	±	0.034	0.728	±	0.031
253	0.776	±	0.251	0.570	±	0.100	0.558	±	0.057	0.628	±	0.054	0.726	±	0.036
254	0.310	±	0.029	0.503	±	0.063	0.509	±	0.036	0.507	±	0.033	0.620	±	0.033
255	0.127	±	0.014	0.223	±	0.029	0.337	±	0.019	0.517	±	0.020	0.569	±	0.015
256	-0.134	±	0.013	0.015	±	0.020	0.161	±	0.012	0.360	±	0.014	0.504	±	0.011
257	-0.592	±	0.010	-0.382	±	0.017	-0.125	±	0.009	0.100	±	0.010	0.320	±	0.008
258	-1.118	±	0.007	-0.962	±	0.015	-0.683	±	0.007	-0.250	±	0.007	0.049	±	0.005
259	-2.011	±	0.017	-1.674	±	0.018	-1.330	±	0.009	-0.702	±	0.007	-0.316	±	0.004

11. Spectral density mapping results:

Table S7: $J(0.87\omega_H)$ (ns) derived at five magnetic fields

residue	9.4 T	11.8 T	14.1 T	18.8 T	23.5 T
145	0.0515 ± 0.0005	0.0459 ± 0.0006	0.0372 ± 0.0005	0.0284 ± 0.0002	0.0230 ± 0.0001
146	0.0549 ± 0.0004	0.0485 ± 0.0005	0.0388 ± 0.0005	0.0273 ± 0.0002	0.0221 ± 0.0001
147	0.0605 ± 0.0006	0.0460 ± 0.0006	0.0409 ± 0.0005	0.0275 ± 0.0002	0.0212 ± 0.0001
148	0.0566 ± 0.0006	0.0442 ± 0.0005	0.0397 ± 0.0005	0.0263 ± 0.0002	0.0199 ± 0.0001
149	0.0529 ± 0.0006	0.0420 ± 0.0006	0.0359 ± 0.0005	0.0239 ± 0.0002	0.0193 ± 0.0001
150	0.0525 ± 0.0005	0.0423 ± 0.0005	0.0364 ± 0.0006	0.0244 ± 0.0002	0.0194 ± 0.0002
151	0.0521 ± 0.0005	0.0424 ± 0.0005	0.0334 ± 0.0007	0.0217 ± 0.0002	0.0189 ± 0.0002
152	0.0510 ± 0.0004	0.0383 ± 0.0004	0.0349 ± 0.0005	0.0230 ± 0.0002	0.0180 ± 0.0001
153	0.0529 ± 0.0004	0.0401 ± 0.0004	0.0338 ± 0.0005	0.0221 ± 0.0002	0.0182 ± 0.0001
154	0.0629 ± 0.0006	0.0432 ± 0.0017	0.0354 ± 0.0004	0.0223 ± 0.0002	0.0187 ± 0.0001
155	0.0526 ± 0.0006	0.0385 ± 0.0005	0.0320 ± 0.0005	0.0226 ± 0.0002	0.0173 ± 0.0002
156	0.0525 ± 0.0004	0.0425 ± 0.0008	0.0335 ± 0.0005	0.0226 ± 0.0002	0.0178 ± 0.0001
157	0.0551 ± 0.0004	0.0414 ± 0.0005	0.0353 ± 0.0006	0.0234 ± 0.0002	0.0178 ± 0.0002
158	0.0535 ± 0.0005	0.0409 ± 0.0005	0.0355 ± 0.0004	0.0233 ± 0.0002	0.0182 ± 0.0001
159	0.0527 ± 0.0005	0.0426 ± 0.0006	0.0321 ± 0.0006	0.0246 ± 0.0002	0.0183 ± 0.0002
160	0.0507 ± 0.0004	0.0373 ± 0.0004	0.0331 ± 0.0005	0.0225 ± 0.0002	0.0174 ± 0.0001
161	0.0506 ± 0.0007	0.0394 ± 0.0006	0.0335 ± 0.0009	0.0231 ± 0.0003	0.0182 ± 0.0002
162	0.0498 ± 0.0006	0.0356 ± 0.0007	0.0336 ± 0.0005	0.0228 ± 0.0002	0.0181 ± 0.0002
163	0.0473 ± 0.0005	0.0393 ± 0.0006	0.0308 ± 0.0006	0.0233 ± 0.0003	0.0169 ± 0.0002
164	0.0512 ± 0.0007	0.0393 ± 0.0006	0.0324 ± 0.0007	0.0220 ± 0.0003	0.0168 ± 0.0002
165	0.0449 ± 0.0006	0.0354 ± 0.0008	0.0307 ± 0.0008	0.0211 ± 0.0003	0.0168 ± 0.0002
167	0.0463 ± 0.0008	0.0357 ± 0.0011	0.0278 ± 0.0009	0.0195 ± 0.0005	0.0151 ± 0.0004
168	0.0419 ± 0.0012	0.0361 ± 0.0012	0.0285 ± 0.0012	0.0190 ± 0.0006	0.0140 ± 0.0005
169	0.0425 ± 0.0011	0.0318 ± 0.0013	0.0225 ± 0.0012	0.0167 ± 0.0006	0.0131 ± 0.0006
171	0.0404 ± 0.0012	0.0293 ± 0.0012	0.0206 ± 0.0014	0.0131 ± 0.0009	0.0122 ± 0.0007
172	0.0339 ± 0.0013	0.0245 ± 0.0017	0.0232 ± 0.0020	0.0120 ± 0.0013	0.0113 ± 0.0009
173	0.0260 ± 0.0013	0.0129 ± 0.0020	0.0205 ± 0.0019	0.0138 ± 0.0013	0.0104 ± 0.0012
174	0.0407 ± 0.0011	0.0253 ± 0.0031	0.0258 ± 0.0029	0.0137 ± 0.0019	0.0105 ± 0.0015
175	0.0429 ± 0.0016	0.0293 ± 0.0016	0.0251 ± 0.0021	0.0161 ± 0.0010	0.0132 ± 0.0008
176	0.0417 ± 0.0034	0.0297 ± 0.0016	0.0215 ± 0.0018	0.0157 ± 0.0009	0.0107 ± 0.0006
177	0.0389 ± 0.0017	0.0317 ± 0.0013	0.0257 ± 0.0016	0.0150 ± 0.0009	0.0129 ± 0.0006
178	0.0408 ± 0.0009	0.0284 ± 0.0014	0.0271 ± 0.0013	0.0179 ± 0.0006	0.0127 ± 0.0004
179	0.0421 ± 0.0009	0.0335 ± 0.0012	0.0274 ± 0.0011	0.0173 ± 0.0005	0.0122 ± 0.0004
180	0.0436 ± 0.0006	0.0345 ± 0.0007	0.0262 ± 0.0008	0.0174 ± 0.0004	0.0139 ± 0.0003
181	0.0556 ± 0.0009	0.0283 ± 0.0007	0.0235 ± 0.0005	0.0185 ± 0.0003	0.0143 ± 0.0003
183	0.0474 ± 0.0009	0.0385 ± 0.0009	0.0306 ± 0.0011	0.0192 ± 0.0004	0.0147 ± 0.0003
184	0.0425 ± 0.0010	0.0369 ± 0.0009	0.0280 ± 0.0013	0.0198 ± 0.0004	0.0150 ± 0.0004
185	0.0450 ± 0.0006	0.0358 ± 0.0006	0.0290 ± 0.0008	0.0211 ± 0.0003	0.0165 ± 0.0002
187	0.0413 ± 0.0008	0.0374 ± 0.0008	0.0307 ± 0.0010	0.0200 ± 0.0004	0.0158 ± 0.0003
188	0.0451 ± 0.0009	0.0369 ± 0.0009	0.0289 ± 0.0012	0.0196 ± 0.0004	0.0152 ± 0.0003
189	0.0522 ± 0.0013	0.0390 ± 0.0013	0.0288 ± 0.0013	0.0187 ± 0.0004	0.0154 ± 0.0004
190	0.0463 ± 0.0007	0.0362 ± 0.0010	0.0296 ± 0.0009	0.0202 ± 0.0004	0.0160 ± 0.0003
192	0.0470 ± 0.0005	0.0348 ± 0.0006	0.0320 ± 0.0006	0.0206 ± 0.0003	0.0148 ± 0.0002
193	0.0476 ± 0.0007	0.0362 ± 0.0009	0.0293 ± 0.0008	0.0202 ± 0.0003	0.0159 ± 0.0003
194	0.0484 ± 0.0007	0.0368 ± 0.0008	0.0312 ± 0.0009	0.0198 ± 0.0003	0.0141 ± 0.0002
195	0.0442 ± 0.0010	0.0372 ± 0.0008	0.0284 ± 0.0013	0.0193 ± 0.0003	0.0152 ± 0.0003
197	0.0448 ± 0.0006	0.0346 ± 0.0006	0.0282 ± 0.0008	0.0200 ± 0.0003	0.0157 ± 0.0002
198	0.0437 ± 0.0005	0.0367 ± 0.0007	0.0300 ± 0.0008	0.0185 ± 0.0004	0.0146 ± 0.0003
199	0.0475 ± 0.0005	0.0369 ± 0.0005	0.0288 ± 0.0005	0.0195 ± 0.0002	0.0152 ± 0.0002
200	0.0502 ± 0.0004	0.0286 ± 0.0004	0.0282 ± 0.0005	0.0208 ± 0.0002	0.0141 ± 0.0002
201	0.0447 ± 0.0006	0.0355 ± 0.0007	0.0270 ± 0.0005	0.0190 ± 0.0003	0.0154 ± 0.0002
202	0.0423 ± 0.0005	0.0332 ± 0.0005	0.0263 ± 0.0005	0.0191 ± 0.0003	0.0142 ± 0.0002
204	0.0423 ± 0.0006	0.0328 ± 0.0007	0.0247 ± 0.0006	0.0188 ± 0.0003	0.0122 ± 0.0002
205	0.0247 ± 0.0004	0.0300 ± 0.0008	0.0249 ± 0.0009	0.0160 ± 0.0004	0.0120 ± 0.0003
206	0.0352 ± 0.0008	0.0268 ± 0.0008	0.0199 ± 0.0007	0.0138 ± 0.0004	0.0112 ± 0.0003
207	0.0232 ± 0.0006	0.0161 ± 0.0008	0.0133 ± 0.0005	0.0091 ± 0.0003	0.0074 ± 0.0003
208	0.0166 ± 0.0008	0.0131 ± 0.0015	0.0104 ± 0.0008	0.0030 ± 0.0007	0.0040 ± 0.0005
209	0.0140 ± 0.0011	0.0107 ± 0.0012	0.0088 ± 0.0006	0.0045 ± 0.0005	0.0031 ± 0.0004

210	0.0198	±	0.0005	0.0110	±	0.0011	0.0081	±	0.0006	0.0055	±	0.0004	0.0025	±	0.0003
211	0.0145	±	0.0014	0.0105	±	0.0014	0.0097	±	0.0007	0.0046	±	0.0006	0.0040	±	0.0003
212	0.0182	±	0.0012	0.0080	±	0.0017	0.0095	±	0.0007	0.0040	±	0.0006	0.0037	±	0.0004
213	0.0224	±	0.0006	0.0134	±	0.0011	0.0095	±	0.0006	0.0061	±	0.0004	0.0043	±	0.0003
214	0.0186	±	0.0012	0.0145	±	0.0013	0.0091	±	0.0008	0.0049	±	0.0006	0.0045	±	0.0004
215	0.0194	±	0.0009	0.0131	±	0.0018	0.0087	±	0.0007	0.0056	±	0.0005	0.0034	±	0.0004
216	0.0159	±	0.0025	0.0133	±	0.0018	0.0079	±	0.0009	0.0054	±	0.0007	0.0038	±	0.0005
217	0.0206	±	0.0007	0.0147	±	0.0015	0.0080	±	0.0007	0.0033	±	0.0006	0.0039	±	0.0004
218	0.0209	±	0.0009	0.0107	±	0.0014	0.0094	±	0.0009	0.0050	±	0.0007	0.0046	±	0.0004
219	0.0126	±	0.0019	0.0145	±	0.0025	0.0097	±	0.0019	0.0057	±	0.0012	0.0051	±	0.0010
220	0.0185	±	0.0013	0.0131	±	0.0017	0.0079	±	0.0009	0.0055	±	0.0007	0.0040	±	0.0006
221	0.0188	±	0.0012	0.0132	±	0.0017	0.0089	±	0.0011	0.0048	±	0.0008	0.0040	±	0.0006
222	0.0177	±	0.0012	0.0153	±	0.0012	0.0099	±	0.0007	0.0070	±	0.0005	0.0041	±	0.0004
223	0.0196	±	0.0017	0.0318	±	0.0019	0.0094	±	0.0011	0.0066	±	0.0009	0.0048	±	0.0007
224	0.0245	±	0.0004	0.0107	±	0.0055	0.0092	±	0.0011	0.0060	±	0.0008	0.0037	±	0.0008
225	0.0236	±	0.0017	0.0280	±	0.0008	0.0098	±	0.0011	0.0052	±	0.0012	0.0070	±	0.0012
226	0.0231	±	0.0007	0.0129	±	0.0013	0.0112	±	0.0008	0.0074	±	0.0005	0.0045	±	0.0004
227	0.0204	±	0.0008	0.0159	±	0.0012	0.0075	±	0.0007	0.0066	±	0.0006	0.0041	±	0.0006
228	0.0213	±	0.0009	0.0133	±	0.0012	0.0100	±	0.0006	0.0048	±	0.0005	0.0033	±	0.0003
229	0.0083	±	0.0014	0.0108	±	0.0016	0.0091	±	0.0008	0.0076	±	0.0005	0.0043	±	0.0004
230	0.0190	±	0.0015	0.0152	±	0.0013	0.0075	±	0.0007	0.0050	±	0.0007	0.0039	±	0.0004
231	0.0239	±	0.0008	0.0141	±	0.0011	0.0091	±	0.0007	0.0054	±	0.0005	0.0027	±	0.0004
232	0.0142	±	0.0014	0.0139	±	0.0010	0.0073	±	0.0005	0.0054	±	0.0005	0.0023	±	0.0003
233	0.0173	±	0.0008	0.0152	±	0.0013	0.0088	±	0.0007	0.0043	±	0.0006	0.0055	±	0.0005
234	0.0273	±	0.0005	0.0287	±	0.0008	0.0070	±	0.0007	0.0044	±	0.0006	0.0037	±	0.0004
235	0.0205	±	0.0005	0.0125	±	0.0011	0.0091	±	0.0005	0.0049	±	0.0004	0.0039	±	0.0003
236	0.0190	±	0.0006	0.0124	±	0.0010	0.0088	±	0.0006	0.0046	±	0.0005	0.0040	±	0.0003
237	0.0196	±	0.0009	0.0147	±	0.0018	0.0106	±	0.0010	0.0065	±	0.0007	0.0035	±	0.0005
238	0.0196	±	0.0008	0.0144	±	0.0016	0.0099	±	0.0009	0.0051	±	0.0007	0.0036	±	0.0005
239	0.0211	±	0.0008	0.0134	±	0.0012	0.0073	±	0.0007	0.0057	±	0.0006	0.0037	±	0.0003
240	0.0205	±	0.0009	0.0129	±	0.0013	0.0112	±	0.0008	0.0078	±	0.0005	0.0037	±	0.0004
241	0.0200	±	0.0008	0.0167	±	0.0019	0.0080	±	0.0013	0.0050	±	0.0010	0.0041	±	0.0008
242	0.0210	±	0.0010	0.0128	±	0.0013	0.0101	±	0.0008	0.0067	±	0.0005	0.0045	±	0.0006
243	0.0217	±	0.0010	0.0126	±	0.0013	0.0083	±	0.0008	0.0045	±	0.0006	0.0042	±	0.0004
244	0.0246	±	0.0013	0.0133	±	0.0017	0.0111	±	0.0009	0.0074	±	0.0008	0.0057	±	0.0005
245	0.0148	±	0.0015	0.0137	±	0.0022	0.0074	±	0.0012	0.0043	±	0.0009	0.0038	±	0.0007
246	0.0203	±	0.0011	0.0112	±	0.0025	0.0099	±	0.0015	0.0060	±	0.0012	0.0050	±	0.0010
247	0.0157	±	0.0011	0.0151	±	0.0013	0.0091	±	0.0007	0.0039	±	0.0006	0.0026	±	0.0005
248	0.0126	±	0.0030	0.0162	±	0.0024	0.0099	±	0.0013	0.0048	±	0.0010	0.0061	±	0.0010
249	0.0199	±	0.0009	0.0122	±	0.0013	0.0075	±	0.0008	0.0028	±	0.0007	0.0042	±	0.0005
250	0.0213	±	0.0009	0.0131	±	0.0012	0.0108	±	0.0008	0.0054	±	0.0006	0.0055	±	0.0004
251	0.0191	±	0.0020	0.0218	±	0.0011	0.0101	±	0.0014	0.0052	±	0.0008	0.0039	±	0.0007
252	0.0232	±	0.0015	0.0145	±	0.0030	0.0117	±	0.0011	0.0056	±	0.0008	0.0060	±	0.0007
253	0.0098	±	0.0104	0.0150	±	0.0036	0.0131	±	0.0019	0.0097	±	0.0014	0.0061	±	0.0009
254	0.0288	±	0.0012	0.0177	±	0.0023	0.0160	±	0.0013	0.0121	±	0.0008	0.0087	±	0.0007
255	0.0335	±	0.0007	0.0249	±	0.0010	0.0198	±	0.0007	0.0120	±	0.0005	0.0100	±	0.0003
256	0.0398	±	0.0005	0.0299	±	0.0006	0.0241	±	0.0005	0.0166	±	0.0004	0.0122	±	0.0003
257	0.0466	±	0.0005	0.0367	±	0.0005	0.0281	±	0.0005	0.0213	±	0.0002	0.0159	±	0.0002
258	0.0481	±	0.0003	0.0411	±	0.0004	0.0360	±	0.0005	0.0245	±	0.0002	0.0196	±	0.0001
259	0.0413	±	0.0003	0.0357	±	0.0003	0.0309	±	0.0002	0.0229	±	0.0001	0.0194	±	0.0001

Table S8: Results for the fit of Equation 2 to the experimental spectral density $J(0.87\omega_H)$

residue	λ (ns)			μ (ns.(rad.s⁻¹))		
145	0.1778	±	0.0024	0.0178	±	0.0002
146	0.2007	±	0.0022	0.0160	±	0.0002
147	0.2393	±	0.0031	0.0139	±	0.0002
148	0.2259	±	0.0029	0.0131	±	0.0002
149	0.2063	±	0.0030	0.0128	±	0.0002
150	0.1980	±	0.0026	0.0135	±	0.0002
151	0.2001	±	0.0027	0.0120	±	0.0002
152	0.1937	±	0.0023	0.0122	±	0.0002
153	0.2039	±	0.0021	0.0116	±	0.0002
154	0.2539	±	0.0033	0.0099	±	0.0002
155	0.2058	±	0.0028	0.0110	±	0.0002
156	0.2031	±	0.0023	0.0115	±	0.0002
157	0.2159	±	0.0025	0.0114	±	0.0002
158	0.2102	±	0.0023	0.0117	±	0.0002
159	0.2033	±	0.0028	0.0124	±	0.0002
160	0.1941	±	0.0023	0.0115	±	0.0002
161	0.1935	±	0.0033	0.0122	±	0.0002
162	0.1851	±	0.0031	0.0125	±	0.0002
163	0.1774	±	0.0027	0.0122	±	0.0002
164	0.2051	±	0.0034	0.0106	±	0.0002
165	0.1649	±	0.0033	0.0118	±	0.0003
167	0.1801	±	0.0046	0.0096	±	0.0004
168	0.1711	±	0.0063	0.0094	±	0.0005
169	0.1687	±	0.0067	0.0077	±	0.0006
171	0.1662	±	0.0072	0.0058	±	0.0007
172	0.1322	±	0.0086	0.0067	±	0.0009
173	0.0797	±	0.0092	0.0087	±	0.0011
174	0.1698	±	0.0096	0.0052	±	0.0014
175	0.1686	±	0.0096	0.0075	±	0.0009
176	0.1822	±	0.0134	0.0051	±	0.0009
177	0.1639	±	0.0084	0.0075	±	0.0007
178	0.1590	±	0.0054	0.0082	±	0.0005
179	0.1778	±	0.0051	0.0070	±	0.0004
180	0.1748	±	0.0036	0.0084	±	0.0004
181	0.1976	±	0.0044	0.0075	±	0.0003
183	0.1999	±	0.0046	0.0084	±	0.0003
184	0.1710	±	0.0052	0.0101	±	0.0004
185	0.1679	±	0.0031	0.0116	±	0.0002
187	0.1610	±	0.0042	0.0111	±	0.0003
188	0.1817	±	0.0049	0.0096	±	0.0004
189	0.2167	±	0.0065	0.0079	±	0.0004
190	0.1757	±	0.0040	0.0106	±	0.0003
192	0.1877	±	0.0028	0.0094	±	0.0002
193	0.1848	±	0.0037	0.0101	±	0.0003
194	0.2010	±	0.0036	0.0082	±	0.0003
195	0.1833	±	0.0048	0.0095	±	0.0003
197	0.1699	±	0.0031	0.0105	±	0.0002
198	0.1739	±	0.0032	0.0093	±	0.0003
199	0.1917	±	0.0026	0.0092	±	0.0002
200	0.1841	±	0.0024	0.0091	±	0.0002
201	0.1735	±	0.0034	0.0098	±	0.0003
202	0.1653	±	0.0028	0.0094	±	0.0002
204	0.1763	±	0.0035	0.0076	±	0.0003
205	0.0736	±	0.0027	0.0111	±	0.0003
206	0.1407	±	0.0044	0.0066	±	0.0003
207	0.0900	±	0.0033	0.0045	±	0.0003
208	0.0780	±	0.0052	0.0011	±	0.0005
209	0.0685	±	0.0059	0.0011	±	0.0005
210	0.0930	±	0.0024	0.0000	±	0.0001
211	0.0676	±	0.0066	0.0018	±	0.0004

212	0.0815	±	0.0064	0.0007	±	0.0005
213	0.1015	±	0.0037	0.0007	±	0.0004
214	0.0840	±	0.0061	0.0014	±	0.0004
215	0.0905	±	0.0051	0.0005	±	0.0004
216	0.0748	±	0.0100	0.0014	±	0.0007
217	0.0961	±	0.0037	0.0002	±	0.0002
218	0.0898	±	0.0055	0.0012	±	0.0005
219	0.0509	±	0.0112	0.0036	±	0.0011
220	0.0819	±	0.0074	0.0011	±	0.0006
221	0.0861	±	0.0068	0.0009	±	0.0006
222	0.0850	±	0.0062	0.0018	±	0.0004
223	0.1128	±	0.0091	0.0011	±	0.0007
224	0.1163	±	0.0025	0.0001	±	0.0002
225	0.1575	±	0.0071	0.0005	±	0.0007
226	0.1032	±	0.0044	0.0014	±	0.0004
227	0.0921	±	0.0050	0.0010	±	0.0005
228	0.1010	±	0.0037	0.0001	±	0.0002
229	0.0376	±	0.0072	0.0041	±	0.0005
230	0.0870	±	0.0066	0.0007	±	0.0005
231	0.1100	±	0.0030	0.0000	±	0.0000
232	0.0809	±	0.0046	0.0002	±	0.0003
233	0.0743	±	0.0051	0.0021	±	0.0005
234	0.1360	±	0.0021	0.0000	±	0.0000
235	0.0945	±	0.0031	0.0005	±	0.0003
236	0.0857	±	0.0037	0.0009	±	0.0003
237	0.0920	±	0.0057	0.0010	±	0.0005
238	0.0929	±	0.0050	0.0006	±	0.0005
239	0.0958	±	0.0041	0.0004	±	0.0003
240	0.0933	±	0.0053	0.0016	±	0.0005
241	0.0934	±	0.0052	0.0007	±	0.0006
242	0.0908	±	0.0061	0.0017	±	0.0005
243	0.0964	±	0.0051	0.0005	±	0.0004
244	0.1022	±	0.0069	0.0021	±	0.0006
245	0.0677	±	0.0089	0.0013	±	0.0007
246	0.0875	±	0.0078	0.0017	±	0.0009
247	0.0835	±	0.0048	0.0002	±	0.0003
248	0.0631	±	0.0138	0.0032	±	0.0011
249	0.0897	±	0.0044	0.0003	±	0.0003
250	0.0897	±	0.0052	0.0021	±	0.0004
251	0.1257	±	0.0061	0.0001	±	0.0003
252	0.1014	±	0.0088	0.0017	±	0.0007
253	0.0919	±	0.0253	0.0036	±	0.0014
254	0.1108	±	0.0077	0.0055	±	0.0007
255	0.1367	±	0.0039	0.0055	±	0.0004
256	0.1596	±	0.0032	0.0076	±	0.0003
257	0.1795	±	0.0027	0.0107	±	0.0002
258	0.1730	±	0.0020	0.0146	±	0.0001
259	0.1380	±	0.0014	0.0152	±	0.0001

Table S9: Spectral density function $J(\omega_N)$ (ns) derived from relaxation data at five magnetic fields)

residue	9.4 T			11.8 T			14.1 T			18.8 T			23.5 T		
145	0.1342	±	0.0013	0.1395	±	0.0015	0.1296	±	0.0024	0.1207	±	0.0004	0.1135	±	0.0004
146	0.1638	±	0.0012	0.1738	±	0.0014	0.1556	±	0.0024	0.1409	±	0.0003	0.1359	±	0.0004
147	0.1814	±	0.0015	0.1753	±	0.0017	0.1834	±	0.0025	0.1585	±	0.0004	0.1487	±	0.0004
148	0.1971	±	0.0019	0.1908	±	0.0016	0.2048	±	0.0030	0.1685	±	0.0005	0.1522	±	0.0004
149	0.2168	±	0.0016	0.2104	±	0.0018	0.2085	±	0.0027	0.1770	±	0.0005	0.1583	±	0.0005
150	0.2354	±	0.0019	0.2305	±	0.0020	0.2232	±	0.0038	0.1880	±	0.0006	0.1671	±	0.0005
151	0.2421	±	0.0022	0.2359	±	0.0022	0.2167	±	0.0048	0.1752	±	0.0006	0.1708	±	0.0006
152	0.2563	±	0.0020	0.2533	±	0.0018	0.2428	±	0.0037	0.2027	±	0.0005	0.1828	±	0.0005
153	0.2623	±	0.0017	0.2385	±	0.0017	0.2285	±	0.0036	0.1911	±	0.0005	0.1732	±	0.0005
154	0.2432	±	0.0019	0.2705	±	0.0112	0.2344	±	0.0033	0.1953	±	0.0005	0.1828	±	0.0005
155	0.2743	±	0.0028	0.2453	±	0.0020	0.2234	±	0.0040	0.2006	±	0.0006	0.1795	±	0.0006
156	0.2857	±	0.0019	0.2662	±	0.0028	0.2479	±	0.0039	0.2146	±	0.0005	0.1904	±	0.0006
157	0.2713	±	0.0020	0.2516	±	0.0021	0.2500	±	0.0040	0.2089	±	0.0006	0.1829	±	0.0006
158	0.2366	±	0.0016	0.2344	±	0.0018	0.2350	±	0.0031	0.1953	±	0.0005	0.1716	±	0.0005
159	0.2360	±	0.0021	0.2282	±	0.0022	0.2001	±	0.0041	0.1875	±	0.0007	0.1577	±	0.0006
160	0.2330	±	0.0021	0.2196	±	0.0018	0.2149	±	0.0039	0.1910	±	0.0005	0.1656	±	0.0005
161	0.2388	±	0.0035	0.2346	±	0.0028	0.2251	±	0.0068	0.1828	±	0.0008	0.1683	±	0.0008
162	0.2513	±	0.0024	0.2451	±	0.0027	0.2350	±	0.0041	0.1968	±	0.0006	0.1710	±	0.0006
163	0.2550	±	0.0021	0.2433	±	0.0023	0.2182	±	0.0045	0.2023	±	0.0008	0.1622	±	0.0007
164	0.2721	±	0.0028	0.2519	±	0.0026	0.2428	±	0.0056	0.2016	±	0.0007	0.1757	±	0.0007
165	0.2760	±	0.0029	0.2457	±	0.0035	0.2404	±	0.0059	0.1940	±	0.0009	0.1644	±	0.0007
167	0.3433	±	0.0041	0.3010	±	0.0043	0.2623	±	0.0069	0.2111	±	0.0012	0.1799	±	0.0011
168	0.3611	±	0.0058	0.3295	±	0.0051	0.2850	±	0.0098	0.2247	±	0.0015	0.1805	±	0.0012
169	0.3705	±	0.0057	0.3327	±	0.0052	0.2774	±	0.0106	0.2183	±	0.0017	0.1783	±	0.0014
171	0.4202	±	0.0064	0.3653	±	0.0063	0.2994	±	0.0125	0.2343	±	0.0023	0.1935	±	0.0018
172	0.4338	±	0.0082	0.3860	±	0.0074	0.3310	±	0.0164	0.2420	±	0.0030	0.1935	±	0.0023
173	0.4176	±	0.0106	0.3518	±	0.0085	0.2989	±	0.0176	0.2597	±	0.0037	0.2123	±	0.0030
174	0.4454	±	0.0092	0.3726	±	0.0127	0.3352	±	0.0235	0.2539	±	0.0047	0.2046	±	0.0038
175	0.4287	±	0.0103	0.3505	±	0.0076	0.3515	±	0.0211	0.2408	±	0.0030	0.1907	±	0.0024
176	0.4455	±	0.0371	0.3059	±	0.0105	0.2742	±	0.0220	0.2343	±	0.0036	0.1877	±	0.0026
177	0.3935	±	0.0089	0.3524	±	0.0073	0.3057	±	0.0168	0.2483	±	0.0026	0.2031	±	0.0020
178	0.3724	±	0.0073	0.3478	±	0.0068	0.3311	±	0.0137	0.2417	±	0.0019	0.2023	±	0.0016
179	0.3714	±	0.0066	0.3363	±	0.0056	0.3202	±	0.0125	0.2327	±	0.0016	0.1889	±	0.0014
180	0.3724	±	0.0038	0.3052	±	0.0033	0.2983	±	0.0073	0.2380	±	0.0011	0.2015	±	0.0010
181	0.3429	±	0.0034	0.3923	±	0.0034	0.2254	±	0.0051	0.2278	±	0.0008	0.1856	±	0.0007
183	0.3104	±	0.0051	0.3024	±	0.0043	0.2822	±	0.0103	0.2062	±	0.0012	0.1844	±	0.0011
184	0.2868	±	0.0067	0.2797	±	0.0052	0.2378	±	0.0127	0.1984	±	0.0018	0.1698	±	0.0013
185	0.2723	±	0.0029	0.2516	±	0.0028	0.2297	±	0.0065	0.1915	±	0.0009	0.1662	±	0.0008
187	0.3065	±	0.0045	0.2978	±	0.0042	0.2701	±	0.0093	0.2066	±	0.0013	0.1761	±	0.0011
188	0.3181	±	0.0058	0.3118	±	0.0052	0.2719	±	0.0118	0.2186	±	0.0015	0.1825	±	0.0012
189	0.3572	±	0.0081	0.3144	±	0.0073	0.2864	±	0.0139	0.2185	±	0.0015	0.1892	±	0.0013
190	0.3375	±	0.0042	0.3106	±	0.0050	0.2771	±	0.0084	0.2238	±	0.0011	0.1889	±	0.0010
192	0.3371	±	0.0029	0.2588	±	0.0029	0.2846	±	0.0056	0.2159	±	0.0007	0.1782	±	0.0007
193	0.3288	±	0.0038	0.2799	±	0.0037	0.2679	±	0.0070	0.2210	±	0.0009	0.1833	±	0.0008
194	0.3282	±	0.0041	0.2925	±	0.0040	0.2787	±	0.0078	0.2137	±	0.0010	0.1826	±	0.0009
195	0.3035	±	0.0071	0.2905	±	0.0045	0.2502	±	0.0129	0.2021	±	0.0013	0.1743	±	0.0012
197	0.3461	±	0.0043	0.2882	±	0.0029	0.2671	±	0.0079	0.2165	±	0.0010	0.1821	±	0.0008
198	0.3393	±	0.0041	0.3157	±	0.0035	0.2877	±	0.0080	0.2140	±	0.0011	0.1782	±	0.0010
199	0.3398	±	0.0026	0.2975	±	0.0022	0.2664	±	0.0043	0.2136	±	0.0006	0.1810	±	0.0006
200	0.3137	±	0.0019	0.2693	±	0.0017	0.2731	±	0.0054	0.2149	±	0.0006	0.1827	±	0.0006
201	0.3404	±	0.0032	0.3438	±	0.0030	0.2647	±	0.0055	0.2067	±	0.0008	0.1767	±	0.0007
202	0.3639	±	0.0030	0.3021	±	0.0023	0.2680	±	0.0049	0.2214	±	0.0007	0.1809	±	0.0007
204	0.3880	±	0.0042	0.3366	±	0.0030	0.2873	±	0.0066	0.2351	±	0.0009	0.1871	±	0.0008
205	0.4116	±	0.0053	0.3436	±	0.0044	0.3014	±	0.0105	0.2305	±	0.0014	0.1744	±	0.0010
206	0.4016	±	0.0054	0.3456	±	0.0045	0.2847	±	0.0087	0.2159	±	0.0011	0.1744	±	0.0009
207	0.4555	±	0.0055	0.3522	±	0.0043	0.2679	±	0.0072	0.1863	±	0.0009	0.1406	±	0.0007
208	0.6039	±	0.0119	0.4713	±	0.0077	0.3472	±	0.0139	0.2342	±	0.0018	0.1658	±	0.0014
209	0.5896	±	0.0091	0.4632	±	0.0054	0.3690	±	0.0123	0.2302	±	0.0013	0.1652	±	0.0011
210	0.5912	±	0.0077	0.4429	±	0.0052	0.3295	±	0.0089	0.2218	±	0.0010	0.1556	±	0.0008

211	0.5703	±	0.0120	0.4641	±	0.0070	0.3546	±	0.0142	0.2214	±	0.0015	0.1570	±	0.0011
212	0.5980	±	0.0090	0.4373	±	0.0059	0.3565	±	0.0124	0.2180	±	0.0013	0.1626	±	0.0011
213	0.6162	±	0.0075	0.4743	±	0.0053	0.3595	±	0.0086	0.2232	±	0.0010	0.1614	±	0.0008
214	0.6186	±	0.0106	0.4607	±	0.0062	0.3648	±	0.0133	0.2226	±	0.0015	0.1577	±	0.0011
215	0.6133	±	0.0193	0.4434	±	0.0093	0.3446	±	0.0131	0.2307	±	0.0013	0.1642	±	0.0012
216	0.6814	±	0.0859	0.4894	±	0.0080	0.3379	±	0.0142	0.2283	±	0.0016	0.1620	±	0.0013
217	0.6108	±	0.0095	0.4844	±	0.0061	0.3592	±	0.0122	0.2300	±	0.0012	0.1629	±	0.0010
218	0.6238	±	0.0109	0.4221	±	0.0065	0.3660	±	0.0150	0.2201	±	0.0019	0.1534	±	0.0014
219	0.5996	±	0.0156	0.4744	±	0.0119	0.3531	±	0.0266	0.2263	±	0.0036	0.1675	±	0.0033
220	0.5943	±	0.0100	0.4766	±	0.0073	0.3414	±	0.0136	0.2269	±	0.0018	0.1618	±	0.0015
221	0.5552	±	0.0100	0.4162	±	0.0083	0.3300	±	0.0158	0.2036	±	0.0020	0.1583	±	0.0016
222	0.5756	±	0.0087	0.4609	±	0.0057	0.3466	±	0.0114	0.2207	±	0.0014	0.1576	±	0.0010
223	0.4409	±	0.0285	0.3342	±	0.0109	0.3458	±	0.0181	0.2179	±	0.0026	0.1628	±	0.0021
224	0.6127	±	0.0100	0.4971	±	0.0199	0.3490	±	0.0160	0.2358	±	0.0021	0.1707	±	0.0017
225	0.4863	±	0.0143	0.2752	±	0.0042	0.2715	±	0.0233	0.2263	±	0.0037	0.1676	±	0.0039
226	0.5376	±	0.0089	0.4404	±	0.0068	0.3596	±	0.0136	0.2245	±	0.0015	0.1607	±	0.0012
227	0.6042	±	0.0138	0.4961	±	0.0072	0.3280	±	0.0158	0.2338	±	0.0019	0.1731	±	0.0018
228	0.6003	±	0.0080	0.4772	±	0.0050	0.3553	±	0.0113	0.2304	±	0.0012	0.1666	±	0.0011
229	0.5946	±	0.0112	0.5231	±	0.0077	0.3709	±	0.0138	0.2361	±	0.0016	0.1742	±	0.0013
230	0.6391	±	0.0106	0.5012	±	0.0065	0.3428	±	0.0125	0.2506	±	0.0016	0.1705	±	0.0014
231	0.6153	±	0.0087	0.4742	±	0.0054	0.3601	±	0.0117	0.2292	±	0.0012	0.1641	±	0.0010
232	0.6131	±	0.0072	0.4754	±	0.0045	0.3540	±	0.0089	0.2384	±	0.0010	0.1638	±	0.0008
233	0.6588	±	0.0101	0.5140	±	0.0066	0.3718	±	0.0117	0.2486	±	0.0014	0.1775	±	0.0013
234	0.5565	±	0.0078	0.4134	±	0.0039	0.3369	±	0.0127	0.2375	±	0.0012	0.1687	±	0.0010
235	0.6105	±	0.0072	0.4631	±	0.0043	0.3480	±	0.0085	0.2216	±	0.0009	0.1582	±	0.0008
236	0.6088	±	0.0075	0.4604	±	0.0047	0.3760	±	0.0092	0.2325	±	0.0010	0.1642	±	0.0009
237	0.6303	±	0.0129	0.4934	±	0.0083	0.3673	±	0.0160	0.2355	±	0.0019	0.1654	±	0.0014
238	0.6039	±	0.0104	0.4792	±	0.0067	0.3639	±	0.0148	0.2291	±	0.0016	0.1701	±	0.0013
239	0.5464	±	0.0091	0.4391	±	0.0061	0.3018	±	0.0114	0.2207	±	0.0013	0.1517	±	0.0010
240	0.5534	±	0.0079	0.4284	±	0.0056	0.3274	±	0.0105	0.2165	±	0.0012	0.1610	±	0.0010
241	0.6138	±	0.0142	0.4822	±	0.0096	0.3661	±	0.0234	0.2397	±	0.0031	0.1686	±	0.0024
242	0.6174	±	0.0105	0.4703	±	0.0058	0.3820	±	0.0149	0.2422	±	0.0017	0.1736	±	0.0015
243	0.6284	±	0.0102	0.4814	±	0.0063	0.3637	±	0.0126	0.2392	±	0.0014	0.1713	±	0.0010
244	0.6269	±	0.0136	0.4655	±	0.0081	0.3641	±	0.0163	0.2391	±	0.0022	0.1660	±	0.0016
245	0.6606	±	0.0132	0.5007	±	0.0105	0.3389	±	0.0174	0.2400	±	0.0023	0.1636	±	0.0017
246	0.6374	±	0.0135	0.4878	±	0.0107	0.3586	±	0.0189	0.2288	±	0.0030	0.1652	±	0.0023
247	0.6641	±	0.0097	0.5348	±	0.0065	0.3725	±	0.0116	0.2483	±	0.0013	0.1781	±	0.0011
248	0.6086	±	0.0161	0.5057	±	0.0127	0.3380	±	0.0217	0.2297	±	0.0032	0.1629	±	0.0025
249	0.6433	±	0.0088	0.4783	±	0.0060	0.3601	±	0.0117	0.2262	±	0.0014	0.1580	±	0.0011
250	0.6269	±	0.0094	0.4693	±	0.0054	0.3707	±	0.0112	0.2429	±	0.0015	0.1670	±	0.0012
251	0.6180	±	0.0135	0.3797	±	0.0071	0.4175	±	0.0202	0.2467	±	0.0021	0.1738	±	0.0027
252	0.6270	±	0.0113	0.4458	±	0.0131	0.3437	±	0.0155	0.2273	±	0.0021	0.1693	±	0.0017
253	0.5700	±	0.0123	0.4229	±	0.0220	0.3309	±	0.0236	0.2451	±	0.0034	0.1731	±	0.0034
254	0.5356	±	0.0094	0.4274	±	0.0082	0.3622	±	0.0156	0.2264	±	0.0022	0.1726	±	0.0019
255	0.4783	±	0.0048	0.3791	±	0.0042	0.3271	±	0.0074	0.2270	±	0.0011	0.1758	±	0.0010
256	0.4190	±	0.0040	0.3467	±	0.0030	0.3072	±	0.0059	0.2337	±	0.0008	0.1836	±	0.0008
257	0.3242	±	0.0028	0.2862	±	0.0021	0.2546	±	0.0050	0.2067	±	0.0006	0.1690	±	0.0006
258	0.2259	±	0.0017	0.2073	±	0.0015	0.2063	±	0.0031	0.1610	±	0.0004	0.1429	±	0.0005
259	0.1113	±	0.0007	0.1153	±	0.0007	0.1129	±	0.0011	0.1024	±	0.0002	0.0965	±	0.0002

Table S10: Spectral density function J(0) (ns) derived from relaxation data at five magnetic fields)

residue	9.4T			11.8T			14.1T			18.8T			23.5T		
145	0.177	±	0.054	0.188	±	0.028	0.142	±	0.043	0.228	±	0.013	0.2078	±	0.0087
146	0.250	±	0.035	0.232	±	0.022	0.261	±	0.030	0.235	±	0.008	0.2648	±	0.0060
147	0.289	±	0.040	0.252	±	0.024	0.300	±	0.033	0.283	±	0.009	0.2851	±	0.0058
148	0.309	±	0.051	0.283	±	0.027	0.368	±	0.046	0.297	±	0.011	0.3190	±	0.0072
149	0.332	±	0.042	0.303	±	0.028	0.340	±	0.034	0.345	±	0.012	0.3382	±	0.0068
150	0.383	±	0.063	0.347	±	0.035	0.330	±	0.051	0.341	±	0.016	0.3499	±	0.0095
151	0.440	±	0.108	0.349	±	0.041	0.339	±	0.078	0.320	±	0.021	0.3643	±	0.0125
152	0.301	±	0.049	0.377	±	0.028	0.394	±	0.048	0.339	±	0.012	0.3696	±	0.0074
153	0.404	±	0.057	0.356	±	0.029	0.447	±	0.050	0.316	±	0.011	0.3819	±	0.0081
154	0.187	±	0.058	0.270	±	0.083	0.333	±	0.032	0.345	±	0.011	0.3740	±	0.0074
155	0.366	±	0.067	0.345	±	0.035	0.297	±	0.048	0.401	±	0.017	0.3902	±	0.0096
156	0.458	±	0.066	0.456	±	0.052	0.423	±	0.052	0.400	±	0.013	0.4462	±	0.0097
157	0.427	±	0.059	0.380	±	0.033	0.421	±	0.049	0.399	±	0.015	0.3875	±	0.0088
158	0.319	±	0.038	0.348	±	0.027	0.388	±	0.040	0.340	±	0.010	0.3543	±	0.0071
159	0.315	±	0.069	0.365	±	0.042	0.268	±	0.053	0.378	±	0.022	0.3250	±	0.0124
160	0.358	±	0.094	0.333	±	0.032	0.310	±	0.055	0.355	±	0.015	0.3567	±	0.0094
161	0.191	±	0.132	0.569	±	0.093	0.257	±	0.113	0.402	±	0.033	0.3331	±	0.0172
162	0.349	±	0.055	0.415	±	0.086	0.420	±	0.055	0.410	±	0.017	0.4036	±	0.0103
163	0.345	±	0.060	0.387	±	0.041	0.408	±	0.067	0.432	±	0.0238	0.4043	±	0.0136
164	0.444	±	0.084	0.391	±	0.040	0.531	±	0.082	0.470	±	0.020	0.5026	±	0.0137
165	0.746	±	0.135	0.531	±	0.066	0.549	±	0.075	0.667	±	0.029	0.6251	±	0.0143
167	0.826	±	0.152	0.826	±	0.088	0.905	±	0.128	0.852	±	0.045	0.8308	±	0.0238
168	0.854	±	0.197	0.839	±	0.105	0.953	±	0.171	1.038	±	0.062	0.9383	±	0.0284
169	1.125	±	0.287	0.966	±	0.129	0.917	±	0.197	1.149	±	0.079	1.1486	±	0.0439
171	1.283	±	0.267	1.073	±	0.140	1.365	±	0.251	1.294	±	0.106	1.3194	±	0.0576
172	1.406	±	0.362	1.202	±	0.192	1.194	±	0.365	1.672	±	0.188	1.4913	±	0.0904
173	1.249	±	0.332	1.106	±	0.163	1.318	±	0.356	1.589	±	0.167	1.6010	±	0.1031
174	1.027	±	0.580	1.420	±	0.451	1.810	±	1.354	1.714	±	0.285	1.4358	±	0.1482
175	2.431	±	5.327	1.016	±	0.215	1.616	±	0.721	1.325	±	0.191	1.1822	±	0.0858
176	1.373	±	9.628	0.612	±	0.242	1.090	±	0.839	1.181	±	0.231	1.0089	±	0.1051
177	0.971	±	0.351	0.948	±	0.175	0.965	±	0.299	1.127	±	0.112	1.0547	±	0.0562
178	0.953	±	0.248	0.882	±	0.135	0.986	±	0.263	1.012	±	0.076	1.0253	±	0.0414
179	0.620	±	0.217	0.774	±	0.129	0.707	±	0.213	0.866	±	0.065	0.8805	±	0.0389
180	0.753	±	0.112	0.688	±	0.055	0.716	±	0.096	0.770	±	0.034	0.7774	±	0.0191
181	0.708	±	0.094	1.172	±	0.083	0.388	±	0.049	0.728	±	0.023	0.7060	±	0.0128
183	0.608	±	0.248	0.577	±	0.101	0.593	±	0.188	0.618	±	0.053	0.5825	±	0.0215
184	0.068	±	10.120	0.448	±	0.141	0.348	±	0.895	0.548	±	0.099	0.5533	±	0.0548
185	0.412	±	0.133	0.413	±	0.059	0.424	±	0.128	0.446	±	0.033	0.4354	±	0.0180
187	0.599	±	0.251	0.538	±	0.095	0.613	±	0.181	0.514	±	0.042	0.5571	±	0.0262
188	0.461	±	0.277	0.872	±	0.252	0.760	±	0.350	0.563	±	0.057	0.5401	±	0.0321
189	0.489	±	0.399	0.668	±	0.198	1.002	±	0.710	0.667	±	0.075	0.6592	±	0.0423
190	0.647	±	0.148	0.634	±	0.088	0.547	±	0.116	0.654	±	0.035	0.6491	±	0.0206
192	0.651	±	0.102	0.513	±	0.046	0.599	±	0.072	0.688	±	0.022	0.6251	±	0.0128
193	0.695	±	0.138	0.523	±	0.063	0.564	±	0.092	0.605	±	0.029	0.6184	±	0.0180
194	0.727	±	0.202	0.585	±	0.069	0.594	±	0.117	0.691	±	0.034	0.6940	±	0.0215
195	0.495	±	2.524	0.631	±	0.144	0.355	±	0.267	0.701	±	0.107	0.7008	±	0.0696
197	0.593	±	0.154	0.574	±	0.064	0.617	±	0.128	0.663	±	0.034	0.7219	±	0.0217
198	0.646	±	0.148	0.707	±	0.078	0.738	±	0.142	0.639	±	0.033	0.6369	±	0.0212
199	0.689	±	0.079	0.637	±	0.040	0.620	±	0.056	0.642	±	0.016	0.6547	±	0.0108
200	0.603	±	0.047	0.470	±	0.030	0.761	±	0.082	0.670	±	0.019	0.6697	±	0.0118
201	0.593	±	0.081	0.733	±	0.062	0.534	±	0.072	0.572	±	0.019	0.6284	±	0.0135
202	0.805	±	0.090	0.740	±	0.047	0.704	±	0.061	0.755	±	0.020	0.7905	±	0.0129
204	0.883	±	0.152	0.847	±	0.070	0.881	±	0.115	0.927	±	0.032	0.8410	±	0.0183
205	0.709	±	0.198	0.908	±	0.104	0.843	±	0.186	0.896	±	0.054	0.9731	±	0.0395
206	1.114	±	0.230	0.973	±	0.104	1.083	±	0.176	1.136	±	0.056	1.1579	±	0.0364
207	1.776	±	0.279	1.453	±	0.133	1.523	±	0.207	1.546	±	0.059	1.5727	±	0.0373
208	2.207	±	0.619	1.960	±	0.284	2.299	±	0.576	2.322	±	0.175	2.1196	±	0.1047
209	2.498	±	0.471	2.588	±	0.261	2.355	±	0.340	2.436	±	0.109	2.5720	±	0.0817
210	2.787	±	0.436	2.223	±	0.183	2.187	±	0.256	2.676	±	0.109	2.6234	±	0.0687

211	2.475	±	0.792	2.216	±	0.356	2.352	±	0.520	2.286	±	0.139	2.3763	±	0.0965
212	1.766	±	0.409	1.604	±	0.200	2.067	±	0.380	2.586	±	0.144	2.4808	±	0.0924
213	2.379	±	0.326	2.480	±	0.206	2.677	±	0.294	2.453	±	0.081	2.4709	±	0.0544
214	2.838	±	0.565	2.500	±	0.276	2.503	±	0.386	2.757	±	0.150	2.6062	±	0.0838
215	2.744	±	0.762	2.420	±	0.632	2.925	±	0.601	2.539	±	0.138	2.4450	±	0.0957
216	3.194	±	0.966	2.388	±	0.362	2.162	±	0.427	2.629	±	0.169	2.5083	±	0.1090
217	2.492	±	0.423	2.534	±	0.255	2.971	±	0.399	2.528	±	0.105	2.5773	±	0.0705
218	2.299	±	0.594	1.899	±	0.260	2.532	±	0.561	2.541	±	0.184	2.4391	±	0.1218
219	3.238	±	1.272	2.582	±	0.675	2.635	±	1.044	2.605	±	0.443	2.4647	±	0.3064
220	2.018	±	0.398	2.185	±	0.286	1.901	±	0.372	2.234	±	0.151	2.4564	±	0.1273
221	2.679	±	0.634	2.183	±	0.349	2.254	±	0.527	2.298	±	0.209	2.3261	±	0.1221
222	2.060	±	0.308	2.280	±	0.223	2.221	±	0.342	2.142	±	0.100	2.1907	±	0.0639
223	1.858	±	9.406	0.865	±	0.484	2.155	±	17.533	2.589	±	0.295	2.1817	±	0.1802
224	2.074	±	0.386	2.125	±	0.910	1.790	±	0.374	2.187	±	0.156	2.2338	±	0.1065
225	1.730	±	1.040	0.381	±	0.106	0.816	±	129.096	1.642	±	0.293	2.1876	±	0.3284
226	1.649	±	0.398	1.609	±	0.203	1.882	±	0.407	1.551	±	0.083	1.6575	±	0.0650
227	2.353	±	1.076	2.251	±	0.306	2.946	±	1.250	2.429	±	0.246	2.5573	±	0.1942
228	2.422	±	0.323	2.399	±	0.190	2.165	±	0.290	2.338	±	0.092	2.4355	±	0.0773
229	2.785	±	0.873	2.007	±	0.241	2.077	±	0.407	2.327	±	0.121	2.4386	±	0.0966
230	2.208	±	0.435	2.247	±	0.228	1.932	±	0.304	2.612	±	0.158	2.3311	±	0.0923
231	2.648	±	0.431	2.350	±	0.203	2.338	±	0.302	2.286	±	0.093	2.5572	±	0.0834
232	2.326	±	0.265	2.323	±	0.153	2.392	±	0.243	2.306	±	0.067	2.5336	±	0.0562
233	2.581	±	0.451	2.312	±	0.228	2.331	±	0.320	2.238	±	0.085	2.2331	±	0.0718
234	1.585	±	0.184	1.106	±	0.083	2.048	±	0.314	2.153	±	0.077	2.4511	±	0.0672
235	2.843	±	0.369	2.176	±	0.151	2.331	±	0.234	2.298	±	0.071	2.4017	±	0.0509
236	2.118	±	0.305	2.105	±	0.157	2.202	±	0.225	2.157	±	0.067	2.2379	±	0.0528
237	2.311	±	0.664	2.027	±	0.319	1.979	±	0.524	2.134	±	0.156	2.2235	±	0.1091
238	2.269	±	0.451	2.495	±	0.333	2.606	±	0.526	2.291	±	0.124	2.5122	±	0.0966
239	2.313	±	0.580	1.999	±	0.221	1.844	±	0.290	2.390	±	0.123	2.1860	±	0.0653
240	1.729	±	0.273	1.741	±	0.162	1.709	±	0.265	1.816	±	0.080	1.9087	±	0.0579
241	4.005	±	2.528	2.116	±	0.412	2.114	±	0.813	2.327	±	0.306	2.3437	±	0.2535
242	2.826	±	0.619	2.157	±	0.221	2.174	±	0.378	2.444	±	0.147	2.5522	±	0.1223
243	3.139	±	0.741	1.973	±	0.209	2.125	±	0.311	2.141	±	0.100	2.2739	±	0.0742
244	2.179	±	0.655	1.903	±	0.258	1.719	±	0.430	2.215	±	0.188	2.1120	±	0.1281
245	2.137	±	0.601	2.800	±	0.524	2.825	±	0.712	2.452	±	0.201	2.3851	±	0.1365
246	2.423	±	0.679	2.303	±	0.427	1.930	±	0.526	2.255	±	0.281	2.4582	±	0.2016
247	2.584	±	0.472	2.357	±	0.244	2.146	±	0.300	2.292	±	0.098	2.4138	±	0.0765
248	1.728	±	0.703	1.818	±	0.570	2.246	±	1.129	2.464	±	0.366	2.1150	±	0.2044
249	2.098	±	0.353	2.260	±	0.241	2.346	±	0.358	2.358	±	0.127	2.2028	±	0.0778
250	2.270	±	0.375	1.873	±	0.182	2.313	±	0.324	2.467	±	0.123	2.2753	±	0.0762
251	2.547	±	0.542	1.309	±	0.410	2.733	±	0.764	1.627	±	0.123	2.0680	±	0.1133
252	2.341	±	0.623	1.790	±	0.422	1.958	±	0.475	2.055	±	0.164	2.1438	±	0.1013
253	1.518	±	0.474	1.240	±	0.413	2.398	±	0.805	1.946	±	0.155	2.0637	±	0.1235
254	1.632	±	0.379	1.538	±	0.255	2.006	±	0.434	1.636	±	0.128	1.5464	±	0.0738
255	1.301	±	0.142	1.145	±	0.084	1.300	±	0.143	1.134	±	0.041	1.1837	±	0.0254
256	0.930	±	0.104	0.843	±	0.051	1.001	±	0.092	0.990	±	0.029	0.9821	±	0.0177
257	0.599	±	0.082	0.539	±	0.038	0.576	±	0.067	0.551	±	0.016	0.5578	±	0.0109
258	0.301	±	0.059	0.354	±	0.028	0.385	±	0.054	0.337	±	0.012	0.3410	±	0.0082
259	0.130	±	0.016	0.140	±	0.011	0.138	±	0.017	0.128	±	0.004	0.1491	±	0.0033

12. Two correlation-time analysis of the spectral density function:

Table S11: Parameters of the two correlation-time analysis of the spectral density function in Engrailed

residue	τ_a (ns)			τ_b (ns)			S^2		
145	0.746	+	0.005	0.100	+	0.000	0.378	+	0.003
146	0.819	+	0.004	0.100	+	0.000	0.454	+	0.002
147	0.772	+	0.008	0.100	+	0.000	0.536	+	0.004
148	8.214	+	0.068	0.462	+	0.001	0.187	+	0.002
149	5.266	+	3.300	0.357	+	0.204	0.293	+	0.148
150	6.284	+	0.106	0.512	+	0.004	0.230	+	0.003
151	8.732	+	0.083	0.517	+	0.002	0.206	+	0.002
152	1.189	+	0.008	0.100	+	0.000	0.548	+	0.003
153	1.192	+	0.007	0.100	+	0.000	0.518	+	0.001
154	0.929	+	0.008	0.100	+	0.000	0.615	+	0.003
155	6.057	+	0.050	0.572	+	0.002	0.225	+	0.002
156	1.216	+	0.011	0.100	+	0.000	0.584	+	0.004
157	1.474	+	1.035	0.143	+	0.142	0.533	+	0.107
158	6.841	+	0.082	0.554	+	0.002	0.194	+	0.002
159	8.449	+	0.084	0.528	+	0.002	0.227	+	0.002
160	7.767	+	0.064	0.546	+	0.002	0.238	+	0.001
161	6.817	+	2.804	0.460	+	0.178	0.312	+	0.095
162	1.723	+	1.341	0.140	+	0.132	0.470	+	0.087
163	1.470	+	0.012	0.100	+	0.000	0.466	+	0.002
164	1.308	+	0.008	0.100	+	0.000	0.520	+	0.002
165	1.819	+	0.023	0.100	+	0.000	0.458	+	0.001
167	5.852	+	1.954	0.535	+	0.211	0.415	+	0.049
168	7.397	+	0.166	0.717	+	0.014	0.399	+	0.005
169	7.091	+	0.153	0.700	+	0.010	0.458	+	0.008
171	8.404	+	0.192	0.929	+	0.017	0.450	+	0.008
172	8.541	+	0.293	1.048	+	0.037	0.481	+	0.012
173	11.005	+	0.333	1.298	+	0.037	0.421	+	0.013
174	9.124	+	0.497	1.113	+	0.047	0.422	+	0.013
175	6.664	+	0.344	0.794	+	0.023	0.433	+	0.012
176	8.383	+	0.392	0.855	+	0.019	0.425	+	0.013
177	7.220	+	0.218	0.905	+	0.016	0.404	+	0.008
178	7.485	+	0.148	0.885	+	0.009	0.384	+	0.005
179	6.913	+	1.572	0.711	+	0.203	0.398	+	0.059
180	7.818	+	0.116	0.822	+	0.008	0.319	+	0.003
181	2.603	+	0.704	0.145	+	0.150	0.548	+	0.050
183	4.052	+	2.209	0.386	+	0.274	0.411	+	0.108
184	9.160	+	0.223	0.646	+	0.010	0.397	+	0.005
185	7.628	+	0.084	0.554	+	0.004	0.331	+	0.003
187	6.309	+	2.223	0.528	+	0.212	0.380	+	0.056
188	5.955	+	0.156	0.636	+	0.010	0.351	+	0.006
189	6.356	+	0.163	0.670	+	0.008	0.341	+	0.006
190	4.003	+	2.150	0.408	+	0.288	0.436	+	0.103
192	2.136	+	1.572	0.148	+	0.160	0.510	+	0.048
193	2.392	+	1.234	0.187	+	0.193	0.497	+	0.078
194	8.837	+	0.137	0.718	+	0.004	0.351	+	0.003
195	8.560	+	0.162	0.653	+	0.006	0.389	+	0.006
197	4.901	+	2.478	0.451	+	0.278	0.428	+	0.077
198	7.055	+	0.144	0.652	+	0.009	0.373	+	0.002
199	4.664	+	2.236	0.431	+	0.262	0.399	+	0.092
200	7.964	+	0.058	0.699	+	0.003	0.313	+	0.002
201	4.085	+	1.520	0.374	+	0.213	0.429	+	0.060
202	5.709	+	0.047	0.621	+	0.004	0.377	+	0.002
204	6.283	+	0.094	0.758	+	0.007	0.418	+	0.004
205	5.999	+	2.332	0.604	+	0.400	0.582	+	0.018
206	5.126	+	2.008	0.422	+	0.308	0.533	+	0.048
207	6.724	+	1.201	0.321	+	0.254	0.729	+	0.006
208	7.793	+	0.458	2.411	+	0.369	0.761	+	0.037
209	8.626	+	0.247	2.516	+	0.287	0.722	+	0.018

210	7.682	+	0.127	1.696	+	0.109	0.823	+	0.009
211	7.077	+	0.104	1.391	+	0.080	0.857	+	0.010
212	7.899	+	0.137	1.580	+	0.096	0.793	+	0.009
213	7.056	+	0.070	1.254	+	0.039	0.822	+	0.005
214	7.148	+	0.195	1.411	+	0.143	0.845	+	0.015
215	7.578	+	0.399	1.872	+	0.231	0.789	+	0.024
216	6.720	+	0.170	1.322	+	0.141	0.859	+	0.014
217	7.047	+	0.182	1.562	+	0.165	0.826	+	0.014
218	7.565	+	0.158	1.311	+	0.099	0.824	+	0.011
219	7.756	+	0.528	2.069	+	0.475	0.789	+	0.036
220	7.695	+	0.325	1.827	+	0.273	0.793	+	0.022
221	8.274	+	0.145	1.313	+	0.059	0.781	+	0.010
222	6.768	+	0.051	0.975	+	0.050	0.845	+	0.008
223	11.581	+	0.610	1.360	+	0.038	0.628	+	0.011
224	6.805	+	0.311	1.235	+	0.130	0.783	+	0.019
225	15.150	+	0.521	1.153	+	0.029	0.528	+	0.005
226	6.927	+	0.115	0.974	+	0.056	0.787	+	0.009
227	7.082	+	0.251	1.641	+	0.158	0.800	+	0.021
228	6.926	+	0.137	1.450	+	0.087	0.820	+	0.012
229	6.610	+	0.149	1.397	+	0.112	0.820	+	0.012
230	7.305	+	0.540	2.926	+	0.602	0.738	+	0.058
231	6.843	+	0.121	1.395	+	0.112	0.838	+	0.007
232	8.580	+	0.350	3.340	+	0.259	0.690	+	0.029
233	6.435	+	0.187	2.048	+	0.299	0.811	+	0.021
234	8.009	+	0.165	1.350	+	0.049	0.686	+	0.008
235	7.068	+	0.098	1.255	+	0.054	0.844	+	0.008
236	7.116	+	0.196	1.645	+	0.161	0.814	+	0.014
237	6.410	+	0.217	1.296	+	0.183	0.857	+	0.017
238	7.640	+	0.206	1.794	+	0.118	0.770	+	0.012
239	7.321	+	0.143	1.114	+	0.071	0.832	+	0.008
240	7.604	+	0.158	1.126	+	0.064	0.772	+	0.009
241	6.855	+	0.364	1.717	+	0.271	0.804	+	0.028
242	7.606	+	0.349	2.128	+	0.271	0.732	+	0.026
243	6.727	+	0.158	1.496	+	0.117	0.807	+	0.012
244	6.375	+	0.246	1.042	+	0.160	0.811	+	0.013
245	6.589	+	0.340	2.338	+	0.797	0.861	+	0.037
246	6.717	+	0.194	1.372	+	0.108	0.840	+	0.017
247	6.777	+	0.187	3.942	+	0.149	0.750	+	0.024
248	6.258	+	0.394	1.135	+	0.463	0.878	+	0.017
249	6.424	+	0.073	0.986	+	0.083	0.894	+	0.006
250	6.230	+	0.159	1.016	+	0.124	0.840	+	0.012
251	7.933	+	0.227	1.488	+	0.082	0.662	+	0.014
252	6.623	+	0.184	1.029	+	0.126	0.798	+	0.009
253	6.760	+	0.329	1.131	+	0.135	0.705	+	0.020
254	5.812	+	0.829	0.623	+	0.264	0.716	+	0.019
255	6.814	+	0.086	0.782	+	0.014	0.587	+	0.004
256	6.427	+	0.067	0.754	+	0.009	0.467	+	0.004
257	3.788	+	1.800	0.325	+	0.215	0.421	+	0.062
258	1.339	+	0.018	0.100	+	0.000	0.394	+	0.003
259	1.004	+	0.006	0.100	+	0.000	0.241	+	0.001

13. Three correlation-time analysis of the spectral density function:

Table S12: Parameters of the three correlation-time analysis of the spectral density function in Engrailed

residue	τ_a (ns)			τ_b (ns)			τ_c (ns)			S^2			S^{2f}		
145	6.852	+	3.202	0.730	+	0.053	0.082	+	0.002	0.030	+	0.012	0.440	+	0.007
146	9.009	+	0.504	0.782	+	0.008	0.086	+	0.001	0.030	+	0.002	0.514	+	0.005
147	8.540	+	0.397	0.753	+	0.008	0.084	+	0.002	0.034	+	0.001	0.601	+	0.005
148	8.214	+	0.068	3.423	+	0.869	0.462	+	0.001	0.187	+	0.002	0.187	+	0.002
149	7.644	+	0.049	2.432	+	1.285	0.504	+	0.002	0.186	+	0.002	0.186	+	0.002
150	5.405	+	0.652	1.101	+	0.025	0.092	+	0.001	0.064	+	0.006	0.585	+	0.007
151	6.454	+	1.456	1.704	+	1.218	0.198	+	0.205	0.119	+	0.056	0.494	+	0.184
152	5.427	+	0.449	1.192	+	0.017	0.087	+	0.001	0.064	+	0.005	0.625	+	0.006
153	5.199	+	0.265	1.057	+	0.017	0.080	+	0.001	0.089	+	0.004	0.630	+	0.006
154	7.344	+	0.420	0.862	+	0.006	0.065	+	0.001	0.054	+	0.002	0.727	+	0.003
155	5.686	+	0.398	2.275	+	1.335	0.304	+	0.256	0.148	+	0.074	0.450	+	0.215
156	6.563	+	0.316	1.172	+	0.012	0.096	+	0.001	0.078	+	0.003	0.665	+	0.006
157	4.775	+	0.329	1.112	+	0.017	0.090	+	0.002	0.085	+	0.007	0.662	+	0.007
158	6.681	+	0.675	1.083	+	0.015	0.083	+	0.001	0.049	+	0.005	0.610	+	0.006
159	2.000	+	0.000	0.906	+	0.048	0.072	+	0.003	0.217	+	0.007	0.611	+	0.010
160	7.270	+	0.903	1.116	+	0.017	0.074	+	0.002	0.046	+	0.005	0.583	+	0.007
161	5.617	+	0.737	1.130	+	0.028	0.079	+	0.001	0.057	+	0.006	0.580	+	0.007
162	7.713	+	0.406	1.221	+	0.021	0.086	+	0.002	0.056	+	0.003	0.577	+	0.005
163	3.605	+	1.304	1.060	+	0.264	0.076	+	0.009	0.181	+	0.105	0.589	+	0.027
164	6.496	+	0.313	1.349	+	0.879	0.128	+	0.153	0.124	+	0.046	0.620	+	0.119
165	8.513	+	0.368	1.255	+	0.035	0.083	+	0.002	0.119	+	0.007	0.596	+	0.008
167	6.738	+	0.333	1.198	+	0.046	0.095	+	0.004	0.230	+	0.010	0.707	+	0.011
168	8.037	+	0.638	1.780	+	0.208	0.129	+	0.007	0.205	+	0.027	0.652	+	0.028
169	7.481	+	0.444	1.269	+	0.102	0.096	+	0.007	0.314	+	0.019	0.759	+	0.018
171	7.810	+	0.516	1.529	+	0.495	0.243	+	0.339	0.367	+	0.039	0.760	+	0.158
172	11.506	+	3.208	2.126	+	0.216	0.103	+	0.022	0.254	+	0.060	0.766	+	0.029
173	20.000	+	0.000	1.923	+	0.061	0.034	+	0.008	0.149	+	0.009	0.774	+	0.012
174	9.214	+	0.951	1.605	+	0.243	0.129	+	0.041	0.310	+	0.051	0.843	+	0.045
175	7.421	+	0.593	1.552	+	0.174	0.150	+	0.026	0.324	+	0.031	0.776	+	0.024
176	8.383	+	0.392	2.550	+	0.919	0.855	+	0.019	0.425	+	0.013	0.425	+	0.013
177	8.870	+	0.807	1.693	+	0.079	0.102	+	0.009	0.203	+	0.020	0.749	+	0.016
178	10.247	+	1.471	1.632	+	0.085	0.095	+	0.005	0.175	+	0.025	0.736	+	0.018
179	6.631	+	0.431	1.346	+	0.062	0.069	+	0.004	0.240	+	0.014	0.749	+	0.012
180	7.866	+	0.171	2.176	+	0.517	0.687	+	0.302	0.289	+	0.066	0.396	+	0.171
181	4.418	+	0.133	1.247	+	0.072	0.083	+	0.003	0.298	+	0.012	0.717	+	0.011
183	5.265	+	0.358	1.121	+	0.040	0.070	+	0.004	0.179	+	0.012	0.698	+	0.010
184	4.511	+	1.964	1.033	+	0.381	0.062	+	0.017	0.226	+	0.119	0.631	+	0.030
185	4.959	+	0.629	1.278	+	0.033	0.075	+	0.002	0.117	+	0.009	0.581	+	0.006
187	5.663	+	0.628	1.494	+	0.056	0.084	+	0.004	0.144	+	0.011	0.604	+	0.009
188	3.746	+	0.909	1.176	+	0.290	0.070	+	0.023	0.230	+	0.086	0.675	+	0.047
189	5.280	+	0.659	1.509	+	1.088	0.192	+	0.239	0.259	+	0.040	0.685	+	0.173
190	6.240	+	0.632	1.398	+	0.078	0.101	+	0.004	0.164	+	0.017	0.670	+	0.015
192	5.550	+	0.344	1.171	+	0.026	0.080	+	0.003	0.191	+	0.007	0.690	+	0.008
193	5.798	+	0.327	1.298	+	0.043	0.092	+	0.003	0.171	+	0.012	0.670	+	0.009
194	6.118	+	0.453	1.072	+	0.023	0.072	+	0.003	0.199	+	0.007	0.734	+	0.010
195	7.509	+	1.482	2.524	+	1.196	0.443	+	0.291	0.326	+	0.090	0.503	+	0.155
197	6.636	+	0.290	1.315	+	0.038	0.095	+	0.002	0.188	+	0.009	0.675	+	0.010
198	5.010	+	0.365	1.295	+	0.041	0.075	+	0.003	0.208	+	0.011	0.666	+	0.008
199	5.299	+	0.150	1.116	+	0.016	0.081	+	0.002	0.218	+	0.004	0.709	+	0.004
200	8.628	+	0.310	1.363	+	0.019	0.073	+	0.001	0.120	+	0.005	0.654	+	0.005
201	4.063	+	0.095	1.038	+	0.035	0.077	+	0.003	0.292	+	0.006	0.690	+	0.006
202	6.454	+	0.194	1.404	+	0.051	0.093	+	0.004	0.218	+	0.007	0.682	+	0.009
204	5.681	+	0.277	1.338	+	0.042	0.079	+	0.003	0.271	+	0.012	0.755	+	0.007
205	16.811	+	7.095	2.278	+	0.791	0.063	+	0.002	0.150	+	0.210	0.607	+	0.016
206	6.889	+	0.163	1.474	+	0.074	0.068	+	0.002	0.342	+	0.011	0.741	+	0.011

207	6.852	+	0.122	1.452	+	0.097	0.042	+	0.003	0.537	+	0.010	0.742	+	0.007
208	7.659	+	0.856	3.691	+	0.513	0.023	+	0.012	0.601	+	0.066	0.918	+	0.014
209	8.862	+	0.404	3.052	+	0.361	0.102	+	0.298	0.639	+	0.040	0.964	+	0.014
210	8.047	+	0.315	2.727	+	0.653	0.531	+	0.410	0.769	+	0.031	0.957	+	0.021
211	8.119	+	0.429	3.753	+	0.477	0.043	+	0.013	0.645	+	0.056	0.926	+	0.011
212	7.731	+	0.199	1.684	+	0.154	0.089	+	0.137	0.771	+	0.022	0.978	+	0.017
213	7.364	+	0.288	3.067	+	1.305	0.605	+	0.429	0.772	+	0.037	0.920	+	0.055
214	7.866	+	0.627	3.149	+	0.983	0.322	+	0.162	0.745	+	0.065	0.960	+	0.013
215	8.015	+	0.733	2.590	+	0.584	0.284	+	0.367	0.712	+	0.061	0.962	+	0.019
216	6.725	+	0.174	1.372	+	0.169	0.260	+	0.113	0.856	+	0.018	0.996	+	0.006
217	7.823	+	0.365	4.000	+	0.000	0.801	+	0.132	0.718	+	0.033	0.931	+	0.014
218	7.475	+	0.285	1.853	+	0.290	0.089	+	0.025	0.784	+	0.023	0.956	+	0.020
219	7.898	+	0.844	3.517	+	0.774	0.073	+	0.049	0.652	+	0.080	0.933	+	0.025
220	7.995	+	0.665	2.926	+	0.790	0.054	+	0.030	0.681	+	0.093	0.948	+	0.025
221	7.574	+	0.235	1.605	+	0.145	0.009	+	0.009	0.711	+	0.021	0.922	+	0.020
222	6.878	+	0.403	2.300	+	1.058	0.067	+	0.016	0.757	+	0.073	0.930	+	0.024
223	11.243	+	1.432	1.667	+	0.166	0.007	+	0.010	0.443	+	0.050	0.850	+	0.031
224	7.004	+	0.547	2.226	+	1.389	0.509	+	0.425	0.750	+	0.052	0.946	+	0.071
225	15.556	+	1.096	1.201	+	0.115	0.476	+	0.360	0.481	+	0.105	0.964	+	0.081
226	5.739	+	0.304	1.288	+	0.184	0.018	+	0.004	0.678	+	0.014	0.859	+	0.013
227	7.299	+	0.649	2.246	+	0.700	0.164	+	0.173	0.757	+	0.057	0.975	+	0.018
228	7.021	+	0.329	2.150	+	1.191	0.392	+	0.458	0.795	+	0.034	0.968	+	0.044
229	6.675	+	0.150	2.037	+	0.206	0.131	+	0.023	0.783	+	0.016	0.966	+	0.013
230	7.795	+	0.416	4.000	+	0.000	0.203	+	0.158	0.638	+	0.029	0.977	+	0.008
231	7.267	+	0.292	3.528	+	1.052	0.893	+	0.257	0.776	+	0.034	0.924	+	0.039
232	8.950	+	0.343	4.000	+	0.000	0.075	+	0.244	0.591	+	0.030	0.967	+	0.008
233	6.916	+	0.292	3.819	+	0.450	0.117	+	0.030	0.676	+	0.043	0.963	+	0.009
234	9.747	+	0.491	3.763	+	0.221	0.857	+	0.059	0.548	+	0.027	0.824	+	0.012
235	7.017	+	0.227	1.562	+	0.811	0.122	+	0.292	0.825	+	0.019	0.976	+	0.028
236	7.349	+	0.358	2.928	+	0.533	0.036	+	0.010	0.682	+	0.051	0.934	+	0.014
237	6.883	+	0.603	3.175	+	1.159	0.386	+	0.296	0.757	+	0.064	0.949	+	0.010
238	7.622	+	0.218	1.811	+	0.131	0.132	+	0.161	0.766	+	0.013	0.997	+	0.005
239	8.357	+	1.164	3.061	+	1.255	0.028	+	0.013	0.588	+	0.133	0.886	+	0.033
240	6.742	+	0.232	1.567	+	0.179	0.022	+	0.008	0.672	+	0.020	0.886	+	0.018
241	7.555	+	0.839	3.234	+	0.923	0.365	+	0.287	0.690	+	0.084	0.955	+	0.020
242	9.536	+	0.593	3.980	+	0.066	0.331	+	0.248	0.526	+	0.038	0.949	+	0.011
243	6.932	+	0.301	2.231	+	0.793	0.259	+	0.265	0.767	+	0.038	0.969	+	0.029
244	7.043	+	0.729	3.294	+	0.992	0.303	+	0.162	0.690	+	0.061	0.917	+	0.011
245	6.558	+	0.422	3.866	+	0.346	0.131	+	0.146	0.789	+	0.041	0.962	+	0.018
246	6.801	+	0.441	2.452	+	1.080	0.270	+	0.276	0.789	+	0.041	0.957	+	0.029
247	6.776	+	0.187	3.942	+	0.150	0.366	+	0.293	0.750	+	0.024	1.000	+	0.000
248	6.167	+	0.529	4.000	+	0.000	0.063	+	0.022	0.759	+	0.044	0.912	+	0.024
249	6.283	+	0.128	1.477	+	0.849	0.163	+	0.278	0.874	+	0.012	0.963	+	0.025
250	7.606	+	0.421	4.000	+	0.000	0.137	+	0.022	0.620	+	0.031	0.932	+	0.006
251	8.007	+	0.315	1.731	+	0.188	0.100	+	0.244	0.608	+	0.037	0.960	+	0.023
252	6.579	+	0.200	1.312	+	0.202	0.181	+	0.253	0.777	+	0.016	0.949	+	0.049
253	10.082	+	3.512	3.496	+	0.359	0.076	+	0.023	0.400	+	0.112	0.863	+	0.028
254	5.834	+	0.334	1.623	+	0.267	0.087	+	0.012	0.592	+	0.029	0.827	+	0.019
255	5.623	+	0.148	1.345	+	0.073	0.066	+	0.005	0.463	+	0.008	0.788	+	0.010
256	5.950	+	0.173	1.485	+	0.064	0.095	+	0.004	0.320	+	0.011	0.743	+	0.007
257	4.169	+	0.160	1.135	+	0.036	0.085	+	0.002	0.226	+	0.008	0.642	+	0.006
258	4.055	+	0.213	0.941	+	0.024	0.082	+	0.001	0.118	+	0.006	0.530	+	0.006
259	9.134	+	0.925	0.769	+	0.010	0.057	+	0.000	0.010	+	0.001	0.353	+	0.004

14. IMPACT analysis of the spectral density function:

Table S13: Impact coefficients obtained from the analysis of relaxation rates at five magnetic field

residue	A_1			A_2			A_3		
145	0.0059	±	0.0010	0.0000	±	0.0000	0.1683	±	0.0031
146	0.0093	±	0.0007	0.0000	±	0.0000	0.2191	±	0.0031
147	0.0091	±	0.0007	0.0000	±	0.0000	0.2489	±	0.0031
148	0.0115	±	0.0009	0.0000	±	0.0000	0.3005	±	0.0035
149	0.0118	±	0.0008	0.0000	±	0.0002	0.3615	±	0.0030
150	0.0107	±	0.0013	0.0017	±	0.0026	0.4039	±	0.0041
151	0.0112	±	0.0022	0.0131	±	0.0070	0.3813	±	0.0091
152	0.0100	±	0.0010	0.0000	±	0.0004	0.4732	±	0.0028
153	0.0078	±	0.0017	0.0305	±	0.0063	0.3993	±	0.0077
154	0.0123	±	0.0008	0.0000	±	0.0000	0.4032	±	0.0030
155	0.0108	±	0.0019	0.0129	±	0.0069	0.4485	±	0.0081
156	0.0138	±	0.0017	0.0177	±	0.0054	0.4857	±	0.0063
157	0.0087	±	0.0016	0.0167	±	0.0049	0.4488	±	0.0061
158	0.0103	±	0.0008	0.0000	±	0.0000	0.4194	±	0.0028
159	0.0031	±	0.0019	0.0286	±	0.0050	0.3654	±	0.0067
160	0.0111	±	0.0012	0.0000	±	0.0000	0.4192	±	0.0024
161	0.0074	±	0.0026	0.0086	±	0.0066	0.4036	±	0.0088
162	0.0140	±	0.0018	0.0091	±	0.0053	0.4471	±	0.0068
163	0.0094	±	0.0022	0.0303	±	0.0071	0.4328	±	0.0096
164	0.0196	±	0.0022	0.0353	±	0.0063	0.4253	±	0.0073
165	0.0302	±	0.0024	0.0604	±	0.0068	0.4075	±	0.0078
167	0.0316	±	0.0038	0.1565	±	0.0100	0.3874	±	0.0112
168	0.0334	±	0.0047	0.2008	±	0.0133	0.3942	±	0.0144
169	0.0565	±	0.0060	0.2108	±	0.0129	0.3785	±	0.0143
171	0.0655	±	0.0085	0.2505	±	0.0152	0.4221	±	0.0163
172	0.0772	±	0.0120	0.2827	±	0.0185	0.4348	±	0.0155
173	0.1105	±	0.0150	0.1873	±	0.0213	0.5342	±	0.0155
174	0.0758	±	0.0200	0.2471	±	0.0268	0.4700	±	0.0298
175	0.0449	±	0.0129	0.2683	±	0.0225	0.4069	±	0.0224
176	0.0480	±	0.0163	0.1650	±	0.0391	0.4625	±	0.0341
177	0.0485	±	0.0083	0.1680	±	0.0178	0.5232	±	0.0168
178	0.0528	±	0.0065	0.1369	±	0.0146	0.5344	±	0.0133
179	0.0249	±	0.0059	0.2009	±	0.0150	0.4257	±	0.0161
180	0.0298	±	0.0034	0.1099	±	0.0108	0.5293	±	0.0107
181	0.0057	±	0.0033	0.1942	±	0.0135	0.4206	±	0.0138
183	0.0107	±	0.0041	0.1144	±	0.0127	0.4097	±	0.0136
184	0.0107	±	0.0073	0.1049	±	0.0151	0.3965	±	0.0169
185	0.0063	±	0.0028	0.0648	±	0.0072	0.4082	±	0.0084
187	0.0068	±	0.0037	0.1200	±	0.0103	0.4250	±	0.0115
188	0.0021	±	0.0030	0.1301	±	0.0108	0.4360	±	0.0125
189	0.0083	±	0.0061	0.1654	±	0.0152	0.3913	±	0.0158
190	0.0147	±	0.0035	0.1219	±	0.0104	0.4625	±	0.0114
192	0.0139	±	0.0026	0.1227	±	0.0088	0.4131	±	0.0087
193	0.0154	±	0.0031	0.1055	±	0.0090	0.4507	±	0.0102
194	0.0211	±	0.0035	0.1285	±	0.0096	0.3999	±	0.0103
195	0.0236	±	0.0095	0.1255	±	0.0139	0.3857	±	0.0143
197	0.0224	±	0.0038	0.1328	±	0.0098	0.4335	±	0.0100
198	0.0023	±	0.0026	0.1845	±	0.0086	0.3827	±	0.0095
199	0.0103	±	0.0019	0.1552	±	0.0061	0.3905	±	0.0065
200	0.0302	±	0.0019	0.0670	±	0.0058	0.4721	±	0.0094
201	0.0000	±	0.0003	0.2058	±	0.0060	0.3567	±	0.0068
202	0.0208	±	0.0023	0.1756	±	0.0071	0.4181	±	0.0073
204	0.0133	±	0.0033	0.2306	±	0.0091	0.4116	±	0.0089
205	0.0016	±	0.0027	0.3675	±	0.0104	0.3268	±	0.0086
206	0.0434	±	0.0054	0.2793	±	0.0121	0.3571	±	0.0118
207	0.0589	±	0.0056	0.4684	±	0.0105	0.1542	±	0.0097
208	0.0642	±	0.0144	0.7322	±	0.0164	0.1128	±	0.0099
209	0.1298	±	0.0122	0.6890	±	0.0148	0.1224	±	0.0090
210	0.1448	±	0.0102	0.6560	±	0.0118	0.1154	±	0.0082

211	0.1035	±	0.0134	0.6929	±	0.0174	0.1008	±	0.0104
212	0.1335	±	0.0133	0.6356	±	0.0165	0.1382	±	0.0096
213	0.1107	±	0.0083	0.7140	±	0.0144	0.0810	±	0.0112
214	0.1239	±	0.0124	0.7171	±	0.0169	0.0789	±	0.0128
215	0.1167	±	0.0131	0.6677	±	0.0180	0.1382	±	0.0107
216	0.1048	±	0.0160	0.7627	±	0.0258	0.0726	±	0.0172
217	0.1228	±	0.0101	0.7142	±	0.0164	0.1054	±	0.0126
218	0.1160	±	0.0170	0.6802	±	0.0217	0.0909	±	0.0168
219	0.1058	±	0.0397	0.7056	±	0.0340	0.1104	±	0.0212
220	0.1081	±	0.0156	0.7012	±	0.0186	0.1106	±	0.0122
221	0.1288	±	0.0174	0.5768	±	0.0196	0.1500	±	0.0122
222	0.0804	±	0.0094	0.6904	±	0.0150	0.0990	±	0.0112
223	0.1627	±	0.0274	0.3527	±	0.0313	0.3045	±	0.0179
224	0.0935	±	0.0161	0.6718	±	0.0246	0.1463	±	0.0204
225	0.2743	±	0.0405	0.0390	±	0.0301	0.4917	±	0.0237
226	0.0312	±	0.0089	0.6241	±	0.0160	0.1584	±	0.0127
227	0.1187	±	0.0251	0.7024	±	0.0265	0.1339	±	0.0175
228	0.1137	±	0.0108	0.6821	±	0.0145	0.1337	±	0.0101
229	0.0931	±	0.0140	0.7396	±	0.0264	0.1168	±	0.0166
230	0.0763	±	0.0141	0.7738	±	0.0182	0.1149	±	0.0116
231	0.1286	±	0.0110	0.6984	±	0.0160	0.1111	±	0.0124
232	0.1134	±	0.0078	0.7364	±	0.0125	0.1065	±	0.0078
233	0.0561	±	0.0100	0.8025	±	0.0163	0.1097	±	0.0106
234	0.1576	±	0.0095	0.5037	±	0.0127	0.2476	±	0.0121
235	0.1027	±	0.0073	0.7114	±	0.0126	0.0844	±	0.0099
236	0.0799	±	0.0073	0.7093	±	0.0104	0.1209	±	0.0067
237	0.0653	±	0.0154	0.7745	±	0.0222	0.0810	±	0.0172
238	0.1218	±	0.0135	0.6764	±	0.0186	0.1413	±	0.0125
239	0.0904	±	0.0097	0.6528	±	0.0154	0.1153	±	0.0102
240	0.0657	±	0.0082	0.6036	±	0.0144	0.1589	±	0.0113
241	0.0960	±	0.0320	0.7170	±	0.0263	0.1286	±	0.0180
242	0.1225	±	0.0155	0.6757	±	0.0184	0.1621	±	0.0121
243	0.0828	±	0.0102	0.7186	±	0.0144	0.1368	±	0.0097
244	0.0601	±	0.0165	0.7330	±	0.0227	0.1054	±	0.0185
245	0.0718	±	0.0196	0.8275	±	0.0251	0.0555	±	0.0155
246	0.0939	±	0.0255	0.7613	±	0.0277	0.0743	±	0.0217
247	0.0762	±	0.0097	0.8198	±	0.0168	0.0994	±	0.0100
248	0.0481	±	0.0285	0.7774	±	0.0368	0.0739	±	0.0238
249	0.0628	±	0.0107	0.7885	±	0.0162	0.0528	±	0.0126
250	0.0779	±	0.0105	0.7316	±	0.0156	0.1203	±	0.0110
251	0.0995	±	0.0177	0.5416	±	0.0361	0.2589	±	0.0240
252	0.0761	±	0.0155	0.6886	±	0.0253	0.1238	±	0.0197
253	0.0845	±	0.0183	0.5823	±	0.0322	0.2234	±	0.0235
254	0.0268	±	0.0108	0.5687	±	0.0190	0.1989	±	0.0180
255	0.0130	±	0.0041	0.4331	±	0.0103	0.2775	±	0.0104
256	0.0183	±	0.0030	0.2884	±	0.0084	0.3784	±	0.0087
257	0.0001	±	0.0004	0.1632	±	0.0042	0.3606	±	0.0055
258	0.0019	±	0.0013	0.0623	±	0.0041	0.2810	±	0.0050
259	0.0014	±	0.0004	0.0000	±	0.0000	0.1558	±	0.0020

Table S13: Impact coefficients obtained from the analysis of relaxation rates at five magnetic field (continued)

residue	A_4			A_5			A_6		
145	0.3742	±	0.0107	0.3030	±	0.0161	0.1486	±	0.0091
146	0.4384	±	0.0118	0.1608	±	0.0181	0.1724	±	0.0095
147	0.5096	±	0.0118	0.0310	±	0.0184	0.2013	±	0.0101
148	0.4160	±	0.0124	0.0877	±	0.0185	0.1843	±	0.0100
149	0.2940	±	0.0110	0.1834	±	0.0170	0.1493	±	0.0094
150	0.2469	±	0.0114	0.2503	±	0.0182	0.0865	±	0.0101
151	0.2672	±	0.0146	0.1712	±	0.0220	0.1559	±	0.0130
152	0.1815	±	0.0090	0.2524	±	0.0143	0.0829	±	0.0082
153	0.2642	±	0.0114	0.1559	±	0.0180	0.1423	±	0.0104
154	0.3910	±	0.0077	0.0001	±	0.0010	0.1934	±	0.0057
155	0.2251	±	0.0147	0.1690	±	0.0204	0.1338	±	0.0100
156	0.2061	±	0.0113	0.2072	±	0.0169	0.0695	±	0.0090
157	0.2747	±	0.0117	0.1354	±	0.0183	0.1158	±	0.0101
158	0.2719	±	0.0100	0.1550	±	0.0162	0.1434	±	0.0094
159	0.2810	±	0.0129	0.1784	±	0.0187	0.1434	±	0.0102
160	0.2202	±	0.0089	0.1798	±	0.0143	0.1696	±	0.0082
161	0.2318	±	0.0162	0.2037	±	0.0233	0.1449	±	0.0125
162	0.1573	±	0.0148	0.2930	±	0.0209	0.0794	±	0.0107
163	0.1554	±	0.0146	0.2715	±	0.0231	0.1006	±	0.0134
164	0.2408	±	0.0148	0.1378	±	0.0230	0.1411	±	0.0127
165	0.1324	±	0.0161	0.2824	±	0.0250	0.0873	±	0.0137
167	0.1899	±	0.0229	0.1448	±	0.0385	0.0898	±	0.0223
168	0.1385	±	0.0284	0.1949	±	0.0455	0.0381	±	0.0258
169	0.1370	±	0.0301	0.1260	±	0.0518	0.0911	±	0.0305
171	0.0826	±	0.0332	0.1073	±	0.0600	0.0719	±	0.0368
172	0.0028	±	0.0087	0.1766	±	0.0299	0.0259	±	0.0261
173	0.0000	±	0.0000	0.0007	±	0.0044	0.1673	±	0.0127
174	0.0797	±	0.0378	0.0801	±	0.0633	0.0472	±	0.0408
175	0.1021	±	0.0359	0.1595	±	0.0511	0.0183	±	0.0259
176	0.0971	±	0.0432	0.0602	±	0.0581	0.1672	±	0.0337
177	0.0241	±	0.0262	0.2159	±	0.0387	0.0204	±	0.0222
178	0.0124	±	0.0164	0.2494	±	0.0241	0.0141	±	0.0149
179	0.1503	±	0.0270	0.0729	±	0.0378	0.1253	±	0.0196
180	0.0679	±	0.0177	0.1996	±	0.0296	0.0636	±	0.0173
181	0.1392	±	0.0283	0.1528	±	0.0399	0.0876	±	0.0194
183	0.2382	±	0.0212	0.0484	±	0.0308	0.1787	±	0.0165
184	0.1620	±	0.0288	0.1682	±	0.0422	0.1577	±	0.0223
185	0.1396	±	0.0150	0.2536	±	0.0227	0.1275	±	0.0125
187	0.0967	±	0.0212	0.2876	±	0.0318	0.0639	±	0.0173
188	0.1470	±	0.0226	0.1882	±	0.0327	0.0965	±	0.0172
189	0.2702	±	0.0228	0.0264	±	0.0303	0.1384	±	0.0175
190	0.1150	±	0.0201	0.2636	±	0.0299	0.0224	±	0.0161
192	0.1949	±	0.0140	0.1340	±	0.0209	0.1215	±	0.0114
193	0.1459	±	0.0185	0.2159	±	0.0276	0.0666	±	0.0151
194	0.2536	±	0.0168	0.0264	±	0.0236	0.1705	±	0.0126
195	0.1934	±	0.0242	0.1340	±	0.0350	0.1379	±	0.0202
197	0.1109	±	0.0154	0.2629	±	0.0234	0.0374	±	0.0134
198	0.1694	±	0.0166	0.1488	±	0.0272	0.1124	±	0.0157
199	0.2102	±	0.0120	0.1165	±	0.0183	0.1173	±	0.0100
200	0.1323	±	0.0297	0.1703	±	0.0425	0.1281	±	0.0210
201	0.1694	±	0.0148	0.1803	±	0.0237	0.0877	±	0.0140
202	0.1025	±	0.0128	0.2252	±	0.0213	0.0578	±	0.0123
204	0.1339	±	0.0157	0.1251	±	0.0262	0.0855	±	0.0153
205	0.0000	±	0.0000	0.1990	±	0.0178	0.1051	±	0.0177
206	0.0548	±	0.0206	0.1458	±	0.0311	0.1197	±	0.0178
207	0.0344	±	0.0161	0.0570	±	0.0261	0.2271	±	0.0153
208	0.0001	±	0.0006	0.0003	±	0.0018	0.0905	±	0.0112
209	0.0000	±	0.0000	0.0000	±	0.0003	0.0587	±	0.0088
210	0.0039	±	0.0049	0.0000	±	0.0000	0.0799	±	0.0081
211	0.0000	±	0.0004	0.0060	±	0.0086	0.0968	±	0.0141
212	0.0000	±	0.0003	0.0001	±	0.0008	0.0926	±	0.0092

213	0.0457	±	0.0077	0.0000	±	0.0005	0.0486	±	0.0087
214	0.0161	±	0.0137	0.0203	±	0.0212	0.0437	±	0.0176
215	0.0007	±	0.0027	0.0005	±	0.0024	0.0762	±	0.0103
216	0.0061	±	0.0099	0.0171	±	0.0177	0.0367	±	0.0188
217	0.0111	±	0.0097	0.0002	±	0.0024	0.0463	±	0.0101
218	0.0198	±	0.0133	0.0098	±	0.0162	0.0833	±	0.0201
219	0.0000	±	0.0002	0.0228	±	0.0256	0.0553	±	0.0336
220	0.0016	±	0.0044	0.0084	±	0.0133	0.0701	±	0.0165
221	0.0008	±	0.0029	0.0003	±	0.0021	0.1433	±	0.0131
222	0.0173	±	0.0121	0.0148	±	0.0191	0.0982	±	0.0148
223	0.0005	±	0.0032	0.0001	±	0.0010	0.1795	±	0.0191
224	0.0470	±	0.0144	0.0000	±	0.0000	0.0414	±	0.0163
225	0.0081	±	0.0118	0.0001	±	0.0020	0.1868	±	0.0310
226	0.0204	±	0.0096	0.0007	±	0.0039	0.1652	±	0.0103
227	0.0058	±	0.0093	0.0125	±	0.0143	0.0266	±	0.0208
228	0.0058	±	0.0063	0.0000	±	0.0002	0.0647	±	0.0094
229	0.0000	±	0.0001	0.0323	±	0.0148	0.0182	±	0.0168
230	0.0007	±	0.0029	0.0056	±	0.0092	0.0287	±	0.0123
231	0.0274	±	0.0094	0.0000	±	0.0000	0.0344	±	0.0106
232	0.0000	±	0.0000	0.0000	±	0.0000	0.0437	±	0.0057
233	0.0000	±	0.0005	0.0160	±	0.0128	0.0156	±	0.0129
234	0.0323	±	0.0118	0.0000	±	0.0000	0.0588	±	0.0104
235	0.0237	±	0.0072	0.0000	±	0.0003	0.0778	±	0.0078
236	0.0003	±	0.0013	0.0022	±	0.0047	0.0875	±	0.0070
237	0.0250	±	0.0145	0.0073	±	0.0154	0.0468	±	0.0173
238	0.0049	±	0.0068	0.0021	±	0.0075	0.0535	±	0.0131
239	0.0041	±	0.0060	0.0001	±	0.0009	0.1373	±	0.0085
240	0.0111	±	0.0093	0.0025	±	0.0078	0.1583	±	0.0102
241	0.0072	±	0.0107	0.0057	±	0.0120	0.0455	±	0.0260
242	0.0022	±	0.0056	0.0266	±	0.0141	0.0108	±	0.0133
243	0.0020	±	0.0044	0.0028	±	0.0066	0.0570	±	0.0100
244	0.0365	±	0.0230	0.0421	±	0.0328	0.0229	±	0.0203
245	0.0007	±	0.0033	0.0119	±	0.0149	0.0327	±	0.0192
246	0.0264	±	0.0202	0.0278	±	0.0276	0.0163	±	0.0203
247	0.0002	±	0.0014	0.0001	±	0.0009	0.0043	±	0.0056
248	0.0025	±	0.0084	0.0500	±	0.0309	0.0481	±	0.0328
249	0.0143	±	0.0101	0.0007	±	0.0037	0.0808	±	0.0105
250	0.0040	±	0.0083	0.0560	±	0.0155	0.0103	±	0.0125
251	0.0027	±	0.0059	0.0000	±	0.0004	0.0972	±	0.0141
252	0.0349	±	0.0206	0.0209	±	0.0292	0.0556	±	0.0225
253	0.0082	±	0.0178	0.0706	±	0.0335	0.0310	±	0.0285
254	0.0378	±	0.0283	0.1434	±	0.0434	0.0244	±	0.0250
255	0.0863	±	0.0187	0.0823	±	0.0324	0.1077	±	0.0194
256	0.0961	±	0.0155	0.1639	±	0.0257	0.0550	±	0.0147
257	0.1879	±	0.0114	0.2021	±	0.0189	0.0861	±	0.0107
258	0.2628	±	0.0111	0.2715	±	0.0158	0.1205	±	0.0082
259	0.2799	±	0.0079	0.2184	±	0.0115	0.3444	±	0.0059

15. Reference:

1. Farrow. N. A.. O. W. Zhang. A. Szabo. D. A. Torchia. and L. E. Kay. 1995. Spectral Density-Function Mapping Using N-15 Relaxation Data Exclusively. J. Biomol. NMR 6:153-162.
