# Supplementary material for: Distribution of Pico- and Nanosecond Motions in Disordered Proteins from Nuclear Spin Relaxation
Source: Biophys J. 2015 Sep 1;109(5):988–99. doi: 10.1016/j.bpj.2015.06.069 (PMC4564687; doi:10.1016/j.bpj.2015.06.069)
Supplement: Document S2. Article plus Supporting Material [file mmc2.pdf]

Article

Distribution of Pico- and Nanosecond Motions in Disordered Proteins from Nuclear Spin Relaxation

Shahid N. Khan,[1,2,3] Cyril Charlier,[1,2,3] Rafal Augustyniak,[1,2,3] Nicola Salvi,[4] Victoire Déjean,[1,2,3] Geoffrey Bodenhausen,[1,2,3,4] Olivier Lequin,[1,2,3] Philippe Pelupessy,[1,2,3] and Fabien Ferrage[1,2,3,*]
[1]Département de Chimie, École Normale Supérieure-PSL Research University, Paris, France; [2]Sorbonne Universités, UPMC Univ Paris 06, LBM, Paris, France; [3]Centre National de la Recherche Scientifique, UMR 7203 LBM, Paris, France; and [4]Institut des Sciences et Ingénierie Chimiques, École Polytechnique Fédérale de Lausanne, BCH, Lausanne, Switzerland

ABSTRACT Intrinsically disordered proteins and intrinsically disordered regions (IDRs) are ubiquitous in the eukaryotic proteome. The description and understanding of their conformational properties require the development of new experimental, computational, and theoretical approaches. Here, we use nuclear spin relaxation to investigate the distribution of timescales of motions in an IDR from picoseconds to nanoseconds. Nitrogen-15 relaxation rates have been measured at five magnetic fields, ranging from 9.4 to 23.5 T (400–1000 MHz for protons). This exceptional wealth of data allowed us to map the spectral density function for the motions of backbone NH pairs in the partially disordered transcription factor Engrailed at 11 different frequencies. We introduce an approach called interpretation of motions by a projection onto an array of correlation times (IMPACT), which focuses on an array of six correlation times with intervals that are equidistant on a logarithmic scale between 21 ps and 21 ns. The distribution of motions in Engrailed varies smoothly along the protein sequence and is multimodal for most residues, with a prevalence of motions around 1 ns in the IDR. We show that IMPACT often provides better quantitative agreement with experimental data than conventional model-free or extended model-free analyses with two or three correlation times. We introduce a graphical representation that offers a convenient platform for a qualitative discussion of dynamics. Even when relaxation data are only acquired at three magnetic fields that are readily accessible, the IMPACT analysis gives a satisfactory characterization of spectral density functions, thus opening the way to a broad use of this approach.

INTRODUCTION

Intrinsically disordered proteins (IDPs) and regions (IDRs) lack a stable three-dimensional structure organized around a hydrophobic core (1). Such proteins nevertheless play crucial roles in many cellular processes (2). The discovery of IDPs and IDRs is a challenge for the structure-function paradigm (3) and has opened the way to new biophysical contributions to modern proteomics (4). The characterization of the conformational space of IDPs and IDRs can provide insight into the ensemble representation of their three-dimensional organization (5–8). A detailed and quantitative description of the time dependence of the exploration of the conformational space of IDPs and IDRs is required to predict (9) and understand the molecular mechanisms underlying their biological function at the atomic scale.

NMR spectroscopy is a powerful tool for probing molecular motions at atomic resolution on a broad range of timescales in both ordered and disordered proteins (6,10,11). In particular, nuclear spin relaxation can be used to probe a diversity of motions from fast (picoseconds to nanoseconds) reorientation to slow (microseconds to milliseconds) chemical exchange (11,12). Pico- and nanosecond motions of protein backbones are most often characterized by analyzing nitrogen-15 relaxation rates, primarily the longitudinal, R_1, and transverse, R_2, relaxation rates, usually supplemented by ^{15}N-{^{1}H} nuclear Overhauser effects (NOEs). The most general level of analysis provides a map of the spectral density for reorientational motions of the internuclear ^{15}N-^{1}H vectors of the protein backbone (13). In folded proteins, a further step consists in the deconvolution of overall motion (rotational diffusion) and internal dynamics, which is possible when these two types of motions are statistically independent (14,15). The most popular framework for such an analysis is the model-free approach (14), for which the motions of each NH vector are described by a correlation time for overall motion and a correlation time and an order parameter for local motions. The so-called extended model-free approach

Submitted January 8, 2015, and accepted for publication June 23, 2015.

*Correspondence: fabien.ferrage@ens.fr

This is an open access article under the CC BY license (http://creativecommons.org/licenses/by/4.0/).

Shahid N. Khan and Cyril Charlier contributed equally to this work.

Rafal Augustyniak's present address is Departments of Biochemistry, Chemistry and Molecular Genetics, University of Toronto, Toronto, Ontario M5S 1A8, Canada.

Nicola Salvi's present address is Institut de Biologie Structurale Jean-Pierre Ebel, CNRS-CEA-UJF UMR 5075, 41 rue Jules Horowitz, 38027 Grenoble Cedex, France.

Editor: Nathan Baker

© 2015 The Authors

0006-3495/15/09/0988/12

http://dx.doi.org/10.1016/j.bpj.2015.06.069

was later introduced to account for internal motions characterized by two correlation times (16).

The structural flexibility of IDPs and IDRs on nanosecond timescales casts serious doubt on the separation of overall and internal motions. The very notion of a single overall motion can be challenged for IDPs. At best, an overall diffusion tensor would correspond to an average over a set of time-dependent diffusion tensors. In addition, local reorientations of bond vectors due to conformational changes that may occur on timescales similar to the instantaneous overall diffusion would make the statistical independence of internal and overall motions less plausible. New methods therefore have to be developed to describe and rationalize the dynamic properties of IDPs and IDRs (17–19).

Several approaches have been developed in the last 15 years to extract quantitative information about pico- and nanosecond dynamics in IDPs and IDRs from nuclear spin relaxation rates. Often based on spectral density mapping (20,21), most of these approaches rely on the model-free formalism, with residue-specific correlation times (22,23) (i.e., without an overall diffusion tensor), a distribution of picosecond and nanosecond correlation times (24,25), or a statistical analysis of extended model-free parameters (26). An analysis based on a distribution of correlation times necessarily introduces a physical bias, since one must choose a mathematical function to describe the distribution. In the case of the model-independent correlation (MIC) time distribution (26), the statistical independence of three types of motions is not required, since the extended model-free results are considered as a simplified representation of a continuous distribution of correlation times. However, the significance of such a statistical treatment is necessarily limited, since it provides little information about the number of modes of the actual distribution of correlation times. Neither approach seems suited to describe a distribution of correlation times that is a priori unknown. However, simply increasing the number of correlation times or distributions in either approach would be questionable, since the empirical information available from nuclear spin relaxation is limited.

Here, we introduce an approach we call interpretation of motions by a projection onto an array of correlation times (IMPACT) to analyze multiple-field relaxation data in disordered proteins. This method relies as little as possible on any particular physical model of protein motions but constitutes a mathematical reconstruction of the distribution of correlation times. We define an array of n correlation times, τ_i (or, equivalently, of reciprocal frequencies, $\omega_i = 1/\tau_i$) in a range that is effectively sampled by nitrogen-15 relaxation. The experimental spectral density function is then reproduced by a sum of n Lorentzian functions, $J_i(\omega)$, one for each correlation time τ_i. The result of this process, similar to a projection onto a basis of Lorentzian functions, is a discrete distribution of correlation times spanning a range that is relevant to rationalize relaxation. This approach is analogous to the discretization step encountered in regularization methods (27,28), but the volume of experimental data exploited in this study is too limited to use a full regularization approach. Nevertheless, the multimodal character of the distribution of correlation times can be nicely revealed, and the most relevant correlation times for backbone motions can clearly be identified.

IMPACT was originally conceived for a set of relaxation rates obtained at five magnetic fields ranging from 9.4 to 23.5 T (i.e., with proton Larmor frequencies of 400, 500, 600, 800, and 1000 MHz) and later applied to a more limited set recorded at 500, 600, and 800 MHz. Relaxation rates were recorded for a uniformly nitrogen-15-labeled sample of the protein Engrailed 2. Engrailed 2 is a transcription factor that possesses a well-folded DNA-binding homeodomain and a long, 200-residue, mostly disordered N-terminal region. The disordered region plays a crucial role in the regulation of the activity of the protein and, in particular, in binding to transcriptional regulators (29,30). We decided to study an Engrailed 2 fragment (residues 146–259) encompassing the folded homeodomain (residues 200–259) and an N-terminal 54-residue disordered region (residues 146–199) (31). The results of IMPACT show that motions with correlation times close to 1 ns dominate reorientational dynamics in the most disordered regions of the protein, which is believed to be a general property of IDPs and IDRs (32). Yet, the broad variability of correlation times of backbone motions throughout the disordered region of Engrailed stands in stark contrast with the homogeneous dynamic properties of the folded homeodomain. This study reveals a surprising richness of backbone dynamics in IDPs and IDRs on pico- and nanosecond timescales, not found in folded proteins that have been widely studied over the past three decades.

MATERIALS AND METHODS

Sample

All experiments were performed on a sample of uniformly nitrogen-15-labeled chicken Engrailed 2 (residues 146–259) at a concentration of 0.6 mM in 40 mM sodium succinate buffer at pH 6 supplemented with 1 μg/mL of each of the three protease inhibitors leupeptin, pepstatin, and AEBSF, as well as 10 μM EDTA, which allow one to increase the lifetime of the protein (33). The protein was prepared as described elsewhere (31). Note that the protein construct comprises the residues Gly-Pro-Met at the N-terminus before residue Glu^{146}, which remain after cleavage of the GST-tag by PreScission protease (GE Healthcare, Little Chalfont, UK). All experiments were carried out at 303 K, which was adjusted in each spectrometer to have a chemical shift difference of 1.462 ppm between the signals of the methyl and hydroxyl protons of pure methanol (4% protonated and 96% deuterated).

NMR spectroscopy

The relaxation rates were measured at five different static fields of 9.4, 11.7, 14.1, 18.8, and 23.5 T, with corresponding proton Larmor frequencies of 400, 500, 600, 800, and 1000 MHz. Three aliquots of the same sample

were used for all experiments, except at 11.7 T, which was performed on a separate, but identical, sample.

At each field, a full set of ^{15}N relaxation measurements was obtained. The longitudinal relaxation rates, $R_1(^{15}N)$, were obtained in the traditional way (34–36), with saturation of the water signal for each scan, whereas the transverse relaxation rates, $R_2(^{15}N)$, were recorded with a train of ^{15}N π-pulses (Carr-Purcell-Meiboom-Gill pulse train), interleaved with ^{1}H π-pulses to suppress cross-correlated relaxation effects. ^{15}N-{^{1}H} NOEs were obtained by detecting the ^{15}N steady-state polarization while saturating the protons with a train of π-pulses, with suitable interpulse delays and *rf* amplitudes (37,38). Finally, experiments to measure the transverse and longitudinal cross-relaxation rates due to correlated fluctuations of the nitrogen-15 chemical shift anisotropy (CSA) and the dipolar coupling between the ^{15}N nucleus and the amide proton were recorded using the so-called symmetrical reconversion principle (39,40). All experiments were recorded on Bruker Avance spectrometers (Billerica, MA). Experiments at 500 MHz, 800 MHz, and 1 GHz, and the NOE at 600 MHz, have been recorded using triple-resonance indirect-detection cryogenic probes (41) equipped with *z*-axis pulsed-field gradients. Other experiments at 600 MHz were recorded on an indirect-detection triple-resonance probe with triple-axis gradients with detection coils at room temperature. Experiments at 400 MHz were recorded on a liquid-nitrogen-cooled cryogenic probe (Prodigy BBO, Bruker) equipped with a *z*-axis gradient.

Spectral density analysis

The full analysis was carried out at 11 points obtained with the reduced spectral mapping, $J(0.87\omega_H)$ and $J(\omega_N)$ at five fields and $J(\omega = 0)$ calculated from relaxation rates measured at 23.5 T. Analyses with two sets of three magnetic fields used seven points on the spectral density function; $J(\omega = 0)$ was derived from the relaxation rates measured at the highest magnetic field, i.e., 18.8 T or 23.5 T. A Monte Carlo simulation with 510 steps was performed to evaluate the error of each parameter, A_i. All simulations were carried out with Mathematica (42).

Supporting Material

The Supporting Material includes tables of all relaxation rates used in the analysis and tables of all parameters resulting from conventional analysis with two and three correlation times as well as from our IMPACT analysis; equations relevant for reduced spectral density mapping; a plot of transverse relaxation rates, R_2, measured at 18.8 T; a comparison of Akaike's Information Criteria (AIC) for IMPACT and conventional analyses with two or three correlation times; one-dimensional plots of AIC for five- and six-correlation-time analyses; a correlation of consecutive IMPACT coefficients; plots of IMPACT coefficients and the IMPACT barcode representation of the analysis of relaxation rates based on data recorded at a set of three fields, which cover a broad range (9.4, 14.1, and 23.5 T) and at a set of three more widely accessible fields (11.7, 14.1, and 18.8 T); and IMPACT coefficients for an analysis with five correlation times and $\tau_{max} = 38$ ns.

RESULTS AND DISCUSSION

Secondary structure

Fig. 1 *e* displays the secondary structure propensity (SSP) (43) based on the assignment of the protein (31). The three α-helices of the homeodomain (residues 200–259) are well identified by SSP scores close to 1. Another region, including the so-called hexapeptide (residues 169–174, WPAWVY) and surrounding residues, displays SSP scores close to 0.3, thus highlighting the presence of some

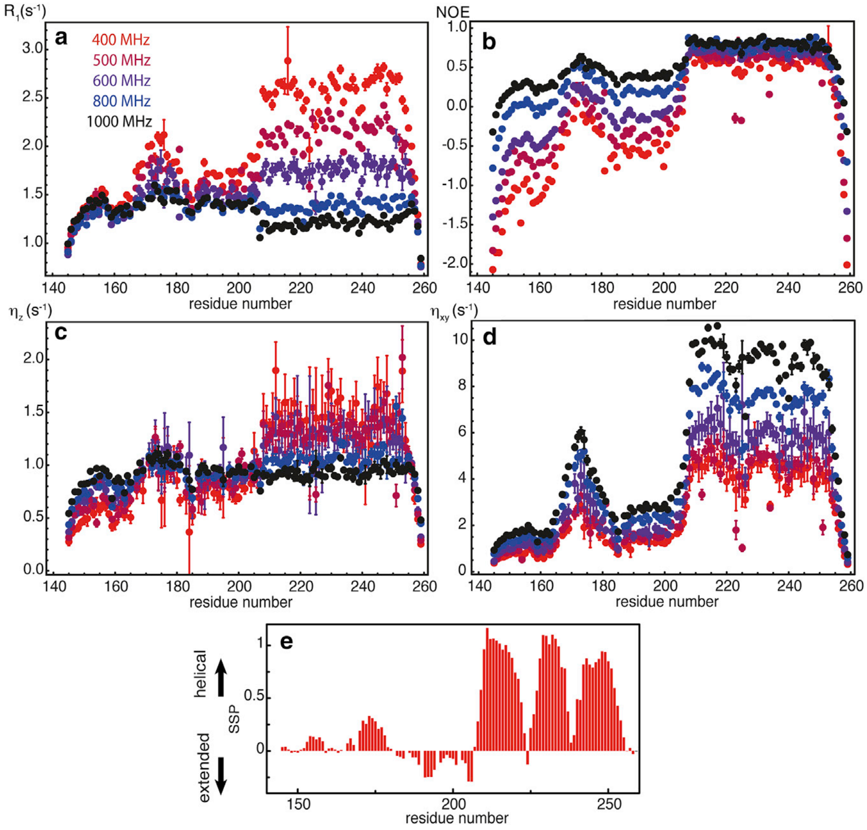

FIGURE 1 Backbone ^{15}N relaxation rates and NOEs measured in Engrailed 2 at five magnetic fields: 400 MHz (*red*), 500 MHz (*burgundy*), 600 MHz (*purple*), 800 MHz (*blue*), and 1000 MHz (*black*). (*a*) Longitudinal relaxation rates, R_1, of ^{15}N. (*b*) ^{15}N-{^{1}H} NOE ratios. (*c*) Longitudinal cross-relaxation rates, η_z, due to correlated fluctuations of the ^{15}N CSA and the ^{15}N-^{1}H dipolar couplings. (*d*) Transverse cross-relaxation rates, η_{xy}, due to the same correlated fluctuations. (*e*) SSP calculated from the chemical shifts of carbonyl and α and β carbon-13 nuclei. To see this figure in color, go online.

(residual) structure. The region connecting the hexapeptide and the homeodomain features negative SSP scores, which suggests a trend toward extended conformations.

Relaxation experiments were carried out at 400, 500, 600, 800, and 1000 MHz (Fig. 1, *a–d*) to determine longitudinal R_1 nitrogen-15 relaxation rates, the steady-state ^{15}N-{^{1}H} NOE, as well as the longitudinal η_z and transverse η_{xy} cross-relaxation rates due to correlated fluctuations of the nitrogen-15 CSA and the dipolar coupling with the amide proton. Transverse relaxation rates, R_2, were measured using Carr-Purcell-Meiboom-Gill echo trains at 800 MHz (Fig. S1 in the Supporting Material). All experiments were analyzed with NMRPipe and the intensities were obtained from a fit of peaks with the routine nlinLS (44). In some exceptional cases, the limited resolution of spectra measured at 9.4 and 11.7 T may have led to inaccuracies in the intensities of a few poorly resolved peaks.

The uniform decrease of the longitudinal relaxation rates, R_1, with increasing magnetic field B_0 in the 200–259 homeodomain (Fig. 1 *a*) indicates motions in the nanosecond range, resulting from overall rotational diffusion. The variations $R_1(B_0)$ are much less pronounced in the disordered region, except in the 169–174 hexapeptide region. Longitudinal cross-relaxation rates, η_z (Fig. 1 *c*), increase with B_0 in the IDR. This reflects the very slow decay, slower than $1/\omega$, of the spectral density function in the range 40–100 MHz (i.e., the range of ^{15}N Larmor frequencies between 9.4 and 23.5 T), as the increase of the amplitude of the CSA interaction counterbalances the decay of the spectral density function with increasing frequency. The profile of transverse relaxation rates, R_2, is marked by variations along the sequence of the protein of both the distribution of picosecond-nanosecond motions and contributions of chemical exchange (Fig. S1). NOEs are sensitive markers of local order in IDPs and IDRs and have been used as such for many years (45). Indeed, the variations of NOEs along the sequence are pronounced at moderate fields (9.4–14.1 T); however, the profile of NOEs is much flatter at high fields, in particular at 23.5 T. On the other hand, transverse cross-correlation rates, η_{xy}, which depend primarily on $J(\omega = 0)$, exhibit sharp variations at all fields that are strongly correlated with SSP scores. This suggests that transverse cross-correlation rates, η_{xy}, should become the method of choice to characterize order in IDPs and IDRs.

Spectral density mapping

Most current software packages designed to characterize protein dynamics based on relaxation rates (46–50) offer a direct derivation of the parameters of local dynamics (order parameters and correlation times for local motions). This approach is efficient and reliable when the theoretical framework of the analysis has been validated. Since a general understanding of motions in intrinsically disordered

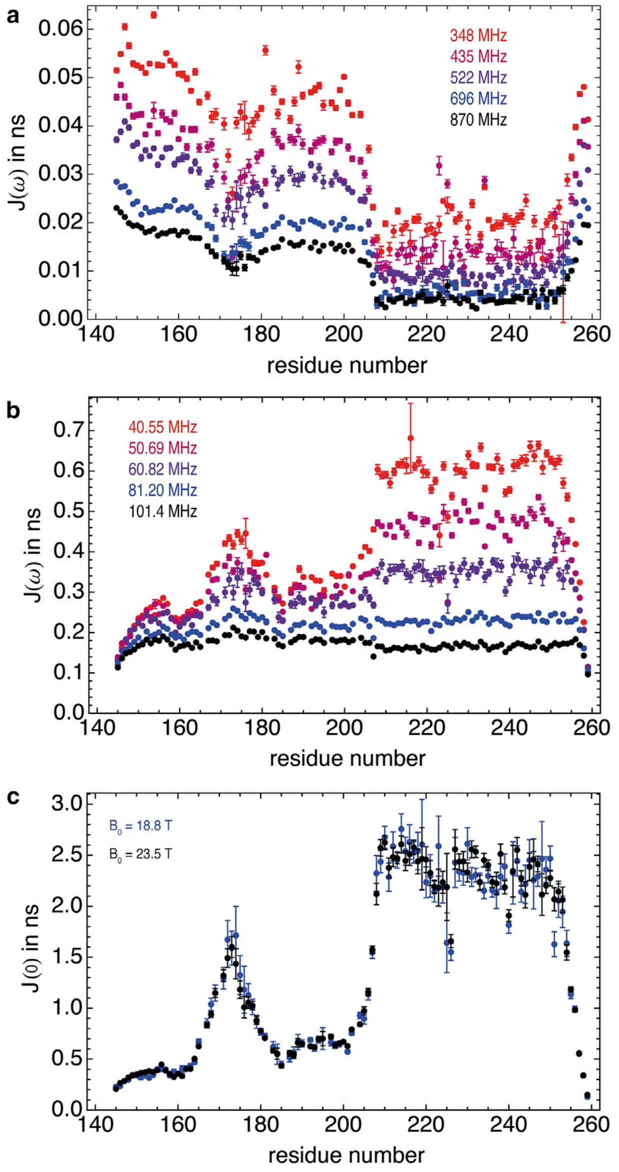

FIGURE 2 Spectral density functions for backbone NH vectors in Engrailed 2. (*a*) Effective spectral density near the proton Larmor frequency, $J(0.87\omega_H)$ (ns). (*b*) Spectral density at the Larmor frequency of nitrogen-15, $J(\omega_N)$ (ns). (*c*) Spectral density at zero frequency, $J(0)$ (ns). All data are color-coded as a function of the magnetic field at which the relaxation rates were recorded, with the same code as in Fig. 1. To see this figure in color, go online.

proteins is still lacking, the derivation of the spectral densities from relaxation rates provides a representation of experimental data that is more amenable to physical analysis than relaxation rates (20).

Spectral density mapping (13) can be achieved without resorting to any proton auto-relaxation rate (51,52). The effective spectral density at high frequency, $J(0.87\omega_H)$ (see Fig. 2 *b*), can be derived from {^{1}H}-^{15}N NOE and longitudinal nitrogen-15 relaxation rates for different magnetic fields according to (51)

$$J(0.87\omega_H) = \frac{4\gamma_N R_1(NOE - 1)}{5d^2\gamma_H}, \quad (1)$$

with $d = (\mu_0/4\pi)(\hbar\gamma_H\gamma_N/\langle r_{NH}^3\rangle)$. μ_0 is the permittivity of free space; γ_H and γ_N are the gyromagnetic ratios of the proton and nitrogen-15 nuclei; $\hbar$ is Planck's constant divided by 2π and $r_{NH} = 1.02$ Å is the distance between the amide proton and the nitrogen-15 nucleus.

Our data recorded at five magnetic fields allowed us to fit the spectral density function at high frequency, $J(0.87\omega_H)$, to the expression

$$J(\omega) = \lambda + \frac{\mu}{\omega^2}, \quad (2)$$

in analogy to an earlier study of carbon-13 relaxation (53). The parameters λ and μ are real positive numbers. This functional form is expected to be a good approximation of the spectral density at high frequency in a folded protein, but not necessarily for a protein with significant motions with correlation times in the hundreds of picoseconds. Nevertheless, we obtain satisfactory fits for all residues in the IDR as well as in the homeodomain. This validates the self-consistency of the use of a single effective frequency, $\omega_{eff} = 0.87\omega_H$, in Eqs. 8–10 of the article by Farrow et al. (51) (see Eqs. S1–S6), where the spectral density function at high frequency was assumed to be of the form of Eq. 2 in both the folded homeodomain and the IDR. Thus, most results of spectral density mapping pertaining to disordered proteins that have been published in recent years are validated.

The results of the fit of the spectral density were used to evaluate contributions to the spectral density at higher frequencies (at $\omega_H \pm \omega_N$) in the derivation of $J(\omega_N)$ from the rate R_1 according to the equation

$$J(\omega_N) = R_1/\left(3d^2/4 + c^2\right) - \left(6J(\omega_H + \omega_N) + J(\omega_H - \omega_N)\right)/\left(3 + 4c^2/d^2\right), \quad (3)$$

with $c = \gamma_N B_0 \Delta\sigma/\sqrt{3}$ and $\Delta\sigma = 160$ ppm is the axially symmetric CSA of the nitrogen-15 nucleus.

Overall, contributions of high-frequency terms to R_1 are small (54), so that the deviations between the values of $J(\omega_N)$ obtained from a series of approximations (51) and the current method are limited to ~2%, which is commensurate with the estimated precision (Fig. S2). Again, this validates, a posteriori, many spectral density mapping studies performed on IDPs. In addition, the low sensitivity of $J(\omega_N)$ upon the model used to describe the spectral density at high frequency shows that the enhanced accuracy expected from more sophisticated approaches, for instance, following Kadeřávek et al. (20), would be smaller than the typical precision of our measurements. The values of $J(\omega_N)$ derived at five magnetic fields are shown in Fig. 2 *b*.

To avoid contributions from line-broadening due to chemical exchange, we did not consider transverse relaxation rates, $R_2(^{15}N)$, and only used longitudinal and transverse CSA/DD cross-correlated relaxation rates, η_z and η_{xy}, to derive the spectral density $J(0)$ from $J(\omega_N)$ using (55)

$$J(0) = J(\omega_N)\frac{3}{4}\left(2\frac{\eta_{xy}}{\eta_z} - 1\right). \quad (4)$$

As can be seen in Fig. S3, measurements of η_z and η_{xy} are not precise enough at lower fields to provide reliable estimates of $J(0)$ by lack of sensitivity (in particular for η_z). However, the data recorded at 18.8 T and 23.5 T (Fig. 2 *c*) are very similar and do not show any of the outliers observed at lower fields. Significant chemical exchange contributions to $R_2(^{15}N)$ can be observed in the hexapeptide region of the disordered region and in the homeodomain (see Fig. S1). Such contributions preclude the proper derivation of $J(0)$ from $R_2(^{15}N)$ rates, in particular at high magnetic fields.

Principles and optimization of IMPACT

The limitations of conventional approaches to the analysis of relaxation rates in IDPs and IDRs result from the complexity of their dynamics. These span at least three orders of magnitude, so that it appears unlikely that they can be accurately described by a single distribution of correlation times or by a small number of correlation times. However, the scarcity of relaxation rates limits the number of adjustable parameters that can be determined and thus the sophistication of spectral density functions that can be postulated. Here, we significantly increase the number of correlation times by defining an array of n fixed correlation times. Only the relative coefficient of each correlation time in the distribution is fitted to experimental data, so that the number of adjustable parameters is reduced. Thus, our only assumption is that the correlation function can be approximated by a sum of exponentials. The physical content of the IMPACT model is thus limited to a minimum. IMPACT can be described as a mathematical approach that converts experimental relaxation rates (or, equivalently, spectral density mapping results) into a distribution of correlation times that is more amenable to physical interpretation than the raw experimental data. The array of n correlation times is defined as a geometric series, so that correlation times are equally spaced on a logarithmic scale (Fig. 3, *a* and *b*):

$$\tau_i = \alpha^{i-1}\tau_{max} \quad \alpha = (\tau_{min}/\tau_{max})^{\frac{1}{n-1}}. \quad (5)$$

Thus, $J(\omega)$ is a sum of Lorentzian functions:

$$J(\omega) = \sum_{i=1}^{n} J_i(\omega) = \frac{2}{5}\sum_{i=1}^{n}\frac{A_i\tau_i}{1 + (\omega\tau_i)^2}, \quad (6)$$

where A_i is the coefficient of correlation time τ_i in the spectral density function. The coefficients A_i must be positive and fulfill the normalization constraint

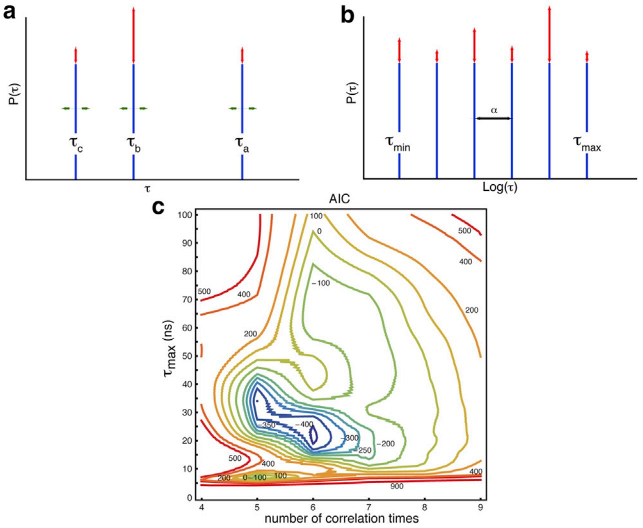

FIGURE 3 Principle and optimization of the parameters of our IMPACT analysis. (*a*) In the 3CT analysis, both the value and the relative weight of each correlation time must be adjusted. (*b*) In IMPACT, the values of the correlation times are fixed and equally spaced on a logarithmic scale, so that only their relative weights need adjusting. (*c*) Optimization of IMPACT by considering AIC. The range (τ_{min}, τ_{max}) of correlation times characterized by IMPACT was varied from (1 ps, 1 ns) to (100 ps, 100 ns) and the number of correlation times was varied in the range $n = 4$–9. Despite the solid lines shown in the contour plot (*c*), the reader should be aware that the number of correlation times is an integer. To see this figure in color, go online.

$$\sum_{i=1}^{n} A_i = 1. \tag{7}$$

Thus, the number of free parameters is $n-1$.

A preliminary step of the IMPACT analysis is the optimization of the three parameters τ_{min}, τ_{max}, and n. The first step is to define the range of correlation times that are probed by relaxation rates. A series of correlation times could be chosen as the inverse of the Larmor frequencies at which the spectral density is mapped, in analogy to the study by LeMaster (56). Considering that the range of frequencies where a Lorentzian function varies extends beyond the inflection point, we chose a slightly different approach. We first define the range of correlation times that are sampled by various ^{15}N relaxation rates. We consider that the lowest magnetic field adapted to protein studies is 400 MHz, whereas the highest accessible field currently is 1 GHz. Thus, the lowest nonzero frequency at which the spectral density is sampled is $\omega_N/2\pi = 40$ MHz, and the highest is $0.87\omega_H/2\pi = 870$ MHz. A Lorentzian function with a correlation time of $\tau_c = 40$ ns drops to 1% of $J(0)$ at $\omega_N/2\pi =$ 40 MHz. A Lorentzian with $\tau_c = 18$ ps merely decreases to 99% of $J(0)$ at $0.87\omega_H/2\pi = 870$ MHz. The resulting range spans slightly more than three orders of magnitude. Therefore, we have decided to limit the range to three orders of magnitude

$$\tau_{max}/\tau_{min} = 10^3. \tag{8}$$

To define the optimal values of τ_{min} and τ_{max}, we carried out a series of IMPACT analyses for $[\tau_{min}, \tau_{max}] = [1$ ps, 1 ns$]$ to $[100$ ps, 100 ns$]$, as well as for $4 < n < 9$. In contrast to the approach of LeMaster (56), the number of correlation times is adjustable. The statistical relevance of each combination of parameters was evaluated from the resulting Akaike's information criteria (AIC) (57–60):

$$\mathrm{AIC} = n_{\mathrm{exp}} \ln\left(\sum_{k=1}^{nres} \chi_k^2 / n_{\mathrm{exp}}\right) + 2n_{\mathrm{model}} + C. \tag{9}$$

$n_{exp} = n_J \times nres$ is the total number of experimental data, with $n_J = 11$ points at which the spectral density function $J(\omega)$ is sampled when relaxation data at five magnetic fields are used; $nres = 108$ is the number of residues included in the analysis; and $n_{model} = (n-1) \times nres$ is the number of free parameters in each model. Here, the constant is $C = 0$. AIC are shown in Fig. 3 *c*. Two local minima were found for $[\tau_{min}, \tau_{max}] = [34$ ps, 34 ns$]$ with $n = 5$ and for $[\tau_{min}, \tau_{max}] = [21$ ps, 21 ns$]$ with $n = 6$. The likelihood of the latter array of correlation times is 10^3 times higher than that of the former. Here, we will thus present the IMPACT analysis with $[\tau_{min}, \tau_{max}] = [21$ ps, 21 ns$]$ and $n = 6$; the analysis with $[\tau_{min}, \tau_{max}] = [34$ ps, 34 ns$]$ and $n = 5$ can be found in the Supporting Material. This result is dominated by the diverse dynamic properties of the IDR of Engrailed. Indeed, if we exclude the rigid residues of the homeodomain, the optimal parameters change slightly (residues 146–207) to $[\tau_{min}, \tau_{max}] = [42$ ps, 42 ns$]$ with $n = 5$, whereas the optimal set of parameters for the rigid part of the homeodomain alone (residues 208–259) is significantly different, $[\tau_{min}, \tau_{max}] = [10$ ps, 10 ns$]$ and $n = 4$.

Application of IMPACT to Engrailed

The optimal parameters $n = 6$ and $[\tau_{min}, \tau_{max}] = [21$ ps, 21 ns$]$ were employed to analyze the spectral density function in Engrailed. Note that the coefficients A_i were fitted to spectral density mapping results. In principle, relaxation rates could also be used directly as input for the IMPACT analysis. Fig. 4 illustrates the remarkable variety of dynamics found in Engrailed. In the homeodomain, the second correlation time, τ_2, lies just below the correlation time for overall rotational diffusion, which is close to 7 ns, as can be seen from the analysis based on only two correlation times (vide infra). Thus, the second coefficient is by far the most important in the homeodomain. A small amplitude A_1 of τ_1 corrects for the fact that τ_2 is shorter than the actual correlation time for the motion of the whole domain. Note that the correlation time for overall diffusion, τ_m, is well approximated by:

$$\tau_m \approx (A_1\tau_1 + A_2\tau_2)/(A_1 + A_2). \tag{10}$$

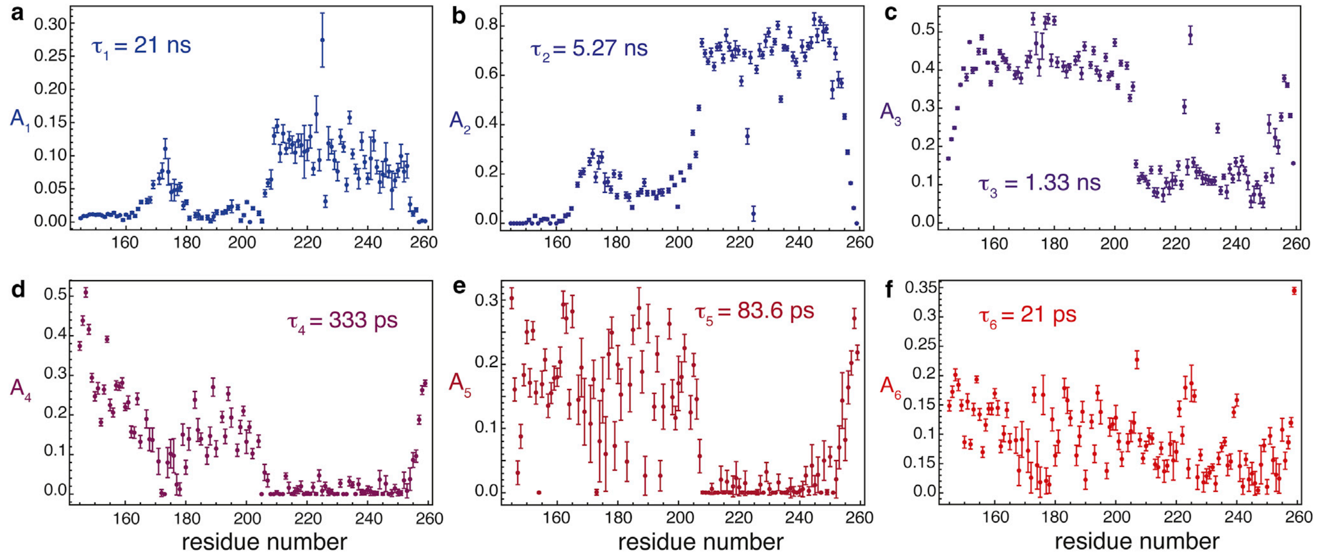

FIGURE 4 Plots of the six coefficients, A_i (i = 1, 2… 6) of the n = 6 correlation times, τ_i, in the range [τ_{min}, τ_{max}] = [21 ps, 21 ns] determined by the IMPACT analysis of Engrailed: (*a*) τ_1 = 21 ns; (*b*) τ_2 = 5.27 ns; (*c*) τ_3 = 1.33 ns; (*d*) τ_4 = 333 ps; (*e*) τ_5 = 83.6 ps; (*f*) τ_6 = 21 ps. To see this figure in color, go online.

The average value over the helices of the homeodomain is $<\tau_m>$ = 7.19 ns, which is in good agreement with an estimate of the correlation time for overall diffusion (see below). Small but significant and mostly uniform contributions A_3 of the correlation time τ_3 in the three α-helices, which are also obtained in a conventional model-free analysis (vide infra), may be attributed to fluctuations of the overall diffusion tensor (61), likely due to conformational fluctuations of the IDR (residues 146–199). Enhanced values of A_3 in the loops may reflect the flexibility of these regions (41). The very small coefficients A_4 and A_5 demonstrate the presence of a gap in the distribution of correlation times, as was also observed in ubiquitin (62). Finally, the coefficients A_6 for the shortest correlation time τ_6 indicate the presence of fast motions in the tens of picoseconds range. Note that the Lorentzian function $J_6(\omega)$ drops by ~1% of $J_6(0)$ at the highest frequency explored in this analysis (i.e., $\omega/2\pi$ = 870 MHz). Thus, this last term in the spectral density function can be approximated to a constant that effectively represents all fast motions:

$$J_6(\omega) \approx \frac{2}{5} A_6 \tau_6 \approx \frac{2}{5} \int_0^{\tau_5} p(\tau) d\tau, \tag{11}$$

where $p(\tau)$ is the probability function of correlation times, containing little information on the complexity of such motions (63).

Results obtained in the disordered region of Engrailed will be discussed with the help of Fig. 4 but also with the IMPACT barcode shown in Fig. 5. In the latter, for each residue, the width of each histogram represents the coefficient A_i associated with the correlation time τ_i that can be read on the y axis. This graph appears to be a convenient way to display the results of the IMPACT analysis in a single figure.

For the first residues at the N-terminus and the last residues at the C-terminus, significant coefficients A_{3-6} are found for the four shortest correlation times. This seems to indicate the presence of motions that are broadly distributed over all timescales up to 1 ns. On the other hand, the two disordered regions just at the N-terminus and the C-terminus of the hexapeptide display a high density of motions around τ_3. The coefficients for the correlation time τ_4 decrease almost linearly with the distance to the N- or C-termini of the polypeptide chain in disordered regions and reach different plateaus in each disordered segment. A notable difference between the disordered region at the N-terminus and the one between the hexapeptide and the homeodomain is the slight decrease of the coefficient A_3 and a significant increase of A_2. It is difficult to assign this change to a particular process without a better characterization of the conformational space of the protein (such characterization is beyond the scope of this article and is a work in progress). Nevertheless, two effects may contribute to the presence of some orientational order beyond 1 ns. First, this IDR is short and located between a folded domain and a small hydrophobic cluster, so that its dynamics is likely influenced by the overall diffusion of both structured elements. Second, this IDR contains a majority of residues that favor extended conformations, as confirmed by SSP scores: three proline residues, nine positively charged, and only one negatively charged residue between positions 177 and 198, which should restrict the conformational space and possibly slow down reorientational dynamics.

The barcode representation of IMPACT coefficients nicely illustrates variations of the ensemble of correlation times

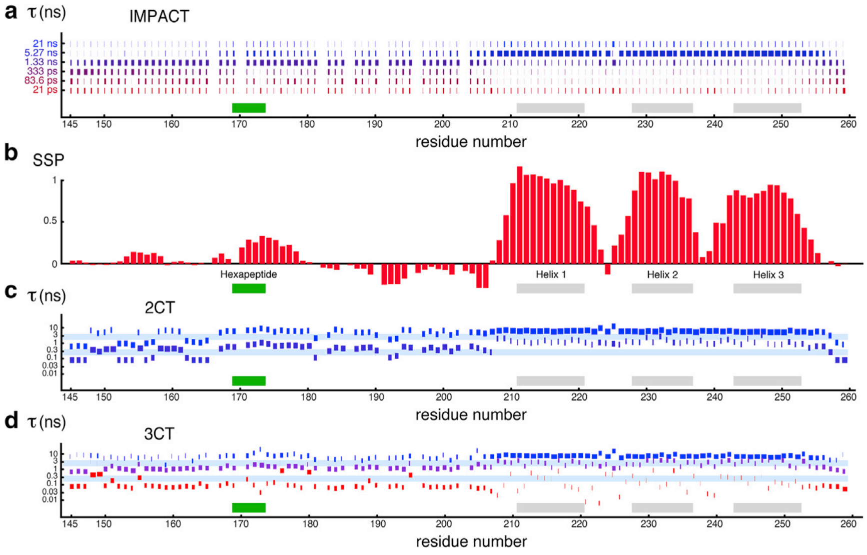

FIGURE 5 Graphical representations of (*a*) IMPACT, (*c*) 2CT, and (*d*) 3CT analyses of the spectral density function in Engrailed 2. Histograms are drawn for all residues and represent the contributions of (*a*) each of the six correlation times, τ_i ($i = 1, 2, \ldots 6$), considered in IMPACT, (*c*) each of the two correlation times, $\tau_{a,b}$, determined by the 2CT analysis, (*d*) each of the three correlation times, $\tau_{a,b,c}$, determined by the 3CT analysis. The width of each rectangle is proportional to the corresponding weights A_i in IMPACT (*a*), $B_{a,b}$ in 2CT (*c*); and $B_{a,b,c}$ in 3CT (*d*). In (*c*) and (*d*), the light blue horizontal bars represent the ranges of correlation times, τ, for which reciprocal frequencies lie in the constrained regions between $40 < 1/(2\pi\ \tau) < 100$ MHz or between $348 < 1/(2\pi\tau) < 870$ MHz. Gray rectangles in (*a*), (*c*), and (*d*) indicate rigid α-helices, and a green rectangle shows the rigid hydrophobic hexapeptide sequence. (*b*) As in Fig. 1 *e*, the SSP is shown to guide the comparison between structural and dynamic features. To see this figure in color, go online.

between successive structural elements. Thus, for instance, the decrease of motions in the subnanosecond range in the hexapeptide is accompanied by an increase in the suprananosecond range. Similarly, variations of the coefficients for correlation times at the N- and C-termini of the homeodomain (residues 200–210 and 254–260) illustrate the smooth transition of motional properties along the sequence of the polypeptide. Finally, even in the homeodomain, where a classical analysis of relaxation data should be most appropriate, the dynamic transitions between helices and the two loops are clearly visible and quantitatively characterized by the IMPACT approach. Loop $\alpha 1$-$\alpha 2$ features enhanced dynamics in both the 1 ns and tens of picoseconds ranges, as expected from the motions demonstrated by paramagnetic relaxation enhancement studies (41), whereas loop $\alpha 2$-$\alpha 3$ shows a significant but more moderate enhancement of motions. Overall, the IMPACT representation offers an elegant view of the correlation of structural and dynamic features, as can be seen from the SSP scores.

Comparison with conventional analyses based on two or three correlation times

For the sake of comparison, we also fit two simple models where the spectral density function is assumed to consist of a sum of two and three Lorentzians in the manner of the familiar model-free and extended model-free approaches. However, as discussed in the Introduction, the core hypotheses of the model-free formalism cannot be fulfilled a priori, since the longest correlation time is probably an effective correlation time rather than the correlation time of overall rotational diffusion. The spectral density, J_{2CT}, assuming two correlation times (2CT) can be written as

$$J_{2CT}(\omega) = \frac{2}{5}\left[S^2\tau_a/\left(1+(\omega\tau_a)^2\right) + \left(1-S^2\right)\tau'_b\Big/\left(1+\left(\omega\tau'_b\right)^2\right)\right], \qquad (12)$$

where $\tau_b'^{-1} = \tau_a^{-1} + \tau_b^{-1}$, τ_a is the long correlation time, τ_b is the short effective correlation time, and S^2 is similar to the model-free order parameter. The spectral density function J_{3CT}, assuming three correlation times (3CT), can be defined as

$$J_{3CT}(\omega) = \frac{2}{5}\left[S^2\tau_a/\left(1+(\omega\tau_a)^2\right) + \left(S_f^2 - S^2\right)\tau'_b/\left(1+\left(\omega\tau'_b\right)^2\right) + \left(1-S_f^2\right)\tau'_c/\left(1+\left(\omega\tau'_c\right)^2\right)\right], \qquad (13)$$

where $\tau_c'^{-1} = \tau_a^{-1} + \tau_c^{-1}$, $\tau_a > \tau_b > \tau_c$, and S_f^2 is equivalent to the extended model-free order parameter for fast processes with a correlation time τ_c. The two functions were fitted to the experimental spectral density, and a simple model selection was based on the comparison of the second order variant of AIC (see the Supporting Material), with $n_J = 11$ and $n_{model} = 3$ for the 2CT analysis and 5 for the 3CT analysis.

Results of this analysis are shown in Fig. 6. From a statistical point of view, the 2CT analysis is found to be sufficient to describe the motions in the mostly rigid homeodomain (residues 200–259), except at the flexible N- and C termini. With few exceptions, the longest correlation time yields a reliable measure of overall rotational diffusion, and the average value over the helices of the homeodomain is $<\tau_a>_{hom} = 7.08$ ns, in good agreement with the IMPACT analysis, with $<\tau_m> = 7.19$ ns. Interestingly, the second

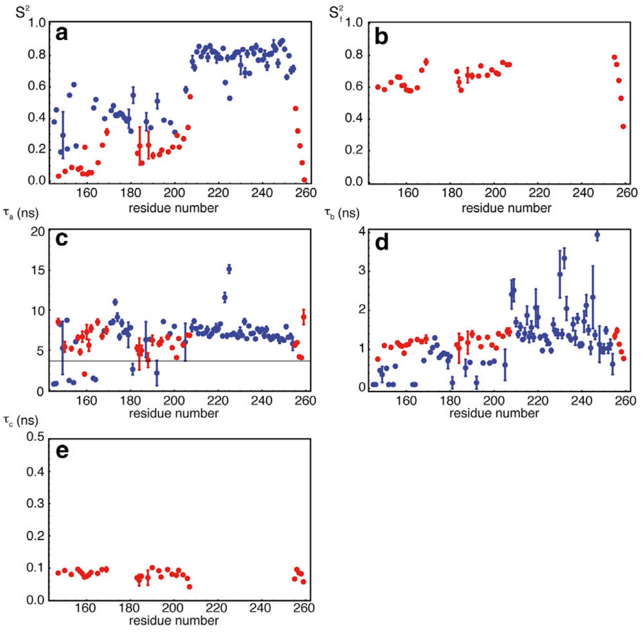

FIGURE 6 Results obtained for a conventional analysis with 2CT (*blue*) or 3CT (*red*). (*a*) Order parameter S^2. (*b*) Order parameter S^2_f for the fastest motion in the 3CT analysis. (*c*) Longest correlation time, τ_a. (*d* and *e*) Intermediate correlation time, τ_b (*d*), and shortest correlation time, τ_c (*e*). Either the 2CT or the 3CT model was selected based on the lowest AIC. To see this figure in color, go online.

correlation time, τ_b, found for most residues in the rigid homeodomain lies in the range $0.9 < \tau_b < 1.8$ ns. This is in agreement with the IMPACT analysis and is possibly due to fluctuations of the overall diffusion tensor resulting from conformational transitions in the disordered N-terminal region on timescales between 1 and 100 ns (61,64). In the disordered region (residues 146–199), the 2CT and 3CT analyses seem equally probable, with no particular pattern along the sequence, except in the hydrophobic hexapeptide cluster (residues 169–174), where the 2CT analysis is more satisfactory. The random patterns of 2CT versus 3CT selection seems to point to some instability of the model-selection step in the fit procedure. A 2CT or 3CT analysis can be performed with no model selection (see Fig. 5). The built-in absence of site-specific model selection in IMPACT shields this analysis from such a drawback. Low order parameters S^2 are found throughout the IDR, with a significant increase in order in the hydrophobic cluster. The long correlation times in the disordered regions have a broad distribution (standard deviation of 2.5 ns), but the average value, $<\tau_a>_{IDR} = 5.9$ ns, is similar to what was found in unfolded proteins (32) and IDPs (65) and very close to the correlation time for overall tumbling of the homeodomain. The intermediate correlation time, which corresponds to the dominant term in the spectral density function, lies in the range $0.1 < \tau_b < 1.4$ ns, in agreement with the IMPACT analysis. The shortest correlation time is rather uniform and lies in the range $40 < \tau_c < 120$ ps.

One should be careful with the physical interpretation of these observations in the IDR of Engrailed. The three correlation times obtained are clearly separated in the time domain, which indicates a broad range of dynamic processes. The results should not be considered a priori as actual correlation times of particular motions, but rather as the best rendition of experimental results using two or three correlation times. This is illustrated by the jumps of order parameters and correlation times observed between the 2CT and 3CT models in the IDR, which illustrates the effective character of the fitted correlation times in this region, at least in the 2CT analysis. For instance, it is difficult to assign the longest correlation time to any particular dynamical process in the absence of complementary experimental or computational information. Such a process could be a single well-defined type of motion, such as the rotational diffusion of an IDR segment. Alternatively, the longest correlation time might account for the tail of a continuous distribution of correlation times and reflect slower motions in parts of the conformational space of the IDR. Interestingly, the correlation times obtained in a 3CT analysis often correspond to reciprocal frequencies ($\omega = 1/\tau$) that lie outside regions where the spectral density function can be adequately sampled (i.e., below 40 MHz, between 100 MHz and 348 MHz, and above 870 MHz). This is particularly true in the flexible region between the rigid hexapeptide and the rigid homeodomain and at the C-terminus of the protein. The regions where the spectral density function is most sensitive to the choice of correlation times correspond to ranges where we lack experimental constraints. This would be expected in the presence of a broad distribution of correlation times that would lead to a smooth decay of the spectral density function.

A direct comparison between the results of the 3CT analysis and our IMPACT approach is illustrated in Fig. 5. For the sake of comparison, we define in Fig. 5 *a* the coefficients $B_{a,b,c}$ associated with the correlation times $\tau_{a,b,c}$, as

$$B_a = S^2, B_b = S^2_f - S^2, B_c = 1 - S^2_f. \quad (14)$$

The statistical significance of the fit resulting from our IMPACT analysis is often better than with either the 2CT or the 3CT analysis in the disordered regions, although it is somehow comparable to that of the 2CT model in the rigid homeodomain (Fig. S4). In particular, the almost complete absence of abnormally elevated χ^2 values in the IDR of Engrailed shows that a faithful set of fitted A_i parameters can be obtained with diverse dynamical features. Interestingly, as can be seen from the schematic representation of correlation times that correspond to reciprocal frequencies that can be determined by spectral density mapping, the inflection points of most of the Lorentzian functions often lie in frequency regions where spectral density mapping does not yield any results. This is particularly true in the IDR between the hexapeptide and the homeodomain and at the C-terminus of the protein. This seems to indicate that the

decay of the spectral density function is smoother than can be described by a sum of three Lorentzian functions. The fit pushes the inflection points of individual contributions to the spectral density function beyond the areas that benefit from rich experimental constraints. In addition, the absence of a residue-specific model selection in IMPACT provides results that are directly comparable, residue by residue, which allows for a better qualitative description of dynamic properties along the protein sequence. Admittedly, model selection can be omitted in the 2CT or 3CT analysis, as in Fig. 5, *a* and *b*.

A potential concern of the IMPACT analysis lies in the fact that the correlation functions $J_i(\omega)$ are not independent, since they suffer from significant overlap. Hence, it is possible that different ensembles of coefficients A_i can describe the same experimental data. To test the sensitivity of our analysis to this potential flaw, we have plotted correlations of consecutive coefficients A_i (i.e., A_i as a function of A_{i+1}) for all 510 steps of the Monte Carlo procedure employed in the fit. Typical results are shown in Fig. S6. There is a small anticorrelation between consecutive coefficients in several instances. This will give rise to a broader distribution of individual coefficients and thus lead to a decrease of the precision of these coefficients. In the worst case, a potential decrease of accuracy due to the interdependence of consecutive coefficients will be accompanied with a decrease in precision.

A potential concern of the IMPACT analysis is the risk of overinterpretation of the results. Here, we should be clear and provide a set of rules that should be followed by users of this approach.

1) The correlation times, τ_i, are not physical correlation times of the system a priori. The range of correlation times is defined by the experimental observables, but the individual values τ_i are derived from a statistical analysis, not a physical analysis.
2) A nonzero coefficient A_1, with τ_1 the longest correlation time, means not that some motions with a correlation time τ_1 were detected but that the distribution of correlation times is larger than zero for some correlation times larger than τ_2.
3) Similarly, as mentioned in Eq. 11, the coefficient A_n of the shortest correlation time τ_n is an effective representation of all fastest motions.
4) If the coefficient A_i is larger than zero, the distribution of correlation times is larger than zero somewhere between τ_{i+1} and τ_{i-1}.
5) If the coefficients A_i and A_{i+1} are both zero, the distribution of correlation times is expected to be zero at least between τ_{i+1} and τ_i.

Finally, very few relaxation studies have compared rates at five or more magnetic fields (66,67). We have tested whether an IMPACT analysis of relaxation rates recorded at only three fields could give meaningful results, using either a broad range of magnetic fields (9.4, 14.1, and 23.5 T) or a narrow range of readily accessible magnetic fields (11.7, 14.1, and 18.8 T). In either case, this requires about two weeks of experimental time. The results of the analysis of relaxation rates at 9.4, 14.1, and 23.5 T shown in Figs. S7 and S8 are remarkably similar to those presented in Figs. 4 and 5. When the range of magnetic fields is restricted, with relaxation rates measured at 11.7, 14.1 and 18.8 T (Figs S9 and S10), some significant changes of IMPACT coefficients can be observed, but the overall description of the distribution of correlation times is very similar to what is obtained with relaxation rates at five magnetic fields. Hence, IMPACT can be applied to many proteins at a moderate cost in experimental time, and does not necessarily require exceptionally large data sets or magnetic fields as high as 23.5 T.

CONCLUSIONS

We have presented a set of nitrogen-15 relaxation rates in the 114-residue, partially disordered protein Engrailed 2 recorded at five different magnetic fields. The transverse cross-correlated rate, η_{xy}, was shown to be the most sensitive to the extent of order and disorder at all magnetic fields. The analysis validates the reduced spectral density mapping approach originally developed for folded proteins and already extensively applied to IDPs and IDRs. The spectral density functions can be fitted reasonably well with two or three correlation times, although such results may be difficult to interpret. We have introduced an approach to the analysis of spectral density functions, which we call IMPACT. This provides a better quantitative description of spectral density functions in IDRs as found in Engrailed than an analysis with three correlation times with the same number of adjustable parameters. We also introduce a barcode representation of IMPACT, which provides a condensed graphical representation of large amounts of data in a single figure. This representation lends itself to a qualitative discussion of order and disorder in proteins. IMPACT can also be useful for analyzing a smaller set of relaxation rates recorded at only three magnetic fields. IMPACT provides a unique framework for the description of the timescales of motions in IDPs and IDRs. Our approach is complementary to the determination of conformational ensembles (7,68). Insight into the dynamics of IDPs and IDRs should greatly benefit from a combined analysis.

SUPPORTING MATERIAL

Supporting Materials and Methods, twelve figures, and thirteen tables are available at http://www.biophysj.org/biophysj/supplemental/S0006-3495(15)00731-6.

AUTHOR CONTRIBUTIONS

F.F., O.L., and P.P. designed the research; S.N.K., C.C., R.A., N.S., O.L., P.P., and F.F. performed the research; C.C., N.S., V.D., G.B., and P.P.

contributed analytical tools; S.N.K., C.C., R.A., and V.D. analyzed the data; and S.N.K., C.C., G.B., O.L., P.P., and F.F. wrote the manuscript.

ACKNOWLEDGMENTS

We thank Bénédicte Elena-Herrmann (École Normale Supérieure de Lyon), Nelly Morellet (International Cancer Screening Network, Gif-sur-Yvette), and Martial Piotto (Bruker, Wissembourg, France) for assistance in recording experiments, and Arthur G. Palmer (Columbia University, New York) and Daniel Abergel (École Normale Supérieure) for many fruitful discussions.

This research was funded by the European Research Council (ERC) under the European Community's Seventh Framework Programme (FP7/2007-2013), ERC grant agreement 279519 (2F4BIODYN), and the Agence Nationale de la Recherche (ANR-11-BS07-031-01). Financial support from the IR-RMN-THC FR3050, Centre Nationale de la Recherche Scientifique, is gratefully acknowledged.

REFERENCES

1. van der Lee, R., M. Buljan, …, M. M. Babu. 2014. Classification of intrinsically disordered regions and proteins. *Chem. Rev.* 114:6589–6631.
2. Dyson, H. J., and P. E. Wright. 2005. Intrinsically unstructured proteins and their functions. *Nat. Rev. Mol. Cell Biol.* 6:197–208.
3. Uversky, V. N., and A. K. Dunker. 2010. Understanding protein non-folding. *Biochim. Biophys. Acta.* 1804:1231–1264.
4. Dunker, A. K., J. D. Lawson, …, Z. Obradovic. 2001. Intrinsically disordered protein. *J. Mol. Graph. Model.* 19:26–59.
5. Jensen, M. R., P. R. L. Markwick, …, M. Blackledge. 2009. Quantitative determination of the conformational properties of partially folded and intrinsically disordered proteins using NMR dipolar couplings. *Structure.* 17:1169–1185.
6. Jensen, M. R., M. Zweckstetter, …, M. Blackledge. 2014. Exploring free-energy landscapes of intrinsically disordered proteins at atomic resolution using NMR spectroscopy. *Chem. Rev.* 114:6632–6660.
7. Marsh, J. A., and J. D. Forman-Kay. 2009. Structure and disorder in an unfolded state under nondenaturing conditions from ensemble models consistent with a large number of experimental restraints. *J. Mol. Biol.* 391:359–374.
8. Tompa, P. 2012. On the supertertiary structure of proteins. *Nat. Chem. Biol.* 8:597–600.
9. Lindorff-Larsen, K., N. Trbovic, …, D. E. Shaw. 2012. Structure and dynamics of an unfolded protein examined by molecular dynamics simulation. *J. Am. Chem. Soc.* 134:3787–3791.
10. Mittermaier, A., and L. E. Kay. 2006. New tools provide new insights in NMR studies of protein dynamics. *Science.* 312:224–228.
11. Palmer, 3rd, A. G. 2004. NMR characterization of the dynamics of biomacromolecules. *Chem. Rev.* 104:3623–3640.
12. Palmer, 3rd, A. G. 2014. Chemical exchange in biomacromolecules: past, present, and future. *J. Magn. Reson.* 241:3–17.
13. Peng, J. W., and G. Wagner. 1992. Mapping of spectral density-functions using heteronuclear NMR relaxation measurements. *J. Magn. Reson.* 98:308–332.
14. Lipari, G., and A. Szabo. 1982. Model-free approach to the interpretation of nuclear magnetic resonance relaxation in macromolecules. 1. Theory and range of validity. *J. Am. Chem. Soc.* 104:4546–4559.
15. Halle, B. 2009. The physical basis of model-free analysis of NMR relaxation data from proteins and complex fluids. *J. Chem. Phys.* 131:224507.
16. Clore, G. M., A. Szabo, …, A. M. Gronenborn. 1990. Deviations from the simple two-parameter model-free approach to the interpretation of nitrogen-15 nuclear magnetic relaxation of proteins. *J. Am. Chem. Soc.* 112:4989–4991.
17. Eliezer, D. 2009. Biophysical characterization of intrinsically disordered proteins. *Curr. Opin. Struct. Biol.* 19:23–30.
18. Jensen, M. R., R. W. H. Ruigrok, and M. Blackledge. 2013. Describing intrinsically disordered proteins at atomic resolution by NMR. *Curr. Opin. Struct. Biol.* 23:426–435.
19. Konrat, R. 2014. NMR contributions to structural dynamics studies of intrinsically disordered proteins. *J. Magn. Reson.* 241:74–85.
20. Kadeřávek, P., V. Zapletal, …, L. Žídek. 2014. Spectral density mapping protocols for analysis of molecular motions in disordered proteins. *J. Biomol. NMR.* 58:193–207.
21. Bussell, Jr., R., and D. Eliezer. 2001. Residual structure and dynamics in Parkinson's disease-associated mutants of α-synuclein. *J. Biol. Chem.* 276:45996–46003.
22. Houben, K., L. Blanchard, …, D. Marion. 2007. Intrinsic dynamics of the partly unstructured PX domain from the Sendai virus RNA polymerase cofactor P. *Biophys. J.* 93:2830–2844.
23. Brutscher, B., R. Brüschweiler, and R. R. Ernst. 1997. Backbone dynamics and structural characterization of the partially folded A state of ubiquitin by ^{1}H, ^{13}C, and ^{15}N nuclear magnetic resonance spectroscopy. *Biochemistry.* 36:13043–13053.
24. Buevich, A. V., and J. Baum. 1999. Dynamics of unfolded proteins: incorporation of distributions of correlation times in the model free analysis of NMR relaxation data. *J. Am. Chem. Soc.* 121:8671–8672.
25. Buevich, A. V., U. P. Shinde, …, J. Baum. 2001. Backbone dynamics of the natively unfolded pro-peptide of subtilisin by heteronuclear NMR relaxation studies. *J. Biomol. NMR.* 20:233–249.
26. Modig, K., and F. M. Poulsen. 2008. Model-independent interpretation of NMR relaxation data for unfolded proteins: the acid-denatured state of ACBP. *J. Biomol. NMR.* 42:163–177.
27. Sternin, E. 2007. Use of inverse theory algorithms in the analysis of biomembrane NMR data. *Methods Mol. Biol.* 400:103–125.
28. Aster, R. C., B. Borchers, and C. H. Thurber. 2012. Parameter Estimation and Inverse Problems, 2nd ed. Academic Press, New York.
29. Foucher, I., M. L. Montesinos, …, A. Trembleau. 2003. Joint regulation of the MAP1B promoter by HNF3β/Foxa2 and Engrailed is the result of a highly conserved mechanism for direct interaction of homeoproteins and Fox transcription factors. *Development.* 130:1867–1876.
30. Peltenburg, L. T. C., and C. Murre. 1996. Engrailed and Hox homeodomain proteins contain a related Pbx interaction motif that recognizes a common structure present in Pbx. *EMBO J.* 15:3385–3393.
31. Augustyniak, R., S. Balayssac, …, O. Lequin. 2011. ^{1}H, ^{13}C and ^{15}N resonance assignment of a 114-residue fragment of Engrailed 2 homeoprotein, a partially disordered protein. *Biomol. NMR Assign.* 5:229–231.
32. Xue, Y., and N. R. Skrynnikov. 2011. Motion of a disordered polypeptide chain as studied by paramagnetic relaxation enhancements, ^{15}N relaxation, and molecular dynamics simulations: how fast is segmental diffusion in denatured ubiquitin? *J. Am. Chem. Soc.* 133:14614–14628.
33. Augustyniak, R., F. Ferrage, …, G. Bodenhausen. 2011. Methods to determine slow diffusion coefficients of biomolecules: applications to Engrailed 2, a partially disordered protein. *J. Biomol. NMR.* 50:209–218.
34. Kay, L. E., L. K. Nicholson, …, D. A. Torchia. 1992. Pulse sequences for removal of the effects of cross-correlation between dipolar and chemical-shift anisotropy relaxation mechanism on the measurement of heteronuclear T_1 and T_2 values in proteins. *J. Magn. Reson.* 97:359–375.
35. Palmer, III, A. G., N. J. Skelton, …, M. Rance. 1992. Suppression of the effects of cross-correlation between dipolar and anisotropic chemical-shift relaxation mechanisms in the measurement of spin spin relaxations rates. *Mol. Phys.* 75:699–711.
36. Ferrage, F. 2012. Protein dynamics by ^{15}N nuclear magnetic relaxation. *Methods Mol. Biol.* 831:141–163.

37. Ferrage, F., D. Cowburn, and R. Ghose. 2009. Accurate sampling of high-frequency motions in proteins by steady-state ^{15}N-^{1}H nuclear Overhauser effect measurements in the presence of cross-correlated relaxation. *J. Am. Chem. Soc.* 131:6048–6049.

38. Ferrage, F., A. Reichel, …, R. Ghose. 2010. On the measurement of ^{15}N-^{1}H nuclear Overhauser effects. 2. Effects of the saturation scheme and water signal suppression. *J. Magn. Reson.* 207:294–303.

39. Pelupessy, P., G. M. Espallargas, and G. Bodenhausen. 2003. Symmetrical reconversion: measuring cross-correlation rates with enhanced accuracy. *J. Magn. Reson.* 161:258–264.

40. Pelupessy, P., F. Ferrage, and G. Bodenhausen. 2007. Accurate measurement of longitudinal cross-relaxation rates in nuclear magnetic resonance. *J. Chem. Phys.* 126:134508.

41. Carlier, L., S. Balayssac, …, O. Lequin. 2013. Investigation of homeodomain membrane translocation properties: insights from the structure determination of engrailed-2 homeodomain in aqueous and membrane-mimetic environments. *Biophys. J.* 105:667–678.

42. Wolfram Research. 2012. Wolfram Mathematica. Wolfram Research, Champaign, IL.

43. Marsh, J. A., V. K. Singh, …, J. D. Forman-Kay. 2006. Sensitivity of secondary structure propensities to sequence differences between α- and γ-synuclein: implications for fibrillation. *Protein Sci.* 15:2795–2804.

44. Delaglio, F., S. Grzesiek, …, A. Bax. 1995. NMRPipe: a multidimensional spectral processing system based on UNIX pipes. *J. Biomol. NMR.* 6:277–293.

45. Dyson, H. J., and P. E. Wright. 2004. Unfolded proteins and protein folding studied by NMR. *Chem. Rev.* 104:3607–3622.

46. Mandel, A. M., M. Akke, and A. G. Palmer, 3rd. 1995. Backbone dynamics of Escherichia coli ribonuclease HI: correlations with structure and function in an active enzyme. *J. Mol. Biol.* 246:144–163.

47. Cole, R., and J. P. Loria. 2003. FAST-Modelfree: a program for rapid automated analysis of solution NMR spin-relaxation data. *J. Biomol. NMR.* 26:203–213.

48. Fushman, D., S. Cahill, and D. Cowburn. 1997. The main-chain dynamics of the dynamin pleckstrin homology (PH) domain in solution: analysis of 15N relaxation with monomer/dimer equilibration. *J. Mol. Biol.* 266:173–194.

49. Dosset, P., J. C. Hus, …, D. Marion. 2000. Efficient analysis of macromolecular rotational diffusion from heteronuclear relaxation data. *J. Biomol. NMR.* 16:23–28.

50. Bieri, M., E. J. d'Auvergne, and P. R. Gooley. 2011. relaxGUI: a new software for fast and simple NMR relaxation data analysis and calculation of ps-ns and μs motion of proteins. *J. Biomol. NMR.* 50:147–155.

51. Farrow, N. A., O. Zhang, …, L. E. Kay. 1995. Spectral density function mapping using 15N relaxation data exclusively. *J. Biomol. NMR.* 6:153–162.

52. Ishima, R., and K. Nagayama. 1995. Protein backbone dynamics revealed by quasi spectral density function analysis of amide N-15 nuclei. *Biochemistry.* 34:3162–3171.

53. Hill, R. B., C. Bracken, …, A. G. Palmer. 2000. Molecular motions and protein folding: Characterization of the backbone dynamics and folding equilibrium of α_2D using ^{13}C NMR spin relaxation. *J. Am. Chem. Soc.* 122:11610–11619.

54. Ropars, V., S. Bouguet-Bonnet, …, C. Roumestand. 2007. Unraveling protein dynamics through fast spectral density mapping. *J. Biomol. NMR.* 37:159–177.

55. Kroenke, C. D., J. P. Loria, …, A. G. Palmer, III. 1998. Longitudinal and transverse ^{1}H-^{15}N dipolar/^{15}N chemical shift anisotropy relaxation interference: unambiguous determination of rotational diffusion tensors and chemical exchange effects in biological macromolecules. *J. Am. Chem. Soc.* 120:7905–7915.

56. LeMaster, D. M. 1995. Larmor frequency selective model free analysis of protein NMR relaxation. *J. Biomol. NMR.* 6:366–374.

57. Akaike, H. 1973. Information theory and an extension of the maximum likelihood principle. *Proc. 2nd Int. Symp. Information Theory.* B. N. Petrov, and F. Csáki, editors. Akadémiai Kiadó, Budapest, Hungary. 267–281.

58. Sugiura, N. 1978. Further analysis of data by Akaike's information criterion and finite corrections. *Commun. Stat. Theory Methods.* 7:13–26.

59. Hurvich, C. M., and C. L. Tsai. 1989. Regression and time-series model selection in small samples. *Biometrika.* 76:297–307.

60. Burnham, K. P., and D. R. Anderson. 2002. Model Selection and Multimodel Inference: A Practical Information-Theoretic Approach. Springer, New York.

61. Wong, V., D. A. Case, and A. Szabo. 2009. Influence of the coupling of interdomain and overall motions on NMR relaxation. *Proc. Natl. Acad. Sci. USA.* 106:11016–11021.

62. Prompers, J. J., and R. Brüschweiler. 2002. General framework for studying the dynamics of folded and nonfolded proteins by NMR relaxation spectroscopy and MD simulation. *J. Am. Chem. Soc.* 124:4522–4534.

63. Calandrini, V., D. Abergel, and G. R. Kneller. 2010. Fractional protein dynamics seen by nuclear magnetic resonance spectroscopy: relating molecular dynamics simulation and experiment. *J. Chem. Phys.* 133:145101.

64. Ryabov, Y. E., and D. Fushman. 2007. A model of interdomain mobility in a multidomain protein. *J. Am. Chem. Soc.* 129:3315–3327.

65. Parigi, G., N. Rezaei-Ghaleh, …, C. Luchinat. 2014. Long-range correlated dynamics in intrinsically disordered proteins. *J. Am. Chem. Soc.* 136:16201–16209.

66. Hall, J. B., and D. Fushman. 2006. Variability of the ^{15}N chemical shielding tensors in the B3 domain of protein G from ^{15}N relaxation measurements at several fields. Implications for backbone order parameters. *J. Am. Chem. Soc.* 128:7855–7870.

67. Charlier, C., S. N. Khan, …, F. Ferrage. 2013. Nanosecond time scale motions in proteins revealed by high-resolution NMR relaxometry. *J. Am. Chem. Soc.* 135:18665–18672.

68. Ozenne, V., F. Bauer, …, M. Blackledge. 2012. Flexible-meccano: a tool for the generation of explicit ensemble descriptions of intrinsically disordered proteins and their associated experimental observables. *Bioinformatics.* 28:1463–1470.

Biophysical Journal

Supporting Material

Distribution of Pico- and Nanosecond Motions in Disordered Proteins from Nuclear Spin Relaxation

Shahid N. Khan,[1,2,3] Cyril Charlier,[1,2,3] Rafal Augustyniak,[1,2,3] Nicola Salvi,[4] Victoire Déjean,[1,2,3] Geoffrey Bodenhausen,[1,2,3,4] Olivier Lequin,[1,2,3] Philippe Pelupessy,[1,2,3] and Fabien Ferrage[1,2,3,*]

[1]Département de Chimie, École Normale Supérieure-PSL Research University, Paris, France; [2]Sorbonne Universités, UPMC Univ Paris 06, LBM, Paris, France; [3]Centre National de la Recherche Scientifique, UMR 7203 LBM, Paris, France; and [4]Institut des Sciences et Ingénierie Chimiques, École Polytechnique Fédérale de Lausanne, BCH, Lausanne, Switzerland

Table of contents:

1. Equations for reduced spectral density mapping: 3
2. Nitrogen-15 transverse relaxation rates at 18.8 T: 4
3. Comparison of reduced spectral density mapping methods: 7
4. Spectral density function at zero frequency: 8
5. Comparison of Akaike Information Criteria: 9
6. 1D Optimization of IMPACT 12
7. Correlations of consecutive IMPACT coefficients: 12
8. IMPACT analysis with relaxation data at five magnetic fields using 5 correlation times: 14
9. IMPACT analysis with relaxation data at three magnetic fields: 15
10. Relaxation rates: 17
11. Spectral density mapping results: 25
12. Two correlation-time analysis of the spectral density function: 33
13. Three correlation-time analysis of the spectral density function: 35
14. IMPACT analysis of the spectral density function: 37
15. References: 40

1. Equations for reduced spectral density mapping:

Reduced spectral density mapping is performed with the use of effective frequencies to account for the spectral density at high frequencies (i.e. $\omega_H + \omega_H$. ω_H. and $\omega_H - \omega_H$). The derivation of the effective frequency for the interpretation of the dipolar cross-relaxation between the ^{15}N and ^{1}H nuclei (Equations 8-10 in Farrow et al.(1)) is reproduced here. We assume that:

$$J(\omega) = \lambda + \mu/\omega^2 \qquad \text{(S1)}$$

We need to derive A and ω_{eff} so that:

$$6J(\omega_H + \omega_N) - J(\omega_H - \omega_N) = AJ(\omega_{eff}) \qquad \text{(S2)}$$

Hence:

$$A = 5 \qquad \text{(S3)}$$

and:

$$6/(\omega_H + \omega_N)^2 - 1/(\omega_H - \omega_N)^2 = 5/\omega_{eff}^2 \qquad \text{(S4)}$$

Using $\omega_H/\omega_N = \gamma_H/\gamma_N$ we obtain:

$$\omega_{eff} = \{5/[6/(1 + \gamma_N/\gamma_H)^2 - 1/(1 - \gamma_N/\gamma_H)^2]\}^{\frac{1}{2}}\omega_H \qquad \text{(S5)}$$

The numeric application gives:

$$\omega_{eff} = 0.870\omega_H \qquad \text{(S6)}$$

2. Nitrogen-15 transverse relaxation rates at 18.8 T:

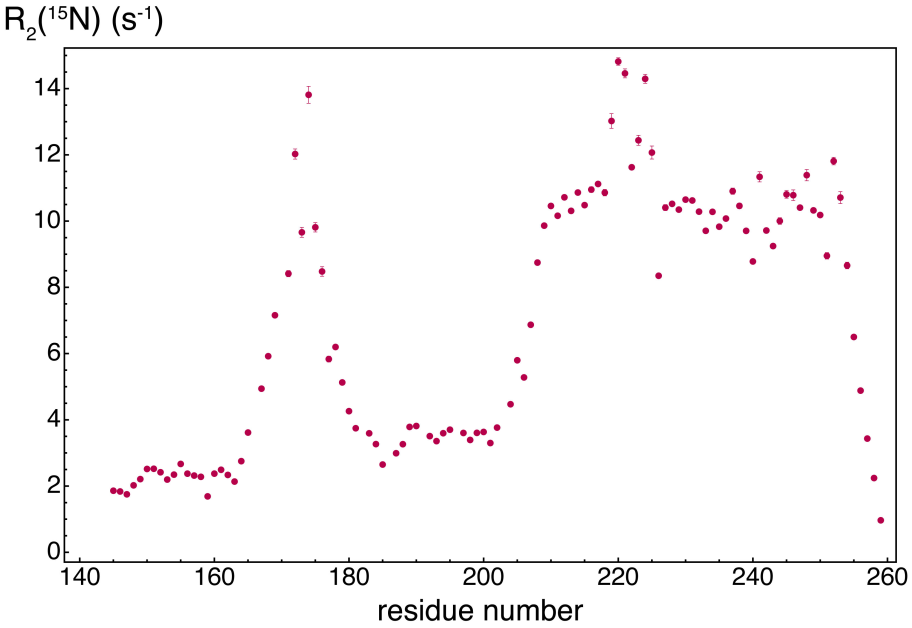

Figure S1. Nitrogen-15 transverse relaxation rates R_2. measured at 18.8 T under a Carr-Purcell-Meiboom-Gill (CPMG) train of 180º pulses and an interpulse delay of 1 ms.

**Table S1**	R_2 (^{15}N) at 18.8 T (s^{-1})		
residue			
145	1.86	±	0.02
146	1.84	±	0.01
147	1.75	±	0.01
148	2.02	±	0.02
149	2.21	±	0.02
150	2.52	±	0.02
151	2.52	±	0.02
152	2.42	±	0.02
153	2.20	±	0.02
154	2.34	±	0.02
155	2.67	±	0.02
156	2.37	±	0.02
157	2.32	±	0.02
158	2.28	±	0.02
159	1.69	±	0.02
160	2.38	±	0.02
161	2.49	±	0.03
162	2.34	±	0.02
163	2.14	±	0.02
164	2.75	±	0.03
165	3.62	±	0.03
167	4.94	±	0.04
168	5.92	±	0.06
169	7.16	±	0.07
171	8.41	±	0.09

172	12.02	±	0.15
173	9.66	±	0.15
174	13.81	±	0.26
175	9.81	±	0.14
176	8.48	±	0.14
177	5.83	±	0.09
178	6.20	±	0.07
179	5.13	±	0.06
180	4.26	±	0.04
181	3.75	±	0.03
183	3.59	±	0.04
184	3.27	±	0.06
185	2.65	±	0.03
187	2.99	±	0.04
188	3.26	±	0.05
189	3.79	±	0.05
190	3.81	±	0.04
192	3.51	±	0.02
193	3.36	±	0.03
194	3.60	±	0.03
195	3.70	±	0.05
197	3.60	±	0.03
198	3.39	±	0.04
199	3.61	±	0.02
200	3.63	±	0.02
201	3.30	±	0.02
202	3.77	±	0.02
204	4.47	±	0.03
205	5.80	±	0.05
206	5.28	±	0.04
207	6.87	±	0.04
208	8.75	±	0.08
209	9.86	±	0.06
210	10.46	±	0.05
211	10.16	±	0.07
212	10.72	±	0.07
213	10.31	±	0.05
214	10.86	±	0.07
215	10.48	±	0.07
216	10.95	±	0.09
217	11.12	±	0.06
218	10.86	±	0.10
219	13.02	±	0.22
220	14.82	±	0.11
221	14.46	±	0.14
222	11.62	±	0.07
223	12.44	±	0.15
224	14.29	±	0.13
225	12.07	±	0.20
226	8.35	±	0.06
227	10.41	±	0.09
228	10.52	±	0.06
229	10.35	±	0.07
230	10.65	±	0.08
231	10.62	±	0.06
232	10.28	±	0.05
233	9.71	±	0.06
234	10.28	±	0.06
235	9.83	±	0.04
236	10.08	±	0.05
237	10.90	±	0.09
238	10.46	±	0.08
239	9.71	±	0.07

240	8.78	±	0.06
241	11.34	±	0.15
242	9.72	±	0.08
243	9.25	±	0.06
244	10.00	±	0.10
245	10.81	±	0.11
246	10.78	±	0.16
247	10.41	±	0.06
248	11.39	±	0.17
249	10.32	±	0.07
250	10.18	±	0.07
251	8.95	±	0.10
252	11.81	±	0.11
253	10.71	±	0.18
254	8.66	±	0.10
255	6.50	±	0.04
256	4.88	±	0.03
257	3.43	±	0.02
258	2.24	±	0.02
259	0.97	±	0.01

3. Comparison of reduced spectral density mapping methods:

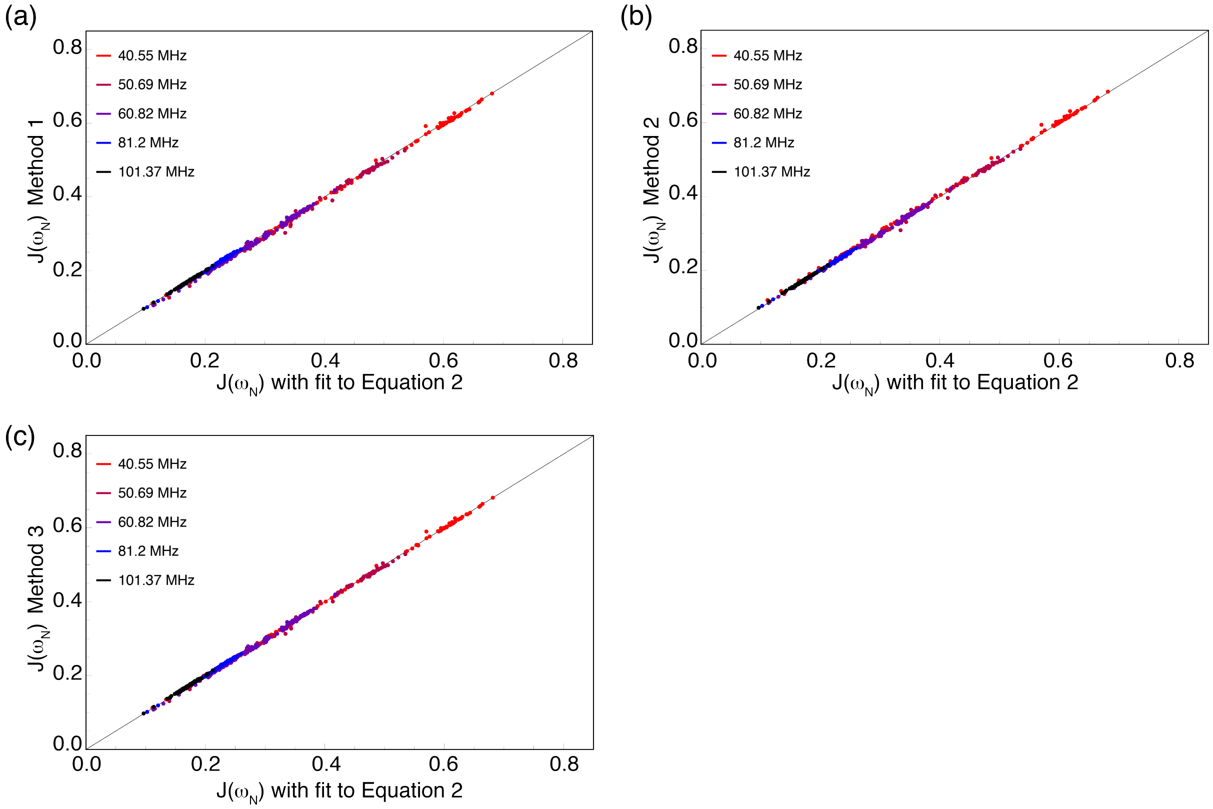

Figure S2. Comparison of the spectral density at the Larmor frequency of nitrogen-15 for a series of approximations. We compare the results obtained in the present study, with a fit of the spectral density function at high frequency with the function of equation 2 with results obtained with the three methods presented in the original reduced spectral density approach.(1) The spectral density function at high frequency (i.e. near ω_H) was derived following: (a) method 1. where for each dataset. the spectral density function is considered to be constant (i.e. $J(\omega_H) = J(\omega_H + \omega_N) = J(\omega_H - \omega_N) = J(0.87\omega_H)$); (b) method 2, where the spectral density at high frequency is derived from $J(0.87\omega_H)$ assuming that $J(\omega) \propto 1/\omega^2$; (c) method 3, where the spectral density at high frequency is derived from the values of $J(0.87\omega_H)$ at two magnetic fields following a linear approximation.

4. Spectral density function at zero frequency:

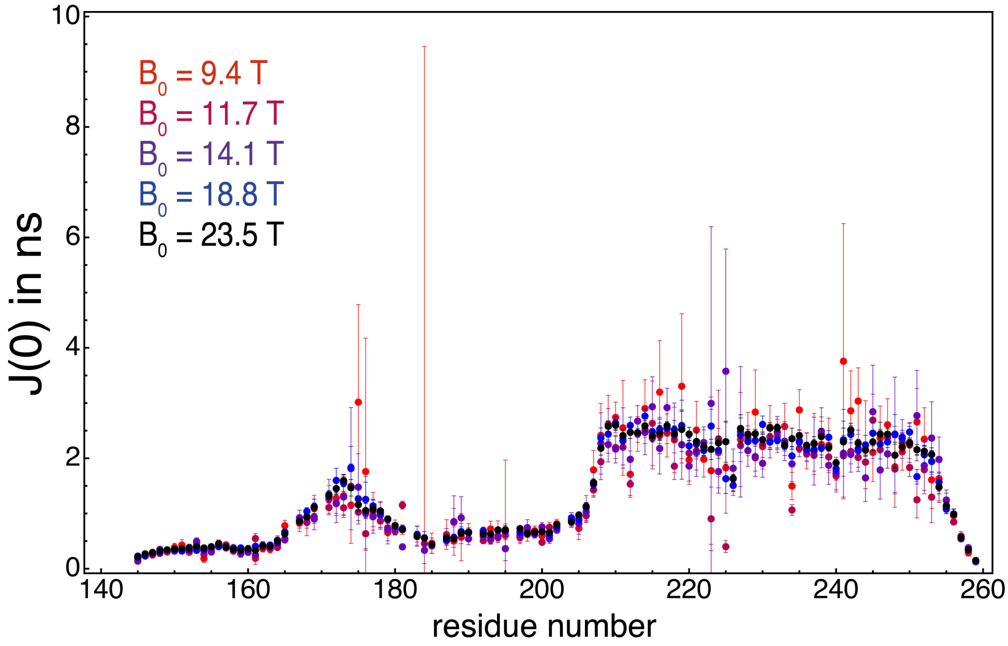

Figure S3. Spectral density function at zero frequency derived from relaxation rates measured at all five magnetic fields. The data obtained at the two highest fields, shown in Figure 2.c are complemented by the values obtained at the three lower fields.

5. Comparison of Akaike Information Criteria:

$$AICc = AIC + \frac{2n_{model}(n_{model}+1)}{n_{exp} - n_{model} - 1}$$

with

$$AIC = n_{exp} ln\left(\frac{\chi^2}{n_{exp}}\right) + 2n_{model} + C$$

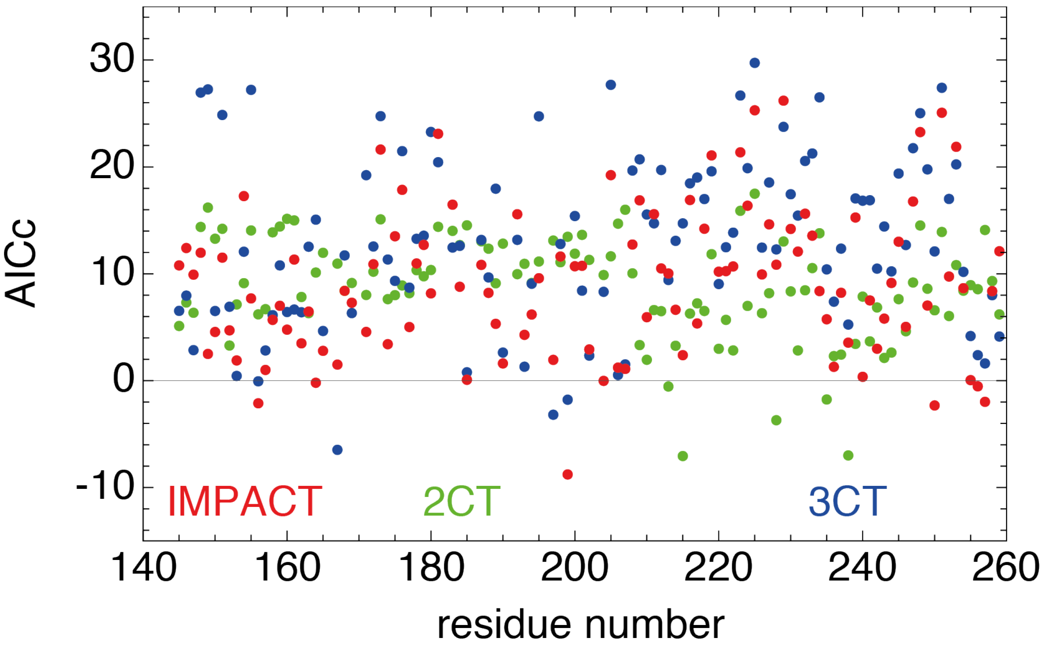

Figure S4. Second order variant of the Akaike Information Criteria AICc's obtained in the IMPACT (red), the two correlation-time (green) and the three correlation-time analyses (blue). Here, n_j = 11, n_{model} = 5 for IMPACT n_{model} = 3 for 2CT and n_{model} = 5 for 3CT analysis.

Table S2

Residue number	AICc 2CT	3CT	IMPACT
145	5.11	6.53	10.78
146	7.31	7.95	12.40
147	6.35	2.85	9.91
148	14.38	26.95	11.97
149	16.19	27.25	2.50
150	13.28	6.51	4.55
151	14.21	24.86	11.51
152	3.28	6.91	4.70
153	7.13	0.44	1.89
154	9.12	12.06	17.27
155	14.05	27.21	7.69
156	6.20	-0.06	-2.12

157	6.69	2.82	1.00
158	13.88	6.12	5.68
159	14.42	10.78	7.00
160	15.14	6.42	4.77
161	14.99	6.65	11.33
162	7.83	6.40	3.49
163	6.31	12.53	6.46
164	10.12	15.06	-0.20
165	11.96	4.64	2.79
167	10.95	-6.48	1.50
168	8.40	11.71	8.40
169	9.14	6.32	7.29
171	8.01	19.22	4.56
172	10.19	12.55	10.89
173	15.09	24.75	21.62
174	7.62	11.33	3.41
175	8.00	9.33	13.51
176	8.90	21.47	17.85
177	8.18	8.70	5.01
178	10.33	13.27	10.96
179	9.76	13.56	12.70
180	10.35	23.27	8.16
181	14.40	20.44	23.10
183	14.01	12.45	16.46
184	12.69	12.61	8.78
185	14.53	0.78	0.08
187	13.03	13.16	10.83
188	12.35	9.66	8.21
189	9.11	17.96	5.31
190	12.82	2.62	1.61
192	9.96	13.17	15.57
193	10.93	1.30	4.28
194	9.14	9.07	6.19
195	11.15	24.73	9.58
197	13.11	-3.19	1.94
198	11.08	12.79	11.61
199	13.46	-1.79	-8.78
200	11.88	15.40	10.70
201	13.64	8.42	10.74
202	11.30	2.34	2.92
204	9.88	8.31	-0.02
205	11.63	27.68	19.23
206	14.69	0.53	1.22
207	15.99	1.52	1.09
208	10.04	19.66	12.74
209	3.33	20.71	16.87
210	1.95	15.54	5.94
211	6.61	14.72	15.58
212	6.50	19.71	10.50
213	-0.55	9.42	10.03

214	3.26	13.08	6.63
215	-7.06	14.71	2.38
216	6.27	18.46	16.90
217	7.23	19.01	5.35
218	6.52	16.99	14.21
219	11.83	19.59	21.07
220	2.98	9.04	10.20
221	5.69	12.48	10.24
222	2.83	13.85	10.69
223	15.89	26.68	21.36
224	6.99	19.88	16.37
225	17.49	29.73	25.30
226	6.31	12.45	9.94
227	8.18	18.54	14.62
228	-3.71	12.28	10.85
229	13.02	23.75	26.20
230	8.35	17.44	14.20
231	2.82	15.44	12.08
232	8.45	20.56	15.62
233	10.53	21.26	13.56
234	13.79	26.51	8.38
235	-1.77	10.41	5.74
236	2.29	7.39	1.29
237	2.43	12.35	8.22
238	-7.00	5.25	3.55
239	3.43	17.05	15.26
240	7.86	16.84	0.36
241	3.67	16.88	7.50
242	6.85	10.48	2.98
243	2.13	14.42	5.80
244	2.63	10.22	9.14
245	7.62	19.38	13.00
246	4.62	12.69	5.03
247	9.18	21.75	16.76
248	14.52	25.03	23.26
249	8.60	19.77	7.02
250	6.58	12.09	-2.32
251	13.91	27.40	25.07
252	6.04	17.00	9.74
253	10.81	20.23	21.88
254	8.41	10.18	8.65
255	8.94	4.17	0.05
256	8.56	2.38	-0.54
257	14.09	1.61	-1.99
258	9.32	7.99	8.37
259	6.20	4.12	12.11

6. 1D Optimization of IMPACT

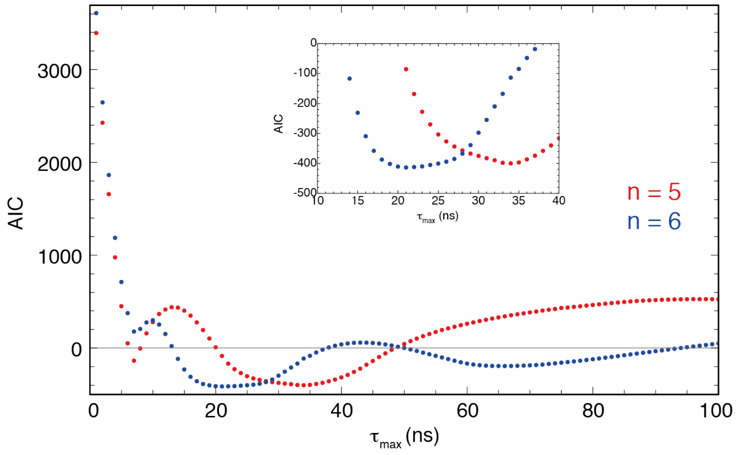

Figure S5. 1D optimization of IMPACT using data at 5 magnetic fields using 5 correlation times (red) of 6 correlation times (bleue).

7. Correlations of consecutive IMPACT coefficients:

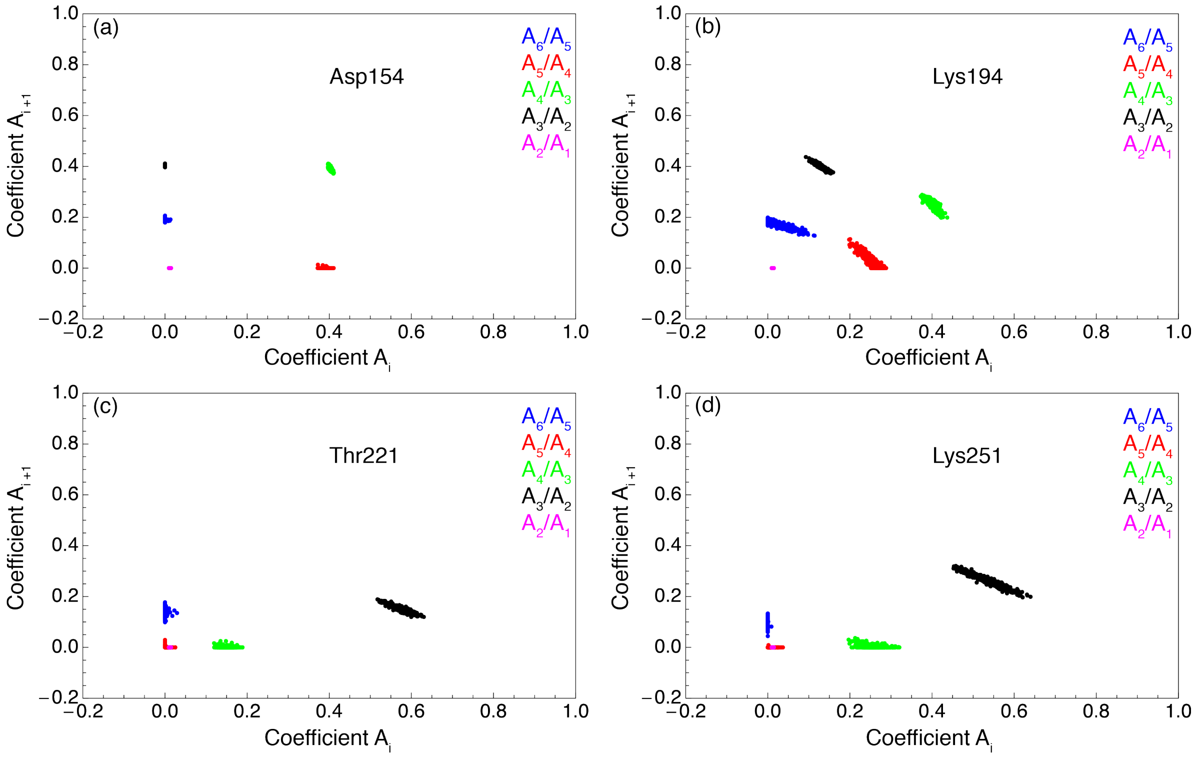

Figure S6. Correlation of consecutive IMPACT coefficients. A_i coefficients are displayed on the x-axis as a function of A_{i+1} coefficients displayed on the y-axis: A_2 as a function of A_1 (magenta); A_3 as a function of A_2 (black); A_4 as a function of A_3 (green); A_5 as a function of

A_4 (red); A_6 as a function of A_5 (blue). Typical results are shown for residues in different regions of the protein: Asp154; Lys194; Thr221; Lys251.

8. IMPACT analysis with relaxation data at five magnetic fields using 5 correlation times:

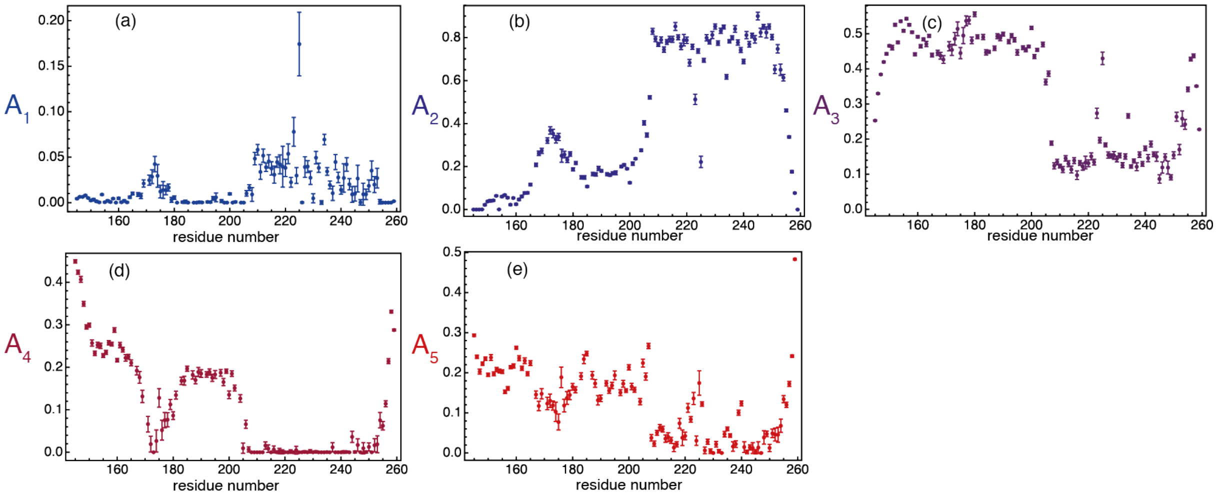

Figure S7. IMPACT results with relaxation rates measured at five magnetic fields. The number of correlation times was n = 5 and the range of correlation times was [34 ps. 34 ns]. (a) A_1 with τ_1 = 34 ns; (b) A_2 with τ_2 = 6.04 ns; (c) A_3 with τ_3 = 1.08 ns; (d) A_4 with τ_4 = 191.2 ps; (e) A_5 with τ_5 = 34 ps.

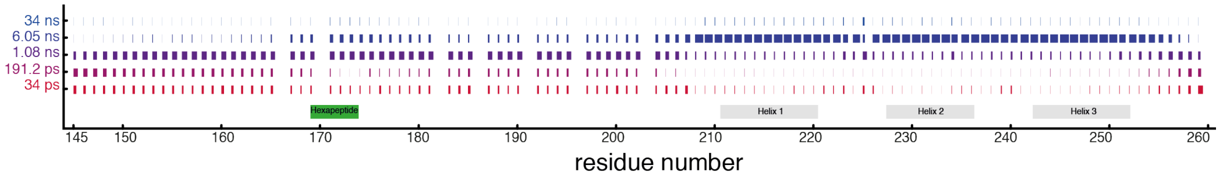

Figure S8. Bar-code representation of the IMPACT analysis of the spectral density function in Engrailed. Histograms are drawn for all residues with the following rules: for each correlation time obtained or used in the analysis of the spectral density function τ_i. a rectangle is represented at the corresponding position along the y-axis. with a logarithmic scale; the width of each rectangle is proportional to the corresponding weight. A_i. The main structural features are illustrated by grey rectangles for alpha helices and a green rectangle for the location of the hydrophobic hexapeptide.

9. IMPACT analysis with relaxation data at three magnetic fields:

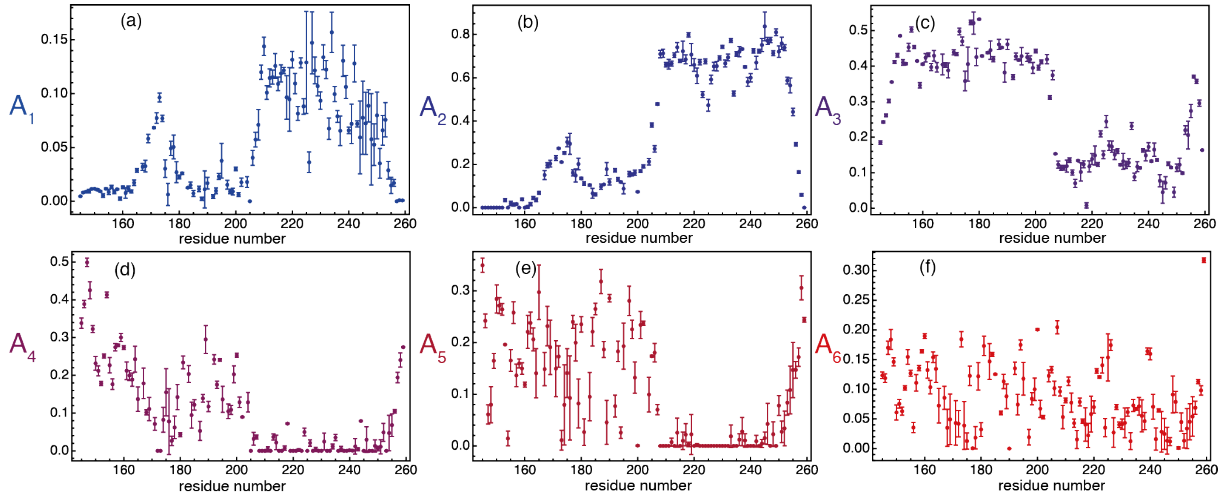

Figure S9. IMPACT results with relaxation rates measured at three magnetic fields: 9.4 T; 14.1 T; and 23.5 T. As for the results presented in Figure 4. the number of correlation times was n = 6 and the range of correlation times was [21 ps. 21 ns]. (a) A_1 with τ_1 = 21 ns; (b) A_2 with τ_2 = 5.27 ns; (c) A_3 with τ_3 = 1.33 ns; (d) A_4 with τ_4 = 333 ps; (e) A_5 with τ_5 = 83.6 ps; (e) A_6 with τ_6 = 21 ps.

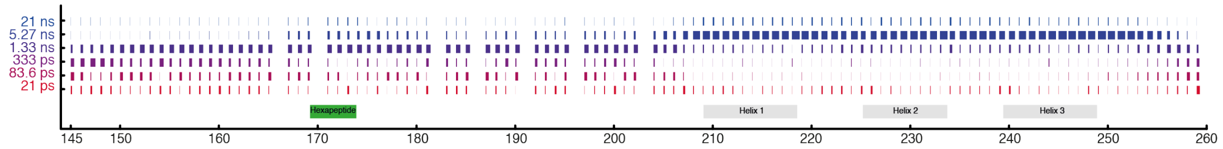

Figure S10. Bar-code representation of the IMPACT analysis of the spectral density function in Engrailed. Histograms are drawn for all residues with the following rules: for each correlation time obtained or used in the analysis of the spectral density function τ_i. a rectangle is represented at the corresponding position along the y-axis. with a logarithmic scale; the width of each rectangle is proportional to the corresponding weight. A_i. The main structural features are illustrated by grey rectangles for alpha helices and a green rectangle for the location of the hydrophobic hexapeptide.

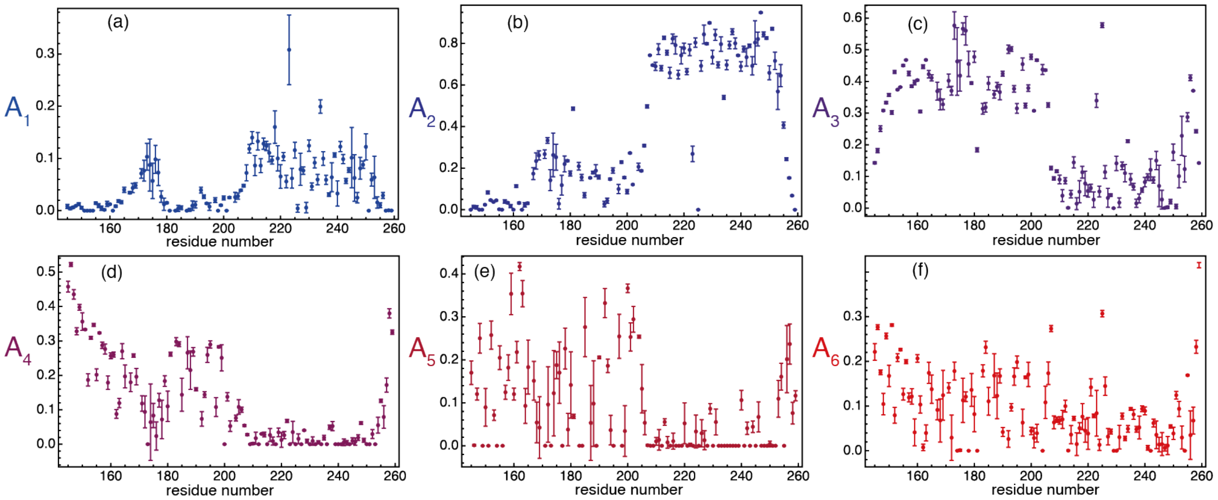

Figure S11. IMPACT results with relaxation rates measured at three magnetic fields: 11.7 T; 14.1 T; and 18.8 T. As for the results presented in Figure 4. the number of correlation times was n = 6 and the range of correlation times was [21 ps. 21 ns]. (a) A_1 with τ_1 = 21 ns; (b) A_2 with τ_2 = 5.27 ns; (c) A_3 with τ_3 = 1.33 ns; (d) A_4 with τ_4 = 333 ps; (e) A_5 with τ_5 = 83.6 ps; (e) A_6 with τ_6 = 21 ps.

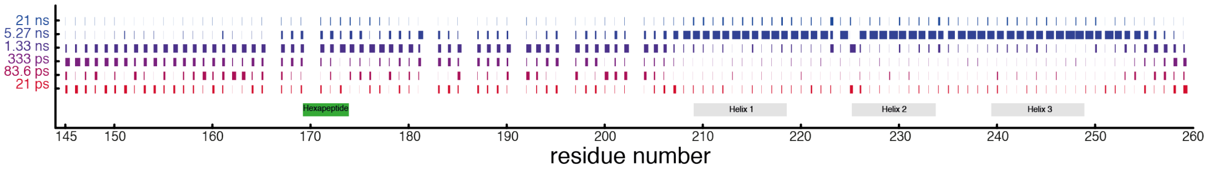

Figure S12. Bar-code representation of the IMPACT analysis of the spectral density function in Engrailed. Histograms are drawn for all residues with the following rules: for each correlation time obtained or used in the analysis of the spectral density function τ_i. a rectangle is represented at the corresponding position along the y-axis. with a logarithmic scale; the width of each rectangle is proportional to the corresponding weight. A_i. The main structural features are illustrated by grey rectangles for alpha helices and a green rectangle for the location of the hydrophobic hexapeptide.

10. Relaxation rates:

Table S3: Nitrogen-15 longitudinal relaxation rates $R_1(^{15}N)$ (s^{-1})

residue	9.4 T			11.8 T			14.1 T			18.8 T			23.5 T		
145	0.956	±	0.007	0.926	±	0.006	0.882	±	0.012	0.911	±	0.002	0.993	±	0.003
146	1.096	±	0.006	1.085	±	0.006	1.008	±	0.011	1.022	±	0.002	1.144	±	0.003
147	1.208	±	0.007	1.111	±	0.008	1.150	±	0.012	1.121	±	0.002	1.227	±	0.003
148	1.246	±	0.009	1.160	±	0.007	1.238	±	0.014	1.168	±	0.003	1.243	±	0.003
149	1.293	±	0.007	1.225	±	0.008	1.238	±	0.014	1.207	±	0.003	1.279	±	0.003
150	1.361	±	0.009	1.310	±	0.009	1.308	±	0.018	1.273	±	0.004	1.344	±	0.004
151	1.379	±	0.011	1.323	±	0.010	1.268	±	0.023	1.188	±	0.004	1.359	±	0.005
152	1.430	±	0.009	1.395	±	0.008	1.388	±	0.018	1.347	±	0.003	1.444	±	0.004
153	1.464	±	0.008	1.335	±	0.008	1.323	±	0.016	1.278	±	0.003	1.374	±	0.004
154	1.449	±	0.009	1.510	±	0.050	1.370	±	0.016	1.308	±	0.003	1.440	±	0.004
155	1.511	±	0.013	1.362	±	0.010	1.296	±	0.019	1.329	±	0.004	1.415	±	0.004
156	1.557	±	0.010	1.455	±	0.013	1.413	±	0.019	1.413	±	0.003	1.495	±	0.004
157	1.516	±	0.010	1.402	±	0.010	1.433	±	0.020	1.384	±	0.004	1.444	±	0.004
158	1.371	±	0.007	1.325	±	0.008	1.359	±	0.015	1.307	±	0.003	1.365	±	0.004
159	1.363	±	0.010	1.295	±	0.009	1.192	±	0.020	1.264	±	0.004	1.269	±	0.005
160	1.331	±	0.010	1.242	±	0.008	1.251	±	0.019	1.274	±	0.003	1.317	±	0.004
161	1.360	±	0.017	1.313	±	0.013	1.304	±	0.033	1.231	±	0.005	1.342	±	0.006
162	1.398	±	0.011	1.354	±	0.013	1.350	±	0.021	1.311	±	0.003	1.361	±	0.005
163	1.400	±	0.011	1.335	±	0.010	1.260	±	0.023	1.338	±	0.005	1.294	±	0.005
164	1.498	±	0.013	1.386	±	0.011	1.382	±	0.026	1.331	±	0.005	1.383	±	0.005
165	1.462	±	0.013	1.330	±	0.015	1.355	±	0.027	1.283	±	0.005	1.304	±	0.005
167	1.742	±	0.020	1.569	±	0.019	1.454	±	0.035	1.369	±	0.007	1.400	±	0.007
168	1.798	±	0.025	1.683	±	0.023	1.551	±	0.046	1.443	±	0.009	1.400	±	0.009
169	1.819	±	0.025	1.680	±	0.023	1.499	±	0.050	1.391	±	0.010	1.369	±	0.010
171	2.000	±	0.029	1.806	±	0.027	1.588	±	0.059	1.468	±	0.013	1.463	±	0.013
172	2.012	±	0.036	1.873	±	0.033	1.727	±	0.082	1.507	±	0.017	1.461	±	0.017
173	1.883	±	0.046	1.689	±	0.036	1.557	±	0.089	1.606	±	0.020	1.599	±	0.021
174	2.103	±	0.044	1.837	±	0.056	1.760	±	0.112	1.578	±	0.027	1.536	±	0.027
175	2.052	±	0.045	1.760	±	0.036	1.849	±	0.111	1.521	±	0.018	1.457	±	0.017
176	2.119	±	0.156	1.560	±	0.052	1.477	±	0.111	1.468	±	0.020	1.420	±	0.018
177	1.904	±	0.039	1.762	±	0.032	1.638	±	0.079	1.561	±	0.014	1.543	±	0.014
178	1.817	±	0.032	1.744	±	0.030	1.754	±	0.064	1.528	±	0.011	1.543	±	0.011
179	1.832	±	0.031	1.701	±	0.027	1.708	±	0.060	1.473	±	0.009	1.442	±	0.010
180	1.840	±	0.017	1.574	±	0.014	1.611	±	0.037	1.514	±	0.006	1.542	±	0.007
181	1.748	±	0.015	1.969	±	0.016	1.270	±	0.025	1.455	±	0.005	1.427	±	0.005
183	1.628	±	0.023	1.585	±	0.019	1.554	±	0.049	1.339	±	0.008	1.428	±	0.008
184	1.503	±	0.032	1.472	±	0.023	1.334	±	0.061	1.297	±	0.010	1.329	±	0.010
185	1.450	±	0.014	1.358	±	0.013	1.304	±	0.030	1.266	±	0.005	1.315	±	0.006
187	1.576	±	0.020	1.551	±	0.019	1.489	±	0.045	1.349	±	0.007	1.381	±	0.008
188	1.640	±	0.027	1.620	±	0.024	1.500	±	0.053	1.413	±	0.008	1.419	±	0.009
189	1.834	±	0.038	1.646	±	0.032	1.580	±	0.069	1.412	±	0.009	1.461	±	0.010
190	1.717	±	0.020	1.615	±	0.022	1.531	±	0.040	1.450	±	0.006	1.471	±	0.007
192	1.725	±	0.015	1.391	±	0.012	1.563	±	0.027	1.399	±	0.004	1.388	±	0.005
193	1.693	±	0.018	1.487	±	0.017	1.486	±	0.033	1.433	±	0.006	1.430	±	0.006
194	1.699	±	0.020	1.542	±	0.017	1.532	±	0.038	1.382	±	0.006	1.413	±	0.007
195	1.586	±	0.032	1.525	±	0.021	1.394	±	0.058	1.318	±	0.008	1.360	±	0.009
197	1.743	±	0.020	1.511	±	0.014	1.473	±	0.036	1.404	±	0.006	1.420	±	0.006
198	1.712	±	0.019	1.626	±	0.016	1.569	±	0.038	1.382	±	0.006	1.384	±	0.007
199	1.740	±	0.012	1.562	±	0.010	1.476	±	0.021	1.384	±	0.003	1.406	±	0.004
200	1.623	±	0.009	1.431	±	0.008	1.502	±	0.024	1.389	±	0.004	1.417	±	0.005
201	1.721	±	0.015	1.753	±	0.014	1.459	±	0.027	1.344	±	0.004	1.378	±	0.005
202	1.801	±	0.014	1.559	±	0.011	1.468	±	0.022	1.422	±	0.004	1.401	±	0.005
204	1.899	±	0.018	1.706	±	0.013	1.554	±	0.033	1.491	±	0.005	1.434	±	0.006
205	1.869	±	0.023	1.664	±	0.019	1.582	±	0.050	1.454	±	0.008	1.347	±	0.007
206	1.891	±	0.023	1.702	±	0.020	1.513	±	0.044	1.358	±	0.007	1.326	±	0.007
207	2.018	±	0.023	1.666	±	0.018	1.375	±	0.035	1.150	±	0.005	1.057	±	0.005

208	2.572	±	0.049	2.148	±	0.032	1.719	±	0.069	1.397	±	0.010	1.205	±	0.009
209	2.501	±	0.037	2.103	±	0.025	1.815	±	0.058	1.370	±	0.007	1.199	±	0.008
210	2.537	±	0.033	2.030	±	0.022	1.641	±	0.044	1.321	±	0.006	1.128	±	0.006
211	2.428	±	0.049	2.111	±	0.032	1.754	±	0.070	1.324	±	0.008	1.147	±	0.008
212	2.551	±	0.039	1.997	±	0.027	1.762	±	0.059	1.300	±	0.008	1.181	±	0.007
213	2.657	±	0.032	2.179	±	0.022	1.794	±	0.043	1.338	±	0.005	1.178	±	0.006
214	2.648	±	0.045	2.109	±	0.027	1.812	±	0.064	1.333	±	0.008	1.152	±	0.008
215	2.621	±	0.080	2.032	±	0.042	1.707	±	0.064	1.376	±	0.008	1.193	±	0.008
216	2.884	±	0.350	2.227	±	0.035	1.675	±	0.065	1.363	±	0.009	1.180	±	0.009
217	2.622	±	0.038	2.214	±	0.027	1.783	±	0.057	1.371	±	0.007	1.183	±	0.007
218	2.672	±	0.048	1.943	±	0.029	1.817	±	0.075	1.319	±	0.010	1.121	±	0.010
219	2.536	±	0.061	2.156	±	0.054	1.749	±	0.119	1.360	±	0.021	1.232	±	0.022
220	2.540	±	0.041	2.175	±	0.031	1.698	±	0.066	1.355	±	0.010	1.178	±	0.010
221	2.384	±	0.044	1.911	±	0.036	1.639	±	0.077	1.219	±	0.012	1.153	±	0.011
222	2.474	±	0.034	2.114	±	0.024	1.728	±	0.054	1.325	±	0.008	1.154	±	0.007
223	1.967	±	0.120	1.583	±	0.049	1.734	±	0.086	1.314	±	0.015	1.192	±	0.014
224	2.655	±	0.044	2.287	±	0.087	1.748	±	0.079	1.412	±	0.012	1.241	±	0.012
225	2.210	±	0.064	1.361	±	0.019	1.416	±	0.117	1.374	±	0.021	1.235	±	0.028
226	2.346	±	0.039	2.038	±	0.029	1.799	±	0.065	1.351	±	0.008	1.178	±	0.008
227	2.596	±	0.056	2.268	±	0.030	1.636	±	0.077	1.398	±	0.011	1.261	±	0.012
228	2.585	±	0.033	2.188	±	0.022	1.770	±	0.052	1.374	±	0.007	1.209	±	0.007
229	2.496	±	0.047	2.362	±	0.035	1.833	±	0.070	1.416	±	0.009	1.281	±	0.009
230	2.725	±	0.043	2.283	±	0.028	1.707	±	0.063	1.491	±	0.009	1.239	±	0.009
231	2.661	±	0.037	2.183	±	0.024	1.798	±	0.053	1.370	±	0.007	1.192	±	0.007
232	2.607	±	0.030	2.161	±	0.020	1.750	±	0.043	1.414	±	0.005	1.185	±	0.006
233	2.794	±	0.042	2.338	±	0.027	1.841	±	0.059	1.486	±	0.008	1.296	±	0.009
234	2.457	±	0.034	1.941	±	0.017	1.696	±	0.059	1.428	±	0.007	1.232	±	0.007
235	2.619	±	0.031	2.123	±	0.019	1.731	±	0.041	1.325	±	0.005	1.151	±	0.005
236	2.603	±	0.030	2.106	±	0.020	1.864	±	0.043	1.387	±	0.006	1.194	±	0.006
237	2.694	±	0.053	2.257	±	0.036	1.821	±	0.077	1.409	±	0.011	1.206	±	0.009
238	2.590	±	0.043	2.192	±	0.030	1.803	±	0.073	1.367	±	0.010	1.235	±	0.009
239	2.361	±	0.039	2.020	±	0.026	1.510	±	0.055	1.319	±	0.008	1.104	±	0.006
240	2.392	±	0.033	1.979	±	0.024	1.641	±	0.050	1.303	±	0.007	1.179	±	0.007
241	2.637	±	0.060	2.206	±	0.042	1.819	±	0.109	1.429	±	0.017	1.226	±	0.017
242	2.653	±	0.042	2.162	±	0.026	1.897	±	0.071	1.452	±	0.010	1.269	±	0.010
243	2.695	±	0.041	2.203	±	0.027	1.804	±	0.060	1.426	±	0.008	1.245	±	0.008
244	2.708	±	0.055	2.151	±	0.034	1.821	±	0.084	1.441	±	0.012	1.220	±	0.012
245	2.791	±	0.057	2.269	±	0.045	1.680	±	0.086	1.427	±	0.013	1.189	±	0.012
246	2.726	±	0.055	2.235	±	0.047	1.777	±	0.093	1.374	±	0.018	1.209	±	0.016
247	2.819	±	0.042	2.424	±	0.028	1.844	±	0.057	1.473	±	0.007	1.287	±	0.008
248	2.591	±	0.065	2.300	±	0.056	1.688	±	0.108	1.382	±	0.018	1.199	±	0.017
249	2.744	±	0.038	2.183	±	0.027	1.783	±	0.057	1.348	±	0.008	1.146	±	0.008
250	2.693	±	0.038	2.158	±	0.025	1.846	±	0.057	1.458	±	0.008	1.225	±	0.008
251	2.695	±	0.054	1.785	±	0.033	2.078	±	0.095	1.479	±	0.013	1.266	±	0.018
252	2.707	±	0.047	2.068	±	0.055	1.726	±	0.076	1.370	±	0.012	1.241	±	0.011
253	2.477	±	0.040	1.966	±	0.097	1.668	±	0.121	1.483	±	0.020	1.282	±	0.025
254	2.381	±	0.040	2.022	±	0.038	1.846	±	0.077	1.399	±	0.013	1.297	±	0.013
255	2.189	±	0.021	1.836	±	0.018	1.701	±	0.038	1.412	±	0.006	1.327	±	0.007
256	2.001	±	0.017	1.734	±	0.013	1.636	±	0.030	1.476	±	0.005	1.405	±	0.006
257	1.670	±	0.013	1.513	±	0.010	1.425	±	0.022	1.352	±	0.004	1.331	±	0.005
258	1.294	±	0.008	1.193	±	0.007	1.220	±	0.015	1.117	±	0.003	1.176	±	0.004
259	0.783	±	0.003	0.762	±	0.003	0.756	±	0.005	0.769	±	0.001	0.843	±	0.001

Table S4: Longitudinal cross-correlated cross-relaxation rates η_z (s^{-1})

residue	9.4 T			11.8 T			14.1 T			18.8 T			23.5 T		
145	0.276	±	0.037	0.315	±	0.016	0.366	±	0.029	0.437	±	0.012	0.543	±	0.007
146	0.357	±	0.024	0.415	±	0.013	0.465	±	0.020	0.583	±	0.008	0.654	±	0.005
147	0.395	±	0.027	0.527	±	0.017	0.526	±	0.021	0.654	±	0.009	0.748	±	0.005
148	0.408	±	0.035	0.603	±	0.021	0.548	±	0.027	0.676	±	0.012	0.747	±	0.006
149	0.457	±	0.029	0.555	±	0.018	0.659	±	0.024	0.723	±	0.011	0.820	±	0.006
150	0.481	±	0.042	0.585	±	0.022	0.653	±	0.039	0.760	±	0.016	0.822	±	0.009
151	0.494	±	0.069	0.572	±	0.029	0.662	±	0.064	0.723	±	0.024	0.827	±	0.013
152	0.613	±	0.040	0.675	±	0.020	0.739	±	0.034	0.839	±	0.013	0.942	±	0.008
153	0.541	±	0.037	0.615	±	0.019	0.657	±	0.031	0.835	±	0.012	0.849	±	0.007
154	0.628	±	0.065	0.452	±	0.036	0.798	±	0.029	0.883	±	0.013	0.969	±	0.007
155	0.577	±	0.050	0.852	±	0.033	0.811	±	0.045	0.797	±	0.017	0.898	±	0.009
156	0.625	±	0.048	0.698	±	0.034	0.819	±	0.040	0.906	±	0.015	0.968	±	0.009
157	0.613	±	0.044	0.683	±	0.023	0.762	±	0.038	0.884	±	0.016	0.948	±	0.009
158	0.536	±	0.030	0.643	±	0.019	0.682	±	0.026	0.832	±	0.012	0.900	±	0.006
159	0.423	±	0.043	0.488	±	0.023	0.629	±	0.043	0.605	±	0.017	0.684	±	0.010
160	0.454	±	0.063	0.594	±	0.022	0.688	±	0.047	0.791	±	0.017	0.849	±	0.001
161	0.598	±	0.139	0.530	±	0.046	0.738	±	0.112	0.746	±	0.036	0.879	±	0.021
162	0.556	±	0.044	0.641	±	0.053	0.708	±	0.040	0.780	±	0.016	0.839	±	0.009
163	0.524	±	0.044	0.568	±	0.024	0.639	±	0.043	0.686	±	0.019	0.738	±	0.010
164	0.631	±	0.064	0.698	±	0.029	0.717	±	0.053	0.833	±	0.021	0.900	±	0.012
165	0.506	±	0.057	0.673	±	0.035	0.797	±	0.051	0.775	±	0.021	0.837	±	0.011
167	0.767	±	0.084	0.782	±	0.045	0.834	±	0.069	0.883	±	0.031	0.931	±	0.015
168	0.864	±	0.111	0.914	±	0.057	0.940	±	0.094	0.921	±	0.040	1.022	±	0.019
169	0.756	±	0.117	0.901	±	0.060	0.962	±	0.102	0.945	±	0.047	0.949	±	0.024
171	1.015	±	0.131	1.024	±	0.073	1.086	±	0.123	1.089	±	0.066	1.066	±	0.034
172	1.054	±	0.158	1.046	±	0.088	1.083	±	0.158	0.979	±	0.079	1.033	±	0.040
173	1.157	±	0.185	1.264	±	0.104	1.233	±	0.195	1.099	±	0.089	1.091	±	0.048
174	1.001	±	0.240	0.916	±	0.159	0.988	±	0.228	1.048	±	0.125	1.109	±	0.070
175	0.668	±	0.260	0.921	±	0.106	0.954	±	0.223	0.940	±	0.099	1.040	±	0.050
176	0.887	±	0.463	0.937	±	0.190	1.140	±	0.354	0.985	±	0.141	1.049	±	0.076
177	1.056	±	0.223	1.023	±	0.102	1.173	±	0.190	1.033	±	0.074	1.103	±	0.040
178	0.991	±	0.150	0.936	±	0.075	1.030	±	0.141	1.000	±	0.055	1.015	±	0.028
179	1.009	±	0.172	0.979	±	0.084	0.976	±	0.149	1.002	±	0.052	0.993	±	0.029
180	0.856	±	0.075	1.082	±	0.045	1.088	±	0.074	1.053	±	0.029	1.080	±	0.015
181	0.669	±	0.051	1.180	±	0.048	1.084	±	0.050	0.959	±	0.018	1.003	±	0.009
183	0.694	±	0.156	0.743	±	0.063	0.936	±	0.141	0.864	±	0.051	0.943	±	0.013
184	0.367	±	0.627	0.735	±	0.100	1.095	±	0.312	0.787	±	0.100	0.858	±	0.060
185	0.517	±	0.081	0.586	±	0.038	0.578	±	0.082	0.690	±	0.029	0.770	±	0.017
187	0.694	±	0.145	0.764	±	0.061	0.878	±	0.127	0.915	±	0.044	0.924	±	0.024
188	0.843	±	0.213	0.653	±	0.115	0.822	±	0.199	0.903	±	0.060	0.982	±	0.037
189	0.759	±	0.230	0.803	±	0.123	0.811	±	0.227	0.927	±	0.074	0.976	±	0.045
190	0.785	±	0.099	0.873	±	0.059	0.839	±	0.081	0.907	±	0.029	0.930	±	0.017
192	0.734	±	0.065	0.820	±	0.032	0.904	±	0.052	0.878	±	0.019	0.972	±	0.011
193	0.732	±	0.088	0.808	±	0.046	0.902	±	0.074	0.909	±	0.027	0.967	±	0.016
194	0.687	±	0.107	0.837	±	0.048	0.914	±	0.090	0.906	±	0.030	0.956	±	0.019
195	0.880	±	0.379	0.797	±	0.097	1.168	±	0.286	0.812	±	0.089	0.862	±	0.064
197	0.785	±	0.110	0.836	±	0.045	0.930	±	0.098	0.967	±	0.032	0.912	±	0.019
198	0.753	±	0.100	0.738	±	0.041	0.828	±	0.082	0.944	±	0.031	0.943	±	0.019
199	0.682	±	0.046	0.782	±	0.023	0.856	±	0.039	0.883	±	0.014	0.935	±	0.008
200	0.691	±	0.033	1.039	±	0.029	0.920	±	0.048	0.941	±	0.017	0.986	±	0.010
201	0.810	±	0.060	1.108	±	0.049	0.876	±	0.052	0.934	±	0.018	0.934	±	0.011
202	0.778	±	0.051	0.992	±	0.032	0.869	±	0.041	0.907	±	0.015	0.932	±	0.009
204	0.827	±	0.084	0.896	±	0.038	0.957	±	0.070	0.967	±	0.023	0.987	±	0.014
205	1.135	±	0.171	0.858	±	0.058	1.019	±	0.129	0.978	±	0.045	0.884	±	0.025
206	0.908	±	0.123	1.033	±	0.061	1.000	±	0.101	0.927	±	0.036	0.926	±	0.021
207	0.913	±	0.099	1.007	±	0.055	0.901	±	0.080	0.818	±	0.027	0.756	±	0.014
208	1.405	±	0.272	1.384	±	0.128	1.183	±	0.187	0.956	±	0.063	0.907	±	0.038
209	1.385	±	0.178	1.205	±	0.084	1.243	±	0.132	1.048	±	0.038	0.904	±	0.025
210	1.291	±	0.142	1.284	±	0.069	1.242	±	0.101	0.926	±	0.031	0.841	±	0.019
211	1.389	±	0.295	1.329	±	0.134	1.298	±	0.196	1.053	±	0.053	0.889	±	0.032

212	1.897	±	0.269	1.146	±	0.092	1.456	±	0.183	1.056	±	0.051	0.893	±	0.029
213	1.486	±	0.131	1.309	±	0.069	1.139	±	0.088	1.024	±	0.029	0.931	±	0.017
214	1.400	±	0.191	1.372	±	0.096	1.301	±	0.132	1.004	±	0.045	0.917	±	0.026
215	1.594	±	0.268	1.299	±	0.215	1.103	±	0.159	1.050	±	0.050	0.961	±	0.031
216	1.383	±	0.235	1.296	±	0.122	1.301	±	0.165	1.029	±	0.054	0.903	±	0.033
217	1.473	±	0.158	1.404	±	0.085	1.182	±	0.112	1.090	±	0.037	0.961	±	0.021
218	1.587	±	0.236	1.209	±	0.096	1.287	±	0.184	0.976	±	0.063	0.888	±	0.040
219	1.332	±	0.321	1.351	±	0.225	1.497	±	0.350	1.019	±	0.134	0.947	±	0.097
220	1.419	±	0.179	1.386	±	0.100	1.323	±	0.147	1.080	±	0.053	0.875	±	0.033
221	1.234	±	0.188	1.240	±	0.123	1.194	±	0.170	0.874	±	0.064	0.852	±	0.036
222	1.425	±	0.135	1.262	±	0.077	1.177	±	0.105	1.054	±	0.039	0.895	±	0.021
223	1.318	±	0.610	0.885	±	0.213	1.194	±	0.651	0.893	±	0.089	0.860	±	0.062
224	1.547	±	0.169	1.267	±	0.291	1.339	±	0.155	1.068	±	0.062	0.960	±	0.034
225	1.446	±	0.477	0.722	±	0.071	0.916	±	0.384	1.003	±	0.135	1.019	±	0.120
226	1.256	±	0.196	1.301	±	0.098	1.044	±	0.141	1.062	±	0.045	0.910	±	0.028
227	1.455	±	0.394	1.351	±	0.125	1.029	±	0.288	0.994	±	0.084	0.897	±	0.065
228	1.453	±	0.137	1.251	±	0.068	1.258	±	0.111	1.008	±	0.035	0.891	±	0.023
229	1.660	±	0.348	1.754	±	0.130	1.336	±	0.169	1.073	±	0.049	0.970	±	0.031
230	1.701	±	0.210	1.469	±	0.096	1.433	±	0.153	1.048	±	0.052	0.974	±	0.035
231	1.409	±	0.151	1.311	±	0.076	1.248	±	0.110	1.083	±	0.037	0.902	±	0.024
232	1.598	±	0.119	1.336	±	0.058	1.226	±	0.084	1.093	±	0.028	0.890	±	0.016
233	1.636	±	0.182	1.446	±	0.088	1.377	±	0.127	1.208	±	0.041	1.069	±	0.027
234	1.209	±	0.089	1.205	±	0.045	1.380	±	0.140	1.202	±	0.036	0.954	±	0.022
235	1.255	±	0.118	1.382	±	0.059	1.202	±	0.082	1.029	±	0.026	0.888	±	0.015
236	1.478	±	0.131	1.364	±	0.063	1.343	±	0.089	1.082	±	0.029	0.945	±	0.018
237	1.410	±	0.240	1.389	±	0.129	1.257	±	0.207	1.024	±	0.060	0.879	±	0.036
238	1.587	±	0.206	1.297	±	0.113	1.199	±	0.165	1.087	±	0.052	0.945	±	0.031
239	1.130	±	0.169	1.274	±	0.086	1.191	±	0.122	0.920	±	0.040	0.839	±	0.021
240	1.385	±	0.143	1.243	±	0.080	1.180	±	0.116	0.993	±	0.039	0.892	±	0.022
241	1.005	±	0.376	1.332	±	0.160	1.349	±	0.323	1.031	±	0.114	0.910	±	0.086
242	1.270	±	0.193	1.331	±	0.086	1.444	±	0.163	1.027	±	0.055	0.900	±	0.037
243	1.190	±	0.183	1.438	±	0.091	1.359	±	0.129	1.116	±	0.042	0.951	±	0.026
244	1.550	±	0.300	1.442	±	0.133	1.498	±	0.231	1.105	±	0.073	0.985	±	0.048
245	1.742	±	0.279	1.360	±	0.166	1.186	±	0.218	1.085	±	0.075	0.959	±	0.046
246	1.528	±	0.280	1.377	±	0.165	1.401	±	0.238	1.108	±	0.108	0.935	±	0.065
247	1.600	±	0.187	1.592	±	0.105	1.438	±	0.134	1.178	±	0.041	1.037	±	0.028
248	1.677	±	0.360	1.433	±	0.255	1.379	±	0.368	0.996	±	0.127	1.005	±	0.082
249	1.415	±	0.160	1.333	±	0.086	1.216	±	0.116	0.985	±	0.044	0.943	±	0.027
250	1.463	±	0.154	1.410	±	0.081	1.265	±	0.118	1.039	±	0.043	0.920	±	0.026
251	1.303	±	0.187	0.713	±	0.105	1.216	±	0.227	1.557	±	0.093	1.005	±	0.045
252	1.366	±	0.219	1.489	±	0.193	1.251	±	0.170	1.056	±	0.068	0.954	±	0.038
253	1.891	±	0.297	2.020	±	0.299	1.186	±	0.257	1.445	±	0.084	0.958	±	0.042
254	1.234	±	0.168	1.167	±	0.102	0.974	±	0.140	1.000	±	0.061	0.953	±	0.034
255	1.105	±	0.073	1.110	±	0.044	1.066	±	0.065	1.082	±	0.026	1.000	±	0.015
256	0.938	±	0.066	0.993	±	0.032	1.019	±	0.052	0.955	±	0.019	0.963	±	0.011
257	0.683	±	0.051	0.764	±	0.025	0.816	±	0.045	0.880	±	0.015	0.914	±	0.010
258	0.489	±	0.043	0.538	±	0.018	0.568	±	0.035	0.683	±	0.013	0.761	±	0.008
259	0.253	±	0.013	0.289	±	0.007	0.319	±	0.010	0.449	±	0.005	0.481	±	0.002

Table S5: Transverse cross-correlated cross-relaxation rates η_{xy} (s^{-1})

residue	9.4 T	11.8 T	14.1 T	18.8 T	23.5 T
145	0.37 ± 0.05	0.44 ± 0.04	0.45 ± 0.07	0.77 ± 0.02	0.93 ± 0.02
146	0.54 ± 0.04	0.58 ± 0.03	0.75 ± 0.05	0.94 ± 0.02	1.18 ± 0.02
147	0.61 ± 0.04	0.77 ± 0.04	0.83 ± 0.05	1.11 ± 0.02	1.33 ± 0.02
148	0.63 ± 0.05	0.90 ± 0.05	0.93 ± 0.07	1.13 ± 0.02	1.42 ± 0.02
149	0.69 ± 0.04	0.81 ± 0.04	1.04 ± 0.06	1.30 ± 0.02	1.58 ± 0.02
150	0.75 ± 0.05	0.88 ± 0.05	0.97 ± 0.08	1.30 ± 0.03	1.56 ± 0.03
151	0.83 ± 0.08	0.85 ± 0.06	1.01 ± 0.11	1.24 ± 0.04	1.59 ± 0.03
152	0.78 ± 0.05	1.01 ± 0.04	1.16 ± 0.08	1.36 ± 0.02	1.74 ± 0.03
153	0.83 ± 0.05	0.92 ± 0.04	1.18 ± 0.07	1.34 ± 0.02	1.67 ± 0.02
154	0.63 ± 0.08	0.52 ± 0.08	1.16 ± 0.06	1.48 ± 0.02	1.81 ± 0.02
155	0.79 ± 0.06	1.22 ± 0.06	1.12 ± 0.10	1.46 ± 0.03	1.75 ± 0.03
156	0.98 ± 0.06	1.14 ± 0.07	1.34 ± 0.08	1.58 ± 0.03	1.99 ± 0.03
157	0.94 ± 0.06	1.03 ± 0.05	1.23 ± 0.08	1.56 ± 0.03	1.81 ± 0.03
158	0.74 ± 0.04	0.96 ± 0.04	1.09 ± 0.06	1.38 ± 0.02	1.69 ± 0.02
159	0.58 ± 0.05	0.76 ± 0.05	0.87 ± 0.09	1.11 ± 0.03	1.28 ± 0.03
160	0.68 ± 0.07	0.90 ± 0.05	0.99 ± 0.09	1.37 ± 0.03	1.64 ± 0.03
161	0.58 ± 0.13	1.11 ± 0.10	0.91 ± 0.17	1.47 ± 0.05	1.60 ± 0.04
162	0.78 ± 0.06	1.05 ± 0.12	1.20 ± 0.09	1.47 ± 0.03	1.74 ± 0.03
163	0.73 ± 0.06	0.88 ± 0.05	1.11 ± 0.10	1.31 ± 0.04	1.60 ± 0.03
164	0.98 ± 0.08	1.07 ± 0.06	1.40 ± 0.12	1.71 ± 0.04	2.16 ± 0.03
165	1.16 ± 0.08	1.31 ± 0.08	1.60 ± 0.12	2.17 ± 0.05	2.54 ± 0.04
167	1.57 ± 0.13	1.81 ± 0.11	2.30 ± 0.20	2.81 ± 0.07	3.34 ± 0.05
168	1.76 ± 0.17	2.00 ± 0.14	2.54 ± 0.25	3.30 ± 0.09	4.05 ± 0.07
169	1.86 ± 0.19	2.19 ± 0.16	2.60 ± 0.31	3.79 ± 0.12	4.54 ± 0.10
171	2.49 ± 0.23	2.52 ± 0.19	3.81 ± 0.41	4.53 ± 0.17	5.37 ± 0.13
172	2.76 ± 0.35	2.69 ± 0.26	3.15 ± 0.60	4.94 ± 0.29	5.82 ± 0.22
173	2.83 ± 0.33	3.27 ± 0.27	4.15 ± 0.64	5.00 ± 0.26	6.03 ± 0.21
174	1.91 ± 0.50	2.70 ± 0.46	3.71 ± 0.95	5.20 ± 0.47	5.70 ± 0.39
175	2.59 ± 0.44	2.22 ± 0.26	3.25 ± 0.77	3.87 ± 0.27	4.81 ± 0.20
176	1.95 ± 0.91	1.67 ± 0.34	3.06 ± 0.96	3.76 ± 0.29	4.25 ± 0.20
177	2.19 ± 0.32	2.30 ± 0.20	2.95 ± 0.46	3.63 ± 0.15	4.36 ± 0.12
178	2.14 ± 0.23	2.03 ± 0.17	2.52 ± 0.39	3.27 ± 0.12	3.95 ± 0.09
179	1.57 ± 0.21	1.96 ± 0.17	1.87 ± 0.28	2.98 ± 0.10	3.59 ± 0.08
180	1.57 ± 0.10	2.17 ± 0.10	2.25 ± 0.17	2.79 ± 0.06	3.31 ± 0.05
181	1.25 ± 0.07	2.93 ± 0.11	1.79 ± 0.13	2.52 ± 0.04	3.04 ± 0.03
183	1.17 ± 0.16	1.33 ± 0.12	1.74 ± 0.26	2.16 ± 0.08	2.46 ± 0.06
184	1.05 ± 0.31	1.14 ± 0.17	1.38 ± 0.43	1.83 ± 0.12	2.28 ± 0.08
185	0.76 ± 0.09	0.93 ± 0.07	0.98 ± 0.15	1.40 ± 0.05	1.72 ± 0.04
187	1.18 ± 0.17	1.29 ± 0.11	1.72 ± 0.25	1.97 ± 0.07	2.41 ± 0.06
188	1.15 ± 0.21	1.48 ± 0.18	1.82 ± 0.29	1.99 ± 0.09	2.42 ± 0.06
189	0.95 ± 0.32	1.50 ± 0.23	2.09 ± 0.39	2.32 ± 0.10	2.75 ± 0.08
190	1.36 ± 0.12	1.62 ± 0.12	1.50 ± 0.17	2.22 ± 0.06	2.60 ± 0.05
192	1.30 ± 0.09	1.50 ± 0.07	1.71 ± 0.11	2.30 ± 0.04	2.76 ± 0.03
193	1.39 ± 0.11	1.40 ± 0.09	1.69 ± 0.15	2.11 ± 0.05	2.66 ± 0.04
194	1.31 ± 0.13	1.53 ± 0.10	1.75 ± 0.18	2.41 ± 0.05	2.90 ± 0.05
195	1.27 ± 0.32	1.53 ± 0.16	1.58 ± 0.48	2.24 ± 0.10	2.73 ± 0.08
197	1.25 ± 0.13	1.52 ± 0.09	1.88 ± 0.19	2.46 ± 0.06	2.86 ± 0.04
198	1.31 ± 0.12	1.46 ± 0.09	1.81 ± 0.17	2.35 ± 0.06	2.72 ± 0.05
199	1.25 ± 0.06	1.51 ± 0.05	1.76 ± 0.09	2.21 ± 0.03	2.72 ± 0.03
200	1.22 ± 0.04	1.73 ± 0.07	2.17 ± 0.13	2.42 ± 0.03	2.90 ± 0.03
201	1.34 ± 0.08	2.12 ± 0.10	1.61 ± 0.12	2.19 ± 0.04	2.68 ± 0.03
202	1.53 ± 0.08	2.12 ± 0.07	1.97 ± 0.10	2.52 ± 0.03	3.18 ± 0.03
204	1.64 ± 0.11	1.94 ± 0.08	2.41 ± 0.16	3.02 ± 0.05	3.45 ± 0.04
205	1.82 ± 0.20	1.93 ± 0.12	2.37 ± 0.25	3.02 ± 0.08	3.72 ± 0.07
206	2.10 ± 0.16	2.46 ± 0.14	3.01 ± 0.24	3.72 ± 0.07	4.56 ± 0.06
207	2.79 ± 0.21	3.27 ± 0.17	3.84 ± 0.27	4.93 ± 0.07	6.01 ± 0.06
208	3.98 ± 0.48	4.47 ± 0.35	5.59 ± 0.63	6.78 ± 0.16	8.17 ± 0.14
209	4.52 ± 0.38	5.11 ± 0.27	5.86 ± 0.44	7.91 ± 0.11	9.82 ± 0.12
210	4.64 ± 0.33	4.95 ± 0.23	6.11 ± 0.39	7.92 ± 0.10	9.88 ± 0.10
211	4.58 ± 0.70	4.84 ± 0.44	6.19 ± 0.71	7.75 ± 0.16	9.41 ± 0.13

212	4.54	±	0.50	3.33	±	0.22	6.24	±	0.56	8.85	±	0.17	9.51	±	0.13
213	4.53	±	0.29	5.20	±	0.22	6.19	±	0.34	8.02	±	0.09	9.96	±	0.09
214	4.86	±	0.49	5.63	±	0.35	6.52	±	0.56	8.77	±	0.15	10.53	±	0.13
215	5.41	±	0.81	5.26	±	0.69	6.65	±	0.69	8.21	±	0.16	10.00	±	0.14
216	4.80	±	0.55	4.84	±	0.38	6.16	±	0.64	8.39	±	0.18	9.80	±	0.16
217	4.69	±	0.39	5.60	±	0.32	7.06	±	0.53	8.53	±	0.13	10.62	±	0.12
218	4.61	±	0.60	4.22	±	0.33	6.37	±	0.74	7.95	±	0.20	9.85	±	0.20
219	5.02	±	0.81	5.40	±	0.76	7.58	±	1.45	8.13	±	0.49	9.75	±	0.50
220	3.89	±	0.41	4.91	±	0.39	5.44	±	0.65	7.60	±	0.23	9.27	±	0.22
221	4.46	±	0.48	4.91	±	0.47	5.85	±	0.80	6.96	±	0.26	8.76	±	0.24
222	4.06	±	0.31	4.75	±	0.28	5.57	±	0.46	7.35	±	0.14	8.73	±	0.13
223	3.53	±	0.90	1.80	±	0.40	5.54	±	0.49	7.49	±	0.33	8.05	±	0.29
224	4.21	±	0.44	4.09	±	0.86	5.17	±	0.67	7.12	±	0.26	8.87	±	0.24
225	3.71	±	0.91	1.03	±	0.14	7.21	±	2.02	5.25	±	0.40	9.26	±	0.71
226	3.11	±	0.33	3.78	±	0.26	4.09	±	0.40	5.40	±	0.12	6.70	±	0.11
227	4.11	±	0.59	4.76	±	0.32	6.00	±	0.78	7.29	±	0.20	9.23	±	0.21
228	4.59	±	0.33	4.81	±	0.22	5.69	±	0.42	7.28	±	0.11	9.13	±	0.11
229	5.68	±	0.78	5.38	±	0.39	5.59	±	0.56	7.56	±	0.14	9.51	±	0.14
230	4.69	±	0.40	5.10	±	0.29	5.98	±	0.51	7.76	±	0.15	9.35	±	0.15
231	4.66	±	0.37	4.96	±	0.24	5.97	±	0.43	7.73	±	0.12	9.82	±	0.12
232	4.81	±	0.28	5.02	±	0.19	6.14	±	0.34	7.60	±	0.09	9.63	±	0.09
233	5.01	±	0.45	5.04	±	0.28	6.40	±	0.48	7.85	±	0.13	9.49	±	0.13
234	2.87	±	0.16	2.75	±	0.12	6.29	±	0.46	7.85	±	0.11	9.72	±	0.11
235	4.49	±	0.27	5.00	±	0.19	5.97	±	0.32	7.61	±	0.08	9.42	±	0.08
236	4.12	±	0.27	4.82	±	0.21	5.90	±	0.33	7.24	±	0.09	9.04	±	0.09
237	4.04	±	0.60	4.47	±	0.43	5.00	±	0.71	6.68	±	0.19	8.29	±	0.17
238	4.70	±	0.42	5.12	±	0.34	6.18	±	0.59	7.76	±	0.16	9.78	±	0.16
239	3.66	±	0.41	4.48	±	0.30	5.37	±	0.51	7.09	±	0.13	8.48	±	0.11
240	3.53	±	0.25	3.98	±	0.22	4.64	±	0.36	6.05	±	0.10	7.48	±	0.10
241	4.21	±	0.67	4.53	±	0.45	5.52	±	1.03	7.13	±	0.33	8.85	±	0.34
242	4.41	±	0.40	4.69	±	0.26	6.07	±	0.54	7.42	±	0.16	9.28	±	0.16
243	4.42	±	0.40	4.66	±	0.28	5.86	±	0.49	7.20	±	0.12	8.89	±	0.11
244	4.18	±	0.50	4.65	±	0.34	5.36	±	0.63	7.35	±	0.20	8.85	±	0.19
245	4.47	±	0.59	5.68	±	0.54	6.90	±	0.94	7.96	±	0.23	9.79	±	0.21
246	4.48	±	0.58	4.97	±	0.49	5.56	±	0.79	7.72	±	0.33	9.66	±	0.30
247	4.88	±	0.40	5.45	±	0.32	6.17	±	0.46	7.83	±	0.12	9.89	±	0.13
248	3.90	±	0.79	4.02	±	0.66	6.09	±	1.16	7.49	±	0.38	9.10	±	0.35
249	3.77	±	0.31	4.84	±	0.27	5.83	±	0.46	7.30	±	0.14	9.24	±	0.14
250	4.21	±	0.35	4.44	±	0.25	5.89	±	0.45	7.56	±	0.14	8.83	±	0.12
251	4.17	±	0.38	1.91	±	0.31	5.68	±	0.79	7.61	±	0.21	8.46	±	0.18
252	3.93	±	0.46	4.70	±	0.68	5.21	±	0.68	6.87	±	0.22	8.54	±	0.19
253	4.27	±	0.78	4.85	±	0.93	6.02	±	0.88	8.34	±	0.37	8.07	±	0.24
254	3.07	±	0.29	3.33	±	0.29	4.04	±	0.46	5.33	±	0.18	6.16	±	0.16
255	2.55	±	0.12	2.79	±	0.11	3.34	±	0.19	4.13	±	0.07	4.99	±	0.06
256	1.85	±	0.09	2.11	±	0.07	2.70	±	0.14	3.17	±	0.04	3.91	±	0.04
257	1.17	±	0.07	1.34	±	0.05	1.62	±	0.11	2.00	±	0.03	2.47	±	0.03
258	0.67	±	0.05	0.88	±	0.04	0.98	±	0.07	1.30	±	0.02	1.59	±	0.02
259	0.32	±	0.02	0.38	±	0.02	0.42	±	0.03	0.60	±	0.01	0.74	±	0.01

Table S6: $^{15}N-\{^{1}H\}$ nuclear Overhauser effects

residue	9.4 T			11.8 T			14.1 T			18.8 T			23.5 T		
145	-2.072	±	0.020	-1.828	±	0.029	-1.401	±	0.014	-0.780	±	0.011	-0.322	±	0.006
146	-1.854	±	0.013	-1.550	±	0.025	-1.195	±	0.013	-0.524	±	0.009	-0.102	±	0.006
147	-1.855	±	0.024	-1.360	±	0.028	-1.029	±	0.014	-0.400	±	0.010	0.016	±	0.006
148	-1.590	±	0.019	-1.172	±	0.024	-0.829	±	0.012	-0.284	±	0.010	0.088	±	0.006
149	-1.330	±	0.021	-0.960	±	0.024	-0.654	±	0.015	-0.126	±	0.010	0.138	±	0.006
150	-1.200	±	0.013	-0.842	±	0.021	-0.587	±	0.014	-0.092	±	0.009	0.176	±	0.007
151	-1.153	±	0.014	-0.827	±	0.020	-0.504	±	0.010	-0.040	±	0.009	0.205	±	0.006
152	-1.034	±	0.011	-0.565	±	0.014	-0.435	±	0.008	0.025	±	0.008	0.289	±	0.006
153	-1.060	±	0.011	-0.714	±	0.016	-0.458	±	0.008	0.012	±	0.008	0.244	±	0.005
154	-1.478	±	0.018	-0.631	±	0.034	-0.471	±	0.008	0.027	±	0.007	0.259	±	0.005
155	-0.986	±	0.013	-0.611	±	0.018	-0.409	±	0.010	0.029	±	0.008	0.302	±	0.006
156	-0.921	±	0.008	-0.665	±	0.026	-0.351	±	0.007	0.090	±	0.007	0.322	±	0.005
157	-1.070	±	0.009	-0.681	±	0.019	-0.408	±	0.010	0.035	±	0.009	0.299	±	0.007
158	-1.225	±	0.014	-0.760	±	0.020	-0.488	±	0.010	-0.017	±	0.008	0.239	±	0.006
159	-1.204	±	0.014	-0.875	±	0.023	-0.535	±	0.011	-0.112	±	0.010	0.177	±	0.007
160	-1.176	±	0.009	-0.711	±	0.016	-0.508	±	0.008	-0.010	±	0.007	0.248	±	0.005
161	-1.126	±	0.007	-0.711	±	0.021	-0.467	±	0.012	-0.070	±	0.011	0.228	±	0.007
162	-1.032	±	0.017	-0.504	±	0.025	-0.422	±	0.010	0.010	±	0.009	0.241	±	0.006
163	-0.926	±	0.012	-0.678	±	0.022	-0.394	±	0.012	0.007	±	0.012	0.256	±	0.009
164	-0.950	±	0.019	-0.617	±	0.022	-0.338	±	0.012	0.055	±	0.010	0.307	±	0.008
165	-0.753	±	0.018	-0.520	±	0.032	-0.291	±	0.020	0.062	±	0.014	0.266	±	0.010
167	-0.515	±	0.020	-0.294	±	0.035	-0.092	±	0.025	0.190	±	0.022	0.386	±	0.016
168	-0.328	±	0.030	-0.224	±	0.039	-0.042	±	0.031	0.250	±	0.023	0.428	±	0.020
169	-0.333	±	0.030	-0.081	±	0.039	0.142	±	0.036	0.315	±	0.028	0.453	±	0.024
171	-0.153	±	0.032	0.076	±	0.039	0.260	±	0.040	0.491	±	0.034	0.526	±	0.028
172	0.038	±	0.034	0.252	±	0.052	0.235	±	0.053	0.548	±	0.048	0.559	±	0.035
173	0.210	±	0.034	0.564	±	0.067	0.246	±	0.056	0.512	±	0.048	0.629	±	0.043
174	-0.104	±	0.020	0.214	±	0.096	0.166	±	0.078	0.506	±	0.069	0.613	±	0.057
175	-0.191	±	0.039	0.051	±	0.050	0.228	±	0.047	0.396	±	0.036	0.486	±	0.032
176	-0.121	±	0.033	-0.086	±	0.045	0.171	±	0.040	0.390	±	0.032	0.568	±	0.025
177	-0.167	±	0.047	-0.026	±	0.043	0.100	±	0.031	0.450	±	0.030	0.523	±	0.021
178	-0.281	±	0.017	0.074	±	0.042	0.118	±	0.028	0.329	±	0.023	0.532	±	0.016
179	-0.313	±	0.018	-0.123	±	0.036	0.085	±	0.019	0.330	±	0.018	0.519	±	0.014
180	-0.351	±	0.012	-0.249	±	0.022	0.074	±	0.017	0.343	±	0.015	0.488	±	0.012
181	-0.817	±	0.025	0.179	±	0.020	-0.056	±	0.014	0.273	±	0.013	0.429	±	0.010
183	-0.658	±	0.019	-0.387	±	0.027	-0.124	±	0.015	0.183	±	0.015	0.411	±	0.010
184	-0.614	±	0.012	-0.432	±	0.028	-0.195	±	0.018	0.128	±	0.017	0.358	±	0.014
185	-0.771	±	0.013	-0.503	±	0.022	-0.268	±	0.012	0.050	±	0.011	0.283	±	0.008
187	-0.496	±	0.019	-0.374	±	0.026	-0.173	±	0.016	0.153	±	0.015	0.348	±	0.011
188	-0.565	±	0.020	-0.302	±	0.026	-0.097	±	0.015	0.209	±	0.016	0.387	±	0.011
189	-0.620	±	0.018	-0.347	±	0.032	-0.043	±	0.020	0.243	±	0.016	0.401	±	0.013
190	-0.534	±	0.016	-0.279	±	0.033	-0.105	±	0.016	0.205	±	0.015	0.380	±	0.012
192	-0.553	±	0.011	-0.430	±	0.021	-0.167	±	0.011	0.162	±	0.011	0.392	±	0.008
193	-0.605	±	0.016	-0.390	±	0.028	-0.125	±	0.015	0.197	±	0.013	0.365	±	0.010
194	-0.623	±	0.014	-0.363	±	0.026	-0.161	±	0.015	0.183	±	0.013	0.430	±	0.010
195	-0.594	±	0.016	-0.391	±	0.023	-0.163	±	0.013	0.165	±	0.014	0.362	±	0.011
197	-0.465	±	0.006	-0.307	±	0.019	-0.090	±	0.012	0.189	±	0.012	0.371	±	0.009
198	-0.456	±	0.008	-0.289	±	0.023	-0.090	±	0.013	0.235	±	0.014	0.400	±	0.012
199	-0.557	±	0.011	-0.347	±	0.018	-0.115	±	0.009	0.196	±	0.009	0.385	±	0.007
200	-0.762	±	0.012	-0.138	±	0.014	-0.071	±	0.009	0.148	±	0.009	0.433	±	0.008
201	-0.483	±	0.014	-0.156	±	0.022	-0.057	±	0.010	0.194	±	0.012	0.363	±	0.009
202	-0.339	±	0.011	-0.216	±	0.017	-0.024	±	0.010	0.235	±	0.011	0.424	±	0.009
204	-0.272	±	0.015	-0.096	±	0.020	0.094	±	0.011	0.282	±	0.011	0.516	±	0.009
205	0.246	±	0.007	-0.028	±	0.024	0.102	±	0.017	0.373	±	0.016	0.494	±	0.013
206	-0.062	±	0.021	0.105	±	0.027	0.246	±	0.017	0.420	±	0.016	0.518	±	0.012
207	0.344	±	0.015	0.448	±	0.029	0.448	±	0.016	0.549	±	0.016	0.600	±	0.014
208	0.631	±	0.017	0.652	±	0.039	0.656	±	0.022	0.876	±	0.029	0.810	±	0.026
209	0.683	±	0.024	0.708	±	0.031	0.722	±	0.017	0.812	±	0.020	0.850	±	0.020
210	0.555	±	0.011	0.690	±	0.031	0.718	±	0.017	0.763	±	0.017	0.876	±	0.017
211	0.661	±	0.029	0.718	±	0.037	0.686	±	0.019	0.801	±	0.024	0.801	±	0.016

212	0.595	±	0.025	0.771	±	0.046	0.693	±	0.022	0.825	±	0.025	0.823	±	0.020
213	0.520	±	0.012	0.651	±	0.030	0.697	±	0.016	0.740	±	0.018	0.793	±	0.015
214	0.601	±	0.024	0.606	±	0.035	0.713	±	0.022	0.790	±	0.023	0.779	±	0.018
215	0.578	±	0.016	0.637	±	0.046	0.713	±	0.021	0.768	±	0.022	0.840	±	0.020
216	0.686	±	0.032	0.659	±	0.045	0.733	±	0.027	0.773	±	0.028	0.815	±	0.026
217	0.551	±	0.014	0.624	±	0.038	0.744	±	0.024	0.860	±	0.025	0.813	±	0.018
218	0.555	±	0.018	0.689	±	0.040	0.704	±	0.025	0.782	±	0.027	0.764	±	0.022
219	0.718	±	0.042	0.615	±	0.065	0.685	±	0.057	0.764	±	0.052	0.757	±	0.047
220	0.585	±	0.028	0.657	±	0.044	0.735	±	0.028	0.766	±	0.031	0.810	±	0.028
221	0.552	±	0.028	0.605	±	0.050	0.695	±	0.037	0.776	±	0.036	0.801	±	0.029
222	0.591	±	0.027	0.589	±	0.031	0.675	±	0.020	0.702	±	0.020	0.797	±	0.018
223	0.431	±	0.038	-0.147	±	0.057	0.692	±	0.031	0.718	±	0.036	0.770	±	0.034
224	0.475	±	0.004	0.734	±	0.145	0.701	±	0.033	0.761	±	0.033	0.828	±	0.037
225	0.393	±	0.040	-0.175	±	0.031	0.603	±	0.031	0.782	±	0.051	0.677	±	0.055
226	0.436	±	0.014	0.641	±	0.035	0.644	±	0.020	0.686	±	0.021	0.780	±	0.019
227	0.551	±	0.013	0.603	±	0.030	0.740	±	0.020	0.729	±	0.022	0.817	±	0.026
228	0.531	±	0.019	0.654	±	0.032	0.677	±	0.018	0.802	±	0.019	0.842	±	0.016
229	0.809	±	0.034	0.737	±	0.037	0.717	±	0.021	0.693	±	0.020	0.810	±	0.017
230	0.602	±	0.032	0.621	±	0.033	0.747	±	0.022	0.805	±	0.026	0.823	±	0.021
231	0.488	±	0.015	0.632	±	0.029	0.709	±	0.019	0.776	±	0.021	0.871	±	0.021
232	0.689	±	0.030	0.634	±	0.027	0.763	±	0.017	0.780	±	0.018	0.893	±	0.015
233	0.647	±	0.015	0.631	±	0.032	0.728	±	0.020	0.836	±	0.023	0.758	±	0.021
234	0.367	±	0.007	0.157	±	0.024	0.764	±	0.021	0.822	±	0.022	0.827	±	0.019
235	0.552	±	0.010	0.665	±	0.027	0.700	±	0.015	0.791	±	0.018	0.806	±	0.013
236	0.585	±	0.013	0.663	±	0.027	0.727	±	0.017	0.809	±	0.019	0.806	±	0.014
237	0.586	±	0.017	0.630	±	0.045	0.668	±	0.028	0.738	±	0.028	0.836	±	0.027
238	0.567	±	0.016	0.627	±	0.039	0.690	±	0.026	0.788	±	0.029	0.834	±	0.025
239	0.491	±	0.018	0.621	±	0.035	0.727	±	0.025	0.755	±	0.024	0.808	±	0.018
240	0.510	±	0.020	0.630	±	0.038	0.609	±	0.023	0.660	±	0.021	0.819	±	0.021
241	0.567	±	0.015	0.574	±	0.047	0.749	±	0.037	0.805	±	0.039	0.808	±	0.039
242	0.550	±	0.022	0.663	±	0.033	0.697	±	0.021	0.738	±	0.021	0.797	±	0.025
243	0.541	±	0.018	0.673	±	0.034	0.738	±	0.022	0.820	±	0.024	0.807	±	0.018
244	0.484	±	0.024	0.647	±	0.045	0.652	±	0.024	0.708	±	0.030	0.733	±	0.025
245	0.701	±	0.031	0.658	±	0.054	0.749	±	0.038	0.827	±	0.036	0.820	±	0.033
246	0.576	±	0.022	0.719	±	0.066	0.680	±	0.046	0.751	±	0.049	0.765	±	0.045
247	0.682	±	0.021	0.643	±	0.031	0.717	±	0.019	0.851	±	0.022	0.884	±	0.020
248	0.724	±	0.068	0.598	±	0.059	0.664	±	0.039	0.803	±	0.042	0.709	±	0.049
249	0.586	±	0.016	0.684	±	0.036	0.762	±	0.024	0.883	±	0.027	0.792	±	0.021
250	0.549	±	0.018	0.654	±	0.033	0.668	±	0.020	0.788	±	0.025	0.745	±	0.018
251	0.596	±	0.041	0.300	±	0.034	0.724	±	0.036	0.800	±	0.034	0.827	±	0.031
252	0.513	±	0.031	0.597	±	0.080	0.613	±	0.033	0.766	±	0.034	0.728	±	0.031
253	0.776	±	0.251	0.570	±	0.100	0.558	±	0.057	0.628	±	0.054	0.726	±	0.036
254	0.310	±	0.029	0.503	±	0.063	0.509	±	0.036	0.507	±	0.033	0.620	±	0.033
255	0.127	±	0.014	0.223	±	0.029	0.337	±	0.019	0.517	±	0.020	0.569	±	0.015
256	-0.134	±	0.013	0.015	±	0.020	0.161	±	0.012	0.360	±	0.014	0.504	±	0.011
257	-0.592	±	0.010	-0.382	±	0.017	-0.125	±	0.009	0.100	±	0.010	0.320	±	0.008
258	-1.118	±	0.007	-0.962	±	0.015	-0.683	±	0.007	-0.250	±	0.007	0.049	±	0.005
259	-2.011	±	0.017	-1.674	±	0.018	-1.330	±	0.009	-0.702	±	0.007	-0.316	±	0.004

11. Spectral density mapping results:

Table S7: J(0.87ω_H) (ns) derived at five magnetic fields

residue	9.4 T			11.8 T			14.1 T			18.8 T			23.5 T		
145	0.0515	±	0.0005	0.0459	±	0.0006	0.0372	±	0.0005	0.0284	±	0.0002	0.0230	±	0.0001
146	0.0549	±	0.0004	0.0485	±	0.0005	0.0388	±	0.0005	0.0273	±	0.0002	0.0221	±	0.0001
147	0.0605	±	0.0006	0.0460	±	0.0006	0.0409	±	0.0005	0.0275	±	0.0002	0.0212	±	0.0001
148	0.0566	±	0.0006	0.0442	±	0.0005	0.0397	±	0.0005	0.0263	±	0.0002	0.0199	±	0.0001
149	0.0529	±	0.0006	0.0420	±	0.0006	0.0359	±	0.0005	0.0239	±	0.0002	0.0193	±	0.0001
150	0.0525	±	0.0005	0.0423	±	0.0005	0.0364	±	0.0006	0.0244	±	0.0002	0.0194	±	0.0002
151	0.0521	±	0.0005	0.0424	±	0.0005	0.0334	±	0.0007	0.0217	±	0.0002	0.0189	±	0.0002
152	0.0510	±	0.0004	0.0383	±	0.0004	0.0349	±	0.0005	0.0230	±	0.0002	0.0180	±	0.0001
153	0.0529	±	0.0004	0.0401	±	0.0004	0.0338	±	0.0005	0.0221	±	0.0002	0.0182	±	0.0001
154	0.0629	±	0.0006	0.0432	±	0.0017	0.0354	±	0.0004	0.0223	±	0.0002	0.0187	±	0.0001
155	0.0526	±	0.0006	0.0385	±	0.0005	0.0320	±	0.0005	0.0226	±	0.0002	0.0173	±	0.0002
156	0.0525	±	0.0004	0.0425	±	0.0008	0.0335	±	0.0005	0.0226	±	0.0002	0.0178	±	0.0001
157	0.0551	±	0.0004	0.0414	±	0.0005	0.0353	±	0.0006	0.0234	±	0.0002	0.0178	±	0.0002
158	0.0535	±	0.0005	0.0409	±	0.0005	0.0355	±	0.0004	0.0233	±	0.0002	0.0182	±	0.0001
159	0.0527	±	0.0005	0.0426	±	0.0006	0.0321	±	0.0006	0.0246	±	0.0002	0.0183	±	0.0002
160	0.0507	±	0.0004	0.0373	±	0.0004	0.0331	±	0.0005	0.0225	±	0.0002	0.0174	±	0.0001
161	0.0506	±	0.0007	0.0394	±	0.0006	0.0335	±	0.0009	0.0231	±	0.0003	0.0182	±	0.0002
162	0.0498	±	0.0006	0.0356	±	0.0007	0.0336	±	0.0005	0.0228	±	0.0002	0.0181	±	0.0002
163	0.0473	±	0.0005	0.0393	±	0.0006	0.0308	±	0.0006	0.0233	±	0.0003	0.0169	±	0.0002
164	0.0512	±	0.0007	0.0393	±	0.0006	0.0324	±	0.0007	0.0220	±	0.0003	0.0168	±	0.0002
165	0.0449	±	0.0006	0.0354	±	0.0008	0.0307	±	0.0008	0.0211	±	0.0003	0.0168	±	0.0002
167	0.0463	±	0.0008	0.0357	±	0.0011	0.0278	±	0.0009	0.0195	±	0.0005	0.0151	±	0.0004
168	0.0419	±	0.0012	0.0361	±	0.0012	0.0285	±	0.0012	0.0190	±	0.0006	0.0140	±	0.0005
169	0.0425	±	0.0011	0.0318	±	0.0013	0.0225	±	0.0012	0.0167	±	0.0006	0.0131	±	0.0006
171	0.0404	±	0.0012	0.0293	±	0.0012	0.0206	±	0.0014	0.0131	±	0.0009	0.0122	±	0.0007
172	0.0339	±	0.0013	0.0245	±	0.0017	0.0232	±	0.0020	0.0120	±	0.0013	0.0113	±	0.0009
173	0.0260	±	0.0013	0.0129	±	0.0020	0.0205	±	0.0019	0.0138	±	0.0013	0.0104	±	0.0012
174	0.0407	±	0.0011	0.0253	±	0.0031	0.0258	±	0.0029	0.0137	±	0.0019	0.0105	±	0.0015
175	0.0429	±	0.0016	0.0293	±	0.0016	0.0251	±	0.0021	0.0161	±	0.0010	0.0132	±	0.0008
176	0.0417	±	0.0034	0.0297	±	0.0016	0.0215	±	0.0018	0.0157	±	0.0009	0.0107	±	0.0006
177	0.0389	±	0.0017	0.0317	±	0.0013	0.0257	±	0.0016	0.0150	±	0.0009	0.0129	±	0.0006
178	0.0408	±	0.0009	0.0284	±	0.0014	0.0271	±	0.0013	0.0179	±	0.0006	0.0127	±	0.0004
179	0.0421	±	0.0009	0.0335	±	0.0012	0.0274	±	0.0011	0.0173	±	0.0005	0.0122	±	0.0004
180	0.0436	±	0.0006	0.0345	±	0.0007	0.0262	±	0.0008	0.0174	±	0.0004	0.0139	±	0.0003
181	0.0556	±	0.0009	0.0283	±	0.0007	0.0235	±	0.0005	0.0185	±	0.0003	0.0143	±	0.0003
183	0.0474	±	0.0009	0.0385	±	0.0009	0.0306	±	0.0011	0.0192	±	0.0004	0.0147	±	0.0003
184	0.0425	±	0.0010	0.0369	±	0.0009	0.0280	±	0.0013	0.0198	±	0.0004	0.0150	±	0.0004
185	0.0450	±	0.0006	0.0358	±	0.0006	0.0290	±	0.0008	0.0211	±	0.0003	0.0165	±	0.0002
187	0.0413	±	0.0008	0.0374	±	0.0008	0.0307	±	0.0010	0.0200	±	0.0004	0.0158	±	0.0003
188	0.0451	±	0.0009	0.0369	±	0.0009	0.0289	±	0.0012	0.0196	±	0.0004	0.0152	±	0.0003
189	0.0522	±	0.0013	0.0390	±	0.0013	0.0288	±	0.0013	0.0187	±	0.0004	0.0154	±	0.0004
190	0.0463	±	0.0007	0.0362	±	0.0010	0.0296	±	0.0009	0.0202	±	0.0004	0.0160	±	0.0003
192	0.0470	±	0.0005	0.0348	±	0.0006	0.0320	±	0.0006	0.0206	±	0.0003	0.0148	±	0.0002
193	0.0476	±	0.0007	0.0362	±	0.0009	0.0293	±	0.0008	0.0202	±	0.0003	0.0159	±	0.0003
194	0.0484	±	0.0007	0.0368	±	0.0008	0.0312	±	0.0009	0.0198	±	0.0003	0.0141	±	0.0002
195	0.0442	±	0.0010	0.0372	±	0.0008	0.0284	±	0.0013	0.0193	±	0.0003	0.0152	±	0.0003
197	0.0448	±	0.0006	0.0346	±	0.0006	0.0282	±	0.0008	0.0200	±	0.0003	0.0157	±	0.0002
198	0.0437	±	0.0005	0.0367	±	0.0007	0.0300	±	0.0008	0.0185	±	0.0004	0.0146	±	0.0003
199	0.0475	±	0.0005	0.0369	±	0.0005	0.0288	±	0.0005	0.0195	±	0.0002	0.0152	±	0.0002
200	0.0502	±	0.0004	0.0286	±	0.0004	0.0282	±	0.0005	0.0208	±	0.0002	0.0141	±	0.0002
201	0.0447	±	0.0006	0.0355	±	0.0007	0.0270	±	0.0005	0.0190	±	0.0003	0.0154	±	0.0002
202	0.0423	±	0.0005	0.0332	±	0.0005	0.0263	±	0.0005	0.0191	±	0.0003	0.0142	±	0.0002
204	0.0423	±	0.0006	0.0328	±	0.0007	0.0247	±	0.0006	0.0188	±	0.0003	0.0122	±	0.0002
205	0.0247	±	0.0004	0.0300	±	0.0008	0.0249	±	0.0009	0.0160	±	0.0004	0.0120	±	0.0003
206	0.0352	±	0.0008	0.0268	±	0.0008	0.0199	±	0.0007	0.0138	±	0.0004	0.0112	±	0.0003
207	0.0232	±	0.0006	0.0161	±	0.0008	0.0133	±	0.0005	0.0091	±	0.0003	0.0074	±	0.0003
208	0.0166	±	0.0008	0.0131	±	0.0015	0.0104	±	0.0008	0.0030	±	0.0007	0.0040	±	0.0005
209	0.0140	±	0.0011	0.0107	±	0.0012	0.0088	±	0.0006	0.0045	±	0.0005	0.0031	±	0.0004

210	0.0198	±	0.0005	0.0110	±	0.0011	0.0081	±	0.0006	0.0055	±	0.0004	0.0025	±	0.0003
211	0.0145	±	0.0014	0.0105	±	0.0014	0.0097	±	0.0007	0.0046	±	0.0006	0.0040	±	0.0003
212	0.0182	±	0.0012	0.0080	±	0.0017	0.0095	±	0.0007	0.0040	±	0.0006	0.0037	±	0.0004
213	0.0224	±	0.0006	0.0134	±	0.0011	0.0095	±	0.0006	0.0061	±	0.0004	0.0043	±	0.0003
214	0.0186	±	0.0012	0.0145	±	0.0013	0.0091	±	0.0008	0.0049	±	0.0006	0.0045	±	0.0004
215	0.0194	±	0.0009	0.0131	±	0.0018	0.0087	±	0.0007	0.0056	±	0.0005	0.0034	±	0.0004
216	0.0159	±	0.0025	0.0133	±	0.0018	0.0079	±	0.0009	0.0054	±	0.0007	0.0038	±	0.0005
217	0.0206	±	0.0007	0.0147	±	0.0015	0.0080	±	0.0007	0.0033	±	0.0006	0.0039	±	0.0004
218	0.0209	±	0.0009	0.0107	±	0.0014	0.0094	±	0.0009	0.0050	±	0.0007	0.0046	±	0.0004
219	0.0126	±	0.0019	0.0145	±	0.0025	0.0097	±	0.0019	0.0057	±	0.0012	0.0051	±	0.0010
220	0.0185	±	0.0013	0.0131	±	0.0017	0.0079	±	0.0009	0.0055	±	0.0007	0.0040	±	0.0006
221	0.0188	±	0.0012	0.0132	±	0.0017	0.0089	±	0.0011	0.0048	±	0.0008	0.0040	±	0.0006
222	0.0177	±	0.0012	0.0153	±	0.0012	0.0099	±	0.0007	0.0070	±	0.0005	0.0041	±	0.0004
223	0.0196	±	0.0017	0.0318	±	0.0019	0.0094	±	0.0011	0.0066	±	0.0009	0.0048	±	0.0007
224	0.0245	±	0.0004	0.0107	±	0.0055	0.0092	±	0.0011	0.0060	±	0.0008	0.0037	±	0.0008
225	0.0236	±	0.0017	0.0280	±	0.0008	0.0098	±	0.0011	0.0052	±	0.0012	0.0070	±	0.0012
226	0.0231	±	0.0007	0.0129	±	0.0013	0.0112	±	0.0008	0.0074	±	0.0005	0.0045	±	0.0004
227	0.0204	±	0.0008	0.0159	±	0.0012	0.0075	±	0.0007	0.0066	±	0.0006	0.0041	±	0.0006
228	0.0213	±	0.0009	0.0133	±	0.0012	0.0100	±	0.0006	0.0048	±	0.0005	0.0033	±	0.0003
229	0.0083	±	0.0014	0.0108	±	0.0016	0.0091	±	0.0008	0.0076	±	0.0005	0.0043	±	0.0004
230	0.0190	±	0.0015	0.0152	±	0.0013	0.0075	±	0.0007	0.0050	±	0.0007	0.0039	±	0.0004
231	0.0239	±	0.0008	0.0141	±	0.0011	0.0091	±	0.0007	0.0054	±	0.0005	0.0027	±	0.0004
232	0.0142	±	0.0014	0.0139	±	0.0010	0.0073	±	0.0005	0.0054	±	0.0005	0.0023	±	0.0003
233	0.0173	±	0.0008	0.0152	±	0.0013	0.0088	±	0.0007	0.0043	±	0.0006	0.0055	±	0.0005
234	0.0273	±	0.0005	0.0287	±	0.0008	0.0070	±	0.0007	0.0044	±	0.0006	0.0037	±	0.0004
235	0.0205	±	0.0005	0.0125	±	0.0011	0.0091	±	0.0005	0.0049	±	0.0004	0.0039	±	0.0003
236	0.0190	±	0.0006	0.0124	±	0.0010	0.0088	±	0.0006	0.0046	±	0.0005	0.0040	±	0.0003
237	0.0196	±	0.0009	0.0147	±	0.0018	0.0106	±	0.0010	0.0065	±	0.0007	0.0035	±	0.0005
238	0.0196	±	0.0008	0.0144	±	0.0016	0.0099	±	0.0009	0.0051	±	0.0007	0.0036	±	0.0005
239	0.0211	±	0.0008	0.0134	±	0.0012	0.0073	±	0.0007	0.0057	±	0.0006	0.0037	±	0.0003
240	0.0205	±	0.0009	0.0129	±	0.0013	0.0112	±	0.0008	0.0078	±	0.0005	0.0037	±	0.0004
241	0.0200	±	0.0008	0.0167	±	0.0019	0.0080	±	0.0013	0.0050	±	0.0010	0.0041	±	0.0008
242	0.0210	±	0.0010	0.0128	±	0.0013	0.0101	±	0.0008	0.0067	±	0.0005	0.0045	±	0.0006
243	0.0217	±	0.0010	0.0126	±	0.0013	0.0083	±	0.0008	0.0045	±	0.0006	0.0042	±	0.0004
244	0.0246	±	0.0013	0.0133	±	0.0017	0.0111	±	0.0009	0.0074	±	0.0008	0.0057	±	0.0005
245	0.0148	±	0.0015	0.0137	±	0.0022	0.0074	±	0.0012	0.0043	±	0.0009	0.0038	±	0.0007
246	0.0203	±	0.0011	0.0112	±	0.0025	0.0099	±	0.0015	0.0060	±	0.0012	0.0050	±	0.0010
247	0.0157	±	0.0011	0.0151	±	0.0013	0.0091	±	0.0007	0.0039	±	0.0006	0.0026	±	0.0005
248	0.0126	±	0.0030	0.0162	±	0.0024	0.0099	±	0.0013	0.0048	±	0.0010	0.0061	±	0.0010
249	0.0199	±	0.0009	0.0122	±	0.0013	0.0075	±	0.0008	0.0028	±	0.0007	0.0042	±	0.0005
250	0.0213	±	0.0009	0.0131	±	0.0012	0.0108	±	0.0008	0.0054	±	0.0006	0.0055	±	0.0004
251	0.0191	±	0.0020	0.0218	±	0.0011	0.0101	±	0.0014	0.0052	±	0.0008	0.0039	±	0.0007
252	0.0232	±	0.0015	0.0145	±	0.0030	0.0117	±	0.0011	0.0056	±	0.0008	0.0060	±	0.0007
253	0.0098	±	0.0104	0.0150	±	0.0036	0.0131	±	0.0019	0.0097	±	0.0014	0.0061	±	0.0009
254	0.0288	±	0.0012	0.0177	±	0.0023	0.0160	±	0.0013	0.0121	±	0.0008	0.0087	±	0.0007
255	0.0335	±	0.0007	0.0249	±	0.0010	0.0198	±	0.0007	0.0120	±	0.0005	0.0100	±	0.0003
256	0.0398	±	0.0005	0.0299	±	0.0006	0.0241	±	0.0005	0.0166	±	0.0004	0.0122	±	0.0003
257	0.0466	±	0.0005	0.0367	±	0.0005	0.0281	±	0.0005	0.0213	±	0.0002	0.0159	±	0.0002
258	0.0481	±	0.0003	0.0411	±	0.0004	0.0360	±	0.0005	0.0245	±	0.0002	0.0196	±	0.0001
259	0.0413	±	0.0003	0.0357	±	0.0003	0.0309	±	0.0002	0.0229	±	0.0001	0.0194	±	0.0001

Table S8: Results for the fit of Equation 2 to the experimental spectral density $J(0.87\omega_H)$

residue	λ (ns)			μ (ns.(rad.s^{-1}))		
145	0.1778	±	0.0024	0.0178	±	0.0002
146	0.2007	±	0.0022	0.0160	±	0.0002
147	0.2393	±	0.0031	0.0139	±	0.0002
148	0.2259	±	0.0029	0.0131	±	0.0002
149	0.2063	±	0.0030	0.0128	±	0.0002
150	0.1980	±	0.0026	0.0135	±	0.0002
151	0.2001	±	0.0027	0.0120	±	0.0002
152	0.1937	±	0.0023	0.0122	±	0.0002
153	0.2039	±	0.0021	0.0116	±	0.0002
154	0.2539	±	0.0033	0.0099	±	0.0002
155	0.2058	±	0.0028	0.0110	±	0.0002
156	0.2031	±	0.0023	0.0115	±	0.0002
157	0.2159	±	0.0025	0.0114	±	0.0002
158	0.2102	±	0.0023	0.0117	±	0.0002
159	0.2033	±	0.0028	0.0124	±	0.0002
160	0.1941	±	0.0023	0.0115	±	0.0002
161	0.1935	±	0.0033	0.0122	±	0.0002
162	0.1851	±	0.0031	0.0125	±	0.0002
163	0.1774	±	0.0027	0.0122	±	0.0002
164	0.2051	±	0.0034	0.0106	±	0.0002
165	0.1649	±	0.0033	0.0118	±	0.0003
167	0.1801	±	0.0046	0.0096	±	0.0004
168	0.1711	±	0.0063	0.0094	±	0.0005
169	0.1687	±	0.0067	0.0077	±	0.0006
171	0.1662	±	0.0072	0.0058	±	0.0007
172	0.1322	±	0.0086	0.0067	±	0.0009
173	0.0797	±	0.0092	0.0087	±	0.0011
174	0.1698	±	0.0096	0.0052	±	0.0014
175	0.1686	±	0.0096	0.0075	±	0.0009
176	0.1822	±	0.0134	0.0051	±	0.0009
177	0.1639	±	0.0084	0.0075	±	0.0007
178	0.1590	±	0.0054	0.0082	±	0.0005
179	0.1778	±	0.0051	0.0070	±	0.0004
180	0.1748	±	0.0036	0.0084	±	0.0004
181	0.1976	±	0.0044	0.0075	±	0.0003
183	0.1999	±	0.0046	0.0084	±	0.0003
184	0.1710	±	0.0052	0.0101	±	0.0004
185	0.1679	±	0.0031	0.0116	±	0.0002
187	0.1610	±	0.0042	0.0111	±	0.0003
188	0.1817	±	0.0049	0.0096	±	0.0004
189	0.2167	±	0.0065	0.0079	±	0.0004
190	0.1757	±	0.0040	0.0106	±	0.0003
192	0.1877	±	0.0028	0.0094	±	0.0002
193	0.1848	±	0.0037	0.0101	±	0.0003
194	0.2010	±	0.0036	0.0082	±	0.0003
195	0.1833	±	0.0048	0.0095	±	0.0003
197	0.1699	±	0.0031	0.0105	±	0.0002
198	0.1739	±	0.0032	0.0093	±	0.0003
199	0.1917	±	0.0026	0.0092	±	0.0002
200	0.1841	±	0.0024	0.0091	±	0.0002
201	0.1735	±	0.0034	0.0098	±	0.0003
202	0.1653	±	0.0028	0.0094	±	0.0002
204	0.1763	±	0.0035	0.0076	±	0.0003
205	0.0736	±	0.0027	0.0111	±	0.0003
206	0.1407	±	0.0044	0.0066	±	0.0003
207	0.0900	±	0.0033	0.0045	±	0.0003
208	0.0780	±	0.0052	0.0011	±	0.0005
209	0.0685	±	0.0059	0.0011	±	0.0005
210	0.0930	±	0.0024	0.0000	±	0.0001
211	0.0676	±	0.0066	0.0018	±	0.0004

212	0.0815	±	0.0064	0.0007	±	0.0005
213	0.1015	±	0.0037	0.0007	±	0.0004
214	0.0840	±	0.0061	0.0014	±	0.0004
215	0.0905	±	0.0051	0.0005	±	0.0004
216	0.0748	±	0.0100	0.0014	±	0.0007
217	0.0961	±	0.0037	0.0002	±	0.0002
218	0.0898	±	0.0055	0.0012	±	0.0005
219	0.0509	±	0.0112	0.0036	±	0.0011
220	0.0819	±	0.0074	0.0011	±	0.0006
221	0.0861	±	0.0068	0.0009	±	0.0006
222	0.0850	±	0.0062	0.0018	±	0.0004
223	0.1128	±	0.0091	0.0011	±	0.0007
224	0.1163	±	0.0025	0.0001	±	0.0002
225	0.1575	±	0.0071	0.0005	±	0.0007
226	0.1032	±	0.0044	0.0014	±	0.0004
227	0.0921	±	0.0050	0.0010	±	0.0005
228	0.1010	±	0.0037	0.0001	±	0.0002
229	0.0376	±	0.0072	0.0041	±	0.0005
230	0.0870	±	0.0066	0.0007	±	0.0005
231	0.1100	±	0.0030	0.0000	±	0.0000
232	0.0809	±	0.0046	0.0002	±	0.0003
233	0.0743	±	0.0051	0.0021	±	0.0005
234	0.1360	±	0.0021	0.0000	±	0.0000
235	0.0945	±	0.0031	0.0005	±	0.0003
236	0.0857	±	0.0037	0.0009	±	0.0003
237	0.0920	±	0.0057	0.0010	±	0.0005
238	0.0929	±	0.0050	0.0006	±	0.0005
239	0.0958	±	0.0041	0.0004	±	0.0003
240	0.0933	±	0.0053	0.0016	±	0.0005
241	0.0934	±	0.0052	0.0007	±	0.0006
242	0.0908	±	0.0061	0.0017	±	0.0005
243	0.0964	±	0.0051	0.0005	±	0.0004
244	0.1022	±	0.0069	0.0021	±	0.0006
245	0.0677	±	0.0089	0.0013	±	0.0007
246	0.0875	±	0.0078	0.0017	±	0.0009
247	0.0835	±	0.0048	0.0002	±	0.0003
248	0.0631	±	0.0138	0.0032	±	0.0011
249	0.0897	±	0.0044	0.0003	±	0.0003
250	0.0897	±	0.0052	0.0021	±	0.0004
251	0.1257	±	0.0061	0.0001	±	0.0003
252	0.1014	±	0.0088	0.0017	±	0.0007
253	0.0919	±	0.0253	0.0036	±	0.0014
254	0.1108	±	0.0077	0.0055	±	0.0007
255	0.1367	±	0.0039	0.0055	±	0.0004
256	0.1596	±	0.0032	0.0076	±	0.0003
257	0.1795	±	0.0027	0.0107	±	0.0002
258	0.1730	±	0.0020	0.0146	±	0.0001
259	0.1380	±	0.0014	0.0152	±	0.0001

Table S9: Spectral density function J(ω_N) (ns) derived from relaxation data at five magnetic fields)

residue	9.4 T			11.8 T			14.1 T			18.8 T			23.5 T		
145	0.1342	±	0.0013	0.1395	±	0.0015	0.1296	±	0.0024	0.1207	±	0.0004	0.1135	±	0.0004
146	0.1638	±	0.0012	0.1738	±	0.0014	0.1556	±	0.0024	0.1409	±	0.0003	0.1359	±	0.0004
147	0.1814	±	0.0015	0.1753	±	0.0017	0.1834	±	0.0025	0.1585	±	0.0004	0.1487	±	0.0004
148	0.1971	±	0.0019	0.1908	±	0.0016	0.2048	±	0.0030	0.1685	±	0.0005	0.1522	±	0.0004
149	0.2168	±	0.0016	0.2104	±	0.0018	0.2085	±	0.0027	0.1770	±	0.0005	0.1583	±	0.0005
150	0.2354	±	0.0019	0.2305	±	0.0020	0.2232	±	0.0038	0.1880	±	0.0006	0.1671	±	0.0005
151	0.2421	±	0.0022	0.2359	±	0.0022	0.2167	±	0.0048	0.1752	±	0.0006	0.1708	±	0.0006
152	0.2563	±	0.0020	0.2533	±	0.0018	0.2428	±	0.0037	0.2027	±	0.0005	0.1828	±	0.0005
153	0.2623	±	0.0017	0.2385	±	0.0017	0.2285	±	0.0036	0.1911	±	0.0005	0.1732	±	0.0005
154	0.2432	±	0.0019	0.2705	±	0.0112	0.2344	±	0.0033	0.1953	±	0.0005	0.1828	±	0.0005
155	0.2743	±	0.0028	0.2453	±	0.0020	0.2234	±	0.0040	0.2006	±	0.0006	0.1795	±	0.0006
156	0.2857	±	0.0019	0.2662	±	0.0028	0.2479	±	0.0039	0.2146	±	0.0005	0.1904	±	0.0006
157	0.2713	±	0.0020	0.2516	±	0.0021	0.2500	±	0.0040	0.2089	±	0.0006	0.1829	±	0.0006
158	0.2366	±	0.0016	0.2344	±	0.0018	0.2350	±	0.0031	0.1953	±	0.0005	0.1716	±	0.0005
159	0.2360	±	0.0021	0.2282	±	0.0022	0.2001	±	0.0041	0.1875	±	0.0007	0.1577	±	0.0006
160	0.2330	±	0.0021	0.2196	±	0.0018	0.2149	±	0.0039	0.1910	±	0.0005	0.1656	±	0.0005
161	0.2388	±	0.0035	0.2346	±	0.0028	0.2251	±	0.0068	0.1828	±	0.0008	0.1683	±	0.0008
162	0.2513	±	0.0024	0.2451	±	0.0027	0.2350	±	0.0041	0.1968	±	0.0006	0.1710	±	0.0006
163	0.2550	±	0.0021	0.2433	±	0.0023	0.2182	±	0.0045	0.2023	±	0.0008	0.1622	±	0.0007
164	0.2721	±	0.0028	0.2519	±	0.0026	0.2428	±	0.0056	0.2016	±	0.0007	0.1757	±	0.0007
165	0.2760	±	0.0029	0.2457	±	0.0035	0.2404	±	0.0059	0.1940	±	0.0009	0.1644	±	0.0007
167	0.3433	±	0.0041	0.3010	±	0.0043	0.2623	±	0.0069	0.2111	±	0.0012	0.1799	±	0.0011
168	0.3611	±	0.0058	0.3295	±	0.0051	0.2850	±	0.0098	0.2247	±	0.0015	0.1805	±	0.0012
169	0.3705	±	0.0057	0.3327	±	0.0052	0.2774	±	0.0106	0.2183	±	0.0017	0.1783	±	0.0014
171	0.4202	±	0.0064	0.3653	±	0.0063	0.2994	±	0.0125	0.2343	±	0.0023	0.1935	±	0.0018
172	0.4338	±	0.0082	0.3860	±	0.0074	0.3310	±	0.0164	0.2420	±	0.0030	0.1935	±	0.0023
173	0.4176	±	0.0106	0.3518	±	0.0085	0.2989	±	0.0176	0.2597	±	0.0037	0.2123	±	0.0030
174	0.4454	±	0.0092	0.3726	±	0.0127	0.3352	±	0.0235	0.2539	±	0.0047	0.2046	±	0.0038
175	0.4287	±	0.0103	0.3505	±	0.0076	0.3515	±	0.0211	0.2408	±	0.0030	0.1907	±	0.0024
176	0.4455	±	0.0371	0.3059	±	0.0105	0.2742	±	0.0220	0.2343	±	0.0036	0.1877	±	0.0026
177	0.3935	±	0.0089	0.3524	±	0.0073	0.3057	±	0.0168	0.2483	±	0.0026	0.2031	±	0.0020
178	0.3724	±	0.0073	0.3478	±	0.0068	0.3311	±	0.0137	0.2417	±	0.0019	0.2023	±	0.0016
179	0.3714	±	0.0066	0.3363	±	0.0056	0.3202	±	0.0125	0.2327	±	0.0016	0.1889	±	0.0014
180	0.3724	±	0.0038	0.3052	±	0.0033	0.2983	±	0.0073	0.2380	±	0.0011	0.2015	±	0.0010
181	0.3429	±	0.0034	0.3923	±	0.0034	0.2254	±	0.0051	0.2278	±	0.0008	0.1856	±	0.0007
183	0.3104	±	0.0051	0.3024	±	0.0043	0.2822	±	0.0103	0.2062	±	0.0012	0.1844	±	0.0011
184	0.2868	±	0.0067	0.2797	±	0.0052	0.2378	±	0.0127	0.1984	±	0.0018	0.1698	±	0.0013
185	0.2723	±	0.0029	0.2516	±	0.0028	0.2297	±	0.0065	0.1915	±	0.0009	0.1662	±	0.0008
187	0.3065	±	0.0045	0.2978	±	0.0042	0.2701	±	0.0093	0.2066	±	0.0013	0.1761	±	0.0011
188	0.3181	±	0.0058	0.3118	±	0.0052	0.2719	±	0.0118	0.2186	±	0.0015	0.1825	±	0.0012
189	0.3572	±	0.0081	0.3144	±	0.0073	0.2864	±	0.0139	0.2185	±	0.0015	0.1892	±	0.0013
190	0.3375	±	0.0042	0.3106	±	0.0050	0.2771	±	0.0084	0.2238	±	0.0011	0.1889	±	0.0010
192	0.3371	±	0.0029	0.2588	±	0.0029	0.2846	±	0.0056	0.2159	±	0.0007	0.1782	±	0.0007
193	0.3288	±	0.0038	0.2799	±	0.0037	0.2679	±	0.0070	0.2210	±	0.0009	0.1833	±	0.0008
194	0.3282	±	0.0041	0.2925	±	0.0040	0.2787	±	0.0078	0.2137	±	0.0010	0.1826	±	0.0009
195	0.3035	±	0.0071	0.2905	±	0.0045	0.2502	±	0.0129	0.2021	±	0.0013	0.1743	±	0.0012
197	0.3461	±	0.0043	0.2882	±	0.0029	0.2671	±	0.0079	0.2165	±	0.0010	0.1821	±	0.0008
198	0.3393	±	0.0041	0.3157	±	0.0035	0.2877	±	0.0080	0.2140	±	0.0011	0.1782	±	0.0010
199	0.3398	±	0.0026	0.2975	±	0.0022	0.2664	±	0.0043	0.2136	±	0.0006	0.1810	±	0.0006
200	0.3137	±	0.0019	0.2693	±	0.0017	0.2731	±	0.0054	0.2149	±	0.0006	0.1827	±	0.0006
201	0.3404	±	0.0032	0.3438	±	0.0030	0.2647	±	0.0055	0.2067	±	0.0008	0.1767	±	0.0007
202	0.3639	±	0.0030	0.3021	±	0.0023	0.2680	±	0.0049	0.2214	±	0.0007	0.1809	±	0.0007
204	0.3880	±	0.0042	0.3366	±	0.0030	0.2873	±	0.0066	0.2351	±	0.0009	0.1871	±	0.0008
205	0.4116	±	0.0053	0.3436	±	0.0044	0.3014	±	0.0105	0.2305	±	0.0014	0.1744	±	0.0010
206	0.4016	±	0.0054	0.3456	±	0.0045	0.2847	±	0.0087	0.2159	±	0.0011	0.1744	±	0.0009
207	0.4555	±	0.0055	0.3522	±	0.0043	0.2679	±	0.0072	0.1863	±	0.0009	0.1406	±	0.0007
208	0.6039	±	0.0119	0.4713	±	0.0077	0.3472	±	0.0139	0.2342	±	0.0018	0.1658	±	0.0014
209	0.5896	±	0.0091	0.4632	±	0.0054	0.3690	±	0.0123	0.2302	±	0.0013	0.1652	±	0.0011
210	0.5912	±	0.0077	0.4429	±	0.0052	0.3295	±	0.0089	0.2218	±	0.0010	0.1556	±	0.0008

211	0.5703	±	0.0120	0.4641	±	0.0070	0.3546	±	0.0142	0.2214	±	0.0015	0.1570	±	0.0011
212	0.5980	±	0.0090	0.4373	±	0.0059	0.3565	±	0.0124	0.2180	±	0.0013	0.1626	±	0.0011
213	0.6162	±	0.0075	0.4743	±	0.0053	0.3595	±	0.0086	0.2232	±	0.0010	0.1614	±	0.0008
214	0.6186	±	0.0106	0.4607	±	0.0062	0.3648	±	0.0133	0.2226	±	0.0015	0.1577	±	0.0011
215	0.6133	±	0.0193	0.4434	±	0.0093	0.3446	±	0.0131	0.2307	±	0.0013	0.1642	±	0.0012
216	0.6814	±	0.0859	0.4894	±	0.0080	0.3379	±	0.0142	0.2283	±	0.0016	0.1620	±	0.0013
217	0.6108	±	0.0095	0.4844	±	0.0061	0.3592	±	0.0122	0.2300	±	0.0012	0.1629	±	0.0010
218	0.6238	±	0.0109	0.4221	±	0.0065	0.3660	±	0.0150	0.2201	±	0.0019	0.1534	±	0.0014
219	0.5996	±	0.0156	0.4744	±	0.0119	0.3531	±	0.0266	0.2263	±	0.0036	0.1675	±	0.0033
220	0.5943	±	0.0100	0.4766	±	0.0073	0.3414	±	0.0136	0.2269	±	0.0018	0.1618	±	0.0015
221	0.5552	±	0.0100	0.4162	±	0.0083	0.3300	±	0.0158	0.2036	±	0.0020	0.1583	±	0.0016
222	0.5756	±	0.0087	0.4609	±	0.0057	0.3466	±	0.0114	0.2207	±	0.0014	0.1576	±	0.0010
223	0.4409	±	0.0285	0.3342	±	0.0109	0.3458	±	0.0181	0.2179	±	0.0026	0.1628	±	0.0021
224	0.6127	±	0.0100	0.4971	±	0.0199	0.3490	±	0.0160	0.2358	±	0.0021	0.1707	±	0.0017
225	0.4863	±	0.0143	0.2752	±	0.0042	0.2715	±	0.0233	0.2263	±	0.0037	0.1676	±	0.0039
226	0.5376	±	0.0089	0.4404	±	0.0068	0.3596	±	0.0136	0.2245	±	0.0015	0.1607	±	0.0012
227	0.6042	±	0.0138	0.4961	±	0.0072	0.3280	±	0.0158	0.2338	±	0.0019	0.1731	±	0.0018
228	0.6003	±	0.0080	0.4772	±	0.0050	0.3553	±	0.0113	0.2304	±	0.0012	0.1666	±	0.0011
229	0.5946	±	0.0112	0.5231	±	0.0077	0.3709	±	0.0138	0.2361	±	0.0016	0.1742	±	0.0013
230	0.6391	±	0.0106	0.5012	±	0.0065	0.3428	±	0.0125	0.2506	±	0.0016	0.1705	±	0.0014
231	0.6153	±	0.0087	0.4742	±	0.0054	0.3601	±	0.0117	0.2292	±	0.0012	0.1641	±	0.0010
232	0.6131	±	0.0072	0.4754	±	0.0045	0.3540	±	0.0089	0.2384	±	0.0010	0.1638	±	0.0008
233	0.6588	±	0.0101	0.5140	±	0.0066	0.3718	±	0.0117	0.2486	±	0.0014	0.1775	±	0.0013
234	0.5565	±	0.0078	0.4134	±	0.0039	0.3369	±	0.0127	0.2375	±	0.0012	0.1687	±	0.0010
235	0.6105	±	0.0072	0.4631	±	0.0043	0.3480	±	0.0085	0.2216	±	0.0009	0.1582	±	0.0008
236	0.6088	±	0.0075	0.4604	±	0.0047	0.3760	±	0.0092	0.2325	±	0.0010	0.1642	±	0.0009
237	0.6303	±	0.0129	0.4934	±	0.0083	0.3673	±	0.0160	0.2355	±	0.0019	0.1654	±	0.0014
238	0.6039	±	0.0104	0.4792	±	0.0067	0.3639	±	0.0148	0.2291	±	0.0016	0.1701	±	0.0013
239	0.5464	±	0.0091	0.4391	±	0.0061	0.3018	±	0.0114	0.2207	±	0.0013	0.1517	±	0.0010
240	0.5534	±	0.0079	0.4284	±	0.0056	0.3274	±	0.0105	0.2165	±	0.0012	0.1610	±	0.0010
241	0.6138	±	0.0142	0.4822	±	0.0096	0.3661	±	0.0234	0.2397	±	0.0031	0.1686	±	0.0024
242	0.6174	±	0.0105	0.4703	±	0.0058	0.3820	±	0.0149	0.2422	±	0.0017	0.1736	±	0.0015
243	0.6284	±	0.0102	0.4814	±	0.0063	0.3637	±	0.0126	0.2392	±	0.0014	0.1713	±	0.0010
244	0.6269	±	0.0136	0.4655	±	0.0081	0.3641	±	0.0163	0.2391	±	0.0022	0.1660	±	0.0016
245	0.6606	±	0.0132	0.5007	±	0.0105	0.3389	±	0.0174	0.2400	±	0.0023	0.1636	±	0.0017
246	0.6374	±	0.0135	0.4878	±	0.0107	0.3586	±	0.0189	0.2288	±	0.0030	0.1652	±	0.0023
247	0.6641	±	0.0097	0.5348	±	0.0065	0.3725	±	0.0116	0.2483	±	0.0013	0.1781	±	0.0011
248	0.6086	±	0.0161	0.5057	±	0.0127	0.3380	±	0.0217	0.2297	±	0.0032	0.1629	±	0.0025
249	0.6433	±	0.0088	0.4783	±	0.0060	0.3601	±	0.0117	0.2262	±	0.0014	0.1580	±	0.0011
250	0.6269	±	0.0094	0.4693	±	0.0054	0.3707	±	0.0112	0.2429	±	0.0015	0.1670	±	0.0012
251	0.6180	±	0.0135	0.3797	±	0.0071	0.4175	±	0.0202	0.2467	±	0.0021	0.1738	±	0.0027
252	0.6270	±	0.0113	0.4458	±	0.0131	0.3437	±	0.0155	0.2273	±	0.0021	0.1693	±	0.0017
253	0.5700	±	0.0123	0.4229	±	0.0220	0.3309	±	0.0236	0.2451	±	0.0034	0.1731	±	0.0034
254	0.5356	±	0.0094	0.4274	±	0.0082	0.3622	±	0.0156	0.2264	±	0.0022	0.1726	±	0.0019
255	0.4783	±	0.0048	0.3791	±	0.0042	0.3271	±	0.0074	0.2270	±	0.0011	0.1758	±	0.0010
256	0.4190	±	0.0040	0.3467	±	0.0030	0.3072	±	0.0059	0.2337	±	0.0008	0.1836	±	0.0008
257	0.3242	±	0.0028	0.2862	±	0.0021	0.2546	±	0.0050	0.2067	±	0.0006	0.1690	±	0.0006
258	0.2259	±	0.0017	0.2073	±	0.0015	0.2063	±	0.0031	0.1610	±	0.0004	0.1429	±	0.0005
259	0.1113	±	0.0007	0.1153	±	0.0007	0.1129	±	0.0011	0.1024	±	0.0002	0.0965	±	0.0002

Table S10: Spectral density function J(0) (ns) derived from relaxation data at five magnetic fields)

residue	9.4T			11.8T			14.1T			18.8T			23.5T		
145	0.177	±	0.054	0.188	±	0.028	0.142	±	0.043	0.228	±	0.013	0.2078	±	0.0087
146	0.250	±	0.035	0.232	±	0.022	0.261	±	0.030	0.235	±	0.008	0.2648	±	0.0060
147	0.289	±	0.040	0.252	±	0.024	0.300	±	0.033	0.283	±	0.009	0.2851	±	0.0058
148	0.309	±	0.051	0.283	±	0.027	0.368	±	0.046	0.297	±	0.011	0.3190	±	0.0072
149	0.332	±	0.042	0.303	±	0.028	0.340	±	0.034	0.345	±	0.012	0.3382	±	0.0068
150	0.383	±	0.063	0.347	±	0.035	0.330	±	0.051	0.341	±	0.016	0.3499	±	0.0095
151	0.440	±	0.108	0.349	±	0.041	0.339	±	0.078	0.320	±	0.021	0.3643	±	0.0125
152	0.301	±	0.049	0.377	±	0.028	0.394	±	0.048	0.339	±	0.012	0.3696	±	0.0074
153	0.404	±	0.057	0.356	±	0.029	0.447	±	0.050	0.316	±	0.011	0.3819	±	0.0081
154	0.187	±	0.058	0.270	±	0.083	0.333	±	0.032	0.345	±	0.011	0.3740	±	0.0074
155	0.366	±	0.067	0.345	±	0.035	0.297	±	0.048	0.401	±	0.017	0.3902	±	0.0096
156	0.458	±	0.066	0.456	±	0.052	0.423	±	0.052	0.400	±	0.013	0.4462	±	0.0097
157	0.427	±	0.059	0.380	±	0.033	0.421	±	0.049	0.399	±	0.015	0.3875	±	0.0088
158	0.319	±	0.038	0.348	±	0.027	0.388	±	0.040	0.340	±	0.010	0.3543	±	0.0071
159	0.315	±	0.069	0.365	±	0.042	0.268	±	0.053	0.378	±	0.022	0.3250	±	0.0124
160	0.358	±	0.094	0.333	±	0.032	0.310	±	0.055	0.355	±	0.015	0.3567	±	0.0094
161	0.191	±	0.132	0.569	±	0.093	0.257	±	0.113	0.402	±	0.033	0.3331	±	0.0172
162	0.349	±	0.055	0.415	±	0.086	0.420	±	0.055	0.410	±	0.017	0.4036	±	0.0103
163	0.345	±	0.060	0.387	±	0.041	0.408	±	0.067	0.432	±	0.0238	0.4043	±	0.0136
164	0.444	±	0.084	0.391	±	0.040	0.531	±	0.082	0.470	±	0.020	0.5026	±	0.0137
165	0.746	±	0.135	0.531	±	0.066	0.549	±	0.075	0.667	±	0.029	0.6251	±	0.0143
167	0.826	±	0.152	0.826	±	0.088	0.905	±	0.128	0.852	±	0.045	0.8308	±	0.0238
168	0.854	±	0.197	0.839	±	0.105	0.953	±	0.171	1.038	±	0.062	0.9383	±	0.0284
169	1.125	±	0.287	0.966	±	0.129	0.917	±	0.197	1.149	±	0.079	1.1486	±	0.0439
171	1.283	±	0.267	1.073	±	0.140	1.365	±	0.251	1.294	±	0.106	1.3194	±	0.0576
172	1.406	±	0.362	1.202	±	0.192	1.194	±	0.365	1.672	±	0.188	1.4913	±	0.0904
173	1.249	±	0.332	1.106	±	0.163	1.318	±	0.356	1.589	±	0.167	1.6010	±	0.1031
174	1.027	±	0.580	1.420	±	0.451	1.810	±	1.354	1.714	±	0.285	1.4358	±	0.1482
175	2.431	±	5.327	1.016	±	0.215	1.616	±	0.721	1.325	±	0.191	1.1822	±	0.0858
176	1.373	±	9.628	0.612	±	0.242	1.090	±	0.839	1.181	±	0.231	1.0089	±	0.1051
177	0.971	±	0.351	0.948	±	0.175	0.965	±	0.299	1.127	±	0.112	1.0547	±	0.0562
178	0.953	±	0.248	0.882	±	0.135	0.986	±	0.263	1.012	±	0.076	1.0253	±	0.0414
179	0.620	±	0.217	0.774	±	0.129	0.707	±	0.213	0.866	±	0.065	0.8805	±	0.0389
180	0.753	±	0.112	0.688	±	0.055	0.716	±	0.096	0.770	±	0.034	0.7774	±	0.0191
181	0.708	±	0.094	1.172	±	0.083	0.388	±	0.049	0.728	±	0.023	0.7060	±	0.0128
183	0.608	±	0.248	0.577	±	0.101	0.593	±	0.188	0.618	±	0.053	0.5825	±	0.0215
184	0.068	±	10.120	0.448	±	0.141	0.348	±	0.895	0.548	±	0.099	0.5533	±	0.0548
185	0.412	±	0.133	0.413	±	0.059	0.424	±	0.128	0.446	±	0.033	0.4354	±	0.0180
187	0.599	±	0.251	0.538	±	0.095	0.613	±	0.181	0.514	±	0.042	0.5571	±	0.0262
188	0.461	±	0.277	0.872	±	0.252	0.760	±	0.350	0.563	±	0.057	0.5401	±	0.0321
189	0.489	±	0.399	0.668	±	0.198	1.002	±	0.710	0.667	±	0.075	0.6592	±	0.0423
190	0.647	±	0.148	0.634	±	0.088	0.547	±	0.116	0.654	±	0.035	0.6491	±	0.0206
192	0.651	±	0.102	0.513	±	0.046	0.599	±	0.072	0.688	±	0.022	0.6251	±	0.0128
193	0.695	±	0.138	0.523	±	0.063	0.564	±	0.092	0.605	±	0.029	0.6184	±	0.0180
194	0.727	±	0.202	0.585	±	0.069	0.594	±	0.117	0.691	±	0.034	0.6940	±	0.0215
195	0.495	±	2.524	0.631	±	0.144	0.355	±	0.267	0.701	±	0.107	0.7008	±	0.0696
197	0.593	±	0.154	0.574	±	0.064	0.617	±	0.128	0.663	±	0.034	0.7219	±	0.0217
198	0.646	±	0.148	0.707	±	0.078	0.738	±	0.142	0.639	±	0.033	0.6369	±	0.0212
199	0.689	±	0.079	0.637	±	0.040	0.620	±	0.056	0.642	±	0.016	0.6547	±	0.0108
200	0.603	±	0.047	0.470	±	0.030	0.761	±	0.082	0.670	±	0.019	0.6697	±	0.0118
201	0.593	±	0.081	0.733	±	0.062	0.534	±	0.072	0.572	±	0.019	0.6284	±	0.0135
202	0.805	±	0.090	0.740	±	0.047	0.704	±	0.061	0.755	±	0.020	0.7905	±	0.0129
204	0.883	±	0.152	0.847	±	0.070	0.881	±	0.115	0.927	±	0.032	0.8410	±	0.0183
205	0.709	±	0.198	0.908	±	0.104	0.843	±	0.186	0.896	±	0.054	0.9731	±	0.0395
206	1.114	±	0.230	0.973	±	0.104	1.083	±	0.176	1.136	±	0.056	1.1579	±	0.0364
207	1.776	±	0.279	1.453	±	0.133	1.523	±	0.207	1.546	±	0.059	1.5727	±	0.0373
208	2.207	±	0.619	1.960	±	0.284	2.299	±	0.576	2.322	±	0.175	2.1196	±	0.1047
209	2.498	±	0.471	2.588	±	0.261	2.355	±	0.340	2.436	±	0.109	2.5720	±	0.0817
210	2.787	±	0.436	2.223	±	0.183	2.187	±	0.256	2.676	±	0.109	2.6234	±	0.0687

211	2.475	±	0.792	2.216	±	0.356	2.352	±	0.520	2.286	±	0.139	2.3763	±	0.0965
212	1.766	±	0.409	1.604	±	0.200	2.067	±	0.380	2.586	±	0.144	2.4808	±	0.0924
213	2.379	±	0.326	2.480	±	0.206	2.677	±	0.294	2.453	±	0.081	2.4709	±	0.0544
214	2.838	±	0.565	2.500	±	0.276	2.503	±	0.386	2.757	±	0.150	2.6062	±	0.0838
215	2.744	±	0.762	2.420	±	0.632	2.925	±	0.601	2.539	±	0.138	2.4450	±	0.0957
216	3.194	±	0.966	2.388	±	0.362	2.162	±	0.427	2.629	±	0.169	2.5083	±	0.1090
217	2.492	±	0.423	2.534	±	0.255	2.971	±	0.399	2.528	±	0.105	2.5773	±	0.0705
218	2.299	±	0.594	1.899	±	0.260	2.532	±	0.561	2.541	±	0.184	2.4391	±	0.1218
219	3.238	±	1.272	2.582	±	0.675	2.635	±	1.044	2.605	±	0.443	2.4647	±	0.3064
220	2.018	±	0.398	2.185	±	0.286	1.901	±	0.372	2.234	±	0.151	2.4564	±	0.1273
221	2.679	±	0.634	2.183	±	0.349	2.254	±	0.527	2.298	±	0.209	2.3261	±	0.1221
222	2.060	±	0.308	2.280	±	0.223	2.221	±	0.342	2.142	±	0.100	2.1907	±	0.0639
223	1.858	±	9.406	0.865	±	0.484	2.155	±	17.533	2.589	±	0.295	2.1817	±	0.1802
224	2.074	±	0.386	2.125	±	0.910	1.790	±	0.374	2.187	±	0.156	2.2338	±	0.1065
225	1.730	±	1.040	0.381	±	0.106	0.816	±	129.096	1.642	±	0.293	2.1876	±	0.3284
226	1.649	±	0.398	1.609	±	0.203	1.882	±	0.407	1.551	±	0.083	1.6575	±	0.0650
227	2.353	±	1.076	2.251	±	0.306	2.946	±	1.250	2.429	±	0.246	2.5573	±	0.1942
228	2.422	±	0.323	2.399	±	0.190	2.165	±	0.290	2.338	±	0.092	2.4355	±	0.0773
229	2.785	±	0.873	2.007	±	0.241	2.077	±	0.407	2.327	±	0.121	2.4386	±	0.0966
230	2.208	±	0.435	2.247	±	0.228	1.932	±	0.304	2.612	±	0.158	2.3311	±	0.0923
231	2.648	±	0.431	2.350	±	0.203	2.338	±	0.302	2.286	±	0.093	2.5572	±	0.0834
232	2.326	±	0.265	2.323	±	0.153	2.392	±	0.243	2.306	±	0.067	2.5336	±	0.0562
233	2.581	±	0.451	2.312	±	0.228	2.331	±	0.320	2.238	±	0.085	2.2331	±	0.0718
234	1.585	±	0.184	1.106	±	0.083	2.048	±	0.314	2.153	±	0.077	2.4511	±	0.0672
235	2.843	±	0.369	2.176	±	0.151	2.331	±	0.234	2.298	±	0.071	2.4017	±	0.0509
236	2.118	±	0.305	2.105	±	0.157	2.202	±	0.225	2.157	±	0.067	2.2379	±	0.0528
237	2.311	±	0.664	2.027	±	0.319	1.979	±	0.524	2.134	±	0.156	2.2235	±	0.1091
238	2.269	±	0.451	2.495	±	0.333	2.606	±	0.526	2.291	±	0.124	2.5122	±	0.0966
239	2.313	±	0.580	1.999	±	0.221	1.844	±	0.290	2.390	±	0.123	2.1860	±	0.0653
240	1.729	±	0.273	1.741	±	0.162	1.709	±	0.265	1.816	±	0.080	1.9087	±	0.0579
241	4.005	±	2.528	2.116	±	0.412	2.114	±	0.813	2.327	±	0.306	2.3437	±	0.2535
242	2.826	±	0.619	2.157	±	0.221	2.174	±	0.378	2.444	±	0.147	2.5522	±	0.1223
243	3.139	±	0.741	1.973	±	0.209	2.125	±	0.311	2.141	±	0.100	2.2739	±	0.0742
244	2.179	±	0.655	1.903	±	0.258	1.719	±	0.430	2.215	±	0.188	2.1120	±	0.1281
245	2.137	±	0.601	2.800	±	0.524	2.825	±	0.712	2.452	±	0.201	2.3851	±	0.1365
246	2.423	±	0.679	2.303	±	0.427	1.930	±	0.526	2.255	±	0.281	2.4582	±	0.2016
247	2.584	±	0.472	2.357	±	0.244	2.146	±	0.300	2.292	±	0.098	2.4138	±	0.0765
248	1.728	±	0.703	1.818	±	0.570	2.246	±	1.129	2.464	±	0.366	2.1150	±	0.2044
249	2.098	±	0.353	2.260	±	0.241	2.346	±	0.358	2.358	±	0.127	2.2028	±	0.0778
250	2.270	±	0.375	1.873	±	0.182	2.313	±	0.324	2.467	±	0.123	2.2753	±	0.0762
251	2.547	±	0.542	1.309	±	0.410	2.733	±	0.764	1.627	±	0.123	2.0680	±	0.1133
252	2.341	±	0.623	1.790	±	0.422	1.958	±	0.475	2.055	±	0.164	2.1438	±	0.1013
253	1.518	±	0.474	1.240	±	0.413	2.398	±	0.805	1.946	±	0.155	2.0637	±	0.1235
254	1.632	±	0.379	1.538	±	0.255	2.006	±	0.434	1.636	±	0.128	1.5464	±	0.0738
255	1.301	±	0.142	1.145	±	0.084	1.300	±	0.143	1.134	±	0.041	1.1837	±	0.0254
256	0.930	±	0.104	0.843	±	0.051	1.001	±	0.092	0.990	±	0.029	0.9821	±	0.0177
257	0.599	±	0.082	0.539	±	0.038	0.576	±	0.067	0.551	±	0.016	0.5578	±	0.0109
258	0.301	±	0.059	0.354	±	0.028	0.385	±	0.054	0.337	±	0.012	0.3410	±	0.0082
259	0.130	±	0.016	0.140	±	0.011	0.138	±	0.017	0.128	±	0.004	0.1491	±	0.0033

12. Two correlation-time analysis of the spectral density function:

Table S11: Parameters of the two correlation-time analysis of the spectral density function in Engrailed

residue	τ_a (ns)			τ_b (ns)			S^2		
145	0.746	+	0.005	0.100	+	0.000	0.378	+	0.003
146	0.819	+	0.004	0.100	+	0.000	0.454	+	0.002
147	0.772	+	0.008	0.100	+	0.000	0.536	+	0.004
148	8.214	+	0.068	0.462	+	0.001	0.187	+	0.002
149	5.266	+	3.300	0.357	+	0.204	0.293	+	0.148
150	6.284	+	0.106	0.512	+	0.004	0.230	+	0.003
151	8.732	+	0.083	0.517	+	0.002	0.206	+	0.002
152	1.189	+	0.008	0.100	+	0.000	0.548	+	0.003
153	1.192	+	0.007	0.100	+	0.000	0.518	+	0.001
154	0.929	+	0.008	0.100	+	0.000	0.615	+	0.003
155	6.057	+	0.050	0.572	+	0.002	0.225	+	0.002
156	1.216	+	0.011	0.100	+	0.000	0.584	+	0.004
157	1.474	+	1.035	0.143	+	0.142	0.533	+	0.107
158	6.841	+	0.082	0.554	+	0.002	0.194	+	0.002
159	8.449	+	0.084	0.528	+	0.002	0.227	+	0.002
160	7.767	+	0.064	0.546	+	0.002	0.238	+	0.001
161	6.817	+	2.804	0.460	+	0.178	0.312	+	0.095
162	1.723	+	1.341	0.140	+	0.132	0.470	+	0.087
163	1.470	+	0.012	0.100	+	0.000	0.466	+	0.002
164	1.308	+	0.008	0.100	+	0.000	0.520	+	0.002
165	1.819	+	0.023	0.100	+	0.000	0.458	+	0.001
167	5.852	+	1.954	0.535	+	0.211	0.415	+	0.049
168	7.397	+	0.166	0.717	+	0.014	0.399	+	0.005
169	7.091	+	0.153	0.700	+	0.010	0.458	+	0.008
171	8.404	+	0.192	0.929	+	0.017	0.450	+	0.008
172	8.541	+	0.293	1.048	+	0.037	0.481	+	0.012
173	11.005	+	0.333	1.298	+	0.037	0.421	+	0.013
174	9.124	+	0.497	1.113	+	0.047	0.422	+	0.013
175	6.664	+	0.344	0.794	+	0.023	0.433	+	0.012
176	8.383	+	0.392	0.855	+	0.019	0.425	+	0.013
177	7.220	+	0.218	0.905	+	0.016	0.404	+	0.008
178	7.485	+	0.148	0.885	+	0.009	0.384	+	0.005
179	6.913	+	1.572	0.711	+	0.203	0.398	+	0.059
180	7.818	+	0.116	0.822	+	0.008	0.319	+	0.003
181	2.603	+	0.704	0.145	+	0.150	0.548	+	0.050
183	4.052	+	2.209	0.386	+	0.274	0.411	+	0.108
184	9.160	+	0.223	0.646	+	0.010	0.397	+	0.005
185	7.628	+	0.084	0.554	+	0.004	0.331	+	0.003
187	6.309	+	2.223	0.528	+	0.212	0.380	+	0.056
188	5.955	+	0.156	0.636	+	0.010	0.351	+	0.006
189	6.356	+	0.163	0.670	+	0.008	0.341	+	0.006
190	4.003	+	2.150	0.408	+	0.288	0.436	+	0.103
192	2.136	+	1.572	0.148	+	0.160	0.510	+	0.048
193	2.392	+	1.234	0.187	+	0.193	0.497	+	0.078
194	8.837	+	0.137	0.718	+	0.004	0.351	+	0.003
195	8.560	+	0.162	0.653	+	0.006	0.389	+	0.006
197	4.901	+	2.478	0.451	+	0.278	0.428	+	0.077
198	7.055	+	0.144	0.652	+	0.009	0.373	+	0.002
199	4.664	+	2.236	0.431	+	0.262	0.399	+	0.092
200	7.964	+	0.058	0.699	+	0.003	0.313	+	0.002
201	4.085	+	1.520	0.374	+	0.213	0.429	+	0.060
202	5.709	+	0.047	0.621	+	0.004	0.377	+	0.002
204	6.283	+	0.094	0.758	+	0.007	0.418	+	0.004
205	5.999	+	2.332	0.604	+	0.400	0.582	+	0.018
206	5.126	+	2.008	0.422	+	0.308	0.533	+	0.048
207	6.724	+	1.201	0.321	+	0.254	0.729	+	0.006
208	7.793	+	0.458	2.411	+	0.369	0.761	+	0.037
209	8.626	+	0.247	2.516	+	0.287	0.722	+	0.018

210	7.682	+	0.127	1.696	+	0.109	0.823	+	0.009
211	7.077	+	0.104	1.391	+	0.080	0.857	+	0.010
212	7.899	+	0.137	1.580	+	0.096	0.793	+	0.009
213	7.056	+	0.070	1.254	+	0.039	0.822	+	0.005
214	7.148	+	0.195	1.411	+	0.143	0.845	+	0.015
215	7.578	+	0.399	1.872	+	0.231	0.789	+	0.024
216	6.720	+	0.170	1.322	+	0.141	0.859	+	0.014
217	7.047	+	0.182	1.562	+	0.165	0.826	+	0.014
218	7.565	+	0.158	1.311	+	0.099	0.824	+	0.011
219	7.756	+	0.528	2.069	+	0.475	0.789	+	0.036
220	7.695	+	0.325	1.827	+	0.273	0.793	+	0.022
221	8.274	+	0.145	1.313	+	0.059	0.781	+	0.010
222	6.768	+	0.051	0.975	+	0.050	0.845	+	0.008
223	11.581	+	0.610	1.360	+	0.038	0.628	+	0.011
224	6.805	+	0.311	1.235	+	0.130	0.783	+	0.019
225	15.150	+	0.521	1.153	+	0.029	0.528	+	0.005
226	6.927	+	0.115	0.974	+	0.056	0.787	+	0.009
227	7.082	+	0.251	1.641	+	0.158	0.800	+	0.021
228	6.926	+	0.137	1.450	+	0.087	0.820	+	0.012
229	6.610	+	0.149	1.397	+	0.112	0.820	+	0.012
230	7.305	+	0.540	2.926	+	0.602	0.738	+	0.058
231	6.843	+	0.121	1.395	+	0.112	0.838	+	0.007
232	8.580	+	0.350	3.340	+	0.259	0.690	+	0.029
233	6.435	+	0.187	2.048	+	0.299	0.811	+	0.021
234	8.009	+	0.165	1.350	+	0.049	0.686	+	0.008
235	7.068	+	0.098	1.255	+	0.054	0.844	+	0.008
236	7.116	+	0.196	1.645	+	0.161	0.814	+	0.014
237	6.410	+	0.217	1.296	+	0.183	0.857	+	0.017
238	7.640	+	0.206	1.794	+	0.118	0.770	+	0.012
239	7.321	+	0.143	1.114	+	0.071	0.832	+	0.008
240	7.604	+	0.158	1.126	+	0.064	0.772	+	0.009
241	6.855	+	0.364	1.717	+	0.271	0.804	+	0.028
242	7.606	+	0.349	2.128	+	0.271	0.732	+	0.026
243	6.727	+	0.158	1.496	+	0.117	0.807	+	0.012
244	6.375	+	0.246	1.042	+	0.160	0.811	+	0.013
245	6.589	+	0.340	2.338	+	0.797	0.861	+	0.037
246	6.717	+	0.194	1.372	+	0.108	0.840	+	0.017
247	6.777	+	0.187	3.942	+	0.149	0.750	+	0.024
248	6.258	+	0.394	1.135	+	0.463	0.878	+	0.017
249	6.424	+	0.073	0.986	+	0.083	0.894	+	0.006
250	6.230	+	0.159	1.016	+	0.124	0.840	+	0.012
251	7.933	+	0.227	1.488	+	0.082	0.662	+	0.014
252	6.623	+	0.184	1.029	+	0.126	0.798	+	0.009
253	6.760	+	0.329	1.131	+	0.135	0.705	+	0.020
254	5.812	+	0.829	0.623	+	0.264	0.716	+	0.019
255	6.814	+	0.086	0.782	+	0.014	0.587	+	0.004
256	6.427	+	0.067	0.754	+	0.009	0.467	+	0.004
257	3.788	+	1.800	0.325	+	0.215	0.421	+	0.062
258	1.339	+	0.018	0.100	+	0.000	0.394	+	0.003
259	1.004	+	0.006	0.100	+	0.000	0.241	+	0.001

13. Three correlation-time analysis of the spectral density function:

Table S12: Parameters of the three correlation-time analysis of the spectral density function in Engrailed

residue	τ_a (ns)			τ_b (ns)			τ_c (ns)			S^2			S^{2f}		
145	6.852	+	3.202	0.730	+	0.053	0.082	+	0.002	0.030	+	0.012	0.440	+	0.007
146	9.009	+	0.504	0.782	+	0.008	0.086	+	0.001	0.030	+	0.002	0.514	+	0.005
147	8.540	+	0.397	0.753	+	0.008	0.084	+	0.002	0.034	+	0.001	0.601	+	0.005
148	8.214	+	0.068	3.423	+	0.869	0.462	+	0.001	0.187	+	0.002	0.187	+	0.002
149	7.644	+	0.049	2.432	+	1.285	0.504	+	0.002	0.186	+	0.002	0.186	+	0.002
150	5.405	+	0.652	1.101	+	0.025	0.092	+	0.001	0.064	+	0.006	0.585	+	0.007
151	6.454	+	1.456	1.704	+	1.218	0.198	+	0.205	0.119	+	0.056	0.494	+	0.184
152	5.427	+	0.449	1.192	+	0.017	0.087	+	0.001	0.064	+	0.005	0.625	+	0.006
153	5.199	+	0.265	1.057	+	0.017	0.080	+	0.001	0.089	+	0.004	0.630	+	0.006
154	7.344	+	0.420	0.862	+	0.006	0.065	+	0.001	0.054	+	0.002	0.727	+	0.003
155	5.686	+	0.398	2.275	+	1.335	0.304	+	0.256	0.148	+	0.074	0.450	+	0.215
156	6.563	+	0.316	1.172	+	0.012	0.096	+	0.001	0.078	+	0.003	0.665	+	0.006
157	4.775	+	0.329	1.112	+	0.017	0.090	+	0.002	0.085	+	0.007	0.662	+	0.007
158	6.681	+	0.675	1.083	+	0.015	0.083	+	0.001	0.049	+	0.005	0.610	+	0.006
159	2.000	+	0.000	0.906	+	0.048	0.072	+	0.003	0.217	+	0.007	0.611	+	0.010
160	7.270	+	0.903	1.116	+	0.017	0.074	+	0.002	0.046	+	0.005	0.583	+	0.007
161	5.617	+	0.737	1.130	+	0.028	0.079	+	0.001	0.057	+	0.006	0.580	+	0.007
162	7.713	+	0.406	1.221	+	0.021	0.086	+	0.002	0.056	+	0.003	0.577	+	0.005
163	3.605	+	1.304	1.060	+	0.264	0.076	+	0.009	0.181	+	0.105	0.589	+	0.027
164	6.496	+	0.313	1.349	+	0.879	0.128	+	0.153	0.124	+	0.046	0.620	+	0.119
165	8.513	+	0.368	1.255	+	0.035	0.083	+	0.002	0.119	+	0.007	0.596	+	0.008
167	6.738	+	0.333	1.198	+	0.046	0.095	+	0.004	0.230	+	0.010	0.707	+	0.011
168	8.037	+	0.638	1.780	+	0.208	0.129	+	0.007	0.205	+	0.027	0.652	+	0.028
169	7.481	+	0.444	1.269	+	0.102	0.096	+	0.007	0.314	+	0.019	0.759	+	0.018
171	7.810	+	0.516	1.529	+	0.495	0.243	+	0.339	0.367	+	0.039	0.760	+	0.158
172	11.506	+	3.208	2.126	+	0.216	0.103	+	0.022	0.254	+	0.060	0.766	+	0.029
173	20.000	+	0.000	1.923	+	0.061	0.034	+	0.008	0.149	+	0.009	0.774	+	0.012
174	9.214	+	0.951	1.605	+	0.243	0.129	+	0.041	0.310	+	0.051	0.843	+	0.045
175	7.421	+	0.593	1.552	+	0.174	0.150	+	0.026	0.324	+	0.031	0.776	+	0.024
176	8.383	+	0.392	2.550	+	0.919	0.855	+	0.019	0.425	+	0.013	0.425	+	0.013
177	8.870	+	0.807	1.693	+	0.079	0.102	+	0.009	0.203	+	0.020	0.749	+	0.016
178	10.247	+	1.471	1.632	+	0.085	0.095	+	0.005	0.175	+	0.025	0.736	+	0.018
179	6.631	+	0.431	1.346	+	0.062	0.069	+	0.004	0.240	+	0.014	0.749	+	0.012
180	7.866	+	0.171	2.176	+	0.517	0.687	+	0.302	0.289	+	0.066	0.396	+	0.171
181	4.418	+	0.133	1.247	+	0.072	0.083	+	0.003	0.298	+	0.012	0.717	+	0.011
183	5.265	+	0.358	1.121	+	0.040	0.070	+	0.004	0.179	+	0.012	0.698	+	0.010
184	4.511	+	1.964	1.033	+	0.381	0.062	+	0.017	0.226	+	0.119	0.631	+	0.030
185	4.959	+	0.629	1.278	+	0.033	0.075	+	0.002	0.117	+	0.009	0.581	+	0.006
187	5.663	+	0.628	1.494	+	0.056	0.084	+	0.004	0.144	+	0.011	0.604	+	0.009
188	3.746	+	0.909	1.176	+	0.290	0.070	+	0.023	0.230	+	0.086	0.675	+	0.047
189	5.280	+	0.659	1.509	+	1.088	0.192	+	0.239	0.259	+	0.040	0.685	+	0.173
190	6.240	+	0.632	1.398	+	0.078	0.101	+	0.004	0.164	+	0.017	0.670	+	0.015
192	5.550	+	0.344	1.171	+	0.026	0.080	+	0.003	0.191	+	0.007	0.690	+	0.008
193	5.798	+	0.327	1.298	+	0.043	0.092	+	0.003	0.171	+	0.012	0.670	+	0.009
194	6.118	+	0.453	1.072	+	0.023	0.072	+	0.003	0.199	+	0.007	0.734	+	0.010
195	7.509	+	1.482	2.524	+	1.196	0.443	+	0.291	0.326	+	0.090	0.503	+	0.155
197	6.636	+	0.290	1.315	+	0.038	0.095	+	0.002	0.188	+	0.009	0.675	+	0.010
198	5.010	+	0.365	1.295	+	0.041	0.075	+	0.003	0.208	+	0.011	0.666	+	0.008
199	5.299	+	0.150	1.116	+	0.016	0.081	+	0.002	0.218	+	0.004	0.709	+	0.004
200	8.628	+	0.310	1.363	+	0.019	0.073	+	0.001	0.120	+	0.005	0.654	+	0.005
201	4.063	+	0.095	1.038	+	0.035	0.077	+	0.003	0.292	+	0.006	0.690	+	0.006
202	6.454	+	0.194	1.404	+	0.051	0.093	+	0.004	0.218	+	0.007	0.682	+	0.009
204	5.681	+	0.277	1.338	+	0.042	0.079	+	0.003	0.271	+	0.012	0.755	+	0.007
205	16.811	+	7.095	2.278	+	0.791	0.063	+	0.002	0.150	+	0.210	0.607	+	0.016
206	6.889	+	0.163	1.474	+	0.074	0.068	+	0.002	0.342	+	0.011	0.741	+	0.011

207	6.852	+	0.122	1.452	+	0.097	0.042	+	0.003	0.537	+	0.010	0.742	+	0.007
208	7.659	+	0.856	3.691	+	0.513	0.023	+	0.012	0.601	+	0.066	0.918	+	0.014
209	8.862	+	0.404	3.052	+	0.361	0.102	+	0.298	0.639	+	0.040	0.964	+	0.014
210	8.047	+	0.315	2.727	+	0.653	0.531	+	0.410	0.769	+	0.031	0.957	+	0.021
211	8.119	+	0.429	3.753	+	0.477	0.043	+	0.013	0.645	+	0.056	0.926	+	0.011
212	7.731	+	0.199	1.684	+	0.154	0.089	+	0.137	0.771	+	0.022	0.978	+	0.017
213	7.364	+	0.288	3.067	+	1.305	0.605	+	0.429	0.772	+	0.037	0.920	+	0.055
214	7.866	+	0.627	3.149	+	0.983	0.322	+	0.162	0.745	+	0.065	0.960	+	0.013
215	8.015	+	0.733	2.590	+	0.584	0.284	+	0.367	0.712	+	0.061	0.962	+	0.019
216	6.725	+	0.174	1.372	+	0.169	0.260	+	0.113	0.856	+	0.018	0.996	+	0.006
217	7.823	+	0.365	4.000	+	0.000	0.801	+	0.132	0.718	+	0.033	0.931	+	0.014
218	7.475	+	0.285	1.853	+	0.290	0.089	+	0.025	0.784	+	0.023	0.956	+	0.020
219	7.898	+	0.844	3.517	+	0.774	0.073	+	0.049	0.652	+	0.080	0.933	+	0.025
220	7.995	+	0.665	2.926	+	0.790	0.054	+	0.030	0.681	+	0.093	0.948	+	0.025
221	7.574	+	0.235	1.605	+	0.145	0.009	+	0.009	0.711	+	0.021	0.922	+	0.020
222	6.878	+	0.403	2.300	+	1.058	0.067	+	0.016	0.757	+	0.073	0.930	+	0.024
223	11.243	+	1.432	1.667	+	0.166	0.007	+	0.010	0.443	+	0.050	0.850	+	0.031
224	7.004	+	0.547	2.226	+	1.389	0.509	+	0.425	0.750	+	0.052	0.946	+	0.071
225	15.556	+	1.096	1.201	+	0.115	0.476	+	0.360	0.481	+	0.105	0.964	+	0.081
226	5.739	+	0.304	1.288	+	0.184	0.018	+	0.004	0.678	+	0.014	0.859	+	0.013
227	7.299	+	0.649	2.246	+	0.700	0.164	+	0.173	0.757	+	0.057	0.975	+	0.018
228	7.021	+	0.329	2.150	+	1.191	0.392	+	0.458	0.795	+	0.034	0.968	+	0.044
229	6.675	+	0.150	2.037	+	0.206	0.131	+	0.023	0.783	+	0.016	0.966	+	0.013
230	7.795	+	0.416	4.000	+	0.000	0.203	+	0.158	0.638	+	0.029	0.977	+	0.008
231	7.267	+	0.292	3.528	+	1.052	0.893	+	0.257	0.776	+	0.034	0.924	+	0.039
232	8.950	+	0.343	4.000	+	0.000	0.075	+	0.244	0.591	+	0.030	0.967	+	0.008
233	6.916	+	0.292	3.819	+	0.450	0.117	+	0.030	0.676	+	0.043	0.963	+	0.009
234	9.747	+	0.491	3.763	+	0.221	0.857	+	0.059	0.548	+	0.027	0.824	+	0.012
235	7.017	+	0.227	1.562	+	0.811	0.122	+	0.292	0.825	+	0.019	0.976	+	0.028
236	7.349	+	0.358	2.928	+	0.533	0.036	+	0.010	0.682	+	0.051	0.934	+	0.014
237	6.883	+	0.603	3.175	+	1.159	0.386	+	0.296	0.757	+	0.064	0.949	+	0.010
238	7.622	+	0.218	1.811	+	0.131	0.132	+	0.161	0.766	+	0.013	0.997	+	0.005
239	8.357	+	1.164	3.061	+	1.255	0.028	+	0.013	0.588	+	0.133	0.886	+	0.033
240	6.742	+	0.232	1.567	+	0.179	0.022	+	0.008	0.672	+	0.020	0.886	+	0.018
241	7.555	+	0.839	3.234	+	0.923	0.365	+	0.287	0.690	+	0.084	0.955	+	0.020
242	9.536	+	0.593	3.980	+	0.066	0.331	+	0.248	0.526	+	0.038	0.949	+	0.011
243	6.932	+	0.301	2.231	+	0.793	0.259	+	0.265	0.767	+	0.038	0.969	+	0.029
244	7.043	+	0.729	3.294	+	0.992	0.303	+	0.162	0.690	+	0.061	0.917	+	0.011
245	6.558	+	0.422	3.866	+	0.346	0.131	+	0.146	0.789	+	0.041	0.962	+	0.018
246	6.801	+	0.441	2.452	+	1.080	0.270	+	0.276	0.789	+	0.041	0.957	+	0.029
247	6.776	+	0.187	3.942	+	0.150	0.366	+	0.293	0.750	+	0.024	1.000	+	0.000
248	6.167	+	0.529	4.000	+	0.000	0.063	+	0.022	0.759	+	0.044	0.912	+	0.024
249	6.283	+	0.128	1.477	+	0.849	0.163	+	0.278	0.874	+	0.012	0.963	+	0.025
250	7.606	+	0.421	4.000	+	0.000	0.137	+	0.022	0.620	+	0.031	0.932	+	0.006
251	8.007	+	0.315	1.731	+	0.188	0.100	+	0.244	0.608	+	0.037	0.960	+	0.023
252	6.579	+	0.200	1.312	+	0.202	0.181	+	0.253	0.777	+	0.016	0.949	+	0.049
253	10.082	+	3.512	3.496	+	0.359	0.076	+	0.023	0.400	+	0.112	0.863	+	0.028
254	5.834	+	0.334	1.623	+	0.267	0.087	+	0.012	0.592	+	0.029	0.827	+	0.019
255	5.623	+	0.148	1.345	+	0.073	0.066	+	0.005	0.463	+	0.008	0.788	+	0.010
256	5.950	+	0.173	1.485	+	0.064	0.095	+	0.004	0.320	+	0.011	0.743	+	0.007
257	4.169	+	0.160	1.135	+	0.036	0.085	+	0.002	0.226	+	0.008	0.642	+	0.006
258	4.055	+	0.213	0.941	+	0.024	0.082	+	0.001	0.118	+	0.006	0.530	+	0.006
259	9.134	+	0.925	0.769	+	0.010	0.057	+	0.000	0.010	+	0.001	0.353	+	0.004

14. IMPACT analysis of the spectral density function:

Table S13: Impact coefficients obtained from the analysis of relaxation rates at five magnetic field

residue	A_1			A_2			A_3		
145	0.0059	±	0.0010	0.0000	±	0.0000	0.1683	±	0.0031
146	0.0093	±	0.0007	0.0000	±	0.0000	0.2191	±	0.0031
147	0.0091	±	0.0007	0.0000	±	0.0000	0.2489	±	0.0031
148	0.0115	±	0.0009	0.0000	±	0.0000	0.3005	±	0.0035
149	0.0118	±	0.0008	0.0000	±	0.0002	0.3615	±	0.0030
150	0.0107	±	0.0013	0.0017	±	0.0026	0.4039	±	0.0041
151	0.0112	±	0.0022	0.0131	±	0.0070	0.3813	±	0.0091
152	0.0100	±	0.0010	0.0000	±	0.0004	0.4732	±	0.0028
153	0.0078	±	0.0017	0.0305	±	0.0063	0.3993	±	0.0077
154	0.0123	±	0.0008	0.0000	±	0.0000	0.4032	±	0.0030
155	0.0108	±	0.0019	0.0129	±	0.0069	0.4485	±	0.0081
156	0.0138	±	0.0017	0.0177	±	0.0054	0.4857	±	0.0063
157	0.0087	±	0.0016	0.0167	±	0.0049	0.4488	±	0.0061
158	0.0103	±	0.0008	0.0000	±	0.0000	0.4194	±	0.0028
159	0.0031	±	0.0019	0.0286	±	0.0050	0.3654	±	0.0067
160	0.0111	±	0.0012	0.0000	±	0.0000	0.4192	±	0.0024
161	0.0074	±	0.0026	0.0086	±	0.0066	0.4036	±	0.0088
162	0.0140	±	0.0018	0.0091	±	0.0053	0.4471	±	0.0068
163	0.0094	±	0.0022	0.0303	±	0.0071	0.4328	±	0.0096
164	0.0196	±	0.0022	0.0353	±	0.0063	0.4253	±	0.0073
165	0.0302	±	0.0024	0.0604	±	0.0068	0.4075	±	0.0078
167	0.0316	±	0.0038	0.1565	±	0.0100	0.3874	±	0.0112
168	0.0334	±	0.0047	0.2008	±	0.0133	0.3942	±	0.0144
169	0.0565	±	0.0060	0.2108	±	0.0129	0.3785	±	0.0143
171	0.0655	±	0.0085	0.2505	±	0.0152	0.4221	±	0.0163
172	0.0772	±	0.0120	0.2827	±	0.0185	0.4348	±	0.0155
173	0.1105	±	0.0150	0.1873	±	0.0213	0.5342	±	0.0155
174	0.0758	±	0.0200	0.2471	±	0.0268	0.4700	±	0.0298
175	0.0449	±	0.0129	0.2683	±	0.0225	0.4069	±	0.0224
176	0.0480	±	0.0163	0.1650	±	0.0391	0.4625	±	0.0341
177	0.0485	±	0.0083	0.1680	±	0.0178	0.5232	±	0.0168
178	0.0528	±	0.0065	0.1369	±	0.0146	0.5344	±	0.0133
179	0.0249	±	0.0059	0.2009	±	0.0150	0.4257	±	0.0161
180	0.0298	±	0.0034	0.1099	±	0.0108	0.5293	±	0.0107
181	0.0057	±	0.0033	0.1942	±	0.0135	0.4206	±	0.0138
183	0.0107	±	0.0041	0.1144	±	0.0127	0.4097	±	0.0136
184	0.0107	±	0.0073	0.1049	±	0.0151	0.3965	±	0.0169
185	0.0063	±	0.0028	0.0648	±	0.0072	0.4082	±	0.0084
187	0.0068	±	0.0037	0.1200	±	0.0103	0.4250	±	0.0115
188	0.0021	±	0.0030	0.1301	±	0.0108	0.4360	±	0.0125
189	0.0083	±	0.0061	0.1654	±	0.0152	0.3913	±	0.0158
190	0.0147	±	0.0035	0.1219	±	0.0104	0.4625	±	0.0114
192	0.0139	±	0.0026	0.1227	±	0.0088	0.4131	±	0.0087
193	0.0154	±	0.0031	0.1055	±	0.0090	0.4507	±	0.0102
194	0.0211	±	0.0035	0.1285	±	0.0096	0.3999	±	0.0103
195	0.0236	±	0.0095	0.1255	±	0.0139	0.3857	±	0.0143
197	0.0224	±	0.0038	0.1328	±	0.0098	0.4335	±	0.0100
198	0.0023	±	0.0026	0.1845	±	0.0086	0.3827	±	0.0095
199	0.0103	±	0.0019	0.1552	±	0.0061	0.3905	±	0.0065
200	0.0302	±	0.0019	0.0670	±	0.0058	0.4721	±	0.0094
201	0.0000	±	0.0003	0.2058	±	0.0060	0.3567	±	0.0068
202	0.0208	±	0.0023	0.1756	±	0.0071	0.4181	±	0.0073
204	0.0133	±	0.0033	0.2306	±	0.0091	0.4116	±	0.0089
205	0.0016	±	0.0027	0.3675	±	0.0104	0.3268	±	0.0086
206	0.0434	±	0.0054	0.2793	±	0.0121	0.3571	±	0.0118
207	0.0589	±	0.0056	0.4684	±	0.0105	0.1542	±	0.0097
208	0.0642	±	0.0144	0.7322	±	0.0164	0.1128	±	0.0099
209	0.1298	±	0.0122	0.6890	±	0.0148	0.1224	±	0.0090
210	0.1448	±	0.0102	0.6560	±	0.0118	0.1154	±	0.0082

211	0.1035	±	0.0134	0.6929	±	0.0174	0.1008	±	0.0104
212	0.1335	±	0.0133	0.6356	±	0.0165	0.1382	±	0.0096
213	0.1107	±	0.0083	0.7140	±	0.0144	0.0810	±	0.0112
214	0.1239	±	0.0124	0.7171	±	0.0169	0.0789	±	0.0128
215	0.1167	±	0.0131	0.6677	±	0.0180	0.1382	±	0.0107
216	0.1048	±	0.0160	0.7627	±	0.0258	0.0726	±	0.0172
217	0.1228	±	0.0101	0.7142	±	0.0164	0.1054	±	0.0126
218	0.1160	±	0.0170	0.6802	±	0.0217	0.0909	±	0.0168
219	0.1058	±	0.0397	0.7056	±	0.0340	0.1104	±	0.0212
220	0.1081	±	0.0156	0.7012	±	0.0186	0.1106	±	0.0122
221	0.1288	±	0.0174	0.5768	±	0.0196	0.1500	±	0.0122
222	0.0804	±	0.0094	0.6904	±	0.0150	0.0990	±	0.0112
223	0.1627	±	0.0274	0.3527	±	0.0313	0.3045	±	0.0179
224	0.0935	±	0.0161	0.6718	±	0.0246	0.1463	±	0.0204
225	0.2743	±	0.0405	0.0390	±	0.0301	0.4917	±	0.0237
226	0.0312	±	0.0089	0.6241	±	0.0160	0.1584	±	0.0127
227	0.1187	±	0.0251	0.7024	±	0.0265	0.1339	±	0.0175
228	0.1137	±	0.0108	0.6821	±	0.0145	0.1337	±	0.0101
229	0.0931	±	0.0140	0.7396	±	0.0264	0.1168	±	0.0166
230	0.0763	±	0.0141	0.7738	±	0.0182	0.1149	±	0.0116
231	0.1286	±	0.0110	0.6984	±	0.0160	0.1111	±	0.0124
232	0.1134	±	0.0078	0.7364	±	0.0125	0.1065	±	0.0078
233	0.0561	±	0.0100	0.8025	±	0.0163	0.1097	±	0.0106
234	0.1576	±	0.0095	0.5037	±	0.0127	0.2476	±	0.0121
235	0.1027	±	0.0073	0.7114	±	0.0126	0.0844	±	0.0099
236	0.0799	±	0.0073	0.7093	±	0.0104	0.1209	±	0.0067
237	0.0653	±	0.0154	0.7745	±	0.0222	0.0810	±	0.0172
238	0.1218	±	0.0135	0.6764	±	0.0186	0.1413	±	0.0125
239	0.0904	±	0.0097	0.6528	±	0.0154	0.1153	±	0.0102
240	0.0657	±	0.0082	0.6036	±	0.0144	0.1589	±	0.0113
241	0.0960	±	0.0320	0.7170	±	0.0263	0.1286	±	0.0180
242	0.1225	±	0.0155	0.6757	±	0.0184	0.1621	±	0.0121
243	0.0828	±	0.0102	0.7186	±	0.0144	0.1368	±	0.0097
244	0.0601	±	0.0165	0.7330	±	0.0227	0.1054	±	0.0185
245	0.0718	±	0.0196	0.8275	±	0.0251	0.0555	±	0.0155
246	0.0939	±	0.0255	0.7613	±	0.0277	0.0743	±	0.0217
247	0.0762	±	0.0097	0.8198	±	0.0168	0.0994	±	0.0100
248	0.0481	±	0.0285	0.7774	±	0.0368	0.0739	±	0.0238
249	0.0628	±	0.0107	0.7885	±	0.0162	0.0528	±	0.0126
250	0.0779	±	0.0105	0.7316	±	0.0156	0.1203	±	0.0110
251	0.0995	±	0.0177	0.5416	±	0.0361	0.2589	±	0.0240
252	0.0761	±	0.0155	0.6886	±	0.0253	0.1238	±	0.0197
253	0.0845	±	0.0183	0.5823	±	0.0322	0.2234	±	0.0235
254	0.0268	±	0.0108	0.5687	±	0.0190	0.1989	±	0.0180
255	0.0130	±	0.0041	0.4331	±	0.0103	0.2775	±	0.0104
256	0.0183	±	0.0030	0.2884	±	0.0084	0.3784	±	0.0087
257	0.0001	±	0.0004	0.1632	±	0.0042	0.3606	±	0.0055
258	0.0019	±	0.0013	0.0623	±	0.0041	0.2810	±	0.0050
259	0.0014	±	0.0004	0.0000	±	0.0000	0.1558	±	0.0020

Table S13: Impact coefficients obtained from the analysis of relaxation rates at five magnetic field (continued)

residue	A_4			A_5			A_6		
145	0.3742	±	0.0107	0.3030	±	0.0161	0.1486	±	0.0091
146	0.4384	±	0.0118	0.1608	±	0.0181	0.1724	±	0.0095
147	0.5096	±	0.0118	0.0310	±	0.0184	0.2013	±	0.0101
148	0.4160	±	0.0124	0.0877	±	0.0185	0.1843	±	0.0100
149	0.2940	±	0.0110	0.1834	±	0.0170	0.1493	±	0.0094
150	0.2469	±	0.0114	0.2503	±	0.0182	0.0865	±	0.0101
151	0.2672	±	0.0146	0.1712	±	0.0220	0.1559	±	0.0130
152	0.1815	±	0.0090	0.2524	±	0.0143	0.0829	±	0.0082
153	0.2642	±	0.0114	0.1559	±	0.0180	0.1423	±	0.0104
154	0.3910	±	0.0077	0.0001	±	0.0010	0.1934	±	0.0057
155	0.2251	±	0.0147	0.1690	±	0.0204	0.1338	±	0.0100
156	0.2061	±	0.0113	0.2072	±	0.0169	0.0695	±	0.0090
157	0.2747	±	0.0117	0.1354	±	0.0183	0.1158	±	0.0101
158	0.2719	±	0.0100	0.1550	±	0.0162	0.1434	±	0.0094
159	0.2810	±	0.0129	0.1784	±	0.0187	0.1434	±	0.0102
160	0.2202	±	0.0089	0.1798	±	0.0143	0.1696	±	0.0082
161	0.2318	±	0.0162	0.2037	±	0.0233	0.1449	±	0.0125
162	0.1573	±	0.0148	0.2930	±	0.0209	0.0794	±	0.0107
163	0.1554	±	0.0146	0.2715	±	0.0231	0.1006	±	0.0134
164	0.2408	±	0.0148	0.1378	±	0.0230	0.1411	±	0.0127
165	0.1324	±	0.0161	0.2824	±	0.0250	0.0873	±	0.0137
167	0.1899	±	0.0229	0.1448	±	0.0385	0.0898	±	0.0223
168	0.1385	±	0.0284	0.1949	±	0.0455	0.0381	±	0.0258
169	0.1370	±	0.0301	0.1260	±	0.0518	0.0911	±	0.0305
171	0.0826	±	0.0332	0.1073	±	0.0600	0.0719	±	0.0368
172	0.0028	±	0.0087	0.1766	±	0.0299	0.0259	±	0.0261
173	0.0000	±	0.0000	0.0007	±	0.0044	0.1673	±	0.0127
174	0.0797	±	0.0378	0.0801	±	0.0633	0.0472	±	0.0408
175	0.1021	±	0.0359	0.1595	±	0.0511	0.0183	±	0.0259
176	0.0971	±	0.0432	0.0602	±	0.0581	0.1672	±	0.0337
177	0.0241	±	0.0262	0.2159	±	0.0387	0.0204	±	0.0222
178	0.0124	±	0.0164	0.2494	±	0.0241	0.0141	±	0.0149
179	0.1503	±	0.0270	0.0729	±	0.0378	0.1253	±	0.0196
180	0.0679	±	0.0177	0.1996	±	0.0296	0.0636	±	0.0173
181	0.1392	±	0.0283	0.1528	±	0.0399	0.0876	±	0.0194
183	0.2382	±	0.0212	0.0484	±	0.0308	0.1787	±	0.0165
184	0.1620	±	0.0288	0.1682	±	0.0422	0.1577	±	0.0223
185	0.1396	±	0.0150	0.2536	±	0.0227	0.1275	±	0.0125
187	0.0967	±	0.0212	0.2876	±	0.0318	0.0639	±	0.0173
188	0.1470	±	0.0226	0.1882	±	0.0327	0.0965	±	0.0172
189	0.2702	±	0.0228	0.0264	±	0.0303	0.1384	±	0.0175
190	0.1150	±	0.0201	0.2636	±	0.0299	0.0224	±	0.0161
192	0.1949	±	0.0140	0.1340	±	0.0209	0.1215	±	0.0114
193	0.1459	±	0.0185	0.2159	±	0.0276	0.0666	±	0.0151
194	0.2536	±	0.0168	0.0264	±	0.0236	0.1705	±	0.0126
195	0.1934	±	0.0242	0.1340	±	0.0350	0.1379	±	0.0202
197	0.1109	±	0.0154	0.2629	±	0.0234	0.0374	±	0.0134
198	0.1694	±	0.0166	0.1488	±	0.0272	0.1124	±	0.0157
199	0.2102	±	0.0120	0.1165	±	0.0183	0.1173	±	0.0100
200	0.1323	±	0.0297	0.1703	±	0.0425	0.1281	±	0.0210
201	0.1694	±	0.0148	0.1803	±	0.0237	0.0877	±	0.0140
202	0.1025	±	0.0128	0.2252	±	0.0213	0.0578	±	0.0123
204	0.1339	±	0.0157	0.1251	±	0.0262	0.0855	±	0.0153
205	0.0000	±	0.0000	0.1990	±	0.0178	0.1051	±	0.0177
206	0.0548	±	0.0206	0.1458	±	0.0311	0.1197	±	0.0178
207	0.0344	±	0.0161	0.0570	±	0.0261	0.2271	±	0.0153
208	0.0001	±	0.0006	0.0003	±	0.0018	0.0905	±	0.0112
209	0.0000	±	0.0000	0.0000	±	0.0003	0.0587	±	0.0088
210	0.0039	±	0.0049	0.0000	±	0.0000	0.0799	±	0.0081
211	0.0000	±	0.0004	0.0060	±	0.0086	0.0968	±	0.0141
212	0.0000	±	0.0003	0.0001	±	0.0008	0.0926	±	0.0092

213	0.0457	±	0.0077	0.0000	±	0.0005	0.0486	±	0.0087
214	0.0161	±	0.0137	0.0203	±	0.0212	0.0437	±	0.0176
215	0.0007	±	0.0027	0.0005	±	0.0024	0.0762	±	0.0103
216	0.0061	±	0.0099	0.0171	±	0.0177	0.0367	±	0.0188
217	0.0111	±	0.0097	0.0002	±	0.0024	0.0463	±	0.0101
218	0.0198	±	0.0133	0.0098	±	0.0162	0.0833	±	0.0201
219	0.0000	±	0.0002	0.0228	±	0.0256	0.0553	±	0.0336
220	0.0016	±	0.0044	0.0084	±	0.0133	0.0701	±	0.0165
221	0.0008	±	0.0029	0.0003	±	0.0021	0.1433	±	0.0131
222	0.0173	±	0.0121	0.0148	±	0.0191	0.0982	±	0.0148
223	0.0005	±	0.0032	0.0001	±	0.0010	0.1795	±	0.0191
224	0.0470	±	0.0144	0.0000	±	0.0000	0.0414	±	0.0163
225	0.0081	±	0.0118	0.0001	±	0.0020	0.1868	±	0.0310
226	0.0204	±	0.0096	0.0007	±	0.0039	0.1652	±	0.0103
227	0.0058	±	0.0093	0.0125	±	0.0143	0.0266	±	0.0208
228	0.0058	±	0.0063	0.0000	±	0.0002	0.0647	±	0.0094
229	0.0000	±	0.0001	0.0323	±	0.0148	0.0182	±	0.0168
230	0.0007	±	0.0029	0.0056	±	0.0092	0.0287	±	0.0123
231	0.0274	±	0.0094	0.0000	±	0.0000	0.0344	±	0.0106
232	0.0000	±	0.0000	0.0000	±	0.0000	0.0437	±	0.0057
233	0.0000	±	0.0005	0.0160	±	0.0128	0.0156	±	0.0129
234	0.0323	±	0.0118	0.0000	±	0.0000	0.0588	±	0.0104
235	0.0237	±	0.0072	0.0000	±	0.0003	0.0778	±	0.0078
236	0.0003	±	0.0013	0.0022	±	0.0047	0.0875	±	0.0070
237	0.0250	±	0.0145	0.0073	±	0.0154	0.0468	±	0.0173
238	0.0049	±	0.0068	0.0021	±	0.0075	0.0535	±	0.0131
239	0.0041	±	0.0060	0.0001	±	0.0009	0.1373	±	0.0085
240	0.0111	±	0.0093	0.0025	±	0.0078	0.1583	±	0.0102
241	0.0072	±	0.0107	0.0057	±	0.0120	0.0455	±	0.0260
242	0.0022	±	0.0056	0.0266	±	0.0141	0.0108	±	0.0133
243	0.0020	±	0.0044	0.0028	±	0.0066	0.0570	±	0.0100
244	0.0365	±	0.0230	0.0421	±	0.0328	0.0229	±	0.0203
245	0.0007	±	0.0033	0.0119	±	0.0149	0.0327	±	0.0192
246	0.0264	±	0.0202	0.0278	±	0.0276	0.0163	±	0.0203
247	0.0002	±	0.0014	0.0001	±	0.0009	0.0043	±	0.0056
248	0.0025	±	0.0084	0.0500	±	0.0309	0.0481	±	0.0328
249	0.0143	±	0.0101	0.0007	±	0.0037	0.0808	±	0.0105
250	0.0040	±	0.0083	0.0560	±	0.0155	0.0103	±	0.0125
251	0.0027	±	0.0059	0.0000	±	0.0004	0.0972	±	0.0141
252	0.0349	±	0.0206	0.0209	±	0.0292	0.0556	±	0.0225
253	0.0082	±	0.0178	0.0706	±	0.0335	0.0310	±	0.0285
254	0.0378	±	0.0283	0.1434	±	0.0434	0.0244	±	0.0250
255	0.0863	±	0.0187	0.0823	±	0.0324	0.1077	±	0.0194
256	0.0961	±	0.0155	0.1639	±	0.0257	0.0550	±	0.0147
257	0.1879	±	0.0114	0.2021	±	0.0189	0.0861	±	0.0107
258	0.2628	±	0.0111	0.2715	±	0.0158	0.1205	±	0.0082
259	0.2799	±	0.0079	0.2184	±	0.0115	0.3444	±	0.0059

15. Reference:

1. Farrow. N. A.. O. W. Zhang. A. Szabo. D. A. Torchia. and L. E. Kay. 1995. Spectral Density-Function Mapping Using N-15 Relaxation Data Exclusively. J. Biomol. NMR 6:153-162.
